# Methodological framework for the surveillance of healthcare-associated infections in high-risk infants: the NeoIPC surveillance core module protocol

**DOI:** 10.1186/s13756-026-01711-0

**Published:** 2026-02-19

**Authors:** Gizem Karadağ, Adrie Bekker, Angela Dramowski, Begoña Loureiro Gonzalez, Javier Pérez López, Jose I. Pijoan Zubizarreta, Paul T. Heath, Seven Johannes Sam Aghdassi, Brar Piening

**Affiliations:** 1 Institute of Hygiene and Environmental Medicine, Charité – Universitätsmedizin Berlin, corporate member of Freie Universität Berlin and Humboldt Universität zu Berlin, Berlin, Germany; 2 Department of Pediatrics and Child Health, Faculty of Medicine and Health Sciences, Stellenbosch University, Cape Town, Republic of South Africa; 3 Department of Pediatrics, Cruces University Hospital, Barakaldo, Bizkaia Spain; 4 Biobizkaia Health Research Institute, Barakaldo, Bizkaia Spain; 5 CIBERESP (CIBER of Epidemiology and Public Health), Madrid, Spain; 6 Centre for Neonatal & Pediatric Infection, Institute of Infection & Immunity, City St. George’s, University of London, London, UK

**Keywords:** Healthcare-associated infections, Neonatal intensive care units, Very low birth weight infant, Surveillance systems, Infection prevention and control, Multidrug-resistant organisms

## Abstract

**Background:**

Healthcare-associated infections pose a significant risk to high-risk infants, particularly those with very low birth weight (< 1500 g) and those born very preterm (< 32 weeks gestation). The burden of healthcare-associated infections, contributing risk factors, and efficacy of prevention strategies in the neonatal population remain underinvestigated, with few national and international networks.

**Methods:**

To address this gap, an international team of experts in neonatology, epidemiology, and infection prevention and control from diverse healthcare settings collaborated as consortium partners within the NeoIPC Project to design a surveillance program focused on healthcare-associated infections and multidrug-resistant organisms in neonatal units. Data collection includes the most prevalent neonatal healthcare-associated infections (primary and secondary bloodstream infections, clinical sepsis, pneumonia, necrotizing enterocolitis, and surgical site infections), the presence of multidrug-resistant bacteria and associated risk and protective factors for healthcare-associated infections.

**Discussion:**

By providing standardized methods and reference data for benchmarking, the NeoIPC Surveillance aims to support infection prevention and antibiotic stewardship programs, improve neonatal care outcomes and foster international collaboration. This article outlines the methods of the NeoIPC Surveillance Core Module (Version 1.2), details data collection, management, and analysis, and serves as a comprehensive reference for healthcare professionals and researchers worldwide aiming to implement effective surveillance for healthcare-associated infections in neonatal units.

**Supplementary Information:**

The online version contains supplementary material available at 10.1186/s13756-026-01711-0.

## Introduction

Healthcare-associated infections (HAIs) in very low birth weight (VLBW; <1500 g) and very preterm (VPT; <32 weeks’ gestation) infants represent a major challenge in neonatal units due to the vulnerability of this population [1, 2]. These high-risk infants are more susceptible to infection owing to their immature immune systems, extended hospital stays, and frequent exposure to invasive procedures and surgeries [3–5]. The associated burden of HAIs in the neonatal period is substantial, given that they occur at an early stage of life, potentially leading to a high number of years of life lost or years lived with disability [6].

Systematic HAI surveillance provides a critical foundation for identifying infection risks, monitoring trends over time and evaluating infection prevention strategies, which are key components shown to contribute to reduced HAI rates in neonatal care settings [7, 8]. In neonatal populations, infection risk is shaped not only by clinical and procedural risk factors but also by protective practices that may mitigate the occurrence of HAIs; however, such protective factors are rarely captured in existing neonatal HAI surveillance systems. Their inclusion is essential to enable a more comprehensive interpretation of infection trends and to support the evaluation of preventive strategies in routine neonatal care.

Systematic surveillance further implies not only effective monitoring of HAIs at the level of an individual healthcare facility but also benchmarking with comparable data from other institutions, ideally within a national or international surveillance network. However, widely adopted HAI surveillance programs are not available in most countries. Where national and supranational (i.e., involving more than one country) surveillance systems exist, they frequently have a broad approach with limited ability to capture neonatal-specific infection prevention and control (IPC) data. The current landscape is characterized by either a lack of or fragmentation of surveillance programs, each with its own methodologies and data collection practices, with little information on the compatibility of the tools and methods employed and the comparability of the data collected [9–11]. Moreover, existing national and supranational programs for neonatal HAI surveillance are typically located in high-income settings [12, 13] and do not adequately incorporate the specific surveillance needs of low- and middle-income countries (LMICs), further increasing the heterogeneity of global neonatal HAI reporting.

The NeoIPC Project, which is part of the European Union’s Horizon 2020 research and innovation program, unites a team of international experts to develop and implement new strategies for preventing HAI and multidrug-resistant organism (MDRO) infections in neonatal care [14]. Led by the University of Padova (Italy), the consortium includes fourteen partner institutions from Europe and Africa, including Fondazione Penta ETS (Italy), City St. George’s University of London (United Kingdom), Universitair Medisch Centrum Utrecht (Netherlands), the European Clinical Research Alliance for Infectious Diseases (Netherlands), Universität Zürich (Switzerland), Universitäts-Kinderspital beider Basel (Switzerland), Tartu Ülikool (Estonia), Schweizerisches Tropen und Public Health Institut (Switzerland), Aristotelio Panepistimio Thessalonikis (Greece), Universiteit Antwerpen (Belgium), Charité – Universitätsmedizin Berlin (Germany), Osakidetza – Servicio Vasco de Salud (Spain), and Stellenbosch University (Republic of South Africa). By integrating clinical and laboratory research, implementation science, and surveillance, the NeoIPC Project aims to strengthen IPC practices and improve health outcomes for high-risk infants (VLBW and VPT). This article describes the methods of the NeoIPC Surveillance Core Module, a surveillance program for HAI and MDRO infections developed as part of the NeoIPC Project and led by Charité – Universitätsmedizin Berlin [15].

### NeoIPC surveillance: insights from the development stage

The development of the NeoIPC Surveillance methods began with an investigation into the methodological approaches employed in current surveillance networks for high-risk infants. The initial identification process was conducted using electronic literature databases, web search engines, expert interviews, and cross-referencing of recent publications to identify relevant surveillance networks. Information was extracted from protocols and additional resources such as manuals, patient data booklets, annual reports, point prevalence survey (PPS) reports, study protocols, and review articles into a standardized dataset, which served as the basis for subsequent comparisons. As the main objective was to collect information on datasets and methods of surveillance networks related to HAIs, with a particular focus on MDRO infections, elements such as congenital malformations, prenatal risk factors, and other unrelated indicators were excluded. Ultimately, 19 surveillance networks and one PPS were identified at the national and supranational levels that focused on or included high-risk infants (Table 1). Methodologies employed across these networks were reviewed to identify overlaps with the aim of assessing data sharing and integration, as well as identifying key gaps that could inform targeted improvement strategies. The review identified notable differences among existing surveillance networks, which pose major challenges to effective benchmarking and data aggregation. However, some similarities suggest potential opportunities for collaboration across networks. Conversely, a lack of consensus on risk standardization and surveillance definition criteria limits the feasibility of applying these neonatal infection surveillance programs in LMICs. The lack of accessible and freely available HAI surveillance tools and the insufficient focus on HAIs and antimicrobial resistance (AMR) in neonatology remain critical barriers to LMIC participation in large surveillance networks.

**Table 1:** Overview of reviewed networks performing infection surveillance in neonatology and their focus

Network	Countries	Population	Focus
*National networks*			
National Healthcare Safety Network (NHSN) [16]	USA	Infants of all birth weights (LOS/MEN module only VLBW)	HAI and AMR
NEO-KISS [17, 18]	Germany and Austria	VLBW/ELBW infants	Neonatal infection surveillance with HAI and AMR focus
NeoKissEs [19, 20]	Spain	VLBW/ELBW infants	Neonatal infection surveillance with healthcare-associated sepsis focus
Belgian surveillance system for VLBW infants	Belgium	VLBW/ELBW infants	Neonatal infection surveillance with HAI focus
Neonatal Research Network (NRN) [21, 22]	USA	ELBW/EPT infants	Morbidity and mortality among high-risk preterm infants
Canadian Neonatal Network (CNN) [23]	Canada	Infants of all birth weights (some modules only VLBW/VPT)	Morbidity and mortality among preterm/term infants
Neonatal Research Network Japan (NRNJ) [24, 25]	Japan	VLBW/VPT infants	Morbidity and mortality among VLBW/VPT infants
Infection in Critical Care Quality Improvement Programme (ICCQIP) [26]	England	Infants of all birth weights	Bloodstream infections (BSI)
Dutch neonatal central line-associated bloodstream infection surveillance criteria (NL CLABSI SC) [10]	Netherlands	Infants of all birth weights	Neonatal CLABSI
The Swiss Neonatal Network & Follow-up Group (SwissNeoNet) [27, 28]	Switzerland	VLBW/VPT infants	Morbidity and mortality among VLBW/VPT infants
Israel Neonatal Network (INN) [29, 30]	Israel	VLBW/VPT infants	Morbidity and mortality among VLBW/VPT infants
Polish Neonatology Surveillance Network (PNSN) [31, 32, 33]	Poland	VLBW infants	Neonatal infection surveillance with HAI focus
National Neonatal Audit Programme (NNAP) [34]	UK	Infants of all birth weights	Morbidity and mortality among preterm/term infants
*Supranational networks*			
International Nosocomial Infection Control Consortium (INICC) [35]	66 countries	Infants of all birth weights	HAI and AMR
Vermont Oxford Network (VON) [36]	34 countries	VLBW infants (Expanded version: Infants of all birthweights)	Morbidity and mortality among Preterm infants
European Neonatal Network (EuroNeoNet) [37]	18 countries	VLBW/VPT infants	Morbidity and mortality among VLBW/VPT infants
International Network for Evaluation of Outcomes (iNeo) [38, 39, 40]	10 countries	VLBW/VPT infants	Morbidity and mortality among VLBW/VPT infants
Australian and New Zealand Neonatal Network (ANZNN) [41]	2 countries	VLBW/VPT or high-risk infants	Morbidity and mortality among VLBW/VPT or high-risk infants
Neonatal Infection Surveillance Network (neonIN) [42, 43]	5 countries	Infants of all birth weights	Neonatal infection surveillance with HAI and AMR focus
ECDC Point prevalence survey (ECDC-PPS) [44]	Europe	Infants of all birth weights	HAI and AMR

LOS, late-onset sepsis; MEN, meningitis; VLBW, very low birth weight; HAI, healthcare-associated infection; AMR, antimicrobial resistance; ELBW, extremely low birth weight; EPT, extremely preterm; VPT, very preterm; BSI, bloodstream infection; CLABSI, central line-associated bloodstream infection; ECDC, European Centre for Disease Prevention and Control; PPS, point prevalence survey.

In light of these findings, an international team of experts within the NeoIPC Project consortium, comprising neonatologists, epidemiologists, and IPC specialists, collaborated to establish a robust but feasible surveillance toolkit that would be applicable across diverse neonatal care settings globally. The NeoIPC Surveillance Core Module was developed to monitor HAI rates and MDRO infections, as well as risk factors and protective factors for HAI in hospitalized very preterm and very low-birth-weight infants.

### Rationale

NeoIPC Surveillance was established to facilitate access to a tailored HAI surveillance system for high-risk infants and to improve data harmonization and comparability through regional and international collaboration. The system is designed to be accessible and user-friendly, ensuring a low barrier to entry and encouraging widespread adoption. It enables healthcare professionals to generate reliable, comparable data on HAIs and AMR, thereby improving the ability to measure HAI burden in their neonatal unit, identify risk factors for infection, and evaluate the effectiveness of IPC intervention strategies. A harmonized surveillance system will not only support individual hospitals in improving neonatal outcomes but also contribute to the creation of a robust global network for HAI surveillance for high-risk infants.

The project aims to support collaborative sharing and learning, examine variations across countries and networks, identify evidence-based practices associated with improved outcomes, and support their implementation to improve outcomes for vulnerable infants. In collaboration with the NeoIPC Clinical Practice Network, a global network that connects healthcare professionals in neonatology, the NeoIPC Surveillance toolkit and network could serve as powerful tools for benchmarking neonatal HAI rates, fostering best practices, and ultimately reducing morbidity and mortality rates in neonatal care [45]. This paper presents the methodological framework of NeoIPC Surveillance with detailed guidelines for data collection, management, and analysis.

## Methods

This section provides insights into the data collection, analysis, submission, and management procedures. It includes specific details on eligibility and follow-up criteria, surveillance definitions for infections, and an overview of additional data elements. More detailed information, including a comprehensive data dictionary that defines each element in the dataset, can be found in the surveillance protocol [see Additional File 1]. In addition, a structured evaluation survey has been initiated to invite LMIC sites to pilot the surveillance system and provide systematic feedback on feasibility, resource requirements, and implementation barriers. The survey was developed based on the Consolidated Framework for Implementation Research (CFIR) [46, 47] and is provided in the supplementary materials [see Additional File 2].

### Data collection

Data for NeoIPC Surveillance are collected locally in participating institutions, typically by IPC staff; however, neonatology staff may also carry out this task in some settings. To ensure data accuracy and clarify unclear or incomplete chart entries, data collection involves reviewing clinical charts and engaging with clinical staff involved in patient care. This process captures the relevant elements of the surveillance dataset, as outlined in the risk stratification variables and outcomes: healthcare-associated infections and MDRO sections. All collected data must be pseudonymized before entry into the online data submission platform to ensure compliance with data privacy standards. To facilitate this process, data collection sheets and templates for pseudonymization are provided as part of a toolkit for participants. These documents, which may include patient identifiers, are used solely as aids for data collection and are not submitted to the NeoIPC data platform [see Additional File 3].

#### Patient eligibility and follow-up criteria

**Fig. 1:**
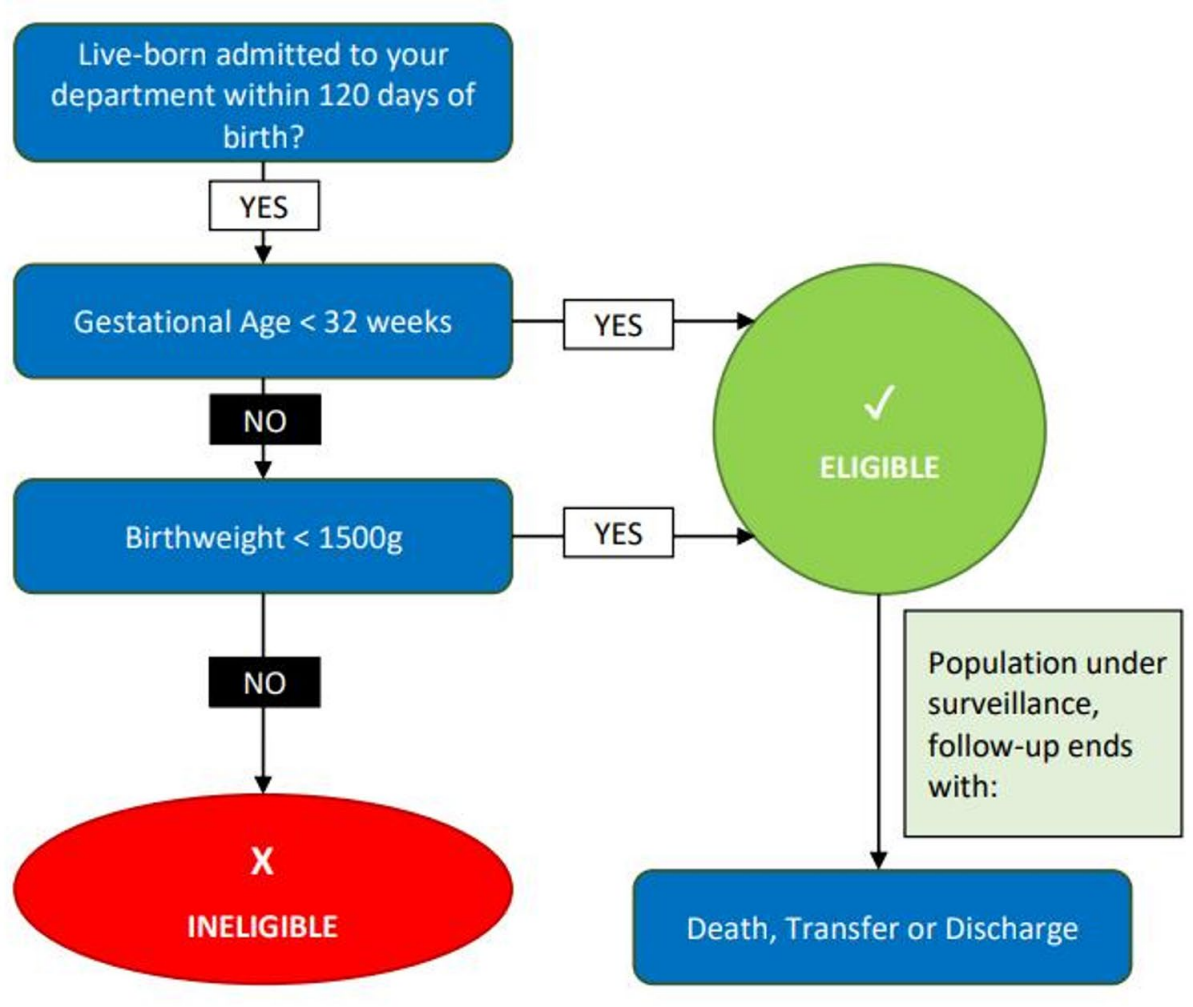
Patient eligibility and follow-up criteria

All the neonatology units that regularly admit VLBW and VPT infants can participate in the network. Any live-born infant admitted to the unit within 120 days of birth with a birth weight of less than 1500 g and/or a gestational age of less than 32 weeks is eligible for inclusion in the NeoIPC Core Module. Stillborn infants and delivery room deaths are excluded. The surveillance follow-up period is terminated in cases of death, discharge or transfer of the patient from the participating facility, except for short absences of up to 48 h (Fig. 1).

#### Risk stratification variables

To structure the NeoIPC Surveillance dataset, the collected variables were grouped according to their primary function in the surveillance system. Denominator data capture the exposure time or procedural counts necessary for calculating incidence rates, whereas risk stratification data describe patient- or procedure-related characteristics that allow for adjustment of infection rates on the basis of individual risk profiles. This approach ensures consistent calculation of infection densities and enables meaningful comparisons across different patient groups and healthcare settings. An overview of the variables assigned to each category is provided in Table 2.

**Table 2:** Overview of risk-associated data elements

Data element	Surveillance definition
*Inherent factors*	
Gestational age	The gestational age, expressed in completed weeks and days (e.g., 25 weeks and 4 days: 25 + 4) at the time of birth.
Birth weight	The infant’s weight immediately after birth in grams.
Sex	Typically, the phenotypic sex of the patient. If sex cannot be determined from the patient’s phenotype or genotype, or if the genotype is neither XX nor XY, it is considered undetermined for purposes of surveillance.
Delivery mode	One of the following: • Vaginal (including assisted vaginal delivery) • Elective cesarean section • Emergency cesarean section
Multiple birth	The number of infants delivered from the (multiple) pregnancy this infant belongs to.
*Expositional and explanatory factors*	
Admission type (Day of life at admission)	One of the following: • Admitted from delivery room (initial admission for infants delivered in your hospital) • Transferred/readmitted to the unit on the day of birth • Transferred/readmitted to the unit the day after birth or later
End-of-surveillance reason	One of the following: • Discharge or transfer • Death
Patient days	Sum of days the patient remains in the unit. No minimum time is needed.
Device utilization (CVC, PVC, INV, NIV)	Sum of days the patient had a device placed for at least 12 h cumulatively.
Antibiotic use	Sum of days the infant received at least one systemic antibiotic, captured both cumulatively and per substance.
Breast milk intake	Sum of days the patient’s enteral feeding exclusively consisted of (donor milk and fortified breast milk are also included) breast milk, regardless of the amount.
Probiotic use	Sum of days the patient received an oral probiotic containing at least one of *Lactobacillus* spp. or *Bifidobacterium* spp., regardless of the amount.
Kangaroo care implementation	Sum of days the patient received kangaroo care (intensive skin-to-skin-contact) for at least 2 h.
*Surgery data (if applicable)*	
Surgery description	Brief description of the surgery.
Day of life at surgery	Day of life when surgery occurred
Surgery duration	The duration of the surgery in minutes (incision-to-suture time if available)
Main and side surgery codes	The International Classification of Health Interventions (ICHI)** code of the main procedure performed. If multiple different procedures are performed during one surgery, the surgeon decides which one is the main procedure.
ASA Score	The American Society of Anesthesiologists (ASA) Physical Status Classification System to assess and communicate a patient’s preanaesthesia medical comorbidities. [48]
Wound class	Assessment of the degree of contamination of a surgical wound at the time of the surgical procedure according to the CDC Guidelines [49].
Endoscopic procedure	Indicate if the surgery was performed endoscopically.
Emergency Procedure	Indicate if the surgery was performed as an emergency.
Revision procedure	Indicate if this surgery was a repeat or corrective procedure following a previous one.
Primary wound closure	The closure of the skin level during the original surgery
Implant	Indicate if a foreign body of nonhuman origin was permanently placed into a patient during an operation and was not routinely manipulated for diagnostic or therapeutic purposes.
Signs of infection at the time of surgery	To be completed if signs of infection were identified during the surgical procedure.

CVC, central venous catheter; PVC, peripheral venous catheter; INV, invasive ventilation; NIV, noninvasive ventilation; CDC, Centers for Disease Control and Prevention.

Risk and protective factors were selected on the basis of well-established key determinants of HAIs in high-risk infants. Medical devices such as intravenous catheters and mechanical ventilation, while essential, compromise natural barriers and increase infection risk [50–52]. Broad-spectrum antibiotics, which are commonly used in NICUs, can disrupt the neonatal microbiome and promote MDROs [53, 54]. Prolonged hospitalization further increases this risk because of extended exposure to invasive procedures and antimicrobial pressure [55–57].

Protective factors include human milk, which supports microbiome development and reduces the risk of necrotizing enterocolitis (NEC) and sepsis [51, 55, 58, 59]; probiotics, which may help prevent dysbiosis and infections [55]; and kangaroo mother care, which lowers infection risk through maternal microbiota transfer and improved physiological stability [60–62].

#### Outcomes: healthcare-associated infections and MDROs

The NeoIPC Surveillance Core Module focuses on late-onset infections, defined as infections occurring ≥ 72 h after birth, which are generally presumed to be associated with healthcare. Case definitions for HAIs were specifically adapted to the neonatal population and diverse clinical settings, considering the unique clinical presentation and diagnostic challenges in this age group, an essential consideration for accurate and meaningful neonatal HAI surveillance. The surveillance system captures five main types of HAIs: primary BSIs, including clinical sepsis and hematogenous meningitis; secondary BSIs; pneumonia; NEC; and surgical site infection (SSI). Reporting of secondary bloodstream infections is optional (Table 3). Each infection type is defined by a standardized set of diagnostic and clinical criteria that must be met for inclusion. Less common infections in infants, such as urinary tract infections (UTIs), bone and joint infections, and cardiovascular infections, were excluded from the surveillance scope due to their rarity in this population and the limited potential for meaningful statistical analysis. In addition to HAI, NeoIPC Surveillance also captures data on MDRO infections, which can be used to monitor AMR patterns and calculate MDRO-related incidence or prevalence rates. Full surveillance infection definitions are available in the appendix [see Tables A–D in Additional File 4].

**Table 3:** Outcomes

Data element	Specifics	Device/procedure- association	Infectious agents^1^
Primary bloodstream infections (BSI)/Sepsis^2^			
Clinical sepsis	Infection without a detected organism. Definition criteria based on clinical and laboratory findings.	Not device-associated, CVC or PVC-associated	n/a
Laboratory-confirmed bloodstream infection (LCBSI) - caused by a recognized pathogen^1^	Definition criteria based on solely positive laboratory result for a recognized pathogen	Not device-associated, CVC or PVC-associated	Recognized pathogens
Laboratory-confirmed bloodstream infection (LCBSI) - caused by a common commensal^1^	Definition criteria based on single or multiple positive laboratory result(s) for a common commensal, together with clinical and laboratory findings.	Not device-associated, CVC or PVC-associated	Common commensals
Secondary bloodstream infection (optional)	LCBSI secondary to pneumonia, NEC or SSI.	n/a	All infectious agents
Pneumonia	Definition criteria based on clinical, imaging, and laboratory findings.	Not device-associated, INV or NIV-associated	All infectious agents
Necrotizing enterocolitis (NEC)	Definition criteria based on clinical and radiological findings or a diagnosis based on surgical evidence.	n/a	n/a
Surgical site infections (SSI)			
Superficial incisional SSI	Infection involving only skin and subcutaneous tissue.	Surgery-associated	All infectious agents
Deep incisional SSI	Infection involving deeper soft tissues (e.g., fascia, muscle).	Surgery-associated	All infectious agents
Organ/Space SSI	Infection involving organs or spaces manipulated during surgery.	Surgery-associated	All infectious agents

BSI, bloodstream infection; LCBSI, laboratory-confirmed bloodstream infection; CVC, central venous catheter; PVC, peripheral venous catheter; INV, invasive ventilation; NIV, noninvasive ventilation; NEC, necrotizing enterocolitis; SSI, surgical site infection; n/a, not applicable.^1^ Includes MDROs where applicable, such as methicillin-resistant *Staphylococcus aureus* (MRSA), vancomycin-resistant enterococci (VREs), third-generation cephalosporin-resistant (3GCR) organisms, and pathogens resistant to carbapenems or colistin. [See the NeoIPC infectious agent list in additional file 5].^2^Includes hematogenous meningitis.

NeoIPC Surveillance employs a time-based criterion for establishing an association between a device and an infection. Device association was assessed for selected HAIs, including pneumonia (in relation to invasive or noninvasive mechanical ventilation) and sepsis or primary bloodstream infections (associated with central or peripheral venous catheters). A HAI was classified as device-associated if the respective device had been in use continuously for at least three days, either on the day of infection onset or the day immediately preceding it. For SSIs, specific postoperative time frames were used to determine their association with a surgical procedure. A wound infection at the incision or surgical site is classified as an SSI if the first symptoms occur within 30 days following surgery. If an implant is placed, the surveillance period extends to 90 days for deep incisional and organ/space SSIs.

The guidelines do not rigidly specify the timeframe within which components of the infection criteria must manifest. However, all requisite elements must typically occur within a period of 7–10 days, with no more than 2–3 days between them, to be considered part of the same infection episode. Moreover, in the presence of a previously recorded infection, when a new pathogen is isolated in the same organ system, it cannot be automatically documented as a new infection. Starting from the date of infection, at least 14 days must pass, including a symptom-free period, before the same type of infection can be registered again. The infection date was defined as the first day on which symptoms appeared. However, if a positive test result from the primary site of infection is obtained before symptom onset, that earlier date is recorded as the infection date.

### Data analysis, benchmarking and reporting

Data analysis in the NeoIPC Surveillance system is conducted at two levels: the unit level and the aggregated reference level. At the unit level, individual units can analyze their own data via integrated reporting tools. The aggregated reference level involves compiling data from all participating units on an annual basis to produce benchmark reports for comparison. Data analysis is typically stratified into four birth weight groups: <500 g, 500–999 g, 1000–1499 g, and ≥ 1500 g. The last group includes a small subset of VPT infants who meet the gestational age criterion but do not fall under the VLBW category.

#### Risk and protective factor use rates

Risk factors refer to the presence of medical devices (i.e., central and peripheral lines, mechanical ventilation) that increase the likelihood of developing HAIs, whereas protective factors (i.e., human milk feeding, probiotic use, and kangaroo care) are associated with a reduced risk. The rate of risk or protective factor use is defined as the percentage of patient surveillance days during which the respective factor was present for at least 12 h. It is calculated by dividing the number of total days in which risk or protective factors are used by the number of patient days under surveillance and multiplying the result by 100.

$$ \begin{gathered} \:Risk\:or\:Protective\:Factor\:Use\:Rate \hfill \\ \quad \quad = \frac{{Total\:days\:risk\:or\:protective\:factor\:was\:in\:use}}{{Total\:patient\:days}} \times \:100 \hfill \\ \end{gathered} $$

#### Infectious agent detection rates

Understanding which agents cause HAI in high-risk infants is challenging and can be influenced by several biases. In general, small blood volumes taken from infants reduce the sensitivity of diagnostic tests, and contamination can lower their specificity. These problems are even more pronounced in settings with limited resources, where microbiological diagnostics may not be fully available or where financial constraints affect testing practices and detection rates.

$$ \begin{gathered} Infectious\:agent\:detection\:rate \hfill \\ \quad \quad = \frac{{Number\:of\:infections\:with\:agent}}{{Number\:of\:infections}} \times \:100 \hfill \\ \end{gathered} $$

$$ \begin{gathered} \:Agent - per - Infection\:rate \hfill \\ \quad = \frac{{Number\:of\:infectious\:agents\:that\:were\:detected\:in\:infections}}{{Number\:of\:infections\:where\:an\:infectious\:agent\:was\:detected}} \times \:100 \hfill \\ \end{gathered} $$

#### Incidence densities

The incidence density rates represent the rate of new cases of a condition over a given period, standardized by the total time at risk [63]. Since many HAIs cannot be linked to a specific risk factor, the total at-risk time for calculating infection incidence densities is often based on cumulative patient days. NeoIPC Surveillance calculates infection density rates of HAIs by dividing the total number of infection cases by the total patient-days and then multiplying by 1000. This provides the infection rate per 1000 patient-days, allowing for standardized monitoring of HAI incidence across different patient populations and time frames.

$$ \begin{gathered} HAI\:incidence\:density\:rate \hfill \\ \quad \quad = \frac{{Number\:of\:infections}}{{Total\:patient\:days}} \times \:1000 \hfill \\ \end{gathered} $$

For device-associated infections, incidence densities measure the occurrence of BSI and pneumonia that are associated with medical devices, specifically catheters (CVC and PVC) or ventilators (INV or NIV), calculated per device-days. This rate is then multiplied by 1000, indicating the number of device-associated infections per 1000 device-days.

$$ \begin{gathered} Device - associated\:incidence\:density\:rate \hfill \\ \quad \quad = \frac{{Number\:of\:device - associated\:infections}}{{Total\:device\:days}} \times \:1000 \hfill \\ \end{gathered} $$

#### Surgical site infection (SSI) rates

SSI rates are the percentage of SSIs that occur during the observation period after an operative procedure. It is calculated by dividing the number of SSIs occurring after a surgical procedure by the number of surgical procedures and multiplying the result by 100. Since the risk of developing an SSI depends on the type of surgical procedure, multiple SSI rates are calculated for groups of similar procedures. Nevertheless, an overall SSI rate will be calculated to account for the fact that surgery is less common in VLBW/VPT infants than in adults, and grouping procedures may result in very low procedure counts per group.

$$ \begin{gathered} \:Overall\:SSI\:rate \hfill \\ \quad \quad = \frac{{Total\:SSI\:cases}}{{Total\:number\:of\:surgeries}} \times \:100 \hfill \\ \end{gathered} $$

$$ \begin{gathered} Specific\:SSI\:rate \hfill \\ \quad \quad = \frac{{Total\:SSI\:cases\:observed\:after\:a\:specific\:surgery\:}}{{Total\:number\:of\:a\:specific\:surgery}} \times \:100 \hfill \\ \end{gathered} $$

#### Standardized infection ratio (SIR)

While HAI rates reveal risk factors and infection patterns across four broad birth weight categories, a 500 g stratification alone may not accurately represent neonatal infection risk. This stratification does not account for patient transfers between units or facilities, such as to surgical units or hospitals closer to the family’s residence, which may influence the attribution and distribution of infection risk. Consequently, some units may accumulate more high-risk days during the early, unstable phase of an infant’s hospital stay, whereas others primarily care for already stabilized infants and therefore accumulate more low-risk days. The standardized infection ratio (SIR) compares observed infections to expected numbers adjusted for specific patient-related risk factors, enabling benchmarking across units or facilities. In NeoIPC Surveillance, expected infections are calculated from reference data and adjusted for two key variables, birth weight and day of life, allowing for a more accurate assessment of infection risk than raw rates. A score of 1.0 indicates that the observed infection rates match the expected rates, whereas values above or below 1.0 indicate higher or lower rates than expected, respectively. The Centers for Disease Control and Prevention (CDC) and similar organizations globally use SIRs for monitoring and improving HAI prevention programs [64–66].

$$\:Standardized\:infection\:ratio=\frac{Total\:HAIs\:observed}{Total\:HAIs\:expected}$$

### Data submission and storage

**Fig. 2:**
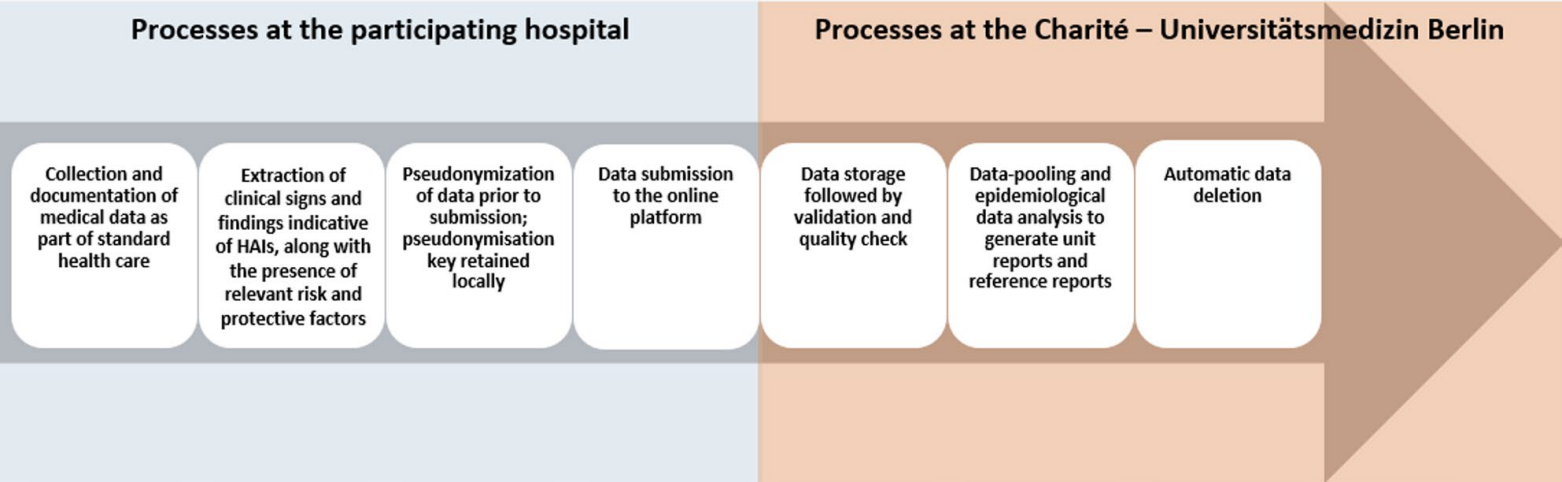
Dataflow within the NeoIPC Surveillance

Participants utilize a free, web-based open source software platform (DHIS2) for data submission, which facilitates both local data management and data exchange with the NeoIPC network [67, 68]. The data entered into the online data collection platform are used to calculate epidemiological metrics, such as standardized and stratified infection rates, both individually for each participating unit (institution-specific analysis) anytime and pooled as reference values for benchmarking, i.e., reference data, annually (Fig. 2). These metrics are provided in the form of reports that contain aggregated data only, ensuring that personal identification is impossible. Regular and complete data submission is essential for generating timely and accurate reports.

To ensure data integrity, all collected data should be stored securely, whether on paper or digitally. Physical records, such as pseudonymization keys and paper datasheets, should be kept in secure locations with controlled access by the sites. The digital data are protected with encryption and stored on secure servers of Charité – Universitätsmedizin Berlin. Regular backups will be performed to prevent data loss, and the stored data will be deleted every 10 years by default.

### Data privacy and security

Healthcare data are highly sensitive and must be protected to maintain patient confidentiality. While the surveillance databases do not contain information that is typically used to identify a person (e.g., names, ID numbers), especially in the context of HAIs in high-risk infants, they may still contain some information (e.g., extremely low birth weight or the day of life) that could be used to identify a patient. None of this potentially identifying information will be published, but it is necessary to collect for the purposes of risk standardization and stratification. Therefore, collected data are subject to data privacy rules and require specific documentation and the maintenance of technical and organizational measures to ensure data security and guarantee the anonymity of patients in public reports and datasets.

To use the NeoIPC Surveillance data submission platform, participating institutions sign a cooperation agreement with Charité – Universitätsmedizin Berlin, detailing the collaboration and ensuring compliance with the European General Data Protection Regulation (GDPR), which is applicable in Germany, where the platform is hosted. While collecting data for NeoIPC Surveillance, information such as patient names, birthdates, and hospital IDs are kept confidential and are never included in external submissions or reports. Data abstractors pseudonymize patient data by removing personal identifiers and assigning a unique NeoIPC identification number, allowing them to trace patients within the system without revealing their identities. NeoIPC Surveillance does not disclose the identities of individual hospitals in its publications and ensures that aggregated data cannot be traced back to any specific institution.

## Discussion

NeoIPC Surveillance provides a comprehensive framework for performing infection surveillance focused primarily on HAIs in VLBW and VPT infants, including data on MDRO infection prevalence and neonatal antibiotic use. On the basis of an analysis of multiple data collection systems, including the National Healthcare Safety Network (NHSN), the European Centre for Disease Prevention and Control (ECDC), and the German National Surveillance System for VLBW (NEO-KISS), an international team of neonatologists, epidemiologists and IPC specialists established methods and surveillance definitions. The toolkit is entirely free to use, and live training courses covering both data collection procedures and use of the web-based data management platform are held regularly in English. Access to the web-based data management platform is provided upon completion of contractual requirements.

NeoIPC Surveillance offers practical support for healthcare providers in neonatology, particularly by enabling robust IPC strategies. Through consistent application, facilities can generate reliable data to assess the burden of HAIs, antibiotic use and MDRO infection statistics, as well as assess key risk and protective factors that are associated with the acquisition of MDROs and the development of HAIs. These data can help neonatal units evaluate the effectiveness of their IPC interventions, enabling them to adjust strategies and improve patient outcomes [69]. The implementation of IPC-focused surveillance in neonatology includes additional benefits, such as data elements, definitions, and data analysis methods such as SIRs, which are customized for this particular patient population.

In settings seeking to establish regional or national surveillance networks for neonatal IPC, NeoIPC provides a foundational tool for consistent data collection and reporting. The system also has strong potential to support an international surveillance network, allowing benchmarking across similar settings on a supranational level. This capability enables neonatology units to compare infection rates, identify common challenges, and foster collaborative improvements in IPC practices globally.

Establishing a neonatal IPC surveillance network on a supranational scale holds great promise but also presents multiple implementation challenges. Key concerns include data privacy, security, and ethical standards, which require a clear, standardized regulatory framework to ensure safe and responsible data use across diverse healthcare and legal settings. Addressing these concerns requires not only adherence to the European General Data Protection Regulation (GDPR) but also a broader ethical approach that can be applied across diverse legal frameworks beyond the European Union.

According to the GDPR, personal data should be collected for only specific legitimate purposes and processed accordingly, with or without consent in certain cases to protect individuals or public health [70, 71]. In hospitals, patients or their guardians typically consent to health data collection, expecting it to be used under professional secrecy for care and management. Surveillance of nosocomial infections, as part of routine patient care, aligns with these purposes and serves public health by monitoring HAIs and threats such as MDROs. Participation in NeoIPC requires a bilateral joint controller agreement to ensure that the GDPR-compliant data are processed.

Another potential challenge that might hinder the adoption of NeoIPC is the additional workload caused by duplicate data entry, especially in settings already engaged with national or regional surveillance programs that use similar datasets. To address this, increasing transparency in data collection methods and surveillance protocols is essential, thereby improving methodological comparability and enabling automated or semiautomated data exchange. This would significantly reduce redundant work and facilitate integration with existing systems. A further considerable obstacle in this context arises from the language barrier, which can impede the effective implementation of a supranational network. The lack of tools and training materials in users’ native languages may limit acceptance and participation among non-English speakers. Offering multilanguage resources can increase accessibility, promote consistent data quality, and foster collaboration across regions.

Establishing neonatal HAI surveillance systems in LMICs remains challenging, particularly in settings with limited staff, diagnostic capacity, and digital infrastructure. The World Health Organization has reported that the African Region has the lowest uptake of HAI surveillance globally, underscoring the need for tailored and feasible approaches [72]. In this context, barriers may range from major structural constraints, such as workforce shortages and absent digital infrastructure, to more practical issues, including limited familiarity with surveillance methodologies. To enhance feasibility across diverse settings, the NeoIPC Surveillance toolkit is freely accessible and can be implemented using paper-based data collection with manual analysis where digital systems are not available. Infection definitions were intentionally developed to be applicable across heterogeneous healthcare environments, and ongoing feedback from LMIC partners will be used to further refine and adapt infection definitions where needed. While paper-based implementation remains feasible in settings without digital infrastructure, system-level developments such as semi-automated data collection interfaces may reduce workload and improve sustainability across resource-constrained environments. Initial pilot implementation in an LMIC setting demonstrated good overall feasibility. During this process, interest was expressed in extending surveillance to larger infants, as VLBW infants may not represent the sole priority population in some settings. Although such an expansion was technically feasible within the existing framework, it was associated with increased workload. Further evaluation is therefore needed to assess the feasibility, resource implications, and added value of expanding the target population, which may result in the development of an optional supplementary module for sites wishing to adopt a broader surveillance scope. To support this process, a structured evaluation survey has been launched inviting LMIC sites to test the surveillance system and provide systematic feedback on implementation experiences, context-specific barriers, and resource requirements.

Beyond implementation-focused adaptations, potential future developments for NeoIPC Surveillance include structural and technical enhancements applicable across diverse healthcare settings. These include the provision of multilingual user interfaces and training materials to improve accessibility, adoption, and data quality, as well as the creation of supplementary surveillance modules, such as an expanded core module with more inclusive eligibility criteria encompassing larger infants. Over time, the surveillance scope may be extended to include additional infections, including early-onset sepsis (EOS), guided by feedback from consortium partners and participating sites. Planned improvements also include streamlining surgical site infection data submission through the integration of standardized lists of commonly performed neonatal procedures. In parallel, unit-level reporting tools may be enhanced to enable real-time, unit-specific data analysis with intuitive visualizations. Automated generation of standardized reports and presentation-ready outputs, such as figures and slides, may support local quality improvement activities, facilitate communication within clinical teams, and reduce the analytical burden on participating units. Finally, the development of semi-automated data collection and data exchange interfaces between the NeoIPC platform and hospital information systems is anticipated to reduce duplicate data entry, support integration with existing surveillance systems, and improve efficiency across both high- and low-resource settings. Building on these interfaces, artificial intelligence-supported solutions may, in the longer term, further enhance interoperability, benchmarking, and decision-making.

## Conclusion

The lack of consensus on risk adjustment and surveillance definitions in current neonatal HAI surveillance systems, together with the widespread use of insufficiently specific datasets and the limited availability of affordable, accessible tools, represents a significant barrier to international benchmarking and quality improvement in neonatal care. NeoIPC Surveillance aims to improve neonatal outcomes by establishing a standardized system for monitoring HAIs, identifying best practices, strengthening IPC strategies, and enabling harmonization and benchmarking between various settings.

The publication of data collection methods and surveillance protocols, including detailed definitions of data elements, is essential to improve transparency and data exchange. As more healthcare systems around the world begin sharing data to support HAI surveillance and quality improvement, the importance of convenient and consistent data privacy regulations continues to grow. Sharing this kind of information is becoming essential for better patient care and for keeping track of public health trends across countries.

## Supplementary Information

Below is the link to the electronic supplementary material.

Additional File 1. NeoIPC surveillance protocol

Additional File 2. LMIC evaluation survey

Additional File 3. Data collection sheets

Additional File 4. Surveillance infection definitons

Additional File 5. Infectious agents list

## Data Availability

This article describes the methodological framework and surveillance protocol of NeoIPC Surveillance. No datasets were analyzed or reported in this manuscript. The aggregated surveillance data and surveillance materials are publicly accessible on the project website at https://neoipc.org/surveillance.

## Abbreviations

**Abbreviation**: **Definition**
AMR: Antimicrobial resistance
ANZNN: Australian and New Zealand Neonatal Network
ASA: American Society of Anesthesiologists
BMC: BioMed Central
BSI: Bloodstream infection
CDC: Centers for Disease Control and Prevention
CLABSI: Central line–associated bloodstream infection
CNN: Canadian Neonatal Network
DHIS2: District Health Information Software 2
ECDC: European Centre for Disease Prevention and Control
ELBW: Extremely low birth weight
EPT: Extremely preterm
EU: European Union
GDPR: General Data Protection Regulation
HAI: Healthcare-associated infection
ICCQIP: Infection in Critical Care Quality Improvement Programme
INEO / iNeo: International Network for Evaluation of Outcomes
INICC: International Nosocomial Infection Control Consortium
IPC: Infection prevention and control
JANIS: Japanese Nosocomial Infection Surveillance
LMIC: Low- and middle-income country
LOS: Late-onset sepsis
MDRO: Multidrug-resistant organism
NeoIPC: Neonatal Infection Prevention and Control Project
NICHD: Eunice Kennedy Shriver National Institute of Child Health and Human Development
NICU: Neonatal intensive care unit
NNAP: National Neonatal Audit Programme
NRN: Neonatal Research Network
NRNJ: Neonatal Research Network of Japan
NHSN: National Healthcare Safety Network
PNSN: Polish Neonatology Surveillance Network
PPS: Point prevalence survey
SIR: Standardized infection ratio
SSI: Surgical site infection
SNN: Swiss Neonatal Network
VLBW: Very low birth weight
VON: Vermont Oxford Network
VPT: Very preterm
WHO: World Health Organization

## References

[1] Stoll BJ, Hansen N, Fanaroff AA, Wright LL, Carlo WA, Ehrenkranz RA, et al. Late-onset sepsis in very low birth weight neonates: the experience of the Nichd neonatal research network. Pediatrics. 2002;110(2):285–91. 12165580 10.1542/peds.110.2.285

[2] Marty D, Sorum K, Smith K, Nicoski P, Sayyed BA, Amin S. Nosocomial infections in the neonatal intensive care unit. Neoreviews. 2024;25(5):e254–64. 38688885 10.1542/neo.25-5-e254

[3] Brito DVDD, Von Dolinger EJO, Abdallah VOS, Darini ALC, Gontijo Filho PP. Two outbreaks of mixed etiology associated with central venous catheters inserted by phlebotomy in critical neonates. Braz J Infect Dis. 2009;13(3):177–82. 20191193 10.1590/s1413-86702009000300005

[4] Cheung GY, Otto M. Understanding the significance of Staphylococcus epidermidis bacteremia in babies and children. Curr Opin Infect Dis. 2010;23(3):208–16. 20179594 10.1097/QCO.0b013e328337fecb PMC2874874

[5] Lachassinne E, Letamendia-Richard E, Gaudelus J. Épidémiologie des infections nosocomiales En néonatalogie. Arch Pediatr. 2004;11(3):229–33. 15049286 10.1016/j.arcped.2003.10.016 PMC7133330

[6] Cassini A, Plachouras D, Eckmanns T, Abu Sin M, Blank H-P, Ducomble T, et al. Burden of six healthcare-associated infections on European population health: estimating incidence-based disability-adjusted life years through a population prevalence-based modelling study. PLoS Med. 2016;13(10):e1002150. 27755545 10.1371/journal.pmed.1002150 PMC5068791

[7] Pittet D. Infection control and quality health care in the new millennium. Am J Infect Control. 2005;33(5):258–67. 15947742 10.1016/j.ajic.2004.11.004

[8] Schwab F, Gastmeier P, Piening B, Geffers C. The step from a voluntary to a mandatory National nosocomial infection surveillance system: the influence on infection rates and surveillance effect. Antimicrob Resist Infect Control. 2012;1(1):24. 22958509 10.1186/2047-2994-1-24 PMC3489557

[9] Cailes B, Kortsalioudaki C, Buttery J, Pattnayak S, Greenough A, Matthes J, et al. Antimicrobial resistance in Uk neonatal units: neonin infection surveillance network. Archives Disease Child - Fetal Neonatal Ed. 2018;103(5):F474–8. 10.1136/archdischild-2017-313238 29074716

[10] Heijting IE, Antonius TAJ, Tostmann A, De Boode WP, Hogeveen M, Hopman J. Sustainable neonatal clabsi surveillance: consensus towards new criteria in the Netherlands. Antimicrob Resist Infect Control. 2021;10(1). 10.1186/s13756-021-00900-3 PMC7866773 33546759

[11] Cailes B, Vergnano S, Kortsalioudaki C, Heath P, Sharland M. The current and future roles of neonatal infection surveillance programmes in combating antimicrobial resistance. Early Hum Dev. 2015;91(11):613–8. 26386612 10.1016/j.earlhumdev.2015.08.012

[12] Dramowski A, Aucamp M, Beales E, Bekker A, Cotton MF, Fitzgerald FC et al. Healthcare-associated infection prevention interventions for neonates in resource-limited settings. Front Pead. 2022;10. 10.3389/fped.2022.919403 PMC9301049 35874586

[13] Dramowski A, Cotton MF, Whitelaw A. Surveillance of healthcare-associated infection in hospitalised South African children: which method performs best? S. Afr Med J. 2016;107(1):56–63. 10.7196/SAMJ.2016.v107.i1.11431 28112093

[14] The neoipc project. Available from: https://neoipc.org/

[15] The neoipc surveillance. system Available from: https://neoipc.org/surveillance/

[16] National healthcare safety network [CDC’s National Healthcare Safety. Network is the nation’s most widely used healthcare-associated infection tracking system.]. Available from: https://www.cdc.gov/nhsn/index.html

[17] Gastmeier P, Geffers C, Schwab F, Fitzner J, Obladen M, Rüden H. Development of a surveillance system for nosocomial infections: the component for neonatal intensive care units in Germany. J Hosp Infect. 2004;57(2):126–31. 15183242 10.1016/j.jhin.2003.12.038

[18] Neo-kiss [cited 2024. Nationales Referenzzentrum für Surveillance von nosokomialen Infektionen]. Available from: https://www.nrz-hygiene.de/KISS-Modul/KISS/NEO

[19] Madrid-Aguilar M, López-Herrera MC, Pérez-López J, Escudero-Argaluza J, Santesteban-Otazu E, Piening B, et al. [implementation of Neokisses in spain: A validated surveillance system for nosocomial sepsis in very low birth weight infants]. Pediatr (Engl Ed). 2019;91(1):3–12. 10.1016/j.anpedi.2018.06.020 30262156

[20] Neokisses surveillance system [cited 2024. Available from: https://www.neokisses.com/en/2801-2/

[21] Eunice kennedy shriver national institute of child health and human development. Neonatal research network (nrn) Available from: https://www.nichd.nih.gov/research/supported/nrn

[22] Neonatal research network [Funded by the Eunice Kennedy Shriver National Institute of Child Health and, Development H. (NICHD)]. Available from: https://neonatal.rti.org/

[23] The canadian neonatal network (cnn) Available from: http://www.canadianneonatalnetwork.org/portal/

[24] Babazono A, Kitajima H, Nishimaki S, Nakamura T, Shiga S, Hayakawa M, et al. Risk factors for nosocomial infection in the neonatal intensive care unit by the Japanese nosocomial infection surveillance (janis). Acta Med Okayama. 2008;62(4):261–8. 18766209 10.18926/AMO/30938

[25] Neonatal research network. Database Japan Available from: https://plaza.umin.ac.jp/nrndata/indexe.htm

[26] The infection in. critical care quality improvement programme (iccqip) Available from: https://ficm.ac.uk/iccqip

[27] Adams M, Hoehre TC, Bucher HU. The Swiss neonatal quality cycle, a monitor for clinical performance and tool for quality improvement. BMC Pediatr. 2013;13:152. 24074151 10.1186/1471-2431-13-152 PMC3856669

[28] The swiss neonatal network & follow-up group. (swissneonet) Available from: https://www.neonet.ch/swissneonet/aims-and-description

[29] Makhoul IR, Sujov P, Smolkin T, Lusky A, Reichman B. Epidemiological, clinical, and Microbiological characteristics of late-onset sepsis among very low birth weight infants in israel: A National survey. Pediatrics. 2002;109(1):34–9. 11773539 10.1542/peds.109.1.34

[30] Reichman B, Klinger G, Zangen S, Levitzki O, Lerner-Geva L. The Israel neonatal network and National very low birth weight infant database. Pediatr Med. 2021;6.

[31] Romaniszyn D, Różańska A, Wójkowska-Mach J, Chmielarczyk A, Pobiega M, Adamski P et al. Epidemiology, antibiotic consumption and molecular characterisation of Staphylococcus aureus infections – data from the Polish neonatology surveillance network, 2009–2012. BMC Infect Dis. 2015;15(1). 10.1186/s12879-015-0890-3 PMC4389670 25888217

[32] Wójkowska-Mach J, Gulczyńska E, Nowiczewski M, Borszewska-Kornacka M, Domańska J, Merritt TA, et al. Late-onset bloodstream infections of very-low-birth-weight infants: data from the Polish neonatology surveillance network in 2009–2011. BMC Infect Dis. 2014;14(1):339. 24939563 10.1186/1471-2334-14-339 PMC4074408

[33] Wójkowska-Mach J, Różańska A, Borszewska-Kornacka M, Domańska J, Gadzinowski J, Gulczyńska E, et al. Necrotising Enterocolitis in preterm infants: epidemiology and antibiotic consumption in the Polish neonatology network neonatal intensive care units in 2009. PLoS ONE. 2014;9(3):e92865. 24658445 10.1371/journal.pone.0092865 PMC3962467

[34] National neonatal audit. programme (nnap) Available from: https://www.rcpch.ac.uk/work-we-do/clinical-audits/nnap

[35] International nosocomial infection. control consortium Available from: https://www.inicc.org/site/

[36] Vermont oxford network. Available from: https://public.vtoxford.org/

[37] Valls-i-Soler A, Carnielli V, Claris O, de la Cruz Bértolo J, Halliday HL, Hallman M, et al. Euroneostat: A European information system on the outcomes of care for very-low-birth-weight infants. Neonatology. 2008;93(1):7–9. 17630492 10.1159/000105519

[38] Shah PS, Lee SK, Lui K, Sjors G, Mori R, Reichman B, et al. The international network for evaluating outcomes of very low birth weight, very preterm neonates (ineo): A protocol for collaborative comparisons of international health services for quality improvement in neonatal care. BMC Pediatr. 2014;14:110. 24758585 10.1186/1471-2431-14-110 PMC4021416

[39] Shah PS, Lui K, Reichman B, Norman M, Kusuda S, Lehtonen L, et al. The international network for evaluating outcomes (ineo) of neonates: evolution, progress and opportunities. Transl Pediatr. 2019;8(3):170–81. 31413951 10.21037/tp.2019.07.06 PMC6675683

[40] The international network for evaluation of. outcomes (ineo) Available from: http://www.ineonetwork.org/

[41] The australian and. new zealand neonatal network Available from: https://www.anznn.net/

[42] Neonin. neonatal infection surveillance network Available from: https://neonin.org.uk/about

[43] Vergnano S, Menson E, Kennea N, Embleton N, Russell AB, Watts T, et al. Neonatal infections in england: the Neonin surveillance network. Archives Disease Child - Fetal Neonatal Ed. 2011;96(1):F9–14. 10.1136/adc.2009.178798 20876594

[44] Zingg W, Hopkins S, Gayet-Ageron A, Holmes A, Sharland M, Suetens C, et al. Health-care-associated infections in neonates, children, and adolescents: an analysis of paediatric data from the European centre for disease prevention and control point-prevalence survey. Lancet Infect Dis. 2017;17(4):381–9. 28089444 10.1016/S1473-3099(16)30517-5

[45] Neoipc clinical practice. network Available from: https://neoipc.org/clinical-practice-network/

[46] Damschroder LJ, Aron DC, Keith RE, Kirsh SR, Alexander JA, Lowery JC. Fostering implementation of health services research findings into practice: A consolidated framework for advancing implementation science. Implement Sci. 2009;4(1):50. 19664226 10.1186/1748-5908-4-50 PMC2736161

[47] Team CR. Consolidated framework for implementation research 2022 [21.01.2026]. Available from: https://cfirguide.org/

[48] Statement on asa physical. status classification system Available from: https://www.asahq.org/standards-and-practice-parameters/statement-on-asa-physical-status-classification-system

[49] Herman TF, Popowicz P, Bordoni B. Wound classification. Statpearls. Treasure Island (FL): StatPearls publishing copyright © 2024. StatPearls Publishing LLC.; 2024. 32119343

[50] Polin RA, Denson S, Brady MT, Papile L-A, Baley JE, Carlo WA, et al. Strategies for prevention of health care–associated infections in the Nicu. Pediatrics. 2012;129(4):e1085–93. 22451712 10.1542/peds.2012-0145

[51] Cernada M, De Alba Romero C, Fernández-Colomer B, González-Pacheco N, González M, Couce ML. Health care-associated infections in neonatology. Anales De Pediatría (English Edition). 2024;100(1):46–56. 38177038 10.1016/j.anpede.2023.12.004

[52] Rosenthal VD, Yin R, Nercelles P, Rivera-Molina SE, Jyoti S, Dongol R, et al. International nosocomial infection control consortium (inicc) report of health care associated infections, data summary of 45 countries for 2015 to 2020, adult and pediatric units, device-associated module. Am J Infect Control. 2024;52(9):1002–11. 38185380 10.1016/j.ajic.2023.12.019

[53] Rallis D, Giapros V, Serbis A, Kosmeri C, Baltogianni M. Fighting antimicrobial resistance in neonatal intensive care units: rational use of antibiotics in neonatal sepsis. Antibiotics. 2023;12(3):508. 36978375 10.3390/antibiotics12030508 PMC10044400

[54] Bhat BV. Fine-tuning the duration of antibiotic therapy for neonatal sepsis. Indian J Pediatr. 2022;89(4):323–4. 35041185 10.1007/s12098-021-04063-2

[55] Manzoni P, De Luca D, Stronati M, Jacqz-Aigrain E, Ruffinazzi G, Luparia M, et al. Prevention of nosocomial infections in neonatal intensive care units. Am J Perinatol. 2013;30(2):81–8. 23292914 10.1055/s-0032-1333131

[56] Polin RA, Denson S, Brady MT, Papile L-A, Baley JE, Carlo WA, et al. Epidemiology and diagnosis of health care–associated infections in the Nicu. Pediatrics. 2012;129(4):e1104–9. 22451708 10.1542/peds.2012-0147

[57] Ramasethu J. Prevention and treatment of neonatal nosocomial infections. Maternal Health Neonatol Perinatol. 2017;3(1). 10.1186/s40748-017-0043-3 PMC5307735 28228969

[58] Goldman AS. The immune system in human milk and the developing infant. Breastfeed Med. 2007;2(4):195–204. 18081456 10.1089/bfm.2007.0024

[59] Nolan LS, Rimer JM, Good M. The role of human milk oligosaccharides and probiotics on the neonatal Microbiome and risk of necrotizing enterocolitis: a narrative review. Nutrients. 2020;12(10):3052. 33036184 10.3390/nu12103052 PMC7600747

[60] Casper C, Sarapuk I, Pavlyshyn H. Regular and prolonged skin-to-skin contact improves short-term outcomes for very preterm infants: a dose-dependent intervention. Arch Pediatr. 2018;25(8):469–75. 30340943 10.1016/j.arcped.2018.09.008

[61] Lamy Filho F, de Sousa SH, Freitas IJ, Lamy ZC, Simões VM, da Silva AA, et al. Effect of maternal skin-to-skin contact on decolonization of methicillin-oxacillin-resistant Staphylococcus in neonatal intensive care units: a randomized controlled trial. BMC Pregnancy Childbirth. 2015;15:63. 25880822 10.1186/s12884-015-0496-1 PMC4374510

[62] Sivanandan S, Sankar MJ. Kangaroo mother care for preterm or low birth weight infants: a systematic review and meta-analysis. BMJ Global Health. 2023;8(6):e010728. 37277198 10.1136/bmjgh-2022-010728 PMC10254798

[63] Rothman KJG, Lash S. Timothy L. Modern epidemiology. 3rd ed. Philadelphia, PA: Lippincott Williams & Wilkins; 2008.

[64] Leistner R, Piening B, Gastmeier P, Geffers C, Schwab F. Nosocomial infections in very low birthweight infants in germany: current data from the National surveillance system neo-kiss. Klin Padiatr. 2013;225(2):75–80. 23526612 10.1055/s-0033-1334886

[65] Tanamas SK, Lim L-L, Bull AL, Malloy MJ, Cheng AC, Worth LJ. Applying the standardized infection ratio for reporting surgical site infections in Australian healthcare facilities. Antimicrob Stewardship Healthc Epidemiol. 2023;3(1). 10.1017/ash.2023.478 PMC10753515 38156237

[66] Promotion NCfEaZIDUSDoHQ. The nhsn standardized infection ratio (sir): A guide to the sir. In: Network NHS, editor. United States.

[67] Neoipc surveillance online. platform Available from: https://neoipc.charite.de/

[68] Oslo Uo. Dhis2: District health information software 2 [cited 2025 July 14]. Available from: https://dhis2.org/

[69] Gastmeier P, Schwab F, Sohr D, Behnke M, Geffers C. Reproducibility of the surveillance effect to decrease nosocomial infection rates. Infect Control Hosp Epidemiol. 2009;30(10):993–9. 19719414 10.1086/605720

[70] Article 5(1)(b) general data protection regulation (gdpr). (2016).

[71] Article 9(2)(a)(i) general data protection regulation (gdpr). (2016).

[72] World Health O. Global report on infection prevention and control 2024: executive summary. Geneva: World Health Organization; 2024. 2024-12-17.

