# Supplementary material for: Methodological framework for the surveillance of healthcare-associated infections in high-risk infants: the NeoIPC surveillance core module protocol
Source: Antimicrob Resist Infect Control. 2026 Feb 19;15:30. doi: 10.1186/s13756-026-01711-0 (PMC12930787; doi:10.1186/s13756-026-01711-0)
Supplement: Supplementary file 1 — Additional File 1. NeoIPC surveillance protocol [file 13756_2026_1711_MOESM1_ESM.pdf]

PROTOCOL

INFECTION SURVEILLANCE FOR NEONATOLOGY

CORE MODULE

VERY LOW BIRTH WEIGHT (VLBW)

VERY PRETERM (VPT)

INFANTS

NeoIPC Project
https://neoipc.org/

This project has received funding from the European Union’s Horizon 2020 research and innovation programme under grant agreement No. 965328.

2023-08-01 Version 1.2

TABLE OF CONTENTS

1 Introduction ... 4
1.1 What are you reading here? ... 4
1.2 What is the NeoIPC project? ... 4
1.3 Background ... 4
1.4 Advice for use and requirements for participation ... 5
2 Data Management ... 6
2.1 Data privacy and security ... 6
2.1.1 Patient information ... 6
2.1.2 Hospital information ... 6
2.2 Data submission ... 7
3 Data Collection ... 7
3.1 Case eligibility criteria for the core module ... 9
3.2 Patient data collection ... 10
3.2.1 Pseudonymisation table ... 10
3.2.2 Master data collection sheet ... 12
3.2.3 Patient progress chart ... 13
3.3 Surgical procedure data collection ... 14
3.4 Infection data collection ... 15
3.4.1 Primary sepsis/BSI ... 16
3.4.2 Necrotizing enterocolitis ... 17
3.4.3 Pneumonia ... 18
3.4.4 Surgical site infections ... 19
3.4.5 Device-associated infection ... 20
3.4.6 Secondary bloodstream infection ... 21
4 Infection Definitions ... 22
4.1 Primary sepsis/bloodstream infection ... 22
4.1.1 Clinical sepsis ... 23
4.1.2 LCBSI caused by a recognised pathogen ... 23
4.1.3 LCBSI caused by common commensals ... 24
4.2 Necrotizing enterocolitis (NEC) ... 27
4.3 Pneumonia ... 28
4.4 Surgical site infection (SSI) ... 30
4.4.1 Superficial incisional SSI ... 31
4.4.2 Deep incisional SSI ... 33
4.4.3 Organ/space SSI ... 35

5 Data Dictionary ... 37
5.1 Patient master data ... 37
5.1.1 Enrolment ... 37
5.1.2 Admission information ... 38
5.1.3 Surveillance end ... 38
5.2 Surgical procedure ... 39
5.3 Infection data ... 41
5.3.1 General infection data ... 41
5.3.2 BSI specific data ... 42
5.3.3 NEC specific data ... 43
5.3.4 Pneumonia specific data ... 43
5.3.5 SSI specific data ... 44
6 Data Analysis ... 46
6.1 Risk factors and protective factors ... 46
6.1.1 Device utilization ... 46
6.1.2 Antibiotic use ... 46
6.1.3 Protective factor implementation ... 47
6.2 Infections ... 47
6.2.1 Incidence densities ... 47
6.2.2 Device-associated infections ... 48
6.2.3 Surgical site infections ... 48
6.2.4 Standardized infection rate ... 49
7 Abbreviations ... 50
8 Imprint ... 51

1 INTRODUCTION

1.1 WHAT ARE YOU READING HERE?

This document is the surveillance protocol for the core module of the NeoIPC surveillance which covers surveillance of nosocomial infections in Very Low Birth Weight (VLBW)/ Very Preterm (VPT) Infants. It is part of a toolkit for the surveillance of healthcare-associated infections (HAI) in neonatology that we developed within the NeoIPC project. If you want to perform surveillance of early-onset infections or of healthcare-associated infections in infants with a birth weight above 1500 g and a gestational age greater than 32 weeks, the toolkit provides additional methods that are covered in separate protocols.

This protocol provides methodological reference and support for HAI surveillance in neonatal units or for those planning to develop an HAI surveillance programme. By adhering to the methods and definitions we specify here, you can ensure that the surveillance data you generate is comparable to the reference data in the NeoIPC project, even if you are not actively participating in the project.

1.2 WHAT IS THE NEOIPC PROJECT?

Within the NeoIPC project, we want to support you with infection prevention and control (IPC) interventions and strategies in your neonatal intensive care unit, especially if you observe a high prevalence of hospital-acquired infections or multidrug-resistant (MDR) bacteria. We are an international team of clinicians and scientists from 13 collaborating partner institutions with a proven record in the areas of neonatal intensive care, neonatal infection, infection prevention and control (IPC), implementation science, microbiology, and surveillance, who collaborate in this project to achieve two overarching goals:

1. Develop and implement an innovative approach towards the evaluation of IPC interventions in neonatology via:
    a. Robust assessment of the effectiveness of interventions in a randomised controlled trial (RCT)
    b. Identification of a suitable implementation strategy.
2. Generate widely relevant and globally transferable outputs to improve IPC in neonatal care through:
    a. Formation of a clinical practice network to support the implementation of IPC in neonatal care, including a variety of practice settings like neonatal intensive care unit (NICU), high care, special care, and kangaroo care (KC).
    b. Development of an open structure for targeted surveillance of IPC processes and outcomes across a large variety of settings.

This project has received funding from the European Union's Horizon 2020 research and innovation programme under grant agreement No. 965328.

1.3 BACKGROUND

Worldwide, neonates and especially preterm infants are among the patient groups with the highest incidence of nosocomial infections. Some of these infections can be prevented by infection-prevention measures, which must be implemented in each individual neonatal unit and adapted to

the specific infection risks of the patients. In many hospitals in Europe and worldwide, healthcare professionals do not have good data to assess the burden of healthcare-associated infections and the risk factors contributing to their development or to evaluate the effectiveness of infection prevention interventions. Support for surveillance of healthcare-associated infections in neonates or reference data for benchmarking is not available in most countries. Where national and supranational data collection systems exist, they frequently have a broad approach that is limited in its ability to assess the situation with respect to infection prevention and control. In the short term, we aim to improve this situation by providing those interested in IPC surveillance in neonatology with tools and methods to get started, and in the longer term by potentially contributing to the formation of regional surveillance infrastructures.

1.4 Advice for use and requirements for participation

If you want to start collecting data using the NeoIPC surveillance methods and tools, you can just do so without asking us for permission or paying any fees. All manuals and tools are free to use, and you can start using them right away if you think they will assist in quality improvement for infection control in your department.

By starting with paper forms and performing calculations with pen and paper or by using a spreadsheet, you have a very low barrier of entry, and it may even help you to gain a thorough understanding of the calculations that are performed internally by the more advanced tools. If you want to keep things running effectively over an extended period and you have the necessary resources, you may want to benefit from those tools by using the advanced surveillance software locally or by participating in NeoIPC or a regional surveillance network based on its methods.

For long-term success in surveillance of nosocomial infections, collecting data and producing reliable and comparable information in your department over a long period of time will be crucial. In order to facilitate the collection of such data, you may want to contribute data to the NeoIPC surveillance network. This data could be used to generate reports and benchmark your neonatal unit’s infection rates against others.

If you collect and submit data, and are responsible for quality improvement, it is essential that you have a clear understanding of the following:

- Patient eligibility criteria
- Data definitions for each data Item
- Procedures for filling and storing forms
- Data security and data privacy (protection of information that might be used to identify a patient outside of your department or hospital)
- Procedures for collecting, submitting, and correcting data
- Procedures for data management and data finalization
- Use of NeoIPC reports for monitoring and improving patient care

You should make sure that the whole data collection team in your centre accept the data definitions contained in this protocol and submit their data accordingly. To be able to generate up-to-date reference reports on a regular schedule, NeoIPC requires participants to regularly submit data via its web-based interface and to ensure that their software and hardware systems meet the minimum requirements.

The policies and procedures of your centre must ensure patient privacy and data security. It is important to protect patient identifier information based on applicable laws and centre policies. Do not submit any patient identifying information to the NeoIPC Network.

We will ensure to keep the disaggregated data submitted by each department strictly confidential and provide you with regular standardized and stratified reference reports that contain aggregated data so that no individual patient or department can be identified.

To help you navigate and better utilize the tools provided, we offer surveillance manuals, datasheets and training materials at https://neoipc.org/surveillance/. While our resources for personal interaction are limited, we will also try to support your team if you send your questions to neoipc-support@charite.de.

2 DATA MANAGEMENT

Routine surveillance of healthcare-associated infections is not research but is rather a way of performing quality assurance which is part of regular patient care. Because of this, the associated data collection is typically covered by the regular hospital treatment contracts or special legislation that mandates it and does not need explicit consent from the affected patients or their legal guardians. Nevertheless, you should make sure that this applies in your country before starting to collect any data and in addition adhere to some universal minimum standards that should apply irrespective of the legal framework.

2.1 DATA PRIVACY AND SECURITY

2.1.1 Patient information

Healthcare information is among the most private categories of information about humans in most cultures and should be handled with special care. When information that can lead to the identification of an individual patient is stored or transmitted, this should generally happen in a way that no unauthorized third party can access it. While this is usually very clear for typical human identifiers like the combination of family name, given name, and birth date, in the case of neonates even a very rare birth weight or gestational age may lead to the identification of an individual patient if combined with enough other information like birth date or the hospital the patient is treated in. Think about this special phenomenon whenever you publish data or information that results from your surveillance-related activities or transmit it to a system that is not approved by your hospital. This may even apply to an externally maintained NeoIPC surveillance software if there are no contracts that govern the data processing and ownership.

2.1.2 Hospital information

Information about hospitals generally is not protected by laws but some information may be considered as company secret and in that case, as a member of staff, you are typically not allowed to disclose it. Whether surveillance information (“hospital infection rates”) is a company secret or should be, is a matter of extensive debate. It is understandable that patient rights organizations advocate for transparency in that regard. Nevertheless, the publication of surveillance data can have a detrimental effect on both the perception of the treatment quality of a hospital and the ability of the surveillance team to perform surveillance in an honest and self-critical way.

Being a self-assessment tool, surveillance cannot rule out all forms of subjectivity and dishonesty and public pressure tends to shift data collection towards an overly critical assessment of infection records leading to low rates that can no longer be acted upon. In addition to the problem of pro-

forma-surveillance, there may be very legitimate or even honourable reasons for high infection rates in a hospital, like treating those who have an especially high risk for infections, that can't be adjusted for by the means of standardization or stratification of the collected data. These reasons are notoriously hard to communicate to the public which can lead to oversimplification and political abuse.

Since there is no easy solution for these problems NeoIPC will not disclose the identity of individual participating hospitals and make sure that publications and reports will not contain information that may lead to the identification of an individual hospital.

When publishing data yourself or as a regional network you should carefully consider the potential effects of including hospital-level information and do so in close coordination with your stakeholders.

2.2 Data submission

As stated above, you can use the tools and methods developed by the NeoIPC project to perform surveillance of nosocomial infections in your neonatology department without submitting any information to the NeoIPC project or a regional network.

If you decide to submit data, however, we strongly advise you to use the web-based reporting system we have developed in addition to the DHIS2 system.

You can use this platform both for internal data management and for the data exchange with NeoIPC or your regional network. Furthermore, you can create unit-based reports at any time and compare them to the NeoIPC reference reports that are published annually.

In order to analyse all data in the NeoIPC database, we will carry out a data extraction at a specified time every year. In order to facilitate benchmarking, you will need to have completed all data entry and addition of missing information from the previous year within 6 weeks of the end of a calendar year. This applies to patients whose surveillance data collection period ended in the previous year.

If DHIS2 platform is temporarily unavailable due to technical problems, please use the paper-based datasheets for documentation during this period and remember to enter this data into the web platform as soon as possible once it becomes available again.

3 Data Collection

The primary aim of the methods used by NeoIPC surveillance system is to support internal quality assurance standards, to make valid statements about the frequency of healthcare-associated infections in eligible infants during their inpatient stay and to promote implementation of IPC strategies in a neonatal unit.

Based on our experience, we anticipate that you will collect data both through the review of medical charts as well as through the interaction with the colleagues who are involved in treatment and care of the patient under surveillance. For this reason, your data collection should ideally occur at the patient's bedside, to maximize the amount of information available (by allowing attending clinicians to clarify unclear or incomplete chart entries), and to minimize the amount of time involved in tracking down medical records once the patient has left the hospital. You will typically identify patients with healthcare-associated infections through the regular examination of existing departmental patient records, including laboratory results. The success of your participation in NeoIPC surveillance will very much depend on the close communication between those who perform the surveillance (typically infection prevention and control professionals) and those who care for the

patient (typically neonatology professionals). Since the collected data will ultimately need to be entered into a computer, by combining data collection and data entry into a single procedure you have the potential to save time and reduce the risk of data copying errors.

There are two patient data collection sheets that must be fulfilled for all eligible patients for the NeoIPC surveillance programme:

- Master data collection sheet
- Patient progress chart

A third data collection sheet must also be fulfilled, if the patient undergoes a surgical procedure:

- Surgical procedure data collection sheet

In addition, if an eligible infant develops a healthcare-associated infection within the NeoIPC follow-up period, the corresponding infection data collection sheet/sheets should also be fulfilled:

- Blood stream infection
- Pneumonia
- Necrotizing enterocilitis
- Surgical site infection

Only these infections acquired in participating neonatology departments should be recorded.

All eligible infants should be observed until the end of the surveillance period, defined as the time when the infant **dies, is transferred, or is discharged** from the hospital.

Patients are additionally followed up for SSIs in case of a surgical procedure for 30 or 90 days, depending on whether an implant is present. For detailed information, see Surgical site infections.

3.1 CASE ELIGIBILITY CRITERIA FOR THE CORE MODULE

Any live-born infant;

a. with a birth weight of less than 1500 grams

AND/OR

b. whose gestational age is less than 32 weeks (31 weeks 6 days inclusive),

admitted to a ward in your neonatal unit **within 120 days of birth** is eligible for inclusion in the Core Module of the NeoIPC Surveillance Programme.

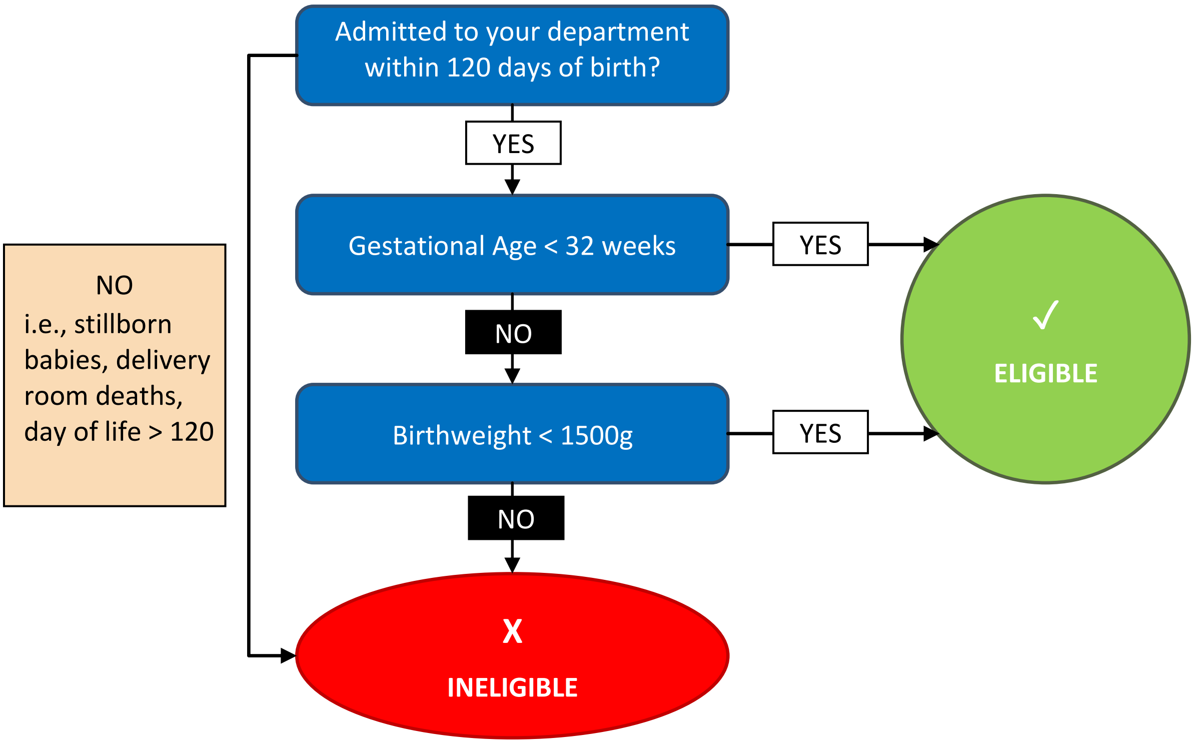

Figure 1: Decision flow to identify eligible infants for the core module denominator

Table 1: Examples of infants eligible/not eligible for core module once admitted to the ward

Day of Life	Birth Weight (grams)	Gestational Age (weeks+days)	Eligible for VLBW/VPT Database
120 ✓	1499 ✓	31+6 ✓	Yes ✓
1 ✓	1500 ✗	31+6 ✓	Yes ✓
120 ✓	1499 ✓	32+0 ✗	Yes ✓
1 ✓	1500 ✗	32+0 ✗	No ✗
121 ✗	1499 ✓	30+5 ✓	No ✗

An infant that is not eligible in the core module may still be eligible in the expanded module if you decided to also perform the extended surveillance described therein.

3.2 PATIENT DATA COLLECTION

3.2.1 Pseudonymisation table

Whenever an eligible patient is admitted or readmitted to one of the wards in your neonatology department, you should enrol this patient, preferably directly into the DHIS2 platform.

At the time of enrolment, you must assign each patient a NeoIPC ID number which is a unique ID that allows you to identify that patient within your department. This makes it possible for you to use the anonymous data stored in the system to refer to specific patients, e.g. in case of readmission or data correction, but makes it impossible for third parties to do the same.

Each patient can only have one NeoIPC identification number. When a patient is discharged and then readmitted, the initial follow-up ends at the time of discharge, and a new enrolment must be made using the same NeoIPC ID number when the patient is readmitted.

You are responsible for organizing patient data retrieval based on unique ids. We advise you to generate a pseudonymisation list that contains NeoIPC ID (unique random numbers) together with other identifying information (name, birthdate, patient id from the hospital information system) or to note that information on the paper-based patient surveillance master data sheet. You must store the pseudonymisation list and the paper sheets under the same conditions you store secret patient healthcare data and destroy it or the contained information in a secure way as soon as it is no longer needed. To ensure data privacy, you should never use an identifier that is used anywhere else and that you do not fully control (e.g. do NOT use the patient id from your hospital information system, patient name, or any unique identifier that is used elsewhere) while creating NeoIPC ID.

For the generation of reference reports, NeoIPC does not need to track individual patients and the IDs you assign will not be used by NeoIPC in any way.

Note: To create a pseudonymisation list you can use the templates (excel spreadsheet and paper datasheet) available on our website.

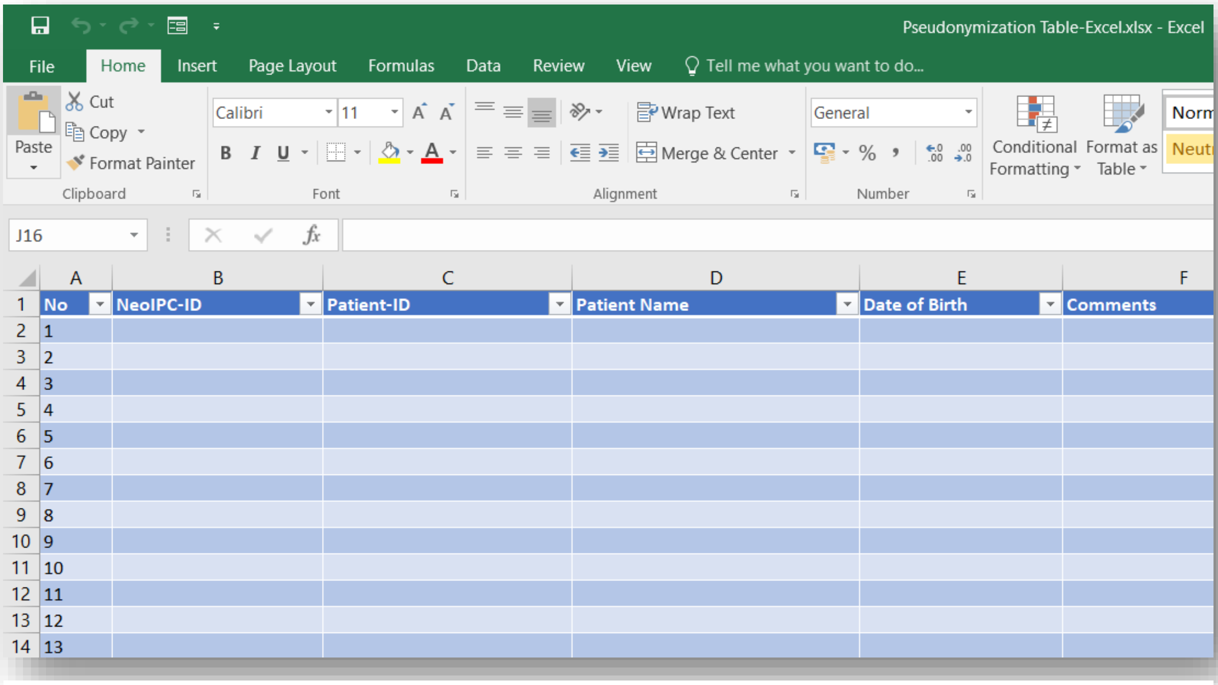

Figure 2: Pseudonymisation list (Excel)

NeoIPC – Core Module

Pseudonymization Table

NeoIPC

#	NeoIPC-ID	Patient-ID	Patient Name	Date of Birth	Comments

Figure 3: Pseudonymisation list (paper datasheet)

3.2.2 Master data collection sheet

Whenever an eligible patient is admitted or readmitted in your neonatology department, you should enrol this patient, preferably directly into the DHIS2 platform. After enrolling a patient, you must enter admission information and keep collecting the follow-up data using patient progress charts. When the patient is discharged, transferred to another hospital, or dies, you need to end the follow-up for that patient and the sum data in the patient progress charts will be the patient's surveillance end data.

You can use the master data collection sheet to save enrolment, admission information and surveillance end data locally. However, if you submit data to NeoIPC, the information you collect on the master data collection sheet must ultimately be entered into the DHIS2 platform.

NeoIPC – Core Module (VLBW/VPT Infants)

Master Data Collection Sheet

NeoIPC

Enrolment
**Patient ID:**
**Patient name:**
**Gestational age:** (weeks + days, e.g. 25+4)
**Birthweight:** grams
**Sex:**<br>○ Female<br>○ Male<br>○ Undetermined
**Delivery mode:**<br>○ Vaginal<br>○ Caesarean section (elective)<br>○ Caesarean section (emergency)
**Multiple birth:**<br>○ Yes, total number of infants at birth: ______________<br>○ No
**Admission Information**
**Admission date:**
**Admission type:**<br>○ Admitted from delivery room (initial admission for infants delivered in your hospital)<br>○ Transferred/readmitted to your hospital on the day of birth<br>○ Transferred/readmitted to your hospital the day after birth or later (Admission on day of life: __________)
**Surveillance End**
**Surveillance end date:**
**Reason:**<br>○ Discharge / Transfer<br>○ Death
**Patient days:**
**CVC days:**
**PVC days:**
**INV days:**
**NIV days:**
**Human milk days:**
**Kangaroo care days:**
**Probiotic days:**
**Antibiotic days (total):**
**Antibiotic days (per substance)**<br>Antibiotic substance 1: _____________________________: __________ days<br>Antibiotic substance 2: _____________________________: __________ days<br>Antibiotic substance 3: _____________________________: __________ days<br>Antibiotic substance 4: _____________________________: __________ days<br>Antibiotic substance 5: _____________________________: __________ days<br>Antibiotic substance 6: _____________________________: __________ days
**Comments:**

For more information, please see sections 5. *Data Dictionary* and 7. *Abbreviations* in the NeoIPC - Core Module Protocol.

Figure 4: Master data collection sheet for patient data collection

3.2.3 Patient progress chart

Eligible patients who have been enrolled in the system must be followed up using patient progress charts until death, transfer, or discharge. Cumulative risk and protective factors such as patient days, device days, and antibiotic days are recorded in the chart, if possible on a daily basis. This chart must be kept at your facility and to simplify data collection and patient follow-up. You must maintain patient progress chart(s) for every eligible infant during the surveillance period. It is possible to enter up to six different antibiotic substances in one chart. If your patient has received more than six types of antibiotics, please use an additional chart to document them.

When a patient is discharged, transferred to another hospital, or dies, you need to end the follow-up for that patient and the sum data in the patient progress charts will be the patient's surveillance end data. This data must be ultimately entered into DHIS2 platform under the event “surveillance end”. You can use the master data collection sheet to save this data locally.

For simplicity, patients who leave their department for up to two days (i.e., for surgery) are not considered transferred or discharged. You should record the data for days not spent in the department upon re-admission. If there are more than 48 hours between transfer and re-admission, you must end data collection for that patient and select "transfer" as the reason for the end of surveillance. In this case, any readmission should be recorded as a new admission, and the admission type must be selected as “transferred to your centre ≥ 24h postnatal”.

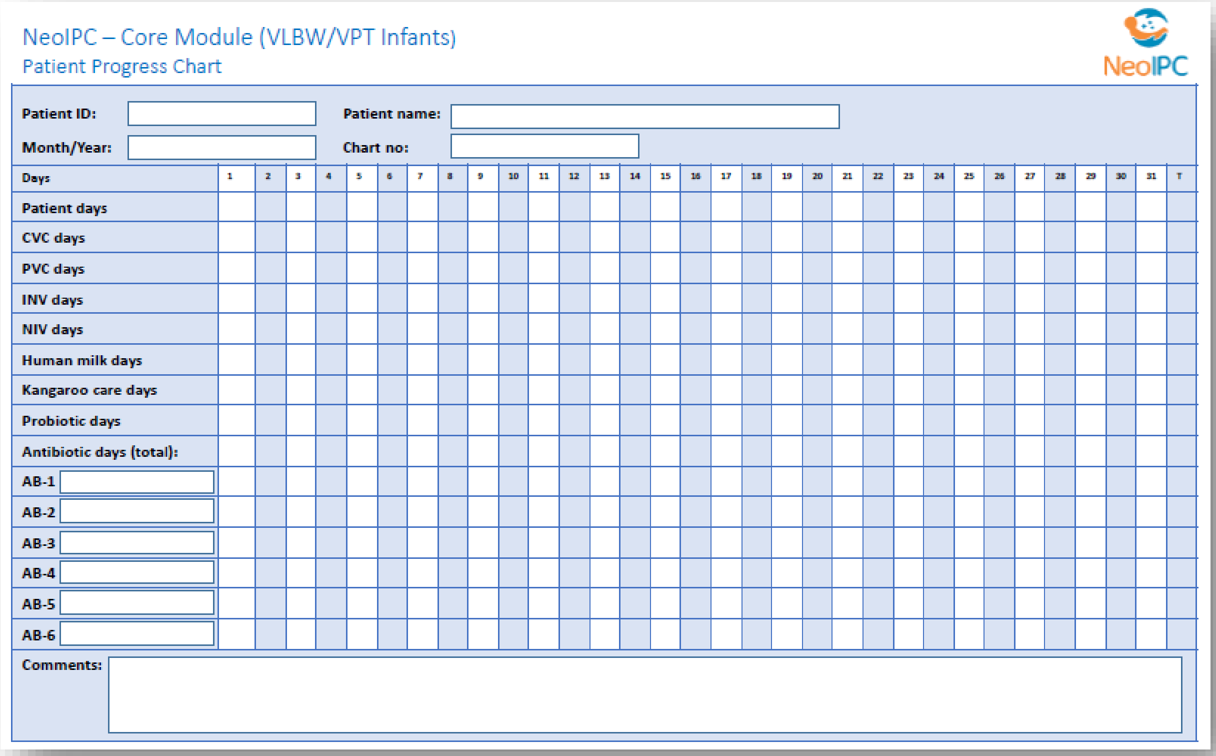
NeoIPC – Core Module (VLBW/VPT Infants)
Patient Progress Chart

NeoIPC

Patient ID: | Patient name:
Month/Year: | Chart no:

Days	1	2	3	4	5	6	7	8	9	10	11	12	13	14	15	16	17	18	19	20	21	22	23	24	25	26	27	28	29	30	31	T
Patient days																																
CVC days																																
PVC days																																
INV days																																
NIV days																																
Human milk days																																
Kangaroo care days																																
Probiotic days																																
Antibiotic days (total):																																
AB-1																																
AB-2																																
AB-3																																
AB-4																																
AB-5																																
AB-6																																

Comments:

Figure 5: Patient progress chart for patient data collection

3.3 SURGICAL PROCEDURE DATA COLLECTION

Whenever surgery must happen in neonates and especially in VLBW/VPT infants it is an indicator for a complicated course and an additional risk factor for nosocomial infections and the occurrence of multidrug-resistant pathogens. For this reason, every surgical procedure that is performed on an eligible infant is recorded in NeoIPC. You can use the surgical procedure paper sheet for local documentation or enter the respective information directly into the reporting system. In addition to the recording of the procedure, you need to start following up the concerned infant for surgical site infections for 30 or 90 days depending on whether an implant has been left in place during the procedure.

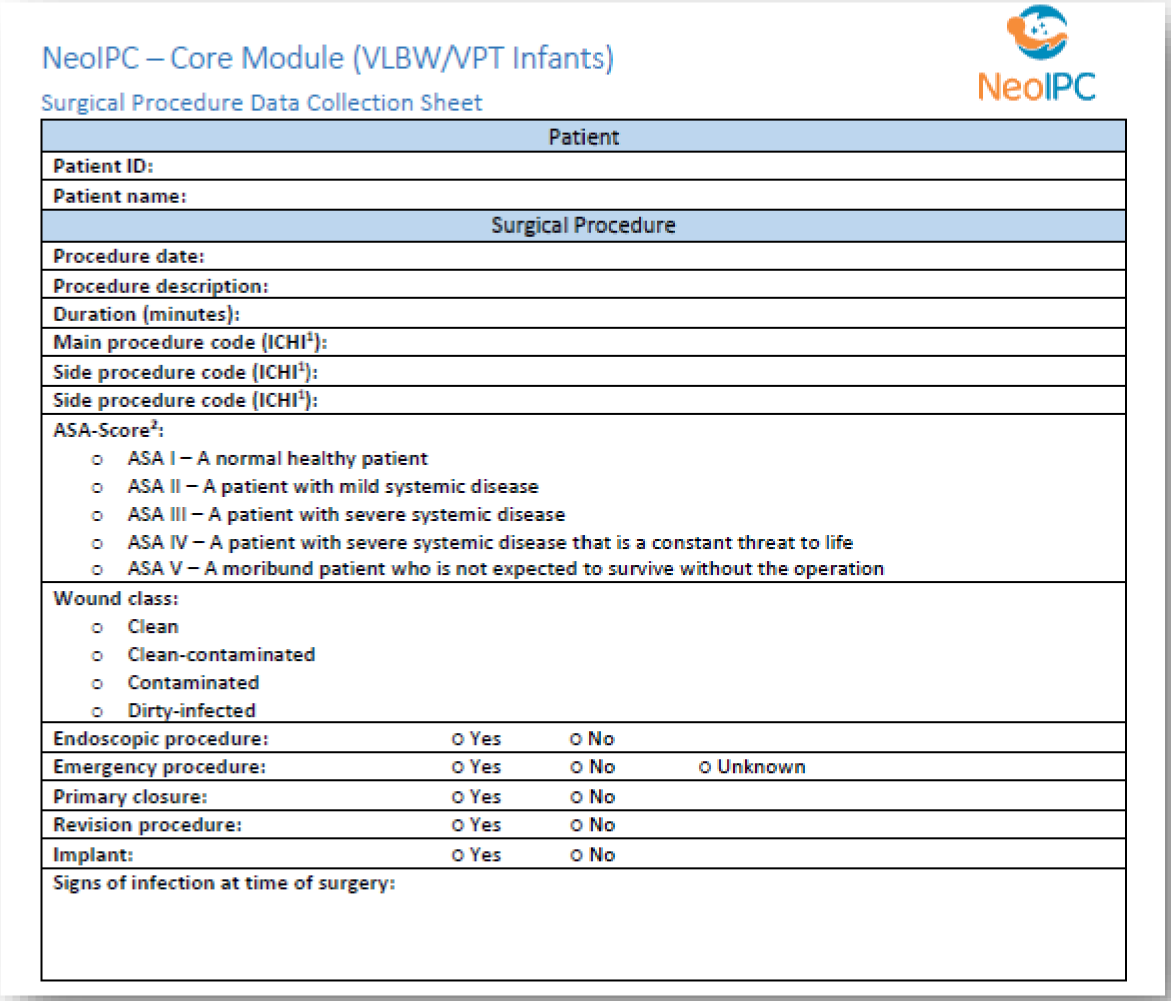

NeoIPC – Core Module (VLBW/VPT Infants)

Surgical Procedure Data Collection Sheet

NeoIPC

Patient			
**Patient ID:**			
**Patient name:**			
**Surgical Procedure**			
**Procedure date:**			
**Procedure description:**			
**Duration (minutes):**			
**Main procedure code (ICHI[1]):**			
**Side procedure code (ICHI[1]):**			
**Side procedure code (ICHI[1]):**			
**ASA-Score[2]:**<br>o ASA I – A normal healthy patient<br>o ASA II – A patient with mild systemic disease<br>o ASA III – A patient with severe systemic disease<br>o ASA IV – A patient with severe systemic disease that is a constant threat to life<br>o ASA V – A moribund patient who is not expected to survive without the operation			
**Wound class:**<br>o Clean<br>o Clean-contaminated<br>o Contaminated<br>o Dirty-infected			
**Endoscopic procedure:**	o Yes	o No	
**Emergency procedure:**	o Yes	o No	o Unknown
**Primary closure:**	o Yes	o No	
**Revision procedure:**	o Yes	o No	
**Implant:**	o Yes	o No	
**Signs of infection at time of surgery:**			

Figure 6: Surgical procedure data collection sheet

3.4 INFECTION DATA COLLECTION

Hospital-acquired bloodstream infections, pneumonia, NEC, and surgical site infections in eligible patients should be documented until the end of the surveillance period.

Neonatal infections can broadly be separated into early-onset and late-onset infections. Most of the early-onset infections result from pregnancy- and delivery-associated risks where the causative pathogens typically affect the mother in the form of microbial colonisation or infection before causing an infection in the child. Most of the late-onset infections, in contrast, result from healthcare-associated risks, and the pathogens causing these infections typically stem from persons or inanimate surfaces that get in close contact with the infant during neonatal care. Since only the latter are immediately actionable for the healthcare professionals working on neonatology wards, NeoIPC puts a focus on those “nosocomial” late-onset infections but tries to also support you, when trying to perform surveillance of early-onset infections. Although a fixed time-based cut-off value does not exactly capture whether an infection was acquired from the NICU environment, there is a relatively broad international consensus that a 72-hour cut-off value serves as a good estimator for nosocomial infections in neonatology. **For this reason, infections, where the first symptoms occur within a 72-hour window after birth, should not be considered nosocomial infections and therefore should not be recorded in the NeoIPC core module**. It is, however, possible to record them separately as early-onset infections if you opt into the NeoIPC early-onset infection module. If an infection begins before 72 hours and is clearly hospital-acquired (i.e., there are clear signs of infection on the catheter entry site) or starts after 72 hours and is clearly vertical (e.g., all transplacental infections that are not evident at birth, like toxoplasmosis, CMV, HIV, rubella, and syphilis), this cut-off value can be ignored.

For patients that are transferred or re-admitted to a ward in your neonatology department, there is an additional cut-off value that needs to be applied. In this case, an infection is only considered a nosocomial infection **if the day of symptom onset is greater than or equal to day 3 of the patient’s hospital stay**, where the day of admission is regarded as day 1.

NeoIPC guidelines do not offer a strict timeframe for elements of definitions to occur but usually, all elements required to meet an infection definition usually occur within a 7-10 day timeframe with typically no more than 2-3 days between elements.

In the presence of a previously recorded infection, when a new pathogen is isolated in the same organ system, we cannot record this as a new infection. A minimum of 14 days and a period without any relevant symptoms of infection are required to register the same type of infection again.

3.4.1 Primary sepsis/BSI

If an eligible infant develops a hospital-acquired primary sepsis/bloodstream infection according to the NeoIPC surveillance criteria (see Infection Definitions: Primary sepsis/bloodstream infection) during the surveillance period, you should record and submit the required data using the data collection sheet below and/or via the online reporting platform DHIS2.

NeoIPC – Infection Data Collection Sheet

Hospital-Acquired Primary Sepsis/BSI

NeoIPC

Patient
**Patient ID:**
**Patient name:**
Hospital-acquired Primary Sepsis/BSI
**Infection date:**
**Vascular catheter association:**<br>○ No ○ CVC-associated ○ PVC-associated
**BSI type:**<br>○ Clinical Sepsis *(no positive blood/cerebrospinal fluid culture)*<br>○ LCBSI-RP *(caused by a recognised pathogen)*<br>○ LCBSI-CC *(caused by a common commensal), recovered:* ○ only once or ○ at least twice
**Intravenous antibiotic therapy for five or more days initiated:**<br>○ Yes<br>○ No
**Please enter organism(s) recovered, if you chose LCBSI-RP or LCBSI-CC:**<br>Organism 1: ____________________, recovered from ☐ Blood ☐ CSF<br>▪ MRSA/VRE/3GCR[1] ○ Yes ○ No ○ Not tested<br>▪ Carbapenem resistant ○ Yes ○ No ○ Not tested<br>▪ Colistin resistant ○ Yes ○ No ○ Not tested<br>Organism 2: ____________________, recovered from ☐ Blood ☐ CSF<br>▪ MRSA/VRE/3GCR[1] ○ Yes ○ No ○ Not tested<br>▪ Carbapenem resistant ○ Yes ○ No ○ Not tested<br>▪ Colistin resistant ○ Yes ○ No ○ Not tested<br>Organism 3: ____________________, recovered from ☐ Blood ☐ CSF<br>▪ MRSA/VRE/3GCR[1] ○ Yes ○ No ○ Not tested<br>▪ Carbapenem resistant ○ Yes ○ No ○ Not tested<br>▪ Colistin resistant ○ Yes ○ No ○ Not tested
**Signs and symptoms of generalized infection:**<br>☐ Temperature instability or fever (>38 °C) or hypothermia (<36.5 °C)<br>☐ Unexplained tachycardia (>200/min) or new/more frequent bradycardia episodes (<80/min)<br>☐ Capillary refill time of > 3s or skin mottling or core/peripheral temperature gap > 2 °C<br>☐ New/more frequent episodes of apnoea (>20s) or increase in oxygen demand or ventilatory support<br>☐ Enteral feeding intolerance, abdominal distension or ileus<br>☐ Irritability, lethargy, apathy or unstable condition<br>☐ Unexplained metabolic acidosis (base excess < −10 mmol/L; <−10 mEq/L)<br>☐ New and unexplained hyperglycaemia (> 140 mg/dl; > 7.8 mmol/L) or hypoglycaemia (< 40 mg/dl; <2.2 mmol/L)
**Laboratory findings:**<br>☐ Platelet count of < 100×10^9/L (<100×10^3/µL)<br>☐ WBC < 4×10^9/L or > 20×10^9/L (< 4×10^3/µL or > 20×10^3/µL)<br>☐ CRP > 10 mg/L (> 1 mg/dL)<br>☐ Procalcitonin ≥ 2µg/L (2 ng/mL; 200 ng/dL)<br>☐ I/T-Ratio > 0,2 (ratio of immature granulocytes to total granulocytes)<br>☐ Increased levels of interleukin 6 (IL-6) or IL-8

Figure 7: BSI reporting sheet

3.4.2 Necrotizing enterocolitis

If an eligible infant develops a necrotizing enterocolitis according to the NeoIPC surveillance criteria (see Infection Definitions: Necrotizing Enterocolitis (NEC)) during the surveillance period, you should record and submit the required data using the data collection sheet below and/or via the online reporting platform DHIS2.

NeoIPC – Infection Data Collection Form

Necrotizing Enterocolitis (NEC)

NeoIPC

Patient	
**Patient ID:**	
**Patient name:**	
**Necrotizing Enterocolitis**	
**Infection date:**	
**Radiological signs (imaging technologies: X-ray, CT, MRI, ultrasound):**<br>□ Pneumoperitoneum<br>□ Pneumatosis intestinalis<br>□ Portal venous gas (Hepatobiliary gas)<br>□ Fixed bowel loops (≥ 24h)	
**Clinical signs and symptoms:**<br>□ Abdominal distention<br>□ Abdominal discoloration or shiny/reddish skin tone<br>□ Repeated occult (guaiac test) or visible blood in stool (no anal fissure)	□ Increasing/pronounced vomiting<br>□ Increased gastric residuals from previous feeding<br>□ Bilious gastric aspirate (not from transpyloric feeding tube)
**Surgical or pathological findings:**<br>□ Extensive bowel necrosis (> 2 cm of bowel affected)<br>□ Pneumatosis intestinalis	□ Intestinal perforation (not a definition criterion)

Secondary BSI:

o Yes o No o No follow-up

If you chose YES, please enter organism(s) recovered from blood culture:

Organism 1: ____________

- MRSA/VRE/3GCR[1] o Yes o No o Not tested
- Carbapenem resistant o Yes o No o Not tested
- Colistin resistant o Yes o No o Not tested

Organism 2: ____________

- MRSA/VRE/3GCR[1] o Yes o No o Not tested
- Carbapenem resistant o Yes o No o Not tested
- Colistin resistant o Yes o No o Not tested

Organism 3: ____________

- MRSA/VRE/3GCR[1] o Yes o No o Not tested
- Carbapenem resistant o Yes o No o Not tested
- Colistin resistant o Yes o No o Not tested

Figure 8: NEC reporting sheet

3.4.3 Pneumonia

If an eligible infant develops a hospital-acquired pneumonia according to the NeoIPC surveillance criteria (see Infection Definitions: Pneumonia) during the surveillance period, you should record and submit the required data using the data collection sheet below and/or via the online reporting platform DHIS2.

NeoIPC – Infection Data Collection Sheet

Pneumonia (PN)

NeoIPC

Patient
**Patient ID:**
**Patient name:**
Hospital-acquired Pneumonia
**Infection date:**
**Device association:**<br>○ No ○ INV-associated ○ NIV-associated
**Organisms identified from respiratory tract (RT):**<br>○ Yes ○ No ○ Not tested<br>**If you chose YES, please enter the organism(s):**<br>**Organism 1:** ________________, recovered from ☐ lower RT ☐ upper RT<br>▪ MRSA/VRE/3GCR[1] ○ Yes ○ No ○ Not tested<br>▪ Carbapenem resistant ○ Yes ○ No ○ Not tested<br>▪ Colistin resistant ○ Yes ○ No ○ Not tested<br>**Organism 2:** ________________, recovered from ☐ lower RT ☐ upper RT<br>▪ MRSA/VRE/3GCR[1] ○ Yes ○ No ○ Not tested<br>▪ Carbapenem resistant ○ Yes ○ No ○ Not tested<br>▪ Colistin resistant ○ Yes ○ No ○ Not tested<br>**Organism 3:** ________________, recovered from ☐ lower RT ☐ upper RT<br>▪ MRSA/VRE/3GCR[1] ○ Yes ○ No ○ Not tested<br>▪ Carbapenem resistant ○ Yes ○ No ○ Not tested<br>▪ Colistin resistant ○ Yes ○ No ○ Not tested
☐ At least one of the following imaging findings (imaging technologies: X-ray, CT, MRI, ultrasound) shows new changes suggestive of pneumonia, such as infiltrate, shadowing, opacification, increased density, fluid in the intrapleural cavity or interlobar fissure
☐ New initiation of respiratory support or escalation of existing level of respiratory support for ≥ 2 days after at least 2 days of stability or improvement
**Clinical and laboratory criteria:**<br>☐ New/more frequent bradycardia episodes (<80/min) or unexplained tachycardia (>200/min)<br>☐ New or increased frequency of episodes of apnoea (> 20 s) or new or more frequent tachypnoea (>60/min).<br>☐ Purulent tracheal aspirate<br>☐ New or more frequent symptoms of respiratory distress (retraction, nasal flaring, grunting, chest indrawing)<br>☐ Temperature instability or fever (>38 °C) or hypothermia (<36.5 °C)<br>☐ Increased respiratory secretion (more frequent endotracheal suctioning required)<br>☐ CRP > 10 mg/L (> 1 mg/dl) or increased levels of interleukin 6 (IL-6) or IL-8<br>☐ I/T - ratio > 0.2
**Secondary BSI:**<br>○ Yes ○ No ○ No follow-up<br>**If you chose YES, please enter organism(s) recovered from blood culture:**<br>**Organism 1:** ________________<br>▪ MRSA/VRE/3GCR[1] ○ Yes ○ No ○ Not tested<br>▪ Carbapenem resistant ○ Yes ○ No ○ Not tested<br>▪ Colistin resistant ○ Yes ○ No ○ Not tested<br>**Organism 2:** ________________<br>▪ MRSA/VRE/3GCR[1] ○ Yes ○ No ○ Not tested<br>▪ Carbapenem resistant ○ Yes ○ No ○ Not tested<br>▪ Colistin resistant ○ Yes ○ No ○ Not tested<br>**Organism 3:** ________________<br>▪ MRSA/VRE/3GCR[1] ○ Yes ○ No ○ Not tested<br>▪ Carbapenem resistant ○ Yes ○ No ○ Not tested<br>▪ Colistin resistant ○ Yes ○ No ○ Not tested

Figure 9: Pneumonia reporting sheet

3.4.4 Surgical site infections

If an eligible infant develops a surgical site infection according to the NeoIPC surveillance criteria (see Definitions: Surgical Site Infection (SSI)) during the surveillance period, you should record and submit the required data using the data collection sheet below and/or via the online reporting platform DHIS2.

NeoIPC – Infection Data Collection Sheet

Surgical Site Infection (SSI)

NeoIPC

Patient	
Patient ID:	
Patient name:	
**Surgical Site Infection**	
Infection date:	
SSI type:<br>○ Superficial (skin, subcutaneous)<br>○ Deep (fascial and muscle)<br>○ Organ/Space (deeper than fascial/muscle)	Infection present at time of surgery:<br>○ Yes<br>○ No
Organism(s) identified from surgical site:<br>○ Yes ○ No ○ Not tested<br>If you chose YES, please enter the organism(s):<br>Organism 1: __________<br>▪ MRSA/VRE/3GCR[1] ○ Yes ○ No ○ Not tested<br>▪ Carbapenem resistant ○ Yes ○ No ○ Not tested<br>▪ Colistin resistant ○ Yes ○ No ○ Not tested<br>Organism 2: __________<br>▪ MRSA/VRE/3GCR[1] ○ Yes ○ No ○ Not tested<br>▪ Carbapenem resistant ○ Yes ○ No ○ Not tested<br>▪ Colistin resistant ○ Yes ○ No ○ Not tested<br>Organism 3: __________<br>▪ MRSA/VRE/3GCR[1] ○ Yes ○ No ○ Not tested<br>▪ Carbapenem resistant ○ Yes ○ No ○ Not tested<br>▪ Colistin resistant ○ Yes ○ No ○ Not tested	
Clinical signs and symptoms:<br>□ Purulent drainage from the incision<br>□ Purulent drainage from a drain<br>□ Incision deliberately opened or aspirated<br>□ Incision spontaneously dehisces<br>□ Abscess or other evidence of infection	□ Localized pain or tenderness<br>□ Localized swelling<br>□ Localized erythema<br>□ Localized heat<br>□ Fever (> 38 °C) or hypothermia (< 36.5 °C) or temperature instability
Secondary BSI:<br>○ Yes ○ No ○ No follow-up<br>If you chose YES, please enter organism(s) recovered from blood culture:<br>Organism 1: __________<br>▪ MRSA/VRE/3GCR[1] ○ Yes ○ No ○ Not tested<br>▪ Carbapenem resistant ○ Yes ○ No ○ Not tested<br>▪ Colistin resistant ○ Yes ○ No ○ Not tested<br>Organism 2: __________<br>▪ MRSA/VRE/3GCR[1] ○ Yes ○ No ○ Not tested<br>▪ Carbapenem resistant ○ Yes ○ No ○ Not tested<br>▪ Colistin resistant ○ Yes ○ No ○ Not tested<br>Organism 3: __________<br>▪ MRSA/VRE/3GCR[1] ○ Yes ○ No ○ Not tested<br>▪ Carbapenem resistant ○ Yes ○ No ○ Not tested<br>▪ Colistin resistant ○ Yes ○ No ○ Not tested	

Figure 10: SSI reporting sheet

3.4.5 Device-associated infection

Intravenous therapy and mechanical ventilation are established risk factors for nosocomial infections in neonatology. These kinds of infections are typically considered device-associated infections and are recorded in association with medical devices like vascular catheters or intubation.

In NeoIPC the following device associations are recorded:

1. Invasive ventilation (INV) associated infections
2. Non-invasive ventilation (NIV) associated infections
3. Central venous catheter (CVC) associated infections
4. Peripheral venous catheter (PVC) associated infections

Since the establishment of a causative relationship between a device and a hospital-acquired infection is difficult and infeasible in many neonatology settings, NeoIPC focuses on a time-based association only. To create an association between a device and infection, the device must have been in use for a defined period prior to infection.

A device association exists if the device has been present for at least three consecutive days on the day of infection (=first symptoms or first positive diagnostic test; e.g. culture) or the day before.

Table 2: Example showing the relationship between infection and device for device associated infections

Days	Device in place	Device Association (if this is the day of infection)	Comments
**Day 1**	**CVC**	No	<3 CVC days on the day of infection
**Day 2**	**CVC**	No	<3 CVC days on the day of infection
**Day 3**	**CVC**	Yes	≥3 CVC days on the day of infection
Day 4	No device	Yes	No CVC on the day of infection but ≥3 CVC days on the Day 3 (the day before infection)
Day 5	No device	No	No CVC on the day of infection or on day before infection
Day 6	No device	No	No CVC on the day of infection or on day before infection

If a patient developing a bloodstream infection meets the criteria for both, PVC and CVC association, the CVC is considered as the device with the relatively higher infection risk and the BSI should be recorded as CVC-associated BSI.

If both invasive and non-invasive ventilation were used intermittently, pneumonia should be recorded as INV-associated pneumonia.

3.4.6 Secondary bloodstream infection

Within NeoIPC surveillance, it is possible to record bloodstream infections secondary to pneumonia, NEC, and SSIs. Collecting data on secondary BSI is optional; therefore, it is possible to select **“Unknown”** if you are not following patients for secondary BSI.

To assign a secondary bloodstream infection to a pneumonia, NEC, or an SSI, the following criteria must be met:

1. The blood specimen is collected in the period between 3 days prior and 13 days after the day of primary infection (day of primary infection=first symptoms or first positive culture at the primary infection site).

And,

2. At least one organism from the blood specimen matches an organism identified from the primary infection site.

Secondary BSI:

o Yes o No o No follow-up

If you chose YES, please enter organism(s) recovered from blood culture:

Organism 1: ____________________

- MRSA/VRE/3GCR[1] o Yes o No o Not tested
- Carbapenem resistant o Yes o No o Not tested
- Colistin resistant o Yes o No o Not tested

Organism 2: ____________________

- MRSA/VRE/3GCR[1] o Yes o No o Not tested
- Carbapenem resistant o Yes o No o Not tested
- Colistin resistant o Yes o No o Not tested

Organism 3: ____________________

- MRSA/VRE/3GCR[1] o Yes o No o Not tested
- Carbapenem resistant o Yes o No o Not tested
- Colistin resistant o Yes o No o Not tested

Figure 11: Secondary BSI data collection

4 INFECTION DEFINITIONS

4.1 PRIMARY SEPSIS/BLOODSTREAM INFECTION

Sepsis is classified as either culture negative or culture proven in the NeoIPC surveillance system. As well, culture proven sepsis (LCBSI) is classified into two categories based on the culture result.

Infections caused by microorganisms that enter the bloodstream from a primary infection site (except for catheter-associated infections) are not counted in this section. For detailed information, please see Secondary bloodstream infection.

1. Clinical sepsis (infection without a detected organism)
2. Laboratory-confirmed bloodstream infection (LCBSI)
    a. LCBSI caused by a recognised pathogen*
    b. LCBSI caused by a common commensal*

*See *Master Organism List* on *https://neoipc.org/surveillance/resources/*

4.1.1 Clinical sepsis

1. **Absence of positive microbiological blood and/or cerebrospinal fluid culture**

   AND

2. **Treatment with FIVE or more days of intravenous antibiotics was initiated***

   AND

3. **Patient has at least TWO of the following clinical or laboratory features of generalized infection:**

   - Temperature instability, fever (> 38 °C) or hypothermia (< 36.5 °C)
   - New/more frequent bradycardia episodes (<80/min) or unexplained tachycardia (>200/min)
   - Impaired peripheral perfusion (Capillary refill time of > 3s or skin mottling or core/peripheral temperature gap > 2 °C)
   - New/more frequent episodes of apnoea (>20s) or increase in oxygen demand or ventilatory support
   - Enteral feeding intolerance, abdominal distension or ileus
   - Irritability, lethargy, apathy or unstable condition
   - Unexplained metabolic acidosis (base excess < −10 mmol/L; <−10 mEq/L)
   - New and unexplained hyperglycaemia (> 140 mg/dl; > 7.8 mmol/L) or hypoglycaemia (< 40 mg/dl; <2.2 mmol/L)
   - At least one of the following laboratory findings:
     - Platelet count of < 100 × 10^9/L (<100 × 10^3/µL)
     - WBC < 4 × 10^9/L or > 20 × 10^9/L (< 4 × 10^3/µL or > 20 × 10^3/µL)
     - CRP > 10 mg/L (> 1 mg/dL)
     - Procalcitonin ≥ 2µg/L (2 ng/mL; 200 ng/dL)
     - I/T-Ratio > 0,2 (ratio of immature granulocytes to total granulocytes)
     - Increased levels of interleukin 6 (IL-6) or IL-8

* Antibiotic treatment for at least five days was initiated. The day of the first dose and the day of the last dose are counted. Days where no dose was administered between the first and the last dose (e.g., skipped doses because of high drug levels in therapeutic drug monitoring) are counted as if a dose had been administered. Days after the last dose are not counted regardless of the patient's measured or assumed drug level. If the infant died, was discharged, or transferred before the end of the five-day course of intravenous antibiotics, this condition is met if treatment was scheduled for five days or more.

4.1.2 LCBSI caused by a recognised pathogen

1. **Recognised pathogen is recovered from a blood and/or cerebrospinal fluid culture**

4.1.3 LCBSI caused by common commensals

If blood cultures show growth of one or multiple common commensals (e.g., coagulase-negative staphylococci), it is unclear in many cases, if the detected bacteria are the causative agent of a suspected infection or if a contamination leads to a wrong diagnosis. There have been multiple approaches to solve this issue by adapting surveillance definitions to this fact. Unfortunately, all these approaches are limited by diagnostic habits and availability of resources, rendering comparison of data from different settings close to impossible.

Since NeoIPC aims at providing tools and definitions that work in a wide range of different settings we offer 3 different definitions for LCBSI caused by common commensals that can be used in a wide range of settings. As the tools collect the raw data leading to the diagnosis of an infection it is possible to assess the application of different definitions in various settings and possibly assess the effect on calculated incidences.

In any case, because of this fact, you should be very careful when comparing rates of LCBSI caused by common commensals across different settings.

1. **The same common commensal is recovered from at least TWO blood culture and/or CSF culture specimen collected on separate occasions**

AND

2. **Patient has at least TWO of the following clinical or laboratory features of generalized infection:**
   - Temperature instability, fever (> 38 °C) or hypothermia (< 36.5 °C)
   - New/more frequent bradycardia episodes (<80/min) or unexplained tachycardia (>200/min)
   - Impaired peripheral perfusion (Capillary refill time of > 3s or skin mottling or core/peripheral temperature gap > 2 °C)
   - New/more frequent episodes of apnoea (>20s) or increase in oxygen demand or ventilatory support
   - Enteral feeding intolerance, abdominal distension or ileus
   - Irritability, lethargy, apathy or unstable condition
   - Unexplained metabolic acidosis (base excess < −10 mmol/L; <−10 mEq/L)
   - New and unexplained hyperglycaemia (> 140 mg/dl; > 7.8 mmol/L) or hypoglycaemia (< 40 mg/dl; <2.2 mmol/L)
   - At least one of the following laboratory findings:
     - Platelet count of < 100 × 10^9/L (<100 × 10^3/µL)
     - WBC < 4 × 10^9/L or > 20 × 10^9/L (< 4 × 10^3/µL or > 20 × 10^3/µL)
     - CRP > 10 mg/L (> 1 mg/dL)
     - Procalcitonin ≥ 2µg/L (2 ng/mL; 200 ng/dL)
     - I/T-Ratio > 0,2 (ratio of immature granulocytes to total granulocytes)
     - Increased levels of interleukin 6 (IL-6) or IL-8

OR

1. **A common commensal is recovered from ONE blood culture and/or CSF culture specimen**

AND

2. **At least one of the following laboratory findings:**
   - WBC < 4 × 10^9/L or > 20 × 10^9/L (< 4 × 10^3/µL or > 20 × 10^3/µL)
   - CRP > 10 mg/L (> 1 mg/dL)
   - Procalcitonin ≥ 2µg/L (2 ng/mL; 200 ng/dL)
   - I/T-Ratio > 0,2 (ratio of immature granulocytes to total granulocytes)
   - Increased levels of interleukin 6 (IL-6) or IL-8

AND

3. **Patient has at least TWO of the following clinical or laboratory features of generalized infection:**
   - Temperature instability, fever (> 38 °C) or hypothermia (< 36.5 °C)
   - New/more frequent bradycardia episodes (<80/min) or unexplained tachycardia (>200/min)
   - Impaired peripheral perfusion (Capillary refill time of > 3s or skin mottling or core/peripheral temperature gap > 2 °C)
   - New/more frequent episodes of apnoea (>20s) or increase in oxygen demand or ventilatory support
   - Enteral feeding intolerance, abdominal distension or ileus
   - Irritability, lethargy, apathy or unstable condition
   - Unexplained metabolic acidosis (base excess < −10 mmol/L; <−10 mEq/L)
   - New and unexplained hyperglycaemia (> 140 mg/dl; > 7.8 mmol/L) or hypoglycaemia (< 40 mg/dl; <2.2 mmol/L)
   - Platelet count of < 100 × 10^9/L (<100 × 10^3/µL)

OR

1. **A common commensal is recovered from ONE blood culture and/or CSF culture specimen**

AND

2. **Treatment with five or more days of intravenous antibiotics was initiated***

AND

3. **Patient has at least TWO of the following clinical or laboratory features of generalized infection:**
   - Temperature instability, fever (> 38 °C) or hypothermia (< 36.5 °C)
   - New/more frequent bradycardia episodes (<80/min) or unexplained tachycardia (>200/min)
   - Impaired peripheral perfusion (Capillary refill time of > 3s or skin mottling or core/peripheral temperature gap > 2 °C)
   - New/more frequent episodes of apnoea (>20s) or increase in oxygen demand or ventilatory support
   - Enteral feeding intolerance, abdominal distension or ileus
   - Irritability, lethargy, apathy or unstable condition
   - Unexplained metabolic acidosis (base excess < −10 mmol/L; <−10 mEq/L)
   - New and unexplained hyperglycaemia (> 140 mg/dl; > 7.8 mmol/L) or hypoglycaemia (< 40 mg/dl; <2.2 mmol/L)
   - At least one of the following laboratory findings:
     - Platelet count of < 100 × 10^9/L (<100 × 10^3/µL)
     - WBC < 4 × 10^9/L or > 20 × 10^9/L (< 4 × 10^3/µL or > 20 × 10^3/µL)
     - CRP > 10 mg/L (> 1 mg/dL)
     - Procalcitonin ≥ 2µg/L (2 ng/mL; 200 ng/dL)
     - I/T-Ratio > 0,2 (ratio of immature granulocytes to total granulocytes)
     - Increased levels of interleukin 6 (IL-6) or IL-8

*If the infant died, was discharged, or transferred prior to the completion of five days of intravenous antibiotics, this condition would still be met if the intention were to treat for five or more days.

4.2 NECROTIZING ENTEROCOLITIS (NEC)

NEC surveillance criteria consist of either a combination of radiological findings and clinical signs or a diagnosis based on surgical evidence. The NEC dataset includes information on whether the patient has an intestinal perforation. However, this is not one of the surveillance definition criteria. This means that you should not report cases where you have surgical evidence of intestinal perforation without evidence of primary necrosis or pneumatosis intestinalis (e.g. spontaneous bowel perforation) as NEC.

1. **At least of ONE the following radiological signs (imaging technologies: X-ray, CT, MRI, ultrasound):**
   - Pneumoperitoneum,
   - Pneumatosis intestinalis,
   - Portal venous gas (Hepatobiliary gas),
   - Fixed bowel loops (≥ 24h)

AND

2. **At least ONE of the following clinical signs:**
   - Abdominal distention
   - Abdominal discoloration or shiny/reddish skin tone,
   - Repeated occult (guaiac test) or visible blood in stool (no anal fissure),
   - Increasing/pronounced vomiting (e.g. bilious or bloody)
   - Increased gastric residuals from previous feeding
   - Bilious gastric aspirate (not from transpyloric feeding tube)

OR

1. **At least of ONE the following surgical or pathological findings:**
   - Extensive bowel necrosis (> 2 cm of bowel affected)
   - Pneumatosis intestinalis

4.3 PNEUMONIA

Pneumonia in neonates is an entity that is very difficult to define for surveillance purposes as one can infer from the fact that many surveillance systems abstain from recording it at all, while others record ventilator-associated events where surveillance is limited to ventilated patients and pneumonia is only one of the entities captured by the definition.

1. **At least ONE of the following imaging findings (imaging technologies: X-ray, CT, MRI, ultrasound) shows <u>new changes</u> suggestive of pneumonia, such as infiltrate, shadowing, opacification, increased density, fluid in the intrapleural cavity or interlobar fissure**

AND

2. **New initiation of respiratory support or escalation of existing level of respiratory support for ≥ 2 days* after at least 2 days of stability or improvement**

AND

3. **At least FOUR of the following clinical or laboratory criteria:**

- Organisms identified⁺ from respiratory tract
- New/more frequent bradycardia episodes (<80/min) or unexplained tachycardia (>200/min)
- New or more frequent tachypnoea (>60/min) or new or more frequent apnoea (> 20 s)
- Purulent tracheal aspirate
- New or more frequent symptoms of respiratory distress (retraction, nasal flaring, grunting, chest indrawing)
- Temperature instability or fever (>38 °C) or hypothermia (<36.5 °C)
- Increased respiratory secretion (more frequent endotracheal suctioning required)
- CRP > 10 mg/L (> 1 mg/dL) or increased levels of interleukin 6 (IL-6) or IL-8[#]
- I/T - ratio > 0.2

* New initiation of respiratory support or escalation of existing level of respiratory support that does not improve within less than two days:

□ Increase in need for FiO_2 ≥ 0.25 (25 points) within 24 hours (daily minimum FiO_2 values must be taken into account)
OR
□ begin of non-invasive ventilatory support (excluding switch from invasive ventilation)
OR
□ begin of invasive mechanical ventilation (including switch from non-invasive ventilatory support)

<u>….that does not improve within less than 2 days:</u> The above-mentioned condition should not improve within two days.
<u>…after at least 2 days of stability or improvement:</u> A stable or improving baseline period of at least two days is required before the above condition occurs.

[+] At least one organism (see below) has been identified from respiratory tract by a culture or non-culture based microbiologic testing method which is performed for purposes of clinical diagnosis or treatment (for example, NOT Active Surveillance Culture/Testing (ASC/AST)):

- ◻ Fungal or bacterial pathogen from secretions of lower respiratory tract
  OR
- ◻ Viral gene, antigen or antibody from secretions of upper or lower respiratory tract (e.g. EIA, FAMA, shell vial assay, PCR)

Interleukin should be used as a parameter when laboratory specifications for a pathological value have been fulfilled.

Note: To meet the criteria of device associated hospital-acquired pneumonia, patients must be ventilated for at least 4 calendar days (day 1 is the day invasive/non-invasive ventilation starts). The earliest date of the event is day 3 of ventilation.

Examples:

Table 3: Hospital-acquired pneumonia- no device association

**Patient day**	**Patient´s condition**	**Comments**
**1**	No device, stable	
**2**	No device, stable	Baseline day 1
**3**	No device, stable	Baseline day 2
**4**	Deteriorating -> CPAP	✓ (infection day)
**5**	No improvement, CPAP continues	

Table 4: Hospital-acquired pneumonia - device associated

**NIV/INV Day**	**Daily minimum FiO2 (oxygen concentration, %)**	**Comments**
**1**	1.00	
**2**	1.00	
**3**	0.70 (improving)	Baseline Day 1
**4**	0.60 (improving)	Baseline Day 2
**5**	0.90	✓ (infection day)
**6**	0.90	No improvement

4.4 SURGICAL SITE INFECTION (SSI)

A wound infection that occurs at the incision or surgical site is recorded as a surgical site infection if it occurs in a certain time window after a surgical procedure that was also recorded in the NeoIPC surveillance system.

SSIs occurring in patients without a surgical procedure record in the NeoIPC surveillance system (e.g. a patient transferred to your centre after a surgical procedure performed at another centre) are not eligible.

The following surveillance times apply in detail (day 1 = the procedure date):

- Superficial incisional SSI: 30 days
- Deep incisional SSI: 30 days or 90 days if an implant has been left in place
- Organ/Space SSI: 30 days or 90 days if an implant has been left in place

Follow-up for SSIs can be terminated preliminarily for a surgical procedure for two reasons:

- Death, transfer, or discharge of the patient.
- A revision procedure in the same area. This will start a new follow-up period for the revision procedure though.

Minor interventions such as simple punctures of hematoma/seroma do not count as revision procedures. Surveillance cannot be terminated by such minor procedures, and they do not start a new follow-up period.

The following inclusion criteria apply to all SSIs:

- Record the main procedure and the associated ICHI[1] code. Only in complex surgical interventions where one main procedure is not sufficient to adequately describe the procedure, up to 2 further ICHI codes can be recorded.
- The type of SSI (superficial incisional, deep incisional, or organ/space) reported must reflect the deepest tissue level where SSI criteria are met during the surveillance period.

  Example: An SSI started as a deep incisional SSI on day 10 of the SSI surveillance period and then a week later (Day 17) meets the criteria for an organ/space SSI. You must report it as organ/space SSI regardless of superficial or deep tissue involvement. The day of infection in this case would be “Day 17”.
- The patient must not be deceased (e.g., post-mortem surgery in case of organ donation is excluded).

---

[1] International Classification of Health Interventions
https://www.who.int/standards/classifications/international-classification-of-health-interventions

4.4.1 Superficial incisional SSI

Surgical site infections involving only the skin and subcutaneous tissue belong to this category.

1. **First symptoms occur within 30 days after the operation**

AND

2. **Infection involves only skin and subcutaneous tissue of the incision**

AND

3. **Patient has at least ONE of the following:**
   a. Purulent drainage from the superficial incision.
   b. Organism(s) identified from an aseptically obtained specimen from the superficial incision or subcutaneous tissue by a culture or non-culture based microbiologic testing method which is performed for purposes of clinical diagnosis or treatment (for example, not Active Surveillance Culture/Testing (ASC/AST)).
   c. Superficial incision that is deliberately opened by a surgeon, physician* or physician designee and culture or non-culture-based testing of the superficial incision or subcutaneous tissue is not performed. A culture or non-culture-based test from the deep soft tissues of the incision that has a negative finding does not meet this criterion.

      AND

      Patient has at least one of the following signs or symptoms: localized pain or tenderness, localized swelling, erythema or heat.
   d. Diagnosis of a superficial incisional SSI by a physician* or physician designee.

* The term physician for the purpose of application of the NHSN SSI criteria may be interpreted to mean a surgeon, infectious disease physician, emergency physician, another physician on the case, or physician's designee (nurse practitioner or physician's assistant).

The following do not qualify as criteria for meeting the definition of superficial incisional SSI

- Diagnosis/treatment of cellulitis (redness/warmth/swelling), by itself, does not meet superficial incisional SSI criterion 'd'.
- A stitch abscess alone (minimal inflammation and discharge confined to the points of suture penetration).
- A localized stab wound or pin site infection. Note: A laparoscopic trocar site is considered a surgical incision and not a stab wound. If a surgeon uses a laparoscopic trocar site to place a drain at the end of a procedure this is considered a surgical incision.

4.4.2 Deep incisional SSI

Surgical site infections involving deep soft tissues of the incision (for example, fascial and muscle layers) belong to this category.

1. **First symptoms occur within 30 or 90# days after the operation**

   AND

2. **Infection involves deep soft tissues of the incision (for example, fascial and muscle layers)**

   AND

3. **Patient has at least ONE of the following**:

   a. Purulent drainage from the deep incision.

   b. A deep incision that spontaneously dehisces, or is deliberately opened or aspirated by a surgeon, physician* or physician designee

      AND

      Organism(s) identified from the deep soft tissues of the incision by a culture or non-culture based microbiologic testing method which is performed for purposes of clinical diagnosis or treatment (for example, not Active Surveillance Culture/Testing (ASC/AST)) or culture or non-culture based microbiologic testing method is not performed. A culture or non-culture-based test from the deep soft tissues of the incision that has a negative finding does not meet this criterion.

      AND

      Patient has at least one of the following signs or symptoms: Temperature instability, fever (> 38 °C) or hypothermia (< 36.5 °C); localized pain or tenderness.

   c. An abscess or other evidence of infection involving the deep incision that is detected on gross anatomical or histopathologic exam, or imaging test.

# Follow-up of 90 days applies when an implant was left in place.

* The term physician for the purpose of application of the NHSN SSI criteria may be interpreted to mean a surgeon, infectious disease physician, emergency physician, another physician on the case, or physician’s designee (nurse practitioner or physician’s assistant).

4.4.3 Organ/space SSI

Surgical site infections involving any part of the body deeper than the fascial/muscle layers that is opened or manipulated during the operative procedure belong to this category.

1. **First symptoms occur within 30 or 90[#] days after the operation**

   AND

2. **Infection involves any part of the body deeper than the fascial/muscle layers that is opened or manipulated during the operative procedure**

   AND

3. **Patient has at least ONE of the following**:

   a. Purulent drainage from a drain that is placed into the organ/space (for example, closed suction drainage system, open drain, T-tube drain, CT-guided drainage).

   b. Organism(s) identified from fluid or tissue in the organ/space by a culture or non-culture based microbiologic testing method which is performed for purposes of clinical diagnosis or treatment (for example, not Active Surveillance Culture/Testing (ASC/AST)).

   c. An abscess or other evidence of infection involving the organ/space that is detected on gross anatomical or histopathologic exam, or imaging test evidence suggestive of infection.

[#] Follow-up of 90 days applies when an implant was left in place.

5 DATA DICTIONARY

The following data items appear in the NeoIPC documentation sheets or in the reporting platform. Some of them (e.g., patient id and patient name) are used for your local documentation only and not used in the NeoIPC software tools.

5.1 PATIENT MASTER DATA

5.1.1 Enrolment

ENROLLING ORGANIZATION UNIT: The unit you want to register the new patient.

NEOIPC-ID (NEOIPC PATIENT IDENTIFIER)*:* The unique id you assign to the patient for tracking in the reporting platform. We advise you to store this information on a pseudonymisation list.

> **Note:** Use this identifier to uniquely identify a patient in the system. Ideally use a unique random string of characters. If you have a requirement to identify a patient you have entered here, you can use this identifier as the NeoIPC key for pseudonymisation. NEVER use an identifier that is used anywhere else and that you do not fully control (e.g. do NOT use the patient id from your hospital information system).

PATIENT-ID: The unique id of the patient in the hospital, which most of the time comes from the hospital information system (HIS) or the patient data management system (PDMS).

PATIENT NAME: Given name followed by family name.

GESTATIONAL AGE (GA): The gestational age, expressed in completed weeks and days (e.g., 25 weeks and 4 days: 25+4) at the time of birth. Typically this refers to the gestational age as it was calculated or estimated by the mother's obstetrician but where this is not available (e.g. in unobserved pregnancies) the gestational age assesed by the treating physician (e.g. via the Ballard score) may be recorded.

BIRTHWEIGHT (BW): The infant's weight immediately after birth in grams. Typically this value is based on a measurement but in case the birth weigth is unknown or has a highly pathological value that does not reflect the maturity of the infant (e.g. hydrops fetalis) you can enter the birth weight that is estimated by the treating physician.

SEX: Typically the phenotypic sex of the patient. If sex cannot be determined from the patient's phenotype or genotype, or if the genotype is neither XX nor XY, it is considered undetermined for purposes of surveillance.

- Female
- Male
- Undetermined

DELIVERY MODE: The mode of the infant's delivery. This can be one of the following:

- Vaginal (including assisted vaginal delivery)
- Elective caesarean section
- Emergency caesarean section

MULTIPLE BIRTH: Check this field if the infant is part of a multiple birth.

NUMBER OF INFANTS AT BIRTH: The number of infants delivered from the (multiple) pregnancy this infant belongs to. Please report the total number of infants including the one you are recording here.

5.1.2 Admission information

ADMISSION DATE: The day the patient is admitted to the hospital, in DD/MM/YYYY format. For inborn infants the same as the date of birth.

ADMISSION TYPE: Describes if the infant was born in your hospital or if it was admitted after birth and if so, how long after birth:

- Admitted from delivery room (initial admission for infants delivered in your hospital)
- Transferred/readmitted to your hospital on the day of birth
- Transferred/readmitted to your hospital the day after birth or later

ADMISSION ON DAY OF LIFE: For infants that have not been delivered in your own hospital, record the infant's day of life on the day of admission (Day of birth= Day of Life 1. The next day, starting at 00:00, is the second day of life.)

5.1.3 Surveillance end

SURVEILLANCE END DATE: The day the data collection and follow-up for the patient has been stopped in DD/MM/YYYY format.

REASON: The reason for ending data collection. One of the following:

- Discharge or transfer
- Death.

PATIENT DAYS: The cumulative number of days the patient stayed in the department, including the day of admission and the day of discharge/transfer/death (no minimum duration of stay)

CVC DAYS: The cumulative number of days when a central venous catheter was in place for at least 12 hours/day.

PVC DAYS: The cumulative number of days when a peripheral vascular catheter was in place for at least 12 hours/day.

INV DAYS: The cumulative number of days when the infant was on invasive ventilation (intubated) for at least 12 hours/day.

NIV DAYS: The cumulative number of days when the infant was on non-invasive ventilation (not intubated; e.g. high flow nasal cannulae or CPAP) for at least 12 hours/day.

HUMAN MILK DAYS: The cumulative number of days the patient’s enteral feeding exclusively consists of (own mother’s or donor) breast milk. Fortified breast milk is considered as breast milk.

KANGAROO-CARE (KC) DAYS: The cumulative number of days the patient received kangaroo care (intensive skin-to-skin-contact) for at least 2 hours.

PROBIOTIC DAYS: The cumulative number of days the patient receives an oral probiotic containing at least one of Lactobacillus spp. or Bifidobacterium spp., regardless of the amount.

Antibiotic Days, total: The cumulative number of days[2] when the infant received (any) systemic antibiotics. Only one antibiotic day can be recorded per day, which means that a day when the patient received multiple antibiotics is still counted as one antibiotic day.

Antibiotic Days, per substance 1,2,3….: The cumulative number of days[2] when the infant received a specified systemic antibiotic substance. *Exp.: Gentamicin days*

5.2 Surgical procedure

Procedure Date: The day of the surgical procedure in *DD/MM/YYYY* format.

Procedure description: A human-readable name or description of the surgical procedure as it is typically called by surgeons in your institution (e.g.; ligation of patent arterial duct).

Main procedure code (ICHI): The International Classification of Health Interventions (ICHI) code of the main procedure performed. If multiple different procedures are performed during one surgery, the surgeon decides which one is the main procedure (typically the most complex procedure or the on causing the highest risk for infection). Please visit https://icd.who.int/dev11/l-ichi/en

Side procedure code (ICHI): The International Classification of Health Interventions (ICHI) code of the side procedure(s) performed. It is possible to capture up to two side procedures.

Duration: The duration of the surgical procedure in minutes (incision-to-suture time if available).

Wound Class: An assessment of the degree of contamination of a surgical wound at the time of the surgical procedure according to the CDC Guidelines. It is assigned by a person involved in the surgical procedure (for example, surgeon, circulating nurse, etc.). The four wound classifications are:

- **Class I/Clean:** An uninfected operative wound in which no inflammation is encountered, and the respiratory, alimentary, genital, or uninfected urinary tract is not entered. In addition, clean wounds are primarily closed and, if necessary, drained with closed drainage. Operative incisional wounds that follow no penetrating (blunt) trauma should be included in this category if they meet the criteria.
- **Class II/Clean-Contaminated:** An operative wound in which the respiratory, alimentary, genital, or urinary tracts are entered under controlled conditions and without unusual contamination. Specifically, operations involving the biliary tract, appendix, vagina, and oropharynx are included in this category, provided no evidence of infection or major break in a sterile technique is encountered.
- **Class III/Contaminated:** Open, fresh, accidental wounds. In addition, operations with major breaks in a sterile technique (eg, open cardiac massage) or gross spillage from the gastrointestinal tract, and incisions in which acute or no purulent inflammation is encountered are included in this category.
- **Class IV/Dirty-Infected:** Old traumatic wounds with retained devitalized tissue and those that involve existing clinical infection or perforated viscera. This definition suggests that the organisms causing postoperative infection were present in the operative field before the operation

[2] The day of the first dose and the day of the last dose of an antibiotic therapy as well as all the days in between are counted. Days where no doses were applied between the first and the last dose (e.g., skipped doses because of high drug levels in therapeutic drug monitoring) are counted as if a dose was applied. Days after the last dose are not counted irrespective of the measured or assumed drug level in the patient.

ASA SCORE: The American Society of Anesthesiologists (ASA) Physical Status Classification System to assess and communicate a patient's pre-anesthesia medical co-morbidities:

- ASA I – A normal healthy patient
- ASA II – A patient with mild systemic disease
- ASA III – A patient with severe systemic disease
- ASA IV – A patient with severe systemic disease that is a constant threat to life
- ASA V – A moribund patient who is not expected to survive without the operation

Visit https://www.asahq.org/standards-and-guidelines/statement-on-asa-physical-status-classification-system.

ENDOSCOPIC PROCEDURE:

- **Yes:** The operation was performed entirely endoscopically,
- **No:** The procedure was performed open or endoscopically assisted, or switched to an open technique during an endoscopic procedure.

EMERGENCY PROCEDURE:

- **Yes:** A procedure that is documented per the facility's protocol to be an Emergency or Urgent procedure.
- **No:** The intervention is initiated and performed in a planned manner.
- **Unknown:** No information available.

PRIMARY CLOSURE: The closure of the skin level during the original surgery, regardless of the presence of wires, wicks, drains, or other devices or objects extruding through the incision. This category includes surgeries where the skin is closed by some means. Thus, if any portion of the incision is closed at the skin level, by any manner, a designation of primary closure should be assigned to the surgery.

> **Note**: If a procedure has multiple incision/laparoscopic trocar sites and any of the incisions are closed primarily then the procedure technique is recorded as primary closed.

REVISION PROCEDURE: Revision procedures are follow-up, replacement or corrective procedures after an initial procedure. A revision procedure terminates the follow-up for the primary procedure and starts a new follow-up period.

IMPLANT: An implant is a foreign body of non-human origin that is permanently placed into a patient during an operation and is not routinely manipulated for diagnostic or therapeutic purposes (e.g.; vascular prostheses, screws, wires, meshes).

SIGNS OF INFECTION AT TIME OF SURGERY*:* Complete this field if signs of infection were identified during the surgical procedure. The signs of infection must be noted intraoperatively and documented in the narrative part of the operation note or report of surgery. If a surgical site infection occurs, this information will be used to determine if the signs listed in this section can be interpreted as "Infection present at time of surgery".

Example:

- Examples that indicate evidence of infection include but are not limited to: abscess, infection, purulence/pus, phlegmon, or "feculent peritonitis". A ruptured/perforated appendix is evidence of infection at the organ/space level.

- Examples of verbiage that is not considered evidence of infection include but are not limited to: colon perforation, contamination, necrosis, gangrene, fecal spillage, nicked bowel during procedure, murky fluid, or documentation of inflammation.
- The use of the ending "itis" in an operative note/report of surgery does not automatically count as evidence of infection, as it may only reflect inflammation which is not infectious in nature (for example, diverticulitis, peritonitis, and appendicitis).
- Pathology report findings and imaging test findings cannot be used as evidence of infection.
- Identification of an organism using culture or non-culture based microbiologic testing method or on a pathology report from a surgical specimen cannot be used as evidence of infection.
- Wound class cannot be used as evidence of infection.
- Trauma resulting in a contaminated case does not automatically count as evidence of infection. For example, a fresh gunshot wound to the abdomen may be a trauma with a high wound class but there would not be time for infection to develop.
- Procedural complications such as bowel perforation during surgery cannot be used as evidence of infection since there was no infection present at time of surgery.

5.3 INFECTION DATA

5.3.1 General infection data

INFECTION DATE: The day the first infection symptoms appeared in *DD/MM/YYYY* format. If no symptoms, the day of first positive culture at the primary infection site.

COMMON COMMENSAL: A type of micro-organism (e.g., coagulase-negative staphylococci), that is commonly present on epithelium-covered body surfaces (e.g., skin). When one or multiple of these species grow in a blood culture, a contamination is more likely than when a recognised pathogen grows. See *Master Organism List* on https://neoipc.org/surveillance/resources/

RECOGNISED PATHOGEN: A type of microorganism (e.g., *Escherichia coli*), that is typically recognised as a pathogen when growth is detected in a microbiological culture that is representative for an invasive infection (e.g., blood culture). See *Master Organism List* on https://neoipc.org/surveillance/resources/

MDROS:

Select the one(s) applicable to the isolated organism;

- ☐ **MRSA/VRE/3GCR:** Choose if one of the following applies:
    - **MRSA:** Methicillin-resistant Staphylococcus aureus
    - **VRE:** Vancomycin-resistant Enterococci
    - **3GCR:** Multi drug resistant gram-negative pathogen resistant to 3rd generation Cephalosporins according to own lab's cut-off values (refers to phenotypic resistance or ESBL-producing organisms)

- ☐ **Carbapenem resistant:** Resistant to at least one of the following (Imipenem, Meropenem, Ertapenem) according to own lab's cut-off values (refers to phenotypic resistance or carbapenemase-producing organisms.)
- ☐ **Colistin resistant:** Resistant to colistin according to own lab's cut-off values.

Options for each group:

- ○ **Yes:** the isolated organism is resistant to the specified antibiotic group(s).
- ○ **No:** the isolated organism is not resistant to the specified antibiotic group(s).
- ○ **Not tested:** the isolated organism was not tested for resistance to the specified antibiotic group(s).

SECONDARY BLOODSTREAM INFECTION: A BSI that is thought to be seeded from a site-specific infection at another body site (except for catheter-associated infections). A BSI can be attributed to a site-specific infection (NEC, PN or SSI) if it occurs in a 17-day period that includes the day of infection (=first symptoms of site-specific infection), 3 days prior, and 13 days after.

Collecting data about the secondary BSI is optional; therefore, it is possible to select **"UNKNOWN"** if you are not following patients for secondary BSI. For detailed information, please see Secondary bloodstream infection.

- ○ **Yes:** if you are following patients for secondary BSI and the patient has developed a secondary sepsis that meets the definition of NeoIPC *(activates a field below where you can enter identified organisms)*
- ○ **No:** if you are following patients for secondary BSI and the patient has not developed a secondary sepsis that meets the definition of NeoIPC
- ○ **No follow-up:** if you are not following patients for secondary BSI.

5.3.2 BSI specific data

CVC (CENTRAL VASCULAR CATHETER): An intravascular catheter that terminates at or close to the heart **or** in one of the great vessels which is used for infusion, withdrawal of blood, or hemodynamic monitoring. Examples:

- Central venous catheter (CVC), including non-tunnelled, tunnelled and implanted central venous catheters.
- Peripherally inserted central venous catheter (PICC)
- Umbilical artery catheter (UAC) or umbilical venous catheter (UVC)

Extracorporeal membrane oxygenation (ECMO) and arterial catheters (except pulmonary artery, aorta, or umbilical artery) are NOT considered as CVC. The following are considered great vessels for the purpose of reporting CVCs:

- Aorta
- Pulmonary artery
- Superior vena cava
- Inferior vena cava
- Brachiocephalic veins
- Internal jugular veins
- Subclavian veins
- External iliac veins

- Common iliac veins
- Femoral veins
- Umbilical artery/vein

CVC-ASSOCIATED BSI: A primary bloodstream infection is considered central vascular catheter (CVC)-associated if the CVC has been present for at least three consecutive days on the day of infection (=first symptoms or first positive diagnostic test; e.g. culture) or the day before.

CVC-DAY: A day in which the patient had a CVC placed for at least 12 hours cumulatively.

PVC (PERIPHERAL VASCULAR CATHETER): PVC is a catheter placed into a peripheral vein and does not reach one of the great vessels mentioned under CVC.

PVC-ASSOCIATED BSI: A primary bloodstream infection is considered peripheral vascular catheter (PVC)-associated if the PVC has been present for at least three consecutive days on the day of infection (=first symptoms or first positive diagnostic test; e.g. culture) or the day before and does not meet the criteria for CVC-associated BSI. That means, if a patient developing a bloodstream infection meets the criteria for both, PVC- and CVC-association, the CVC is considered as the device with the relatively higher infection risk and the BSI should be recorded as CVC-associated BSI.

PVC-DAY: A day in which the patient had a PVC placed for at least 12 hours cumulatively.

INTRAVENOUS ANTIBIOTIC THERAPY FOR FIVE OR MORE DAYS INITIATED: Antibiotic treatment for at least five days was initiated. The day of the first dose and the day of the last dose are counted. Days where no dose was administered between the first and the last dose (e.g., skipped doses because of high drug levels in therapeutic drug monitoring) are counted as if a dose had been administered. Days after the last dose are not counted regardless of the patient's measured or assumed drug level. If the infant died, was discharged, or transferred before the end of the five-day course of intravenous antibiotics, this condition is met if treatment was scheduled for five days or more.

NEW/MORE FREQUENT EPISODES OF APNEA OR INCREASES IN OXYGEN DEMAND OR VENTILATORY SUPPORT: New or increased frequency of episodes of apnea lasting more than 20 seconds, or an increase in the amount of oxygen required to maintain adequate oxygenation, or an escalation of ventilatory support, such as increased flow with high-flow therapy or intubation for invasive ventilation.

ENTERAL FEEDING INTOLERANCE, ABDOMINAL DISTENSION OR ILEUS: Enteral feeding intolerance, abdominal distension or ileus without imaging findings or surgical findings that would otherwise suggest a necrotizing entrocolitis or a spontaneous intestinal perforation.

UNEXPLAINED METABOLIC ACIDOSIS: Unexplained metabolic acidosis with a base deficit greater (more negative) than 10 mmol/L (10 mEq/L).

5.3.3 NEC specific data

PORTAL VENOUS GAS (HEPATOBILIARY GAS): Accumulation of gas (-bubbles) in the portal vein and its branches.

5.3.4 Pneumonia specific data

BEGINNING OR INCREASE IN RESPIRATORY SUPPORT:

New initiation of respiratory support or escalation of existing level of respiratory support…:

- □ Increase in need for $FiO_2 \geq 0.25$ (25 points) within 24 hours (daily minimum FiO_2 values must be taken into account here)
  OR

- ☐ begin of non-invasive ventilatory support (excluding switch from invasive ventilation) OR
- ☐ begin of invasive mechanical ventilation (including switch from non-invasive ventilatory support)

….that does not improve within less than 2 days: The above-mentioned condition should not improve within two days.

…after at least 2 days of stability or improvement: A stable or improving baseline period of at least two days is required before the above condition occurs.

Note: To meet the criteria of device associated hospital-acquired pneumonia, patients must be ventilated for at least 4 calendar days (day 1 is the day invasive/non-invasive ventilation starts). The earliest date of the event is day 3 of ventilation.

INV*:* Invasive mechanical ventilation via endotracheal or tracheostomy tube

INV-ASSOCIATED PNEUMONIA: A pneumonia is associated with INV if the patient had an endotracheal or tracheostomy tube for at least 3 consecutive days on the day of infection (=first symptoms or first positive culture) or on the day before.

INV-DAY: A day in which the patient was ventilated invasively via endotracheal or tracheostomy tube for at least 12 hours cumulatively.

NIV: Non-invasive ventilatory support via CPAP or High-Flow Nasal Cannula.

NIV-ASSOCIATED PNEUMONIA: A pneumonia is associated with NIV if the patient received non-invasive ventilatory support (e.g., CPAP or High-Flow Nasal Cannula) for at least 3 consecutive days on the day of infection (=first symptoms or first positive culture) or on the day before.

NIV-DAY: A day in which the patient received non-invasive ventilatory support via CPAP or High-Flow Nasal Cannula for at least 12 hours cumulatively.

ORGANISMS IDENTIFIED FROM RESPIRATORY TRACT: At least one organism has been identified from respiratory tract by a culture or non-culture based microbiologic testing method which is performed for purposes of clinical diagnosis or treatment (for example, NOT Active Surveillance Culture/Testing (ASC/AST))

- ☐ **Recovered from lower respiratory tract:** Select if fungal, bacterial or viral pathogens (viral gene, antigen or antibody via e.g. EIA, FAMA, shell vial assay, PCR) are detected in lower respiratory tract
- ☐ **Recovered from upper respiratory tract:** Select only if viral pathogens are detected in upper respiratory tract

5.3.5 SSI specific data

INFECTION PRESENT AT TIME OF SURGERY: Select YES, only if the sign of infection identified during the surgical procedure applies to the depth of the SSI that is being attributed to the procedure.

EXAMPLE:

- If a patient has documentation of an intra-abdominal infection at time of surgery and then later returns with an organ/space SSI = YES.
- If a patient has documentation of an intra-abdominal infection at time of surgery and then later returns with a superficial or deep incisional SSI = NO.

SSI TYPE:

- **A superficial incisional SSI** involves only skin and subcutaneous tissue of the incision and first symptoms occur within 30 days after the operation.
- **A deep incisional SSI** involves deep soft tissues of the incision (for example, fascial and muscle layers) and first symptoms occur within 30 days after the operation or 90 days after the operation when an implant was left in place.
- **An organ/space SSI** involves any part of the body deeper than the fascial/muscle layers that is opened or manipulated during the operative procedure and first symptoms occur within 30 days after the operation or 90 days after the operation when an implant was left in place.

Note: The SSI reported must reflect the deepest tissue level where SSI criteria are met during the surveillance period.

Example:

- If an SSI started as a deep incisional SSI on day 10 of the SSI surveillance period and then a week later (Day 17) meets criteria for an organ/space SSI. You must report it as organ/space SSI regardless of superficial or deep tissue involvement. The day of infection in this case would be "Day 17".

ORGANISM(S) IDENTIFIED FROM SURGICAL SITE: At least one organism has been identified from surgical site by a culture or non-culture based microbiologic testing method which is performed for purposes of clinical diagnosis or treatment (for example, NOT Active Surveillance Culture/Testing (ASC/AST)).

6 DATA ANALYSIS

The following rates are calculated both for the departments' reports and for the reference reports and can serve as starting point for further analyses and discussions in your department.

For the core module, the rates are generally stratified into 4 groups according to the birth weight of the infants:

1. < 500 g
2. 500 g – 999 g
3. 1000 g – 1499 g
4. >1500 g

6.1 RISK FACTORS AND PROTECTIVE FACTORS

6.1.1 Device utilization

A device utilization rate describes the percentage of patient days on which a specific device was used. It is calculated by dividing the number of device days by the number of patient days and multiplying the result by 100.

$$CVC\ Utilization\ Rate = \frac{\text{Total CVC Days}}{\text{Total Patient Days}}\ x\ 100$$

$$PVC\ Utilization\ Rate = \frac{\text{Total PVC Days}}{\text{Total Patient Days}}\ x\ 100$$

$$INV\ Utilization\ Rate = \frac{\text{Total INV Days}}{\text{Total Patient Days}}\ x\ 100$$

$$NIV\ Utilization\ Rate = \frac{\text{Total NIV Days}}{\text{Total Patient Days}}\ x\ 100$$

6.1.2 Antibiotic use

Antibiotic use rate describes the percentage of patient days on which systemic antibiotics were used in relation to the total patient days. It is calculated by dividing the number of antibiotic days by the number of patient days and multiplying the result by 100.

$$Antibiotic\ Use\ Rate = \frac{\text{Total Antibiotic Days}}{\text{Total Patient Days}}\ x\ 100$$

In addition to the overall antibiotic use rate, antibiotic use rates and proportions of patients receiving a substance are calculated for individual substances and groups of substances.

The substances and groups are derived from the Anatomical Therapeutic Chemical (ATC) Classification (https://www.who.int/tools/atc-ddd-toolkit/atc-classification) as published in the most recent ATC/DDD Index (https://www.whocc.no/atc_ddd_index/).

Currently the ATC 1st, 2nd, 4th and 5th level are used for grouping substances.

Since the numbers in the individual groups usually get very small, the substance and group specific use rates are calculated by dividing the number of substance (group) days by the number of patient days and multiplying the result by 1000.

$$Substance\ Group\ Use\ Rate = \frac{\text{Total Therapy Days for Substance Group}}{\text{Total Patient Days}}\ x\ 1000$$

The proportion of patients receiving any specific substance(s) of a substance group is calculated as ratio between the total number of patients receiving a specific substance or group of antibiotics and the total number of patients receiving any kind of antibiotic multiplied by 100.

$$Proportion\ of\ Patients\ receiving\ any\ AB\ substance = \frac{\text{Number of Patients Substance}}{\text{Number of Patients Total}}\ x\ 100$$

6.1.3 Protective factor implementation

A protective factor utilization rate describes the percentage of patient days on which a patient received a specific protective factor, such as breast milk, probiotic or kangaroo mother care. It is calculated by dividing the number of protective factor days by the number of patient days and multiplying the result by 100.

$$Breast\ Milk\ Intake\ Rate = \frac{\text{Total Breast Milk Days}}{\text{Total Patient Days}}\ x\ 100$$

$$Probiotic\ Usage\ Rate = \frac{\text{Total Probiotic Days}}{\text{Total Patient Days}}\ x\ 100$$

$$KMC\ Implementation\ Rate = \frac{\text{Total KMC Days}}{\text{Total Patient Days}}\ x\ 100$$

6.2 INFECTIONS

6.2.1 Incidence densities

Since a substantial proportion of infections cannot be linked to a specific risk factor, incidence densities are calculated for bloodstream infections, pneumonia, NEC. The risk factor here represents the cumulative number of patient days and is standardized as 1000 patient days.

$$BSI\ incidence\ density = \frac{\text{Total BSI cases}}{\text{Total patient days}}\ x\ 1000$$

$$Pneumonia\ incidence\ density = \frac{\text{Total Pneumonia cases}}{\text{Total patient days}}\ x\ 1000$$

$$NEC\ incidence\ density = \frac{\text{Total NEC cases}}{\text{Total patient days}}\ x\ 1000$$

6.2.2 Device-associated infections

A device-associated infection rate is an important quality management metric and describes how many device-associated infections occur per 1000 device days. In this process called standardization, infections (e.g., BSI) occurring in the presence of a certain risk factor (e.g., CVC) are associated with the total number of days at risk (e.g., CVC Days) and the result is multiplied by 1000.

$$CVC\ associated\ BSI\ Rate = \frac{\text{Total BSI cases in patients with CVC}}{\text{Total CVC Days}}\ x\ 1000$$

$$PVC\ associated\ BSI\ Rate = \frac{\text{Total BSI cases in patients with PVC}}{\text{Total PVC Days}}\ x\ 1000$$

$$INV\ associated\ \ Pneumonia\ Rate = \frac{\text{Total pneumonia cases in patients with INV}}{\text{Total INV days}}\ x\ 1000$$

$$NIV\ associated\ \ Pneumonia\ Rate = \frac{\text{Total pneumonia cases in patients with NIV}}{\text{Total NIV days}}\ x\ 1000$$

6.2.3 Surgical site infections

Surgical site infection (SSI) rates define the percentage of SSI that occur during the observation period after an operative procedure. It is calculated by dividing the number of SSIs occurring after a surgical procedure by the number of surgical procedures and multiplying the result by 100. Since the risk of developing a SSI depends on the type of surgical procedure, multiple SSI rates are calculated for groups of similar procedures. Nevertheless, an overall SSI rate will be calculated to account for the fact that surgery in VLBW/VPT infants is less frequent than in adults and grouping procedures may result in very low procedure counts per group.

$$\text{Overall SSI rate} = \frac{\text{Total SSI cases}}{\text{Total number of Surgeries}}\ x\ 100$$

$$\text{Grouped SSI rate} = \frac{\text{Total SSI cases observed after the procedures in this group}}{\text{Total number of procedures in this group}}\ x\ 100$$

6.2.4 Standardized infection rate

With the infection rates described above, it is possible to observe risk factors and infections for the 3 birthweight classes in detail. However, 500 g birth weight is a relatively crude stratification for neonatal infection risk, and it also does not account the fact that infants may be transferred to other departments (e.g., for surgery or to a department closer to the parents' home) long before they would normally be discharged and that the high-risk days of the stabilization phase would accumulate in some neonatology departments while others would accumulate the low risk days of already stabilized infants. To account for this, NeoIPC calculates a standardized infection rate (SIR) , which compares infection frequencies between departments based on their patient population composition

Standardized infection rates are calculated based on two factors:

1. Risk of infection for a certain birth weight
2. Risk of infection for a certain day of life

From the reference database, the average risk of BSI or pneumonia for a certain day of life of an infant with a certain birth weight is calculated.

After this, the number of expected infections (cumulated risk) for a department is calculated by summing the average risks of the infants and the respective days they spent in the department.

The SIR represents the ratio of observed infections to expected infections in a department and can give a general overview of the infection risk in a department.

$$Standardized\ infection\ rate = \frac{\text{Total infections observed}}{\text{Total infections expected}}$$

When the standardized infection rate is greater than one, you observed more infections than you would expect from your patient composition when compared to the reference data.

When the standardized infection rate equals one, you observed the same number of infections as you would expect from your patient composition when compared to the reference data.

When the standardized infection rate is less than one, you observed less infections than you would expect from your patient composition when compared to the reference data.

7 ABBREVIATIONS

**3GCR:**	Multi drug resistant gram-negative pathogen resistant to 3rd generation Cephalosporins
**AB**	Antibiotic
**ASA**	American Society of Anaesthesiologists
**BE**	Base Excess
**BSI**	Bloodstream Infection
**BW**	Birthweight
**CDC**	Centers for Disease Control and Prevention
**CMV**	Cytomegalovirus
**CRP**	C-reactive Protein
**CSF**	Cerebrospinal Fluid
**CT**	Computer Tomography
**CVC**	Central Venous Catheter
**ECMO**	Extracorporeal Membrane Oxygenation
**ESBL**	Extended spectrum beta-lactamase
**GA**	Gestational Age
**HIS**	Hospital Information System
**HIV**	Human Immunodeficiency Virus
**ICHI**	International classification of health interventions
**INV**	Invasive Ventilation
**IPC**	Infection Prevention and Control
**KC**	Kangaroo care
**LCBSI**	Laboratory Confirmed Bloodstream Infection
**MDRO**	Multidrug Resistant Organism
**MRSA**	Methicillin-resistant Staphylococcus aureus
**NEC**	Necrotizing Enterocolitis
**NICU**	Neonatal intensive care unit
**NIV**	Non-invasive Ventilation
**OP**	Operative Procedure
**PDMS**	Patient Data Management System
**PICC**	Peripherally Inserted Central Venous Catheter
**PLT**	Platelet
**PVC**	Peripheral Venous Catheter
**RCT**	Randomised Controlled Trial
**SSI**	Surgical Site Infection
**UAC/UVC**	Umbilical Artery Catheter/ Umbilical Venous Catheter
**VAE**	Ventilator Associated Event
**VAP**	Ventilator Associated Pneumonia
**VLBW**	Very Low Birthweight
**VPT**	Very Preterm
**VRE**	Vancomycin-resistant Enterococcus
**WBC**	White Blood Cells

8 IMPRINT

NeoIPC Project

Fondazione Penta Onlus
Corso Stati Uniti, 4
35127 Padova
ITALY

https://neoipc.org/

To know more about NeoIPC, write to:

communication@pentafoundation.org

To know more about the Trial (NeoDeco) and the Clinical Practice Network, write to:

neoipc@sgul.ac.uk

To know more about Surveillance, write to:

neoipc-support@charite.de

This project has received funding from the European Union's Horizon 2020 research and innovation programme under grant agreement No. 965328.
