# Supplementary material for: Methodological framework for the surveillance of healthcare-associated infections in high-risk infants: the NeoIPC surveillance core module protocol
Source: Antimicrob Resist Infect Control. 2026 Feb 19;15:30. doi: 10.1186/s13756-026-01711-0 (PMC12930787; doi:10.1186/s13756-026-01711-0)
Supplement: Supplementary file 2 — Additional File 2. LMIC evaluation survey [file 13756_2026_1711_MOESM2_ESM.pdf]

NeoIPC Surveillance Evaluation Survey

This survey aims to understand how the NeoIPC surveillance approach fits into your local context. Please answer based on your current experience with NeoIPC surveillance program at your neonatal unit.

1. Site Specifications

a) Country: Freetext

b) Hospital: Freetext

c) Number of very low birth weight (VLBW: Birthweight<1500g) infants are admitted to your unit per year (estimated):

- ○ 1-25
- ○ 26-50
- ○ 51-100
- ○ 101-250
- ○ 250-500
- ○ >500
- ○ Not sure/ unknown

d) Do you have a (pediatric) surgery department that routinely performs surgery in neonates?

- ○ Yes
- ○ No

2. Current Surveillance Practices

a) Apart from NeoIPC, do you currently perform surveillance for hospital-acquired (nosocomial) infections in neonatology?

- ○ Yes
- ○ No

i. If YES:
How effective do you consider your current surveillance program for infection prevention and control (IPC)?

- ◯ Sufficient
- ◯ Insufficient
- ◯ Not sure

ii. If NO:
How effective do you consider your current IPC practices (without formal surveillance)?

- ◯ Sufficient
- ◯ Insufficient
- ◯ Not sure

3. Current Surveillance Practices

a) Currently you are:

- ◯ using NeoIPC surveillance as part of routine/regular practice
- ◯ using NeoIPC surveillance in a pilot or limited capacity

4. Comparing NeoIPC Surveillance to Your Current Practices

a) What are the advantages of NeoIPC surveillance compared to your current surveillance system or practices? *(Select all that apply)*

- ☐ Clearer case definitions
- ☐ Better data quality
- ☐ Easier to use in daily practice
- ☐ Improved comparability with other sites
- ☐ Less time-consuming
- ☐ More useful for IPC decision-making
- ☐ Good training and support
- ☐ Other [Free text]

b) What are the disadvantages of NeoIPC surveillance compared to your current surveillance system or practices? *(Select all that apply)*

- ☐ Too time-consuming
- ☐ Too limited in scope
- ☐ Difficult to integrate into routine workflow
- ☐ Definitions are unclear or difficult to apply
- ☐ Lack of automation or digital tools

☐ Limited feedback or benchmarking

☐ Other [Free Text]

5. Adapting NeoIPC Surveillance to Your Setting

a) What changes or adaptations would you make to NeoIPC surveillance so it would work effectively in your setting?

[Free text]

6. Integration into Your Current Practices

a) How would NeoIPC surveillance fit into your current work processes?

◉ It would replace our current surveillance system and/or practices

○ It would complement our current surveillance system and/or practices

○ Not sure

b) What challenges do you expect when implementing NeoIPC surveillance?

[Free text]

c) How might you address or overcome these challenges?

[Free text]

7. Infrastructure and Organizational Factors

a) What aspects of your hospital infrastructure and/or organizational structure would help you implement NeoIPC surveillance?

[Free text]

b) What aspects might make it difficult to implement NeoIPC surveillance?

[Free text]

c) What kind of infrastructure or system changes would be needed? *(Select all that apply)*

☐ Addition of extra human resources

☐ Changes in staff duties and/or work flow

☐ Changes in hospital policies or approvals

☐ Additional internet and/or computer access

☐ Changes in IT systems or electronic medical records

☐ Other [Free Text]

8. Resources for Implementation

a) Do you expect to have enough resources to implement and maintain NeoIPC surveillance?

◯ Yes

◯ No

◯ Not sure

i. If YES:
What resources do you already have or expect to use? Are there others you would need?

[Free text]

ii. If NO:
What resources are missing? What challenges do you expect?

[Free text]

iii. If NOT SURE:
Please explain:

[Free text]

9. Overall Assessment

a) Based on your experience and impressions so far, how likely would you be to adopt NeoIPC as the default surveillance system to support IPC in your neonatal unit? *(Please rate on a scale of 0 to 10, 0 = Not at all likely, 10 = Very likely)*

0 1 2 3 4 5 6 7 8 9 10

Not at all likely Very likely

b) Please add any comments or explanations if needed *(optional)*:

[Free Text]
