# Supplementary material for: Methodological framework for the surveillance of healthcare-associated infections in high-risk infants: the NeoIPC surveillance core module protocol
Source: Antimicrob Resist Infect Control. 2026 Feb 19;15:30. doi: 10.1186/s13756-026-01711-0 (PMC12930787; doi:10.1186/s13756-026-01711-0)
Supplement: Supplementary file 3 — Additional File 3. Data collection sheets [file 13756_2026_1711_MOESM3_ESM.pdf]

NeoIPC

NeoIPC – Core Module (VLBW/VPT Infants)

Pseudonymization Table

#	NeoIPC-ID	Patient-ID	Patient Name	Date of Birth	Comments

Page: ________

NeoIPC – Core Module (VLBW/VPT Infants)

Patient Progress Chart

NeoIPC

Patient ID: | **Patient name:**

Month/Year: | **Chart no:**

Days	1	2	3	4	5	6	7	8	9	10	11	12	13	14	15	16	17	18	19	20	21	22	23	24	25	26	27	28	29	30	31	T
**Patient days**																																
**CVC days**																																
**PVC days**																																
**INV days**																																
**NIV days**																																
**Human milk days**																																
**Kangaroo care days**																																
**Probiotic days**																																
**Antibiotic days (total):**																																
**AB-1**																																
**AB-2**																																
**AB-3**																																
**AB-4**																																
**AB-5**																																
**AB-6**																																

Comments:

NeoIPC – Core Module (VLBW/VPT Infants)

Master Data Collection Sheet

Enrolment

Patient ID:

Patient name:

Gestational age: (weeks + days, e.g. 25+4)

Birthweight: grams

Sex:

- ○ Female
- ○ Male
- ○ Undetermined

Delivery mode:

- ○ Vaginal
- ○ Caesarean section (elective)
- ○ Caesarean section (emergency)

Multiple birth:

- ○ Yes, total number of infants at birth: ______________
- ○ No

Admission Information

Admission date:

Admission type:

- ○ Admitted from delivery room (initial admission for infants delivered in your hospital)
- ○ Transferred/readmitted to your hospital on the day of birth
- ○ Transferred/readmitted to your hospital the day after birth or later **(Admission on day of life: __________)**

Surveillance End

Surveillance end date:

Reason:

- ○ Discharge / Transfer
- ○ Death

Patient days:

CVC days:

PVC days:

INV days:

NIV days:

Human milk days:

Kangaroo care days:

Probiotic days:

Antibiotic days (total):

Antibiotic days (per substance)

Antibiotic substance 1: ______________________________: __________ days

Antibiotic substance 2: ______________________________: __________ days

Antibiotic substance 3: ______________________________: __________ days

Antibiotic substance 4: ______________________________: __________ days

Antibiotic substance 5: ______________________________: __________ days

Antibiotic substance 6: ______________________________: __________ days

Comments:

For more information, please see sections *5. Data Dictionary* and *7. Abbreviations* in the NeoIPC - Core Module Protocol.

○ *You can select only one option.*

☐ *You can select multiple options.*

NeoIPC – Core Module (VLBW/VPT Infants)

Surgical Procedure Data Collection Sheet

Patient			
**Patient ID:**			
**Patient name:**			
Surgical Procedure			
**Procedure date:**			
**Procedure description:**			
**Duration (minutes):**			
**Main procedure code (ICHI[1]):**			
**Side procedure code (ICHI[1]):**			
**Side procedure code (ICHI[1]):**			
**ASA-Score[2]:**<br>○ ASA I – A normal healthy patient<br>○ ASA II – A patient with mild systemic disease<br>○ ASA III – A patient with severe systemic disease<br>○ ASA IV – A patient with severe systemic disease that is a constant threat to life<br>○ ASA V – A moribund patient who is not expected to survive without the operation			
**Wound class:**<br>○ Clean<br>○ Clean-contaminated<br>○ Contaminated<br>○ Dirty-infected			
**Endoscopic procedure:**	○ Yes	○ No	
**Emergency procedure:**	○ Yes	○ No	○ Unknown
**Primary closure:**	○ Yes	○ No	
**Revision procedure:**	○ Yes	○ No	
**Implant:**	○ Yes	○ No	
**Signs of infection at time of surgery:**			

○ *You can select only one option.*
☐ *You can select multiple options.*

[1] ***https://www.who.int/standards/classifications/international-classification-of-health-interventions***
[2] Classification of the American Society of Anesthesiologists
For more information, please see sections *5. Data Dictionary* and *7. Abbreviations* in the NeoIPC - Core Module Protocol.

NeoIPC – Infection Data Collection Sheet

Hospital-Acquired Primary Sepsis/BSI

Patient
**Patient ID:**
**Patient name:**
Hospital-acquired Primary Sepsis/BSI
**Infection date:**
**Vascular catheter association:**<br>○ No ○ CVC-associated ○ PVC-associated
**BSI type:**<br>○ Clinical Sepsis *(no positive blood/cerebrospinal fluid culture)*<br>○ LCBSI-RP *(caused by a recognised pathogen)*<br>○ LCBSI-CC *(caused by a common commensal), recovered*: ○ only once or ○ at least twice
**Intravenous antibiotic therapy for five or more days initiated:**<br>○ Yes<br>○ No
**Please enter organism(s) recovered, if you chose LCBSI-RP or LCBSI-CC:**<br>**Organism 1:** ______________________________ , recovered from □ Blood □ CSF<br>▪ MRSA/VRE/3GCR[1] ○ Yes ○ No ○ Not tested<br>▪ Carbapenem resistant ○ Yes ○ No ○ Not tested<br>▪ Colistin resistant ○ Yes ○ No ○ Not tested<br>**Organism 2:** ______________________________ , recovered from □ Blood □ CSF<br>▪ MRSA/VRE/3GCR[1] ○ Yes ○ No ○ Not tested<br>▪ Carbapenem resistant ○ Yes ○ No ○ Not tested<br>▪ Colistin resistant ○ Yes ○ No ○ Not tested<br>**Organism 3:** ______________________________ , recovered from □ Blood □ CSF<br>▪ MRSA/VRE/3GCR[1] ○ Yes ○ No ○ Not tested<br>▪ Carbapenem resistant ○ Yes ○ No ○ Not tested<br>▪ Colistin resistant ○ Yes ○ No ○ Not tested
**Signs and symptoms of generalized infection:**<br>□ Temperature instability or fever (>38 °C) or hypothermia (<36.5 °C)<br>□ Unexplained tachycardia (>200/min) or new/more frequent bradycardia episodes (<80/min)<br>□ Capillary refill time of > 3s or skin mottling or core/peripheral temperature gap > 2 °C<br>□ New/more frequent episodes of apnoea (>20s) or increase in oxygen demand or ventilatory support<br>□ Enteral feeding intolerance, abdominal distension or ileus<br>□ Irritability, lethargy, apathy or unstable condition<br>□ Unexplained metabolic acidosis (base excess < −10 mmol/L; <−10 mEq/L)<br>□ New and unexplained hyperglycaemia (> 140 mg/dl; > 7.8 mmol/L) or hypoglycaemia (< 40 mg/dl; <2.2 mmol/L)
**Laboratory findings:**<br>□ Platelet count of < 100 × 10^9/L (<100 × 10^3/µL)<br>□ WBC < 4 × 10^9/L or > 20 × 10^9/L (< 4 × 10^3/µL or > 20 × 10^3/µL)<br>□ CRP > 10 mg/L (> 1 mg/dL)<br>□ Procalcitonin ≥ 2µg/L (2 ng/mL; 200 ng/dL)<br>□ I/T-Ratio > 0,2 (ratio of immature granulocytes to total granulocytes)<br>□ Increased levels of interleukin 6 (IL-6) or IL-8

○ *You can select only one option.*
□ *You can select multiple options.*

[1] Please mark the antibiotic resistance profile appropriate to the isolated microorganism and answer accordingly.
For more information, please see sections *5. Data Dictionary* and *7. Abbreviations* in the NeoIPC - Core Module Protocol.

NeoIPC – Infection Data Collection Form

Necrotizing Enterocolitis (NEC)

Patient	
**Patient ID:**	
**Patient name:**	
**Necrotizing Enterocolitis**	
**Infection date:**	
**Radiological signs (imaging technologies: X-ray, CT, MRI, ultrasound):**<br>□ Pneumoperitoneum<br>□ Pneumatosis intestinalis<br>□ Portal venous gas (Hepatobiliary gas)<br>□ Fixed bowel loops (≥ 24h)	
**Clinical signs and symptoms:**<br>□ Abdominal distention<br>□ Abdominal discoloration or shiny/reddish skin tone<br>□ Repeated occult (guaiac test) or visible blood in stool (no anal fissure)	□ Increasing/pronounced vomiting<br>□ Increased gastric residuals from previous feeding<br>□ Bilious gastric aspirate (not from transpyloric feeding tube)
**Surgical or pathological findings:**<br>□ Extensive bowel necrosis (> 2 cm of bowel affected)<br>□ Pneumatosis intestinalis	□ Intestinal perforation (not a definition criterion)

Secondary BSI:

○ Yes ○ No ○ No follow-up

If you chose YES, please enter organism(s) recovered from blood culture:

Organism 1: ______________________________

- MRSA/VRE/3GCR[1] ○ Yes ○ No ○ Not tested
- Carbapenem resistant ○ Yes ○ No ○ Not tested
- Colistin resistant ○ Yes ○ No ○ Not tested

Organism 2: ______________________________

- MRSA/VRE/3GCR[1] ○ Yes ○ No ○ Not tested
- Carbapenem resistant ○ Yes ○ No ○ Not tested
- Colistin resistant ○ Yes ○ No ○ Not tested

Organism 3: ______________________________

- MRSA/VRE/3GCR[1] ○ Yes ○ No ○ Not tested
- Carbapenem resistant ○ Yes ○ No ○ Not tested
- Colistin resistant ○ Yes ○ No ○ Not tested

○ *You can select only one option.*
□ *You can select multiple options.*

[1] Please mark the antibiotic resistance profile appropriate to the isolated microorganism and answer accordingly.
For more information, please see sections 5. Data Dictionary and 7. Abbreviations in the NeoIPC - Core Module Protocol.

NeoIPC – Infection Data Collection Sheet

Pneumonia (PN)

Patient
**Patient ID:**
**Patient name:**
Hospital-acquired Pneumonia
**Infection date:**
**Device association:**<br>○ No ○ INV-associated ○ NIV-associated
**Organisms identified from respiratory tract (RT):**<br>○ Yes ○ No ○ Not tested<br>**If you chose YES, please enter the organism(s):**<br>**Organism 1:** ______________________, recovered from □ lower RT □ upper RT<br>▪ MRSA/VRE/3GCR[1] ○ Yes ○ No ○ Not tested<br>▪ Carbapenem resistant ○ Yes ○ No ○ Not tested<br>▪ Colistin resistant ○ Yes ○ No ○ Not tested<br>**Organism 2:** ______________________, recovered from □ lower RT □ upper RT<br>▪ MRSA/VRE/3GCR[1] ○ Yes ○ No ○ Not tested<br>▪ Carbapenem resistant ○ Yes ○ No ○ Not tested<br>▪ Colistin resistant ○ Yes ○ No ○ Not tested<br>**Organism 3:** ______________________ , recovered from □ lower RT □ upper RT<br>▪ MRSA/VRE/3GCR[1] ○ Yes ○ No ○ Not tested<br>▪ Carbapenem resistant ○ Yes ○ No ○ Not tested<br>▪ Colistin resistant ○ Yes ○ No ○ Not tested
□ **At least one of the following imaging findings (imaging technologies: X-ray, CT, MRI, ultrasound) shows new changes suggestive of pneumonia, such as infiltrate, shadowing, opacification, increased density, fluid in the intrapleural cavity or interlobar fissure**
□ **New initiation of respiratory support or escalation of existing level of respiratory support for ≥ 2 days after at least 2 days of stability or improvement**
**Clinical and laboratory criteria:**<br>□ New/more frequent bradycardia episodes (<80/min) or unexplained tachycardia (>200/min)<br>□ New or increased frequency of episodes of apnoea (> 20 s) or new or more frequent tachypnoea (>60/min).<br>□ Purulent tracheal aspirate<br>□ New or more frequent symptoms of respiratory distress (retraction, nasal flaring, grunting, chest indrawing)<br>□ Temperature instability or fever (>38 °C) or hypothermia (<36.5 °C)<br>□ Increased respiratory secretion (more frequent endotracheal suctioning required)<br>□ CRP > 10 mg/L (> 1 mg/dl) or increased levels of interleukin 6 (IL-6) or IL-8<br>□ I/T - ratio > 0.2
**Secondary BSI:**<br>○ Yes ○ No ○ No follow-up<br>**If you chose YES, please enter organism(s) recovered from blood culture:**<br>**Organism 1:** ______________________<br>▪ MRSA/VRE/3GCR[1] ○ Yes ○ No ○ Not tested<br>▪ Carbapenem resistant ○ Yes ○ No ○ Not tested<br>▪ Colistin resistant ○ Yes ○ No ○ Not tested<br>**Organism 2:** ______________________<br>▪ MRSA/VRE/3GCR[1] ○ Yes ○ No ○ Not tested<br>▪ Carbapenem resistant ○ Yes ○ No ○ Not tested<br>▪ Colistin resistant ○ Yes ○ No ○ Not tested<br>**Organism 3:** ______________________<br>▪ MRSA/VRE/3GCR[1] ○ Yes ○ No ○ Not tested<br>▪ Carbapenem resistant ○ Yes ○ No ○ Not tested<br>▪ Colistin resistant ○ Yes ○ No ○ Not tested

○ *You can select only one option.* □ *You can select multiple options.*

[1] Please mark the antibiotic resistance profile appropriate to the isolated microorganism and answer accordingly.
For more information, please see sections 5. *Data Dictionary* and 7. *Abbreviations* in the NeoIPC - Core Module Protocol.

NeoIPC – Infection Data Collection Sheet

Surgical Site Infection (SSI)

Patient	
**Patient ID:**	
**Patient name:**	
**Surgical Site Infection**	
**Infection date**:	
**SSI type:**<br>○ Superficial (skin, subcutaneous)<br>○ Deep (fascial and muscle)<br>○ Organ/Space (deeper than fascial/muscle)	**Infection present at time of surgery:**<br>○ Yes<br>○ No

Organism(s) identified from surgical site:

○ Yes ○ No ○ Not tested

If you chose YES, please enter the organism(s):

Organism 1: ____

- MRSA/VRE/3GCR[1] ○ Yes ○ No ○ Not tested
- Carbapenem resistant ○ Yes ○ No ○ Not tested
- Colistin resistant ○ Yes ○ No ○ Not tested

Organism 2: ____

- MRSA/VRE/3GCR[1] ○ Yes ○ No ○ Not tested
- Carbapenem resistant ○ Yes ○ No ○ Not tested
- Colistin resistant ○ Yes ○ No ○ Not tested

Organism 3: ____

- MRSA/VRE/3GCR[1] ○ Yes ○ No ○ Not tested
- Carbapenem resistant ○ Yes ○ No ○ Not tested
- Colistin resistant ○ Yes ○ No ○ Not tested

Clinical signs and symptoms:

- ☐ Purulent drainage from the incision
- ☐ Purulent drainage from a drain
- ☐ Incision deliberately opened or aspirated
- ☐ Incision spontaneously dehisces
- ☐ Abscess or other evidence of infection
- ☐ Localized pain or tenderness
- ☐ Localized swelling
- ☐ Localized erythema
- ☐ Localized heat
- ☐ Fever (> 38 °C) or hypothermia (< 36.5 °C) or temperature instability

Secondary BSI:

○ Yes ○ No ○ No follow-up

If you chose YES, please enter organism(s) recovered from blood culture:

Organism 1: ____

- MRSA/VRE/3GCR[1] ○ Yes ○ No ○ Not tested
- Carbapenem resistant ○ Yes ○ No ○ Not tested
- Colistin resistant ○ Yes ○ No ○ Not tested

Organism 2: ____

- MRSA/VRE/3GCR[1] ○ Yes ○ No ○ Not tested
- Carbapenem resistant ○ Yes ○ No ○ Not tested
- Colistin resistant ○ Yes ○ No ○ Not tested

Organism 3: ____

- MRSA/VRE/3GCR[1] ○ Yes ○ No ○ Not tested
- Carbapenem resistant ○ Yes ○ No ○ Not tested
- Colistin resistant ○ Yes ○ No ○ Not tested

○ *You can select only one option.*

☐ *You can select multiple options.*

[1] Please mark the antibiotic resistance profile appropriate to the isolated microorganism and answer accordingly.
For more information, please see sections *5. Data Dictionary* and *7. Abbreviations* in the NeoIPC - Core Module Protocol.
