# Supplementary material for: Methodological framework for the surveillance of healthcare-associated infections in high-risk infants: the NeoIPC surveillance core module protocol
Source: Antimicrob Resist Infect Control. 2026 Feb 19;15:30. doi: 10.1186/s13756-026-01711-0 (PMC12930787; doi:10.1186/s13756-026-01711-0)
Supplement: Supplementary file 4 — Additional File 4. Surveillance infection definitons [file 13756_2026_1711_MOESM4_ESM.pdf]

Table A. Surveillance definition criteria of BSI/Sepsis

Surveillance definition criteria of BSI/Sepsis

Clinical Sepsis

1. Absence of positive microbiological blood and/or cerebrospinal fluid culture

AND,

2. Treatment with five or more days of intravenous antibiotics was initiated[1]

AND,

3. Patient has at least two of the following clinical or laboratory features of generalized infection:

- Temperature instability, fever (> 38 °C) or hypothermia (< 36.5 °C)
- New/more frequent bradycardia episodes (<80/min) or unexplained tachycardia (>200/min)
- Impaired peripheral perfusion (Capillary refill time of > 3s or skin mottling or core/peripheral temperature gap > 2 °C)
- New/more frequent episodes of apnoea (>20s) or increase in oxygen demand or ventilatory support
- Enteral feeding intolerance, abdominal distension or ileus
- Irritability, lethargy, apathy or unstable condition
- Unexplained metabolic acidosis (base excess < −10 mmol/L; <−10 mEq/L)
- New and unexplained hyperglycaemia (> 140 mg/dl; > 7.8 mmol/L) or hypoglycaemia (< 40 mg/dl; <2.2 mmol/L)
- At least one of the following laboratory findings:
    - Platelet count of < 100 × 109/L (<100 × 10^3/µL)
    - WBC < 4 × 109/L or > 20 × 109/L (< 4 × 103/µL or > 20 × 103/µL)
    - CRP > 10 mg/L (> 1 mg/dL)
    - Procalcitonin ≥ 2µg/L (2 ng/mL; 200 ng/dL)
    - I/T-Ratio > 0,2
    - Increased levels of interleukin 6 (IL-6) or IL-8[3]

LCBSI caused by a recognised pathogen[2]

Recognised pathogen[2] is recovered from a blood and/or cerebrospinal fluid culture

LCBSI caused by a common commensal[2] (1)

1. The same common commensal[2] is recovered from at least two blood culture and/or cerebrospinal fluid culture specimen collected on separate occasions

AND,

2. Patient has at least two of the following clinical or laboratory features of generalized infection:

- Temperature instability, fever (> 38 °C) or hypothermia (< 36.5 °C)
- New/more frequent bradycardia episodes (<80/min) or unexplained tachycardia (>200/min)
- Impaired peripheral perfusion (Capillary refill time of > 3s or skin mottling or core/peripheral temperature gap > 2 °C)
- New/more frequent episodes of apnoea (>20s) or increase in oxygen demand or ventilatory support
- Enteral feeding intolerance, abdominal distension or ileus
- Irritability, lethargy, apathy or unstable condition
- Unexplained metabolic acidosis (base excess < −10 mmol/L; <−10 mEq/L)
- New and unexplained hyperglycaemia (> 140 mg/dl; > 7.8 mmol/L) or hypoglycaemia (< 40 mg/dl; <2.2 mmol/L)
- At least one of the following laboratory findings:
    - Platelet count of < 100 × 109/L (<100 × 10^3/µL)
    - WBC < 4 × 109/L or > 20 × 109/L (< 4 × 103/µL or > 20 × 103/µL)
    - CRP > 10 mg/L (> 1 mg/dL)
    - Procalcitonin ≥ 2µg/L (2 ng/mL; 200 ng/dL)
    - I/T-Ratio > 0,2
    - Increased levels of interleukin 6 (IL-6) or IL-8[3]

LCBSI caused by a common commensal[2] (2)

1. A common commensal[2] is recovered from one blood culture and/or CSF culture specimen

AND,

2. At least one of the following laboratory findings:

- WBC < 4 × 109/L or > 20 × 109/L (< 4 × 103/µL or > 20 × 103/µL)
- CRP > 10 mg/L (> 1 mg/dL)
- Procalcitonin ≥ 2µg/L (2 ng/mL; 200 ng/dL)
- I/T-Ratio > 0,2

- Increased levels of interleukin 6 (IL-6) or IL-8[3]

AND,

3. Patient has at least two of the following clinical or laboratory features of generalized infection:
- Temperature instability, fever (> 38 °C) or hypothermia (< 36.5 °C)
- New/more frequent bradycardia episodes (<80/min) or unexplained tachycardia (>200/min)
- Impaired peripheral perfusion (Capillary refill time of > 3s or skin mottling or core/peripheral temperature gap > 2 °C)
- New/more frequent episodes of apnoea (>20s) or increase in oxygen demand or ventilatory support
- Enteral feeding intolerance, abdominal distension or ileus
- Irritability, lethargy, apathy or unstable condition
- Unexplained metabolic acidosis (base excess < −10 mmol/L; <−10 mEq/L)
- New and unexplained hyperglycaemia (> 140 mg/dl; > 7.8 mmol/L) or hypoglycaemia (< 40 mg/dl; <2.2 mmol/L)
- Platelet count of < 100 × 109/L (<100 × 10^3/µL)

LCBSI caused by a common commensal[2] (3)

1. A common commensal[2] is recovered from one blood culture and/or CSF culture specimen

AND,

2. Treatment with five or more days of intravenous antibiotics was initiated[1]

AND,

3. Patient has at least two of the following clinical or laboratory features of generalized infection:
- Temperature instability, fever (> 38 °C) or hypothermia (< 36.5 °C)
- New/more frequent bradycardia episodes (<80/min) or unexplained tachycardia (>200/min)
- Impaired peripheral perfusion (Capillary refill time of > 3s or skin mottling or core/peripheral temperature gap > 2 °C)
- New/more frequent episodes of apnoea (>20s) or increase in oxygen demand or ventilatory support
- Enteral feeding intolerance, abdominal distension or ileus
- Irritability, lethargy, apathy or unstable condition
- Unexplained metabolic acidosis (base excess < −10 mmol/L; <−10 mEq/L)
- New and unexplained hyperglycaemia (> 140 mg/dl; > 7.8 mmol/L) or hypoglycaemia (< 40 mg/dl; <2.2 mmol/L)
- At least one of the following laboratory findings:
    - Platelet count of < 100 × 109/L (<100 × 10^3/µL)
    - WBC < 4 × 109/L or > 20 × 109/L (< 4 × 103/µL or > 20 × 103/µL)
    - CRP > 10 mg/L (> 1 mg/dL)
    - Procalcitonin ≥ 2µg/L (2 ng/mL; 200 ng/dL)
    - I/T-Ratio > 0,2
    - Increased levels of interleukin 6 (IL-6) or IL-8[3]

Secondary sepsis (optional)

1. The blood specimen is collected in the period between 3 days prior and 13 days after the day of primary infection[4]

AND**,**

2. At least one organism from the blood specimen matches an organism identified from the primary infection site.

BSI, bloodstream infection; WBC, white blood cells; CRP, C-Reactive protein; I/T-Ratio, immature to total granulocyte ratio; CSF, cerebrospinal fluid

[1] Antibiotic treatment for at least five days was initiated. The day of the first dose and the day of the last dose are counted. Days where no dose was administered between the first and the last dose (e.g., skipped doses because of high drug levels in therapeutic drug monitoring) are counted as if a dose had been administered. Days after the last dose are not counted regardless of the patient's measured or assumed drug level. If the infant died, was discharged, or transferred before the end of the five-day course of intravenous antibiotics, this condition is met if treatment was scheduled for five days or more.

[2] See NeoIPC infectious agent list in S4 File.

[3] Interleukin should be used as a parameter when laboratory specifications for a pathological value have been fulfilled.

[4] Day of primary infection is either the day of first symptoms or the day of first positive culture at the primary infection site

Table B. Surveillance definition criteria of pneumonia

**Surveillance definition criteria of pneumonia**	
**1.**	At least one of the following imaging findings (imaging technologies: X-ray, CT, MRI, ultrasound) that show new changes suggestive of pneumonia:<br><br>• infiltrate,<br>• shadowing,<br>• opacification,<br>• increased density,<br>• fluid in the intrapleural cavity<br>• interlobar fissure<br><br>AND,
**2.**	New initiation of respiratory support or escalation of existing level of respiratory support for ≥ 2 days after at least 2 days of stability or improvement[1]<br><br>AND,
**3.**	At least four of the following clinical or laboratory criteria:<br><br>• Organisms identified[2] from respiratory tract<br>• New/more frequent bradycardia episodes (<80/min) or unexplained tachycardia (>200/min)<br>• New or more frequent tachypnoea (>60/min) or new or more frequent apnoea (> 20 s)<br>• Purulent tracheal aspirate<br>• New or more frequent symptoms of respiratory distress (retraction, nasal flaring, grunting, chest indrawing)<br>• Temperature instability or fever (>38 °C) or hypothermia (<36.5 °C)<br>• Increased respiratory secretion (more frequent endotracheal suctioning required)<br>• CRP > 10 mg/L (> 1 mg/dL) or increased levels of interleukin 6 (IL-6) or IL-8[3]<br>• I/T - ratio > 0.2

CT, computer tomography; MRI, magnetic resonance imaging; CRP, C-Reactive protein; I/T-Ratio, immature to total granulocyte ratio

[1]New initiation of respiratory support or escalation of existing level of respiratory support that does not improve within less than two days:

- Increase in need for FiO2 ≥ 0.25 (25 points) within 24 hours (daily minimum FiO2 values must be considered) OR,
- begin of non-invasive ventilatory support (excluding switch from invasive ventilation) OR,
- begin of invasive mechanical ventilation (including switch from non-invasive ventilatory support)

...that does not improve within less than 2 days: The above-mentioned condition should not improve within two days.
…after at least 2 days of stability or improvement: A stable or improving baseline period of at least two days is required before the above condition occurs.

[2] At least one organism (see below) has been identified from respiratory tract by a culture or non-culture based microbiologic testing method which is performed for purposes of clinical diagnosis or treatment (for example, NOT Active Surveillance Culture/Testing (ASC/AST)):

- Fungal or bacterial pathogen from secretions of lower respiratory tract OR,
- Viral gene, antigen or antibody from secretions of upper or lower respiratory tract (e.g. EIA, FAMA, shell vial assay, PCR)

[3] Interleukin should be used as a parameter when laboratory specifications for a pathological value have been fulfilled.

Table C. Surveillance definition criteria of NEC

Surveillance definition criteria of NEC
**1.** At least of one the following radiological signs (imaging technologies: X-ray, CT, MRI, ultrasound):<br>• Pneumoperitoneum,<br>• Pneumatosis intestinalis,<br>• Portal venous gas (Hepatobiliary gas),<br>• Fixed bowel loops (≥ 24h)<br><br>AND,<br><br>**2.** At least one of the following clinical signs:<br>• Abdominal distention<br>• Abdominal discoloration or shiny/reddish skin tone,<br>• Repeated occult (guaiac test) or visible blood in stool[1],<br>• Increasing/pronounced vomiting (e.g. bilious or bloody)<br>• Increased gastric residuals from previous feeding<br>• Bilious gastric aspirate[2]
OR**,**
**1.** At least of one the following surgical or pathological findings:<br>• Extensive bowel necrosis[3]<br>• Pneumatosis intestinalis

NEC, necrotising enterocolitis; CT, computer tomography; MRI, magnetic resonance imaging

[1] no anal fissure

[2] not from transpyloric feeding tube

[3] More than 2 cm of bowel involvement

Table D. Surveillance definition criteria of SSI

Surveillance definition criteria of SSI

Superficial incisional SSI

1. First symptoms occur within 30 days after the operation

AND,

2. Infection involves only skin and subcutaneous tissue of the incision

AND,

3. Patient has at least one of the following:

- Purulent drainage from the superficial incision
- Organism(s) identified from an aseptically obtained specimen from the superficial incision or subcutaneous tissue by a culture or non-culture based microbiologic testing method which is performed for purposes of clinical diagnosis or treatment (for example, not Active Surveillance Culture/Testing (ASC/AST))
- Superficial incision that is deliberately opened by a surgeon, physician[1] or physician designee and culture or non-culture-based testing of the superficial incision or subcutaneous tissue is not performed. A culture or non-culture-based test from the deep soft tissues of the incision that has a negative finding does not meet this criterion
  AND,
  Patient has at least one of the following signs or symptoms: localized pain or tenderness, localized swelling, erythema or heat.
- Diagnosis of a superficial incisional SSI by a physician[1] or physician designee.

Deep incisional SSI

1. First symptoms occur within 30 or 90 days[2] after the operation

AND,

2. Infection involves deep soft tissues of the incision[3]

AND,

3. Patient has at least one of the following:

- Purulent drainage from the deep incision
- A deep incision that spontaneously dehisces, or is deliberately opened or aspirated by a surgeon, physician[1] or physician designee
  AND,
  Organism(s) identified from the deep soft tissues of the incision by a culture or non-culture based microbiologic testing method which is performed for purposes of clinical diagnosis or treatment (for example, not Active Surveillance Culture/Testing (ASC/AST)) or culture or non-culture based microbiologic testing method is not performed. A culture or non-culture-based test from the deep soft tissues of the incision that has a negative finding does not meet this criterion
  AND,
  Patient has at least one of the following signs or symptoms: Temperature instability, fever (> 38 °C) or hypothermia (< 36.5 °C); localized pain or tenderness.
- An abscess or other evidence of infection involving the deep incision that is detected on gross anatomical or histopathologic exam, or imaging test.

Organ/space SSI

1. First symptoms occur within 30 or 90 days[2] after the operation

AND,

2. Infection involves any part of the body deeper than the fascial/muscle layers that is opened or manipulated during the operative procedure

AND,

3. Patient has at least one of the following:

- Purulent drainage from a drain that is placed into the organ/space (for example, closed suction drainage system, open drain, T-tube drain, CT-guided drainage).
- Organism(s) identified from fluid or tissue in the organ/space by a culture or non-culture based microbiologic testing method which is performed for purposes of clinical diagnosis or treatment (for example, not Active Surveillance Culture/Testing (ASC/AST)).

•	An abscess or other evidence of infection involving the organ/space that is detected on gross anatomical or histopathologic exam, or imaging test evidence suggestive of infection.

SSI, surgical site infection; CT, computer tomography

[1] The term physician for the purpose of application of the NeoIPC SSI criteria may be interpreted to mean a surgeon, infectious disease physician, emergency physician, another physician on the case, or physician’s designee (nurse practitioner or physician’s assistant).

[2] Follow-up of 90 days applies when an implant was left in place.

[3] e.g., fascial and muscle layers
