# Supplementary material for: Methodological framework for the surveillance of healthcare-associated infections in high-risk infants: the NeoIPC surveillance core module protocol
Source: Antimicrob Resist Infect Control. 2026 Feb 19;15:30. doi: 10.1186/s13756-026-01711-0 (PMC12930787; doi:10.1186/s13756-026-01711-0)
Supplement: Supplementary file 5 — Additional File 5. Infectious agents list [file 13756_2026_1711_MOESM5_ESM.pdf]

NeoIPC Infectious Agent List

The following list contains the current taxonomic names and common synonyms of the infectious agent species and genera as well as some diagnostic groups that can currently be recorded in the NeoIPC Surveillance.

Originally derived from the NHSN Organism List [NHSN], it is regularly updated with current information from the List of Prokaryotic names with Standing in Nomenclature [LPSN], the MycoBank database [MycoBank] and the ICTV database [ICTV].

We thank these organisations for generously publishing their data under a permissive licence, enabling its use in the NeoIPC Surveillance.

Name	Type	Common Commensal	Recorded Resistances
Abiotrophia	Bacterium		
Abiotrophia adiacens	Bacterium (synonym for Granulicatella adiacens)		
Abiotrophia defectiva	Bacterium		
Abiotrophia elegans	Bacterium (synonym for Granulicatella elegans)		
Acaulium acremonium	Fungus		
Acholeplasma	Bacterium		
Acholeplasma laidlawii	Bacterium		
Acholeplasma oculi	Bacterium		
Achromobacter	Bacterium		
Achromobacter denitrificans	Bacterium		
Achromobacter piechaudii	Bacterium		
Achromobacter ruhlandii	Bacterium		
Achromobacter xylosoxidans	Bacterium		
Achromobacter xylosoxidans subsp. xylosoxidans	Bacterium (synonym for Achromobacter xylosoxidans)		
Acidaminococcus	Bacterium		
Acidaminococcus fermentans	Bacterium		
Acidipropionibacterium acidipropionici	Bacterium	Yes	
Acidipropionibacterium jensenii	Bacterium	Yes	
Acidipropionibacterium microaerophilum	Bacterium	Yes	
Acidipropionibacterium thoenii	Bacterium	Yes	
Acidovorax	Bacterium		
Acidovorax delafieldii	Bacterium		
Acidovorax facilis	Bacterium		
Acidovorax temperans	Bacterium		
Acinetobacter	Bacterium		Carbapenems, Colistin
Acinetobacter baumannii	Bacterium		Carbapenems, Colistin
Acinetobacter baylyi	Bacterium		Carbapenems, Colistin
Acinetobacter beijerinckii	Bacterium		Carbapenems, Colistin
Acinetobacter bereziniae	Bacterium		Carbapenems, Colistin
Acinetobacter calcoaceticus	Bacterium		Carbapenems, Colistin
Acinetobacter calcoaceticus-Acinetobacter baumannii complex	Bacterium		Carbapenems, Colistin
Acinetobacter guillouiae	Bacterium		Carbapenems, Colistin
Acinetobacter gyllenbergii	Bacterium		Carbapenems, Colistin
Acinetobacter haemolyticus	Bacterium		Carbapenems, Colistin
Acinetobacter johnsonii	Bacterium		Carbapenems, Colistin
Acinetobacter junii	Bacterium		Carbapenems, Colistin
Acinetobacter lwoffii	Bacterium		Carbapenems, Colistin

Name	Type	Common Commensal	Recorded Resistances
Acinetobacter nosocomialis	Bacterium		Carbapenems, Colistin
Acinetobacter parvus	Bacterium		Carbapenems, Colistin
Acinetobacter pittii	Bacterium		Carbapenems, Colistin
Acinetobacter radioresistens	Bacterium		Carbapenems, Colistin
Acinetobacter schindleri	Bacterium		Carbapenems, Colistin
Acinetobacter seifertii	Bacterium		Carbapenems, Colistin
Acinetobacter septicus	Bacterium		Carbapenems, Colistin
Acinetobacter ursingii	Bacterium		Carbapenems, Colistin
Acinetobacter variabilis	Bacterium		Carbapenems, Colistin
Acladium castellanii	Fungus (synonym for Scedosporium boydii)		
Acremonium	Fungus		
Acremonium alabamense	Fungus		
Acremonium alabamensis	Fungus (synonym for Acremonium alabamense)		
Acremonium kiliense	Fungus (synonym for Sarocladium kiliense)		
Acremonium potronii	Fungus		
Acremonium recifei	Fungus (synonym for Xenoacremonium recifei)		
Acremonium strictum	Fungus (synonym for Sarocladium strictum)		
Acrotheca aquaspersa	Fungus (synonym for Rhinocladiella aquaspersa)		
Actinobacillus	Bacterium		
Actinobacillus actinomycetemcomitans	Bacterium (synonym for Aggregatibacter actinomycetemcomitans)		
Actinobacillus equuli	Bacterium		
Actinobacillus hominis	Bacterium		
Actinobacillus lignieresii	Bacterium		
Actinobacillus suis	Bacterium		
Actinobacillus ureae	Bacterium		
Actinobaculum	Bacterium		
Actinobaculum massiliense	Bacterium		
Actinobaculum schaalii	Bacterium (synonym for Actinotignum schaalii)		
Actinobaculum urinale	Bacterium (synonym for Actinotignum urinale)		
Actinomadura	Bacterium		
Actinomadura latina	Bacterium		
Actinomadura madurae	Bacterium		
Actinomadura pelletieri	Bacterium		
Actinomortierella wolfii	Fungus		
Actinomyces	Bacterium	Yes	
Actinomyces bernardiae	Bacterium (synonym for Trueperella bernardiae)	Yes	
Actinomyces bovis	Bacterium	Yes	
Actinomyces cardiffensis	Bacterium (synonym for Schaalia cardiffensis)	Yes	
Actinomyces dentalis	Bacterium	Yes	
Actinomyces europaeus	Bacterium (synonym for Gleimia europaea)	Yes	
Actinomyces funkei	Bacterium (synonym for Schaalia funkei)	Yes	
Actinomyces georgiae	Bacterium (synonym for Schaalia georgiae)	Yes	
Actinomyces gerencseriae	Bacterium	Yes	
Actinomyces graevenitzii	Bacterium	Yes	
Actinomyces hongkongensis	Bacterium (synonym for Pauljensenia hongkongensis)	Yes	
Actinomyces israelii	Bacterium	Yes	

Name	Type	Common Commensal	Recorded Resistances
Actinomyces meyeri	Bacterium (synonym for Schaalia meyeri)	Yes	
Actinomyces naeslundii	Bacterium	Yes	
Actinomyces nasicola	Bacterium (synonym for Bowdeniella nasicola)	Yes	
Actinomyces neuii	Bacterium (synonym for Winkia neuii)	Yes	
Actinomyces odontolyticus	Bacterium (synonym for Schaalia odontolytica)	Yes	
Actinomyces oricola	Bacterium	Yes	
Actinomyces oris	Bacterium	Yes	
Actinomyces radicidentis	Bacterium	Yes	
Actinomyces radingae	Bacterium (synonym for Schaalia radingae)	Yes	
Actinomyces turicensis	Bacterium (synonym for Schaalia turicensis)	Yes	
Actinomyces urogenitalis	Bacterium	Yes	
Actinomyces viscosus	Bacterium	Yes	
Actinotignum	Bacterium		
Actinotignum schaalii	Bacterium		
Actinotignum urinale	Bacterium		
Aerococcus	Bacterium	Yes	
Aerococcus christensenii	Bacterium	Yes	
Aerococcus sanguinicola	Bacterium	Yes	
Aerococcus urinae	Bacterium	Yes	
Aerococcus urinaeequi	Bacterium	Yes	
Aerococcus urinaehominis	Bacterium	Yes	
Aerococcus viridans	Bacterium	Yes	
Aeromonas	Bacterium		Colistin
Aeromonas bestiarum	Bacterium		Colistin
Aeromonas caviae	Bacterium		Colistin
Aeromonas encheleia	Bacterium		Colistin
Aeromonas enteropelogenes	Bacterium		Colistin
Aeromonas eucrenophila	Bacterium		Colistin
Aeromonas hydrophila	Bacterium		Colistin
Aeromonas jandaei	Bacterium		Colistin
Aeromonas media	Bacterium		Colistin
Aeromonas molluscorum	Bacterium		Colistin
Aeromonas popoffii	Bacterium		Colistin
Aeromonas punctata	Bacterium (synonym for Aeromonas caviae)		Colistin
Aeromonas salmonicida	Bacterium		Colistin
Aeromonas schubertii	Bacterium		Colistin
Aeromonas simiae	Bacterium		Colistin
Aeromonas sobria	Bacterium		Colistin
Aeromonas trota	Bacterium (synonym for Aeromonas enteropelogenes)		Colistin
Aeromonas veronii	Bacterium		Colistin
Afipia	Bacterium		
Afipia clevelandensis	Bacterium		
Afipia felis	Bacterium		
Agathobacter rectalis	Bacterium		
Aggregatibacter	Bacterium		
Aggregatibacter actinomycetemcomitans	Bacterium		
Aggregatibacter aphrophilus	Bacterium		

Name	Type	Common Commensal	Recorded Resistances
Aggregatibacter segnis	Bacterium		
Agrobacterium	Bacterium		
Agrobacterium radiobacter	Bacterium		
Agrobacterium rubi	Bacterium		
Agromyces	Bacterium		
Alcaligenes	Bacterium		
Alcaligenes faecalis	Bacterium		
Alcaligenes piechaudii	Bacterium (synonym for Achromobacter piechaudii)		
Alcaligenes xylosoxidans	Bacterium (synonym for Achromobacter xylosoxidans)		
Alcaligenes xylosoxidans subsp. xylosoxidans	Bacterium (synonym for Achromobacter xylosoxidans)		
Aleurisma	Fungus		
Aleurisma apiospermum	Fungus (synonym for Scedosporium apiospermum)		
Alishewanella	Bacterium		
Alishewanella fetalis	Bacterium		
Alistipes	Bacterium		
Alistipes putredinis	Bacterium		
Alkalicoccobacillus gibsonii	Bacterium		
Alkalicoccus saliphilus	Bacterium	Yes	
Alkalihalobacillus alcalophilus	Bacterium	Yes	
Alkalihalobacillus algicola	Bacterium	Yes	
Alkalihalobacillus macyae	Bacterium	Yes	
Alkalihalobacillus pseudalcaliphilus	Bacterium	Yes	
Alkalihalophilus pseudofirmus	Bacterium	Yes	
Allescheria boydii	Fungus (synonym for Scedosporium boydii)		
Alloiococcus	Bacterium		
Alloiococcus otitis	Bacterium		
Alloprevotella tannerae	Bacterium		Colistin
Allorhizobium vitis	Bacterium		
Alloscardovia	Bacterium		
Alloscardovia omnicolens	Bacterium		
Alpha-hemolytic streptococci	Bacterium		
Alphainfluenzavirus influenzae	Virus		
Alphavirus chikungunya	Virus		
Alphavirus eastern	Virus		
Alphavirus madariaga	Virus		
Alphavirus venezuelan	Virus		
Alphavirus western	Virus		
Alternaria	Fungus		
Alternaria alternata	Fungus		
Alternaria dianthicola	Fungus		
Alternaria infectoria	Fungus		
Alternaria tenuissima	Fungus		
Alysiella	Bacterium		
Alysiella crassa	Bacterium		
Amedibacillus dolichus	Bacterium		
Amesia atrobrunnea	Fungus		
Amycolatopsis	Bacterium		

Name	Type	Common Commensal	Recorded Resistances
Amycolatopsis orientalis	Bacterium		
Anaerobacillus arseniciselenatis	Bacterium	Yes	
Anaerobiospirillum	Bacterium		
Anaerobiospirillum succiniciproducens	Bacterium		
Anaerobiospirillum thomasii	Bacterium		
Anaerobutyricum hallii	Bacterium		
Anaerococcus	Bacterium		
Anaerococcus hydrogenalis	Bacterium		
Anaerococcus lactolyticus	Bacterium		
Anaerococcus octavius	Bacterium		
Anaerococcus prevotii	Bacterium		
Anaerococcus tetradius	Bacterium		
Anaerococcus vaginalis	Bacterium		
Anaerorhabdus	Bacterium		
Anaerorhabdus furcosa	Bacterium		
Anaerorhabdus furcosus	Bacterium (synonym for Anaerorhabdus furcosa)		
Anaerostipes hadrus	Bacterium		
Anaplasma	Bacterium		
Aneurinibacillus	Bacterium		
Aneurinibacillus aneurinilyticus	Bacterium		
Apiotrichum loubieri	Fungus		
Apiotrichum mycotoxinovorans	Fungus		
Apophysomyces	Fungus		
Apophysomyces elegans	Fungus		
Aquabacterium	Bacterium		
Aquabacterium parvum	Bacterium		
Arachnia propionica	Bacterium	Yes	
Arcanobacterium	Bacterium	Yes	
Arcanobacterium bernardiae	Bacterium (synonym for Trueperella bernardiae)	Yes	
Arcanobacterium haemolyticum	Bacterium	Yes	
Arcanobacterium pluranimalium	Bacterium	Yes	
Arcanobacterium pyogenes	Bacterium (synonym for Trueperella pyogenes)	Yes	
Arcobacter	Bacterium		
Arcobacter butzleri	Bacterium		
Arcobacter nitrofigilis	Bacterium		
Arsenicicoccus	Bacterium		
Arsenicicoccus bolidensis	Bacterium		
Arthrobacter	Bacterium	Yes	
Arthrobacter agilis	Bacterium	Yes	
Arthrobacter albus	Bacterium (synonym for Pseudoglutamicibacter albus)	Yes	
Arthrobacter arilaitensis	Bacterium (synonym for Glutamicibacter arilaitensis)	Yes	
Arthrobacter aurescens	Bacterium (synonym for Paenarthrobacter aurescens)	Yes	
Arthrobacter bergerei	Bacterium (synonym for Glutamicibacter bergerei)	Yes	
Arthrobacter chlorophenolicus	Bacterium (synonym for Pseudarthrobacter chlorophenolicus)	Yes	
Arthrobacter citreus	Bacterium	Yes	

Name	Type	Common Commensal	Recorded Resistances
Arthrobacter creatinolyticus	Bacterium (synonym for Glutamicibacter creatinolyticus)	Yes	
Arthrobacter crystallopoietes	Bacterium	Yes	
Arthrobacter cumminsii	Bacterium (synonym for Pseudoglutamicibacter cumminsii)	Yes	
Arthrobacter flavus	Bacterium	Yes	
Arthrobacter gandavensis	Bacterium	Yes	
Arthrobacter gangotriensis	Bacterium (synonym for Paeniglutamicibacter gangotriensis)	Yes	
Arthrobacter globiformis	Bacterium	Yes	
Arthrobacter histidinolovorans	Bacterium (synonym for Paenarthrobacter histidinolovorans)	Yes	
Arthrobacter ilicis	Bacterium (synonym for Paenarthrobacter ilicis)	Yes	
Arthrobacter kerguelensis	Bacterium (synonym for Paeniglutamicibacter kerguelensis)	Yes	
Arthrobacter koreensis	Bacterium	Yes	
Arthrobacter luteolus	Bacterium	Yes	
Arthrobacter methylotrophus	Bacterium	Yes	
Arthrobacter mysorens	Bacterium (synonym for Glutamicibacter nicotianae)	Yes	
Arthrobacter nasiphocae	Bacterium (synonym for Falsarthrobacter nasiphocae)	Yes	
Arthrobacter nicotianae	Bacterium (synonym for Glutamicibacter nicotianae)	Yes	
Arthrobacter nicotinovorans	Bacterium (synonym for Paenarthrobacter nicotinovorans)	Yes	
Arthrobacter nitroguajacolicus	Bacterium (synonym for Paenarthrobacter nitroguajacolicus)	Yes	
Arthrobacter oryzae	Bacterium	Yes	
Arthrobacter oxydans	Bacterium (synonym for Pseudarthrobacter oxydans)	Yes	
Arthrobacter pascens	Bacterium	Yes	
Arthrobacter polychromogenes	Bacterium (synonym for Pseudarthrobacter polychromogenes)	Yes	
Arthrobacter protophormiae	Bacterium (synonym for Glutamicibacter protophormiae)	Yes	
Arthrobacter psychrolactophilus	Bacterium	Yes	
Arthrobacter psychrophenolicus	Bacterium (synonym for Paeniglutamicibacter psychrophenolicus)	Yes	
Arthrobacter ramosus	Bacterium	Yes	
Arthrobacter rhombi	Bacterium	Yes	
Arthrobacter roseus	Bacterium	Yes	
Arthrobacter russicus	Bacterium	Yes	
Arthrobacter scleromae	Bacterium (synonym for Pseudarthrobacter scleromae)	Yes	
Arthrobacter siderocapsulatus	Bacterium (synonym for Pseudomonas putida)		3GCR, Carbapenems, Colistin
Arthrobacter sulfonivorans	Bacterium (synonym for Pseudarthrobacter sulfonivorans)	Yes	
Arthrobacter sulfureus	Bacterium (synonym for Paeniglutamicibacter sulfureus)	Yes	
Arthrobacter uratoxydans	Bacterium (synonym for Glutamicibacter uratoxydans)	Yes	
Arthrobacter ureafaciens	Bacterium (synonym for Paenarthrobacter ureafaciens)	Yes	
Arthrobacter variabilis	Bacterium (synonym for Corynebacterium variabile)	Yes	
Arthrobacter viscosus	Bacterium (synonym for Rhizobium viscosum)	Yes	
Arthrobacter woluwensis	Bacterium	Yes	

Name	Type	Common Commensal	Recorded Resistances
Arthroderma	Fungus		
Arthrographis	Fungus		
Asaccharospora irregularis	Bacterium		
Ascomycota	Fungus		
Aspergillus candidus	Fungus		
Aspergillus clavatus	Fungus		
Aspergillus deflectus	Fungus		
Aspergillus fischeri	Fungus		
Aspergillus flavipes	Fungus		
Aspergillus flavus	Fungus		
Aspergillus flavus var. oryzae	Fungus		
Aspergillus fumigatus	Fungus		
Aspergillus glaucus	Fungus		
Aspergillus nidulans	Fungus		
Aspergillus niger	Fungus		
Aspergillus niveus	Fungus		
Aspergillus ochraceus	Fungus		
Aspergillus oryzae	Fungus (synonym for Aspergillus flavus var. oryzae)		
Aspergillus parasiticus	Fungus		
Aspergillus restrictus	Fungus		
Aspergillus sydowii	Fungus		
Aspergillus terreus	Fungus		
Aspergillus ustus	Fungus		
Aspergillus versicolor	Fungus		
Aspergillus wentii	Fungus		
Astroviridae	Virus		
Atopobium	Bacterium		
Atopobium fossor	Bacterium		
Atopobium minutum	Bacterium		
Atopobium parvulum	Bacterium (synonym for Lancefieldella parvula)		Carbapenems
Atopobium rimae	Bacterium (synonym for Lancefieldella rimae)		Carbapenems
Atopobium vaginae	Bacterium (synonym for Fannyhessea vaginae)		
Aurantimonas altamirensis	Bacterium (synonym for Aureimonas altamirensis)		
Aureimonas	Bacterium		
Aureimonas altamirensis	Bacterium		
Aureobacterium liquefaciens	Bacterium (synonym for Microbacterium liquefaciens)	Yes	
Aureobasidium	Fungus		
Aureobasidium pullulans	Fungus		
Averyella	Bacterium		3GCR, Carbapenems, Colistin
Averyella dalhousiensis	Bacterium		3GCR, Carbapenems, Colistin
Azorhizobium	Bacterium		
Azorhizobium caulinodans	Bacterium		
Azospirillum	Bacterium		
Azospirillum brasilense	Bacterium		
Azymocandida mycoderma	Fungus		
Azymoprocandida lipolytica	Fungus (synonym for Yarrowia lipolytica)		
B19 virus	Virus (synonym for Erythroparvovirus primate1)		

Name	Type	Common Commensal	Recorded Resistances
Bacillus	Bacterium	Yes	
Bacillus aeolius	Bacterium	Yes	
Bacillus aerius	Bacterium	Yes	
Bacillus agaradhaerens	Bacterium (synonym for Salipaludibacillus agaradhaerens)	Yes	
Bacillus agri	Bacterium (synonym for Brevibacillus agri)	Yes	
Bacillus alcalophilus	Bacterium (synonym for Alkalihalobacillus alcalophilus)	Yes	
Bacillus algicola	Bacterium (synonym for Alkalihalobacillus algicola)	Yes	
Bacillus alvei	Bacterium (synonym for Paenibacillus alvei)	Yes	
Bacillus amyloliquefaciens	Bacterium	Yes	
Bacillus aneurinilyticus	Bacterium (synonym for Aneurinibacillus aneurinilyticus)		
Bacillus aneurinolyticus	Bacterium (synonym for Aneurinibacillus aneurinilyticus)		
Bacillus anthracis	Bacterium		
Bacillus aquimaris	Bacterium (synonym for Rossellomorea aquimaris)	Yes	
Bacillus arseniciselenatis	Bacterium (synonym for Anaerobacillus arseniciselenatis)	Yes	
Bacillus asahii	Bacterium (synonym for Peribacillus asahii)	Yes	
Bacillus atrophaeus	Bacterium	Yes	
Bacillus azotoformans	Bacterium (synonym for Schinkia azotoformans)	Yes	
Bacillus badius	Bacterium (synonym for Pseudobacillus badius)		
Bacillus barbaricus	Bacterium (synonym for Fictibacillus barbaricus)	Yes	
Bacillus bataviensis	Bacterium (synonym for Neobacillus bataviensis)	Yes	
Bacillus benzoevorans	Bacterium	Yes	
Bacillus boroniphilus	Bacterium (synonym for Mesobacillus boroniphilus)	Yes	
Bacillus brevis	Bacterium (synonym for Brevibacillus brevis)	Yes	
Bacillus butanolivorans	Bacterium (synonym for Peribacillus butanolivorans)	Yes	
Bacillus carboniphilus	Bacterium	Yes	
Bacillus centrosporus	Bacterium (synonym for Brevibacillus centrosporus)	Yes	
Bacillus cereus	Bacterium	Yes	
Bacillus cereus group	Bacterium		
Bacillus circulans	Bacterium	Yes	
Bacillus clarkii	Bacterium (synonym for Evansella clarkii)	Yes	
Bacillus clausii	Bacterium (synonym for Shouchella clausii)	Yes	
Bacillus coagulans	Bacterium	Yes	
Bacillus cohnii	Bacterium (synonym for Sutcliffiella cohnii)	Yes	
Bacillus cytotoxicus	Bacterium	Yes	
Bacillus decisifrondis	Bacterium	Yes	
Bacillus decolorationis	Bacterium (synonym for Pseudalkalibacillus decolorationis)	Yes	
Bacillus dipsosauri	Bacterium (synonym for Gracilibacillus dipsosauri)	Yes	
Bacillus drentensis	Bacterium (synonym for Neobacillus drentensis)	Yes	
Bacillus edaphicus	Bacterium (synonym for Paenibacillus edaphicus)	Yes	
Bacillus ehimensis	Bacterium (synonym for Paenibacillus ehimensis)	Yes	
Bacillus endophyticus	Bacterium (synonym for Priestia endophytica)	Yes	
Bacillus farraginis	Bacterium (synonym for Siminovitchia farraginis)	Yes	
Bacillus fastidiosus	Bacterium (synonym for Metabacillus fastidiosus)	Yes	

Name	Type	Common Commensal	Recorded Resistances
Bacillus firmus	Bacterium (synonym for Cytobacillus firmus)	Yes	
Bacillus flexus	Bacterium (synonym for Priestia flexa)	Yes	
Bacillus fordii	Bacterium (synonym for Siminovitchia fordii)	Yes	
Bacillus fortis	Bacterium (synonym for Siminovitchia fortis)	Yes	
Bacillus fumarioli	Bacterium (synonym for Neobacillus fumarioli)	Yes	
Bacillus funiculus	Bacterium (synonym for Ectobacillus funiculus)	Yes	
Bacillus galactophilus	Bacterium (synonym for Brevibacillus agri)	Yes	
Bacillus galactosidilyticus	Bacterium (synonym for Lederbergia galactosidilytica)	Yes	
Bacillus gelatini	Bacterium (synonym for Fictibacillus gelatini)	Yes	
Bacillus gibsonii	Bacterium (synonym for Alkalicoccobacillus gibsonii)		
Bacillus ginsengi	Bacterium (synonym for Bhargavaea ginsengi)	Yes	
Bacillus halmapalus	Bacterium (synonym for Sutcliffiella halmapala)	Yes	
Bacillus halodenitrificans	Bacterium (synonym for Virgibacillus halodenitrificans)	Yes	
Bacillus halodurans	Bacterium (synonym for Halalkalibacterium halodurans)	Yes	
Bacillus horikoshii	Bacterium (synonym for Sutcliffiella horikoshii)	Yes	
Bacillus horti	Bacterium	Yes	
Bacillus humi	Bacterium (synonym for Ferdinandcohnia humi)	Yes	
Bacillus hwajinpoensis	Bacterium (synonym for Pseudalkalibacillus hwajinpoensis)	Yes	
Bacillus idriensis	Bacterium (synonym for Metabacillus idriensis)	Yes	
Bacillus inaquosorum	Bacterium	Yes	
Bacillus indicus	Bacterium (synonym for Metabacillus indicus)	Yes	
Bacillus infantis	Bacterium	Yes	
Bacillus infernus	Bacterium	Yes	
Bacillus jeotgali	Bacterium (synonym for Mesobacillus jeotgali)	Yes	
Bacillus kaustophilus	Bacterium (synonym for Geobacillus kaustophilus)	Yes	
Bacillus korlensis	Bacterium (synonym for Robertmurraya korlensis)	Yes	
Bacillus krulwichiae	Bacterium (synonym for Halalkalibacter krulwichiae)	Yes	
Bacillus laevolacticus	Bacterium (synonym for Sporolactobacillus laevolacticus)		
Bacillus larvae	Bacterium (synonym for Paenibacillus larvae)	Yes	
Bacillus laterosporus	Bacterium (synonym for Brevibacillus laterosporus)	Yes	
Bacillus lentimorbus	Bacterium (synonym for Paenibacillus lentimorbus)	Yes	
Bacillus lentus	Bacterium (synonym for Lederbergia lenta)	Yes	
Bacillus licheniformis	Bacterium	Yes	
Bacillus luciferensis	Bacterium (synonym for Gottfriedia luciferensis)	Yes	
Bacillus macerans	Bacterium (synonym for Paenibacillus macerans)	Yes	
Bacillus macyae	Bacterium (synonym for Alkalihalobacillus macyae)	Yes	
Bacillus marinus	Bacterium (synonym for Jeotgalibacillus marinus)	Yes	
Bacillus marisflavi	Bacterium (synonym for Rossellomorea marisflavi)	Yes	
Bacillus massiliensis	Bacterium (synonym for Ureibacillus massiliensis)	Yes	
Bacillus megaterium	Bacterium (synonym for Priestia megaterium)	Yes	
Bacillus methanolicus	Bacterium	Yes	
Bacillus mojavensis	Bacterium	Yes	
Bacillus mucilaginosus	Bacterium (synonym for Paenibacillus mucilaginosus)	Yes	
Bacillus muralis	Bacterium (synonym for Peribacillus muralis)	Yes	
Bacillus mycoides	Bacterium	Yes	

Name	Type	Common Commensal	Recorded Resistances
Bacillus naganoensis	Bacterium (synonym for Pullulanibacillus naganoensis)	Yes	
Bacillus nealsonii	Bacterium	Yes	
Bacillus niacini	Bacterium (synonym for Neobacillus niacini)	Yes	
Bacillus novalis	Bacterium (synonym for Neobacillus novalis)	Yes	
Bacillus odysseyi	Bacterium (synonym for Lysinibacillus odysseyi)	Yes	
Bacillus okuhidensis	Bacterium (synonym for Halalkalibacterium halodurans)	Yes	
Bacillus oleronius	Bacterium (synonym for Heyndrickxia oleronia)	Yes	
Bacillus pabuli	Bacterium (synonym for Paenibacillus pabuli)	Yes	
Bacillus pallidus	Bacterium (synonym for Falsibacillus pallidus)	Yes	
Bacillus pantothenticus	Bacterium (synonym for Virgibacillus pantothenticus)	Yes	
Bacillus parabrevis	Bacterium (synonym for Brevibacillus parabrevis)	Yes	
Bacillus pasteurii	Bacterium (synonym for Sporosarcina pasteurii)	Yes	
Bacillus patagoniensis	Bacterium (synonym for Shouchella patagoniensis)	Yes	
Bacillus pocheonensis	Bacterium (synonym for Neobacillus pocheonensis)	Yes	
Bacillus polymyxa	Bacterium (synonym for Paenibacillus polymyxa)	Yes	
Bacillus popilliae	Bacterium (synonym for Paenibacillus popilliae)	Yes	
Bacillus pseudalcaliphilus	Bacterium (synonym for Alkalihalobacillus pseudalcaliphilus)	Yes	
Bacillus pseudofirmus	Bacterium (synonym for Alkalihalophilus pseudofirmus)	Yes	
Bacillus pseudomycoides	Bacterium	Yes	
Bacillus psychrophilus	Bacterium (synonym for Sporosarcina psychrophila)	Yes	
Bacillus psychrosaccharolyticus	Bacterium (synonym for Peribacillus psychrosaccharolyticus)	Yes	
Bacillus pulvifaciens	Bacterium (synonym for Paenibacillus larvae)	Yes	
Bacillus pumilus	Bacterium	Yes	
Bacillus pycnus	Bacterium (synonym for Rummeliibacillus pycnus)	Yes	
Bacillus salexigens	Bacterium (synonym for Virgibacillus salexigens)	Yes	
Bacillus saliphilus	Bacterium (synonym for Alkalicoccus saliphilus)	Yes	
Bacillus selenitireducens	Bacterium (synonym for Salisediminibacterium selenitireducens)	Yes	
Bacillus shackletonii	Bacterium (synonym for Heyndrickxia shackletonii)		
Bacillus silvestris	Bacterium (synonym for Solibacillus silvestris)	Yes	
Bacillus simplex	Bacterium (synonym for Peribacillus simplex)	Yes	
Bacillus siralis	Bacterium (synonym for Robertmurraya siralis)	Yes	
Bacillus smithii	Bacterium	Yes	
Bacillus soli	Bacterium (synonym for Neobacillus soli)	Yes	
Bacillus sonorensis	Bacterium	Yes	
Bacillus sphaericus	Bacterium (synonym for Lysinibacillus sphaericus)	Yes	
Bacillus spizizenii	Bacterium	Yes	
Bacillus sporothermodurans	Bacterium (synonym for Heyndrickxia sporothermodurans)	Yes	
Bacillus subterraneus	Bacterium (synonym for Mesobacillus subterraneus)	Yes	
Bacillus subtilis	Bacterium	Yes	
Bacillus subtilis group	Bacterium		
Bacillus subtilis subsp. inaquosorum	Bacterium (synonym for Bacillus inaquosorum)	Yes	
Bacillus subtilis subsp. spizizenii	Bacterium (synonym for Bacillus spizizenii)	Yes	
Bacillus subtilis subsp. subtilis	Bacterium (synonym for Bacillus subtilis)	Yes	

Name	Type	Common Commensal	Recorded Resistances
Bacillus tequilensis	Bacterium	Yes	
Bacillus thermantarcticus	Bacterium (synonym for Parageobacillus thermantarcticus)	Yes	
Bacillus thermoamylovorans	Bacterium	Yes	
Bacillus thermocatenulatus	Bacterium (synonym for Geobacillus thermocatenulatus)	Yes	
Bacillus thermocloacae	Bacterium	Yes	
Bacillus thermodenitrificans	Bacterium (synonym for Geobacillus thermodenitrificans)		
Bacillus thermoglucosidasius	Bacterium (synonym for Parageobacillus thermoglucosidasius)		
Bacillus thiaminolyticus	Bacterium (synonym for Paenibacillus thiaminolyticus)	Yes	
Bacillus thuringiensis	Bacterium	Yes	
Bacillus tusciae	Bacterium (synonym for Kyrpidia tusciae)	Yes	
Bacillus validus	Bacterium (synonym for Paenibacillus validus)	Yes	
Bacillus vallismortis	Bacterium	Yes	
Bacillus vedderi	Bacterium (synonym for Evansella vedderi)	Yes	
Bacillus velezensis	Bacterium	Yes	
Bacillus vietnamensis	Bacterium (synonym for Rossellomorea vietnamensis)	Yes	
Bacillus vireti	Bacterium (synonym for Neobacillus vireti)	Yes	
Bacillus weihenstephanensis	Bacterium (synonym for Bacillus mycoides)	Yes	
Bacterionema matruchotii	Bacterium (synonym for Corynebacterium matruchotii)	Yes	
Bacteroides	Bacterium		
Bacteroides bivius	Bacterium (synonym for Prevotella bivia)		
Bacteroides buccae	Bacterium (synonym for Prevotella buccae)		
Bacteroides buccalis	Bacterium (synonym for Prevotella buccalis)		
Bacteroides caccae	Bacterium		
Bacteroides capillosus	Bacterium (synonym for Pseudoflavonifractor capillosus)		
Bacteroides capillus	Bacterium (synonym for Prevotella buccae)		
Bacteroides coagulans	Bacterium (synonym for Ezakiella coagulans)		Carbapenems
Bacteroides corporis	Bacterium (synonym for Prevotella corporis)		
Bacteroides denticanum	Bacterium		
Bacteroides denticola	Bacterium (synonym for Prevotella denticola)		
Bacteroides disiens	Bacterium (synonym for Prevotella disiens)		
Bacteroides distasonis	Bacterium (synonym for Parabacteroides distasonis)		
Bacteroides dorei	Bacterium (synonym for Phocaeicola dorei)		Carbapenems
Bacteroides eggerthii	Bacterium		
Bacteroides endodontalis	Bacterium (synonym for Porphyromonas endodontalis)		
Bacteroides faecis	Bacterium		
Bacteroides fluxus	Bacterium		
Bacteroides forsythus	Bacterium (synonym for Tannerella forsythia)		
Bacteroides fragilis	Bacterium		
Bacteroides fragilis group	Bacterium		
Bacteroides furcosus	Bacterium (synonym for Anaerorhabdus furcosa)		
Bacteroides galacturonicus	Bacterium		
Bacteroides gingivalis	Bacterium (synonym for Porphyromonas gingivalis)		
Bacteroides goldsteinii	Bacterium (synonym for Parabacteroides goldsteinii)		

Name	Type	Common Commensal	Recorded Resistances
Bacteroides gracilis	Bacterium (synonym for Campylobacter gracilis)		
Bacteroides heparinolyticus	Bacterium (synonym for Prevotella heparinolytica)		
Bacteroides hypermegas	Bacterium (synonym for Megamonas hypermegale)		
Bacteroides intermedius	Bacterium (synonym for Prevotella intermedia)		
Bacteroides levii	Bacterium (synonym for Porphyromonas levii)		
Bacteroides macacae	Bacterium (synonym for Porphyromonas macacae)		
Bacteroides massiliensis	Bacterium (synonym for Phocaeicola massiliensis)		Carbapenems
Bacteroides melaninogenicus	Bacterium (synonym for Prevotella melaninogenica)		
Bacteroides merdae	Bacterium (synonym for Parabacteroides merdae)		
Bacteroides multiacidus	Bacterium (synonym for Mitsuokella multacida)		
Bacteroides nodosus	Bacterium (synonym for Dichelobacter nodosus)		
Bacteroides nordii	Bacterium		
Bacteroides ochraceus	Bacterium (synonym for Capnocytophaga ochracea)		
Bacteroides oralis	Bacterium (synonym for Prevotella oralis)		
Bacteroides oris	Bacterium (synonym for Prevotella oris)		
Bacteroides oulorum	Bacterium (synonym for Prevotella oulorum)		
Bacteroides ovatus	Bacterium		
Bacteroides pectinophilus	Bacterium		
Bacteroides pentosaceus	Bacterium (synonym for Prevotella buccae)		
Bacteroides praeacutus	Bacterium (synonym for Tissierella praeacuta)		
Bacteroides putredinis	Bacterium (synonym for Alistipes putredinis)		
Bacteroides pyogenes	Bacterium		
Bacteroides ruminicola	Bacterium (synonym for Prevotella ruminicola)		
Bacteroides salivosus	Bacterium (synonym for Porphyromonas macacae)		
Bacteroides salyersiae	Bacterium		
Bacteroides splanchnicus	Bacterium (synonym for Odoribacter splanchnicus)		
Bacteroides stercoris	Bacterium		
Bacteroides suis	Bacterium (synonym for Bacteroides pyogenes)		
Bacteroides tectus	Bacterium (synonym for Bacteroides pyogenes)		
Bacteroides thetaiotaomicron	Bacterium		
Bacteroides uniformis	Bacterium		
Bacteroides ureolyticus	Bacterium (synonym for Campylobacter ureolyticus)		
Bacteroides veroralis	Bacterium (synonym for Prevotella veroralis)		
Bacteroides vulgatus	Bacterium (synonym for Phocaeicola vulgatus)		Carbapenems
Bacteroides zoogleoformans	Bacterium		
Balneatrix	Bacterium		
Balneatrix alpica	Bacterium		
Bartonella	Bacterium		
Bartonella bacilliformis	Bacterium		
Bartonella clarridgeiae	Bacterium		
Bartonella elizabethae	Bacterium		
Bartonella henselae	Bacterium		
Bartonella quintana	Bacterium		
Bartonella vinsonii	Bacterium		
Basidiobolus	Fungus		
Basidiobolus haptosporus	Fungus		
Basidiobolus heterosporus	Fungus		

Name	Type	Common Commensal	Recorded Resistances
Basidiobolus meristosporus	Fungus		
Basidiobolus ranarum	Fungus		
Beauveria	Fungus		
Beauveria bassiana	Fungus		
Beneckea alginolytica	Bacterium (synonym for Vibrio alginolyticus)		Colistin
Beneckea harveyi	Bacterium (synonym for Vibrio harveyi)		Colistin
Beneckea parahaemolytica	Bacterium (synonym for Vibrio parahaemolyticus)		Colistin
Beneckea vulnifica	Bacterium (synonym for Vibrio vulnificus)		Colistin
Bergeyella	Bacterium		
Bergeyella zoohelcum	Bacterium		
Beta-hemolytic streptococci	Bacterium		
Betacoronavirus pandemicum	Virus		
Betainfluenzavirus influenzae	Virus		
Betapolyomavirus hominis	Virus		
Betapolyomavirus macacae	Virus		
Betapolyomavirus secuhominis	Virus		
Bhargavaea ginsengi	Bacterium	Yes	
Bifidobacterium	Bacterium		
Bifidobacterium adolescentis	Bacterium		
Bifidobacterium angulatum	Bacterium		
Bifidobacterium bifidum	Bacterium		
Bifidobacterium breve	Bacterium		
Bifidobacterium catenulatum	Bacterium		
Bifidobacterium denticolens	Bacterium (synonym for Parascardovia denticolens)		
Bifidobacterium dentium	Bacterium		
Bifidobacterium gallicum	Bacterium		
Bifidobacterium infantis	Bacterium (synonym for Bifidobacterium longum subsp. infantis)		
Bifidobacterium inopinatum	Bacterium (synonym for Scardovia inopinata)		
Bifidobacterium longum	Bacterium		
Bifidobacterium longum subsp. infantis	Bacterium		
Bifidobacterium longum subsp. suis	Bacterium		
Bifidobacterium pseudocatenulatum	Bacterium		
Bifidobacterium scardovii	Bacterium		
Bifidobacterium suis	Bacterium (synonym for Bifidobacterium longum subsp. suis)		
Bilophila	Bacterium		
Bilophila wadsworthia	Bacterium		
Bipolaris	Fungus		
Bipolaris australiensis	Fungus (synonym for Curvularia australiensis)		
Bipolaris hawaiiensis	Fungus (synonym for Curvularia hawaiiensis)		
Bipolaris spicifera	Fungus (synonym for Curvularia spicifera)		
Bisifusarium dimerum	Fungus		
BK polyomavirus	Virus (synonym for Betapolyomavirus hominis)		
Blastobotrys	Fungus		
Blastoschizomyces	Fungus (synonym for Magnusiomyces)		
Blautia	Bacterium		

Name	Type	Common Commensal	Recorded Resistances
Blautia coccoides	Bacterium		
Blautia hansenii	Bacterium		
Blautia producta	Bacterium		
Bocaparvovirus	Virus		
Bocavirus	Virus (synonym for Bocaparvovirus)		
Bordetella	Bacterium		
Bordetella avium	Bacterium		
Bordetella bronchiseptica	Bacterium		
Bordetella hinzii	Bacterium		
Bordetella holmesii	Bacterium		
Bordetella parapertussis	Bacterium		
Bordetella pertussis	Bacterium		
Bordetella petrii	Bacterium		
Bordetella trematum	Bacterium		
Borrelia	Bacterium		
Borrelia afzelii	Bacterium		
Borrelia anserina	Bacterium		
Borrelia burgdorferi	Bacterium		
Borrelia caucasica	Bacterium		
Borrelia coriaceae	Bacterium		
Borrelia crocidurae	Bacterium		
Borrelia duttonii	Bacterium		
Borrelia garinii	Bacterium		
Borrelia hermsii	Bacterium		
Borrelia hispanica	Bacterium		
Borrelia japonica	Bacterium		
Borrelia lusitaniae	Bacterium		
Borrelia mazzottii	Bacterium		
Borrelia miyamotoi	Bacterium		
Borrelia parkeri	Bacterium		
Borrelia recurrentis	Bacterium		
Borrelia tanukii	Bacterium		
Borrelia theileri	Bacterium		
Borrelia turicatae	Bacterium		
Borrelia valaisiana	Bacterium		
Borrelia venezuelensis	Bacterium		
Botryodiplodia	Fungus		
Botryodiplodia theobromae	Fungus (synonym for Lasiodiplodia theobromae)		
Bowdeniella nasicola	Bacterium	Yes	
Brachybacterium	Bacterium		
Brachybacterium muris	Bacterium		
Brachycladium spiciferum	Fungus (synonym for Curvularia spicifera)		
Brachyspira	Bacterium		
Brachyspira aalborgi	Bacterium		
Brachyspira pilosicoli	Bacterium		
Branhamella catarrhalis	Bacterium (synonym for Moraxella catarrhalis)		
Brevibacillus	Bacterium	Yes	

Name	Type	Common Commensal	Recorded Resistances
Brevibacillus agri	Bacterium	Yes	
Brevibacillus brevis	Bacterium	Yes	
Brevibacillus centrosporus	Bacterium	Yes	
Brevibacillus laterosporus	Bacterium	Yes	
Brevibacillus parabrevis	Bacterium	Yes	
Brevibacterium	Bacterium	Yes	
Brevibacterium albidum	Bacterium (synonym for Curtobacterium citreum)		
Brevibacterium ammoniagenes	Bacterium (synonym for Corynebacterium ammoniagenes)	Yes	
Brevibacterium casei	Bacterium	Yes	
Brevibacterium epidermidis	Bacterium	Yes	
Brevibacterium imperiale	Bacterium (synonym for Microbacterium imperiale)	Yes	
Brevibacterium linens	Bacterium	Yes	
Brevibacterium luteolum	Bacterium	Yes	
Brevibacterium mcbrellneri	Bacterium	Yes	
Brevibacterium oxydans	Bacterium (synonym for Microbacterium oxydans)	Yes	
Brevibacterium ravenspurgense	Bacterium	Yes	
Brevibacterium sanguinis	Bacterium	Yes	
Brevibacterium vitaeruminis	Bacterium (synonym for Corynebacterium vitaeruminis)	Yes	
Brevibacterium vitarumen	Bacterium (synonym for Corynebacterium vitaeruminis)	Yes	
Brevundimonas	Bacterium		
Brevundimonas diminuta	Bacterium		
Brevundimonas vesicularis	Bacterium		
Brucella	Bacterium		
Brucella abortus	Bacterium (synonym for Brucella melitensis)		
Brucella anthropi	Bacterium		
Brucella canis	Bacterium (synonym for Brucella melitensis)		
Brucella intermedia	Bacterium		
Brucella melitensis	Bacterium		
Brucella neotomae	Bacterium (synonym for Brucella melitensis)		
Brucella ovis	Bacterium (synonym for Brucella melitensis)		
Brucella suis	Bacterium (synonym for Brucella melitensis)		
Budvicia	Bacterium		3GCR, Carbapenems, Colistin
Budvicia aquatica	Bacterium		3GCR, Carbapenems, Colistin
Bulleidia	Bacterium		
Bulleidia extructa	Bacterium		
Burkholderia	Bacterium		
Burkholderia ambifaria	Bacterium		
Burkholderia anthina	Bacterium		
Burkholderia arboris	Bacterium		
Burkholderia cenocepacia	Bacterium		
Burkholderia cepacia	Bacterium		
Burkholderia cepacia complex	Bacterium		
Burkholderia contaminans	Bacterium		
Burkholderia diffusa	Bacterium		
Burkholderia dolosa	Bacterium		

Name	Type	Common Commensal	Recorded Resistances
Burkholderia fungorum	Bacterium (synonym for Paraburkholderia fungorum)		
Burkholderia gladioli	Bacterium		
Burkholderia lata	Bacterium		
Burkholderia latens	Bacterium		
Burkholderia mallei	Bacterium		
Burkholderia metallica	Bacterium		
Burkholderia multivorans	Bacterium		
Burkholderia pickettii	Bacterium (synonym for Ralstonia pickettii)		
Burkholderia pseudomallei	Bacterium		
Burkholderia pyrrocinia	Bacterium		
Burkholderia stabilis	Bacterium		
Burkholderia thailandensis	Bacterium		
Burkholderia ubonensis	Bacterium		
Burkholderia vietnamiensis	Bacterium		
Buttiauxella	Bacterium		3GCR, Carbapenems, Colistin
Buttiauxella agrestis	Bacterium		3GCR, Carbapenems, Colistin
Buttiauxella brennerae	Bacterium		3GCR, Carbapenems, Colistin
Buttiauxella ferragutiae	Bacterium		3GCR, Carbapenems, Colistin
Buttiauxella gaviniae	Bacterium		3GCR, Carbapenems, Colistin
Buttiauxella izardii	Bacterium		3GCR, Carbapenems, Colistin
Buttiauxella noackiae	Bacterium		3GCR, Carbapenems, Colistin
Buttiauxella warmboldiae	Bacterium		3GCR, Carbapenems, Colistin
Butyricimonas	Bacterium		
Butyricimonas virosa	Bacterium		
Butyrivibrio	Bacterium		
Butyrivibrio fibrisolvens	Bacterium		
Butyrivibrio hungatei	Bacterium		
Caliciviridae	Virus		
California encephalitis orthobunyavirus	Virus (synonym for Orthobunyavirus encephalitidis)		
California encephalitis virus	Virus (synonym for Orthobunyavirus encephalitidis)		
Calymmatobacterium	Bacterium (synonym for Klebsiella)		3GCR, Carbapenems, Colistin
Calymmatobacterium granulomatis	Bacterium (synonym for Klebsiella granulomatis)		3GCR, Carbapenems, Colistin
Campylobacter	Bacterium		
Campylobacter butzleri	Bacterium (synonym for Arcobacter butzleri)		
Campylobacter cinaedi	Bacterium (synonym for Helicobacter cinaedi)		
Campylobacter coli	Bacterium		
Campylobacter concisus	Bacterium		
Campylobacter curvus	Bacterium		
Campylobacter fennelliae	Bacterium (synonym for Helicobacter fennelliae)		
Campylobacter fetus	Bacterium		
Campylobacter fetus subsp. fetus	Bacterium		
Campylobacter fetus subsp. venerealis	Bacterium		
Campylobacter gracilis	Bacterium		
Campylobacter helveticus	Bacterium		
Campylobacter hominis	Bacterium		
Campylobacter hyointestinalis	Bacterium		
Campylobacter insulaenigrae	Bacterium		

Name	Type	Common Commensal	Recorded Resistances
Campylobacter jejuni	Bacterium		
Campylobacter jejuni subsp. doylei	Bacterium		
Campylobacter jejuni subsp. jejuni	Bacterium		
Campylobacter lanienae	Bacterium		
Campylobacter lari	Bacterium		
Campylobacter laridis	Bacterium (synonym for Campylobacter lari)		
Campylobacter mucosalis	Bacterium		
Campylobacter mustelae	Bacterium (synonym for Helicobacter mustelae)		
Campylobacter nitrofigilis	Bacterium (synonym for Arcobacter nitrofigilis)		
Campylobacter pylori	Bacterium (synonym for Helicobacter pylori)		
Campylobacter rectus	Bacterium		
Campylobacter showae	Bacterium		
Campylobacter sputorum	Bacterium		
Campylobacter sputorum subsp. bubulus	Bacterium		
Campylobacter sputorum subsp. mucosalis	Bacterium (synonym for Campylobacter mucosalis)		
Campylobacter sputorum subsp. sputorum	Bacterium		
Campylobacter upsaliensis	Bacterium		
Campylobacter ureolyticus	Bacterium		
Candida	Fungus		
Candida albicans	Fungus		
Candida auris	Fungus (synonym for Candidozyma auris)		
Candida brumptii	Fungus		
Candida catenulata	Fungus (synonym for Diutina catenulata)		
Candida ciferrii	Fungus (synonym for Trichomonascus ciferrii)		
Candida dubliniensis	Fungus		
Candida duobushaemuli	Fungus (synonym for Candidozyma duobushaemuli)		
Candida duobushaemulonii	Fungus (synonym for Candidozyma duobushaemuli)		
Candida duobushaemulonis	Fungus (synonym for Candidozyma duobushaemuli)		
Candida guilliermondii	Fungus (synonym for Meyerozyma guilliermondii)		
Candida guilliermondii var. membranaefaciens	Fungus (synonym for Kodamaea ohmeri)		
Candida guilliermondii var. membranifaciens	Fungus (synonym for Kodamaea ohmeri)		
Candida haemuli	Fungus (synonym for Candidozyma haemuli)		
Candida haemuloni	Fungus (synonym for Candidozyma haemuli)		
Candida haemulonii	Fungus (synonym for Candidozyma haemuli)		
Candida inconspicua	Fungus (synonym for Pichia inconspicua)		
Candida intermedia	Fungus (synonym for Sungouiella intermedia)		
Candida krusei	Fungus (synonym for Pichia kudriavzevii)		
Candida lambica	Fungus (synonym for Pichia fermentans)		
Candida lipolytica	Fungus (synonym for Yarrowia lipolytica)		
Candida lusitaniae	Fungus (synonym for Clavispora lusitaniae)		
Candida metapsilosis	Fungus		
Candida orthopsilosis	Fungus		
Candida parapsilosis	Fungus		
Candida parapsilosis complex	Fungus		
Candida pararugosa	Fungus (synonym for Wickerhamiella pararugosa)		
Candida pintolopesii	Fungus		
Candida pintolopesii var. pintolopesii	Fungus		

Name	Type	Common Commensal	Recorded Resistances
Candida ravautii	Fungus (synonym for Diutina catenulata)		
Candida rugosa	Fungus (synonym for Diutina rugosa)		
Candida sake	Fungus		
Candida slooffiae	Fungus		
Candida slooffii	Fungus (synonym for Candida slooffiae)		
Candida stellatoidea	Fungus (synonym for Candida albicans)		
Candida tropicalis	Fungus		
Candida vini	Fungus (synonym for Azymocandida mycoderma)		
Candida zeylanoides	Fungus		
Candidozyma	Fungus		
Candidozyma auris	Fungus		
Candidozyma duobushaemuli	Fungus		
Candidozyma haemuli	Fungus		
Canicola haemoglobinophilus	Bacterium (synonym for Haemophilus haemoglobinophilus)		3GCR, Carbapenems, Colistin
Capnocytophaga	Bacterium		
Capnocytophaga canimorsus	Bacterium		
Capnocytophaga cynodegmi	Bacterium		
Capnocytophaga gingivalis	Bacterium		
Capnocytophaga granulosa	Bacterium		
Capnocytophaga haemolytica	Bacterium		
Capnocytophaga ochracea	Bacterium		
Capnocytophaga sputigena	Bacterium		
Cardiobacterium	Bacterium		
Cardiobacterium hominis	Bacterium		
Cardiobacterium valvarum	Bacterium		
Catabacter	Bacterium (synonym for Christensenella)		
Catabacter hongkongensis	Bacterium		
Catenibacterium	Bacterium		
Catenibacterium mitsuokai	Bacterium		
Catonella	Bacterium		
Catonella morbi	Bacterium		
Cedecea	Bacterium		3GCR, Carbapenems, Colistin
Cedecea davisae	Bacterium		3GCR, Carbapenems, Colistin
Cedecea lapagei	Bacterium		3GCR, Carbapenems, Colistin
Cedecea neteri	Bacterium		3GCR, Carbapenems, Colistin
Cellulomonas	Bacterium	Yes	
Cellulomonas cellulans	Bacterium (synonym for Cellulosimicrobium cellulans)	Yes	
Cellulomonas hominis	Bacterium	Yes	
Cellulomonas humilata	Bacterium	Yes	
Cellulosimicrobium	Bacterium	Yes	
Cellulosimicrobium cellulans	Bacterium	Yes	
Centipeda	Bacterium		
Centipeda periodontii	Bacterium		
Cephalosporium	Fungus		
Cephalosporium recifei	Fungus (synonym for Xenoacremonium recifei)		
Chaetomium	Fungus		

Name	Type	Common Commensal	Recorded Resistances
Chaetomium atrobrunneum	Fungus (synonym for Amesia atrobrunnea)		
Chaetomium globosum	Fungus		
Chikungunya virus	Virus (synonym for Alphavirus chikungunya)		
Chlamydia	Bacterium		
Chlamydia abortus	Bacterium		
Chlamydia pneumoniae	Bacterium		
Chlamydia psittaci	Bacterium		
Chlamydia trachomatis	Bacterium		
Chlamydophila	Bacterium (synonym for Chlamydia)		
Chlamydophila abortus	Bacterium (synonym for Chlamydia abortus)		
Chlamydophila pneumoniae	Bacterium (synonym for Chlamydia pneumoniae)		
Chlamydophila psittaci	Bacterium (synonym for Chlamydia psittaci)		
Christensenella	Bacterium		
Christensenella hongkongensis	Bacterium (synonym for Catabacter hongkongensis)		
Chromobacterium	Bacterium		
Chromobacterium violaceum	Bacterium		
Chryseobacterium	Bacterium		
Chryseobacterium gleum	Bacterium		
Chryseobacterium indologenes	Bacterium		
Chryseobacterium meningosepticum	Bacterium (synonym for Elizabethkingia meningoseptica)		
Chryseomonas	Bacterium (synonym for Pseudomonas)		3GCR, Carbapenems, Colistin
Chryseomonas luteola	Bacterium (synonym for Pseudomonas luteola)		
Chryseomonas polytricha	Bacterium (synonym for Pseudomonas luteola)		
Chrysosporium	Fungus		
Citrobacter	Bacterium		3GCR, Carbapenems, Colistin
Citrobacter amalonaticus	Bacterium		3GCR, Carbapenems, Colistin
Citrobacter braakii	Bacterium		3GCR, Carbapenems, Colistin
Citrobacter diversus	Bacterium (synonym for Citrobacter koseri)		3GCR, Carbapenems, Colistin
Citrobacter farmeri	Bacterium		3GCR, Carbapenems, Colistin
Citrobacter freundii	Bacterium		3GCR, Carbapenems, Colistin
Citrobacter gillenii	Bacterium		3GCR, Carbapenems, Colistin
Citrobacter koseri	Bacterium		3GCR, Carbapenems, Colistin
Citrobacter murliniae	Bacterium		3GCR, Carbapenems, Colistin
Citrobacter pasteurii	Bacterium		3GCR, Carbapenems, Colistin
Citrobacter rodentium	Bacterium		3GCR, Carbapenems, Colistin
Citrobacter sedlakii	Bacterium		3GCR, Carbapenems, Colistin
Citrobacter werkmanii	Bacterium		3GCR, Carbapenems, Colistin
Citrobacter youngae	Bacterium		3GCR, Carbapenems, Colistin
Cladophialophora	Fungus		
Cladophialophora bantiana	Fungus		
Cladosporium	Fungus		
Cladosporium cladosporioides	Fungus		
Cladosporium herbarum	Fungus		
Cladosporium sphaerospermum	Fungus		
Cladosporium werneckii	Fungus (synonym for Hortaea werneckii)		
Clavispora	Fungus		

Name	Type	Common Commensal	Recorded Resistances
Clavispora lusitaniae	Fungus		
Clostridioides	Bacterium		
Clostridioides difficile	Bacterium		
Clostridium	Bacterium		
Clostridium absonum	Bacterium (synonym for Clostridium sardiniense)		
Clostridium aldenense	Bacterium (synonym for Enterocloster aldenensis)		
Clostridium amygdalinum	Bacterium (synonym for Lacrimispora amygdalina)		
Clostridium argentinense	Bacterium		
Clostridium barati	Bacterium (synonym for Clostridium baratii)		
Clostridium baratii	Bacterium		
Clostridium barkeri	Bacterium (synonym for Eubacterium barkeri)		
Clostridium bartlettii	Bacterium (synonym for Intestinibacter bartlettii)		
Clostridium beijerinckii	Bacterium		
Clostridium bifermentans	Bacterium (synonym for Paraclostridium bifermentans)		
Clostridium bolteae	Bacterium (synonym for Enterocloster bolteae)		
Clostridium botulinum	Bacterium		
Clostridium budayi	Bacterium		
Clostridium butyricum	Bacterium		
Clostridium cadaveris	Bacterium		
Clostridium carnis	Bacterium		
Clostridium celatum	Bacterium		
Clostridium celerecrescens	Bacterium (synonym for Lacrimispora celerecrescens)		
Clostridium chauvoei	Bacterium		
Clostridium citroniae	Bacterium (synonym for Enterocloster citroniae)		
Clostridium clostridioforme	Bacterium (synonym for Enterocloster clostridioformis)		
Clostridium cochlearium	Bacterium		
Clostridium cocleatum	Bacterium		
Clostridium combesii	Bacterium		
Clostridium difficile	Bacterium (synonym for Clostridioides difficile)		
Clostridium disporicum	Bacterium		
Clostridium fallax	Bacterium		
Clostridium ghoni	Bacterium (synonym for Paeniclostridium ghonii)		
Clostridium ghonii	Bacterium (synonym for Paeniclostridium ghonii)		
Clostridium glycolicum	Bacterium (synonym for Terrisporobacter glycolicus)		
Clostridium haemolyticum	Bacterium		
Clostridium hastiforme	Bacterium (synonym for Tissierella praeacuta)		
Clostridium hathewayi	Bacterium (synonym for Hungatella hathewayi)		
Clostridium histolyticum	Bacterium (synonym for Hathewaya histolytica)		
Clostridium hydrogeniformans	Bacterium		
Clostridium indolis	Bacterium (synonym for Lacrimispora indolis)		
Clostridium innocuum	Bacterium		
Clostridium intestinale	Bacterium		
Clostridium irregulare	Bacterium (synonym for Asaccharospora irregularis)		
Clostridium irregularis	Bacterium (synonym for Asaccharospora irregularis)		
Clostridium lavalense	Bacterium (synonym for Enterocloster lavalensis)		
Clostridium lentoputrescens	Bacterium (synonym for Clostridium cochlearium)		
Clostridium leptum	Bacterium		

Name	Type	Common Commensal	Recorded Resistances
Clostridium limosum	Bacterium (synonym for Hathewaya limosa)		
Clostridium malenominatum	Bacterium		
Clostridium moniliforme	Bacterium		
Clostridium neonatale	Bacterium		
Clostridium novyi	Bacterium		
Clostridium orbiscindens	Bacterium (synonym for Flavonifractor plautii)		
Clostridium oroticum	Bacterium (synonym for Faecalicatena orotica)		
Clostridium paraperfringens	Bacterium (synonym for Clostridium baratii)		
Clostridium paraputrificum	Bacterium		
Clostridium perenne	Bacterium (synonym for Clostridium baratii)		
Clostridium perfringens	Bacterium		
Clostridium putrefaciens	Bacterium		
Clostridium ramosum	Bacterium		
Clostridium sardiniense	Bacterium		
Clostridium septicum	Bacterium		
Clostridium sordellii	Bacterium (synonym for Paeniclostridium sordellii)		
Clostridium sphenoides	Bacterium (synonym for Lacrimispora sphenoides)		
Clostridium spiroforme	Bacterium		
Clostridium sporogenes	Bacterium		
Clostridium sporosphaeroides	Bacterium (synonym for Faecalispora sporosphaeroides)		
Clostridium subterminale	Bacterium		
Clostridium symbiosum	Bacterium		
Clostridium tertium	Bacterium		
Clostridium tetani	Bacterium		
Clostridium ventriculi	Bacterium (synonym for Sarcina ventriculi)		
Clostridium villosum	Bacterium (synonym for Filifactor villosus)		
Coagulase-negative staphylococci	Bacterium		
Coagulase-positive staphylococci	Bacterium		
Cokeromyces	Fungus		
Cokeromyces recurvatus	Fungus		
Collinsella	Bacterium		
Collinsella aerofaciens	Bacterium		
Collinsella intestinalis	Bacterium		
Collinsella stercoris	Bacterium		
Colorado tick fever coltivirus	Virus (synonym for Coltivirus dermacentoris)		
Colorado tick fever virus	Virus (synonym for Coltivirus dermacentoris)		
Coltivirus dermacentoris	Virus		
Comamonas	Bacterium		
Comamonas acidovorans	Bacterium (synonym for Delftia acidovorans)		
Comamonas aquatica	Bacterium		
Comamonas kerstersii	Bacterium		
Comamonas terrigena	Bacterium		
Comamonas testosteroni	Bacterium		
Conidiobolus	Fungus		
Conidiobolus coronatus	Fungus		
Conidiobolus incongruus	Fungus		
Coniochaeta	Fungus		

Name	Type	Common Commensal	Recorded Resistances
Coniochaeta hoffmannii	Fungus		
Coniochaeta mutabilis	Fungus		
Coniothyrium	Fungus		
Coprococcus	Bacterium		
Coprococcus eutactus	Bacterium		
Cordyceps javanica	Fungus		
Corynebacterium	Bacterium	Yes	
Corynebacterium accolens	Bacterium	Yes	
Corynebacterium afermentans	Bacterium	Yes	
Corynebacterium afermentans subsp. afermentans	Bacterium	Yes	
Corynebacterium afermentans subsp. lipophilum	Bacterium	Yes	
Corynebacterium ammoniagenes	Bacterium	Yes	
Corynebacterium amycolatum	Bacterium	Yes	
Corynebacterium appendicis	Bacterium	Yes	
Corynebacterium aquatimens	Bacterium	Yes	
Corynebacterium aquilae	Bacterium	Yes	
Corynebacterium argentoratense	Bacterium	Yes	
Corynebacterium atypicum	Bacterium	Yes	
Corynebacterium aurimucosum	Bacterium	Yes	
Corynebacterium auris	Bacterium	Yes	
Corynebacterium auriscanis	Bacterium	Yes	
Corynebacterium belfantii	Bacterium	Yes	
Corynebacterium beticola	Bacterium	Yes	
Corynebacterium bovis	Bacterium	Yes	
Corynebacterium callunae	Bacterium	Yes	
Corynebacterium camporealense	Bacterium	Yes	
Corynebacterium camporealensis	Bacterium (synonym for Corynebacterium camporealense)	Yes	
Corynebacterium canis	Bacterium	Yes	
Corynebacterium capitovis	Bacterium	Yes	
Corynebacterium casei	Bacterium	Yes	
Corynebacterium caspium	Bacterium	Yes	
Corynebacterium ciconiae	Bacterium	Yes	
Corynebacterium confusum	Bacterium	Yes	
Corynebacterium coyleae	Bacterium	Yes	
Corynebacterium cystitidis	Bacterium	Yes	
Corynebacterium dentalis	Bacterium	Yes	
Corynebacterium diphtheriae	Bacterium		
Corynebacterium durum	Bacterium	Yes	
Corynebacterium efficiens	Bacterium	Yes	
Corynebacterium equi	Bacterium (synonym for Rhodococcus equi)	Yes	
Corynebacterium falsenii	Bacterium	Yes	
Corynebacterium fascians	Bacterium (synonym for Rhodococcus fascians)	Yes	
Corynebacterium felinum	Bacterium	Yes	
Corynebacterium flavescens	Bacterium	Yes	
Corynebacterium fournieri	Bacterium	Yes	
Corynebacterium freiburgense	Bacterium	Yes	

Name	Type	Common Commensal	Recorded Resistances
Corynebacterium freneyi	Bacterium	Yes	
Corynebacterium genitalium	Bacterium	Yes	
Corynebacterium glaucum	Bacterium	Yes	
Corynebacterium glucuronolyticum	Bacterium	Yes	
Corynebacterium glutamicum	Bacterium	Yes	
Corynebacterium gottingense	Bacterium	Yes	
Corynebacterium halotolerans	Bacterium	Yes	
Corynebacterium hansenii	Bacterium	Yes	
Corynebacterium hoagii	Bacterium (synonym for Rhodococcus hoagii)	Yes	
Corynebacterium imitans	Bacterium	Yes	
Corynebacterium jeikeium	Bacterium	Yes	
Corynebacterium kroppenstedtii	Bacterium	Yes	
Corynebacterium kutscheri	Bacterium	Yes	
Corynebacterium lilium	Bacterium (synonym for Corynebacterium glutamicum)	Yes	
Corynebacterium lipophiloflavum	Bacterium	Yes	
Corynebacterium lowii	Bacterium	Yes	
Corynebacterium macginleyi	Bacterium	Yes	
Corynebacterium massiliense	Bacterium	Yes	
Corynebacterium mastitidis	Bacterium	Yes	
Corynebacterium matruchotii	Bacterium	Yes	
Corynebacterium mediolanum	Bacterium	Yes	
Corynebacterium minutissimum	Bacterium	Yes	
Corynebacterium mooreparkense	Bacterium (synonym for Corynebacterium variabile)	Yes	
Corynebacterium mucifaciens	Bacterium	Yes	
Corynebacterium mycetoides	Bacterium	Yes	
Corynebacterium nigricans	Bacterium (synonym for Corynebacterium aurimucosum)	Yes	
Corynebacterium oculi	Bacterium	Yes	
Corynebacterium otitidis	Bacterium	Yes	
Corynebacterium paurometabolum	Bacterium (synonym for Tsukamurella paurometabola)	Yes	
Corynebacterium phocae	Bacterium	Yes	
Corynebacterium pilbarense	Bacterium	Yes	
Corynebacterium pilosum	Bacterium	Yes	
Corynebacterium propinquum	Bacterium	Yes	
Corynebacterium pseudodiphtheriticum	Bacterium	Yes	
Corynebacterium pseudogenitalium	Bacterium	Yes	
Corynebacterium pseudotuberculosis	Bacterium	Yes	
Corynebacterium pyogenes	Bacterium (synonym for Trueperella pyogenes)	Yes	
Corynebacterium pyruviciproducens	Bacterium	Yes	
Corynebacterium renale	Bacterium	Yes	
Corynebacterium resistens	Bacterium	Yes	
Corynebacterium riegelii	Bacterium	Yes	
Corynebacterium rouxii	Bacterium	Yes	
Corynebacterium rubrum	Bacterium	Yes	
Corynebacterium seminale	Bacterium (synonym for Corynebacterium glucuronolyticum)	Yes	
Corynebacterium simulans	Bacterium	Yes	

Name	Type	Common Commensal	Recorded Resistances
Corynebacterium singulare	Bacterium	Yes	
Corynebacterium sphenisci	Bacterium	Yes	
Corynebacterium spheniscorum	Bacterium	Yes	
Corynebacterium sputi	Bacterium	Yes	
Corynebacterium striatum	Bacterium	Yes	
Corynebacterium suicordis	Bacterium	Yes	
Corynebacterium sundsvallense	Bacterium	Yes	
Corynebacterium terpenotabidum	Bacterium	Yes	
Corynebacterium testudinoris	Bacterium	Yes	
Corynebacterium thomssenii	Bacterium	Yes	
Corynebacterium timonense	Bacterium	Yes	
Corynebacterium tuberculostearicum	Bacterium	Yes	
Corynebacterium tuscaniae	Bacterium (synonym for Corynebacterium tuscaniense)	Yes	
Corynebacterium tuscaniense	Bacterium	Yes	
Corynebacterium ulcerans	Bacterium	Yes	
Corynebacterium urealyticum	Bacterium	Yes	
Corynebacterium ureicelerivorans	Bacterium	Yes	
Corynebacterium variabile	Bacterium	Yes	
Corynebacterium variabilis	Bacterium (synonym for Corynebacterium variabile)	Yes	
Corynebacterium vitaeruminis	Bacterium	Yes	
Corynebacterium vitarumen	Bacterium (synonym for Corynebacterium vitaeruminis)	Yes	
Corynebacterium xerosis	Bacterium	Yes	
Cosenzaea	Bacterium (synonym for Proteus)		3GCR, Carbapenems
Cosenzaea myxofaciens	Bacterium (synonym for Proteus myxofaciens)		3GCR, Carbapenems
Coxiella	Bacterium		
Coxiella burnetii	Bacterium		
Crimean-Congo hemorrhagic fever orthonairovirus	Virus (synonym for Orthonairovirus haemorrhagiae)		
Crimean-Congo hemorrhagic fever virus	Virus (synonym for Orthonairovirus haemorrhagiae)		
Cronobacter	Bacterium		3GCR, Carbapenems, Colistin
Cronobacter dublinensis	Bacterium		3GCR, Carbapenems, Colistin
Cronobacter malonaticus	Bacterium		3GCR, Carbapenems, Colistin
Cronobacter muytjensii	Bacterium		3GCR, Carbapenems, Colistin
Cronobacter sakazakii	Bacterium		3GCR, Carbapenems, Colistin
Cronobacter turicensis	Bacterium		3GCR, Carbapenems, Colistin
Cryptobacterium	Bacterium		
Cryptobacterium curtum	Bacterium		
Cryptococcus aggregatus	Fungus (synonym for Wickerhamiella pararugosa)		
Cunninghamella	Fungus		
Cunninghamella bertholletiae	Fungus		
Cupriavidus	Bacterium		
Cupriavidus basilensis	Bacterium		
Cupriavidus gilardii	Bacterium		
Cupriavidus metallidurans	Bacterium		
Cupriavidus necator	Bacterium		
Cupriavidus pauculus	Bacterium		
Cupriavidus respiraculi	Bacterium		

Name	Type	Common Commensal	Recorded Resistances
Curtobacterium	Bacterium		
Curtobacterium albidum	Bacterium (synonym for Curtobacterium citreum)		
Curtobacterium citreum	Bacterium		
Curvularia	Fungus		
Curvularia australiensis	Fungus		
Curvularia geniculata	Fungus		
Curvularia hawaiiensis	Fungus		
Curvularia lunata	Fungus		
Curvularia spicifera	Fungus		
Cutaneotrichosporon cutaneum	Fungus		
Cutaneotrichosporon mucoides	Fungus		
Cutibacterium	Bacterium	Yes	
Cutibacterium acnes	Bacterium	Yes	
Cutibacterium avidum	Bacterium	Yes	
Cutibacterium granulosum	Bacterium	Yes	
Cyphellophora europaea	Fungus		
Cystobasidium minutum	Fungus		
Cytobacillus firmus	Bacterium	Yes	
Cytomegalovirus humanbeta5	Virus		
Cytophaga heparina	Bacterium (synonym for Pedobacter heparinus)		
Dactylaria constricta	Fungus (synonym for Scolecobasidium constrictum)		
Debaryomyces	Fungus		
Debaryomyces hansenii	Fungus		
Debaryomyces polymorphus	Fungus (synonym for Schwanniomyces polymorphus)		
Debaryomycetaceae	Fungus		
Deinococcus	Bacterium		
Delftia	Bacterium		
Delftia acidovorans	Bacterium		
Deltainfluenzavirus influenzae	Virus		
Deltaretrovirus priTlym1	Virus		
Deltaretrovirus priTlym2	Virus		
Deltaretrovirus priTlym3	Virus		
Deltavirus	Virus		
Dengue virus	Virus (synonym for Orthoflavivirus denguei)		
Dermabacter	Bacterium	Yes	
Dermabacter hominis	Bacterium	Yes	
Dermacoccus	Bacterium	Yes	
Dermacoccus nishinomiyaensis	Bacterium	Yes	
Dermatophilus	Bacterium		
Dermatophilus congolensis	Bacterium		
Desulfomonas	Bacterium (synonym for Desulfovibrio)		
Desulfovibrio	Bacterium		
Desulfovibrio desulfuricans	Bacterium		
Desulfovibrio piger	Bacterium		
Desulfovibrio vulgaris	Bacterium (synonym for Nitratidesulfovibrio vulgaris)		
Dialister	Bacterium		
Dialister micraerophilus	Bacterium		

Name	Type	Common Commensal	Recorded Resistances
Dialister pneumosintes	Bacterium		
Dichelobacter	Bacterium		
Dichelobacter nodosus	Bacterium		
Dietzia	Bacterium		
Dietzia cinnamea	Bacterium (synonym for Dietzia maris)		
Dietzia maris	Bacterium		
Dikarya	Fungus		
Dipodascales	Fungus		
Dipodascomycetes	Fungus		
Dipodascus klebahnii	Fungus (synonym for Geotrichum klebahnii)		
Diutina catenulata	Fungus		
Diutina rugosa	Fungus		
Dolosicoccus	Bacterium		
Dolosicoccus paucivorans	Bacterium		
Dolosigranulum	Bacterium		
Dolosigranulum pigrum	Bacterium		
Dorea	Bacterium		
Dorea formicigenerans	Bacterium		
Dorea longicatena	Bacterium		
Drechslera	Fungus (synonym for Pyrenophora)		
Drechslera halodes	Fungus (synonym for Exserohilum rostratum)		
Drechslera rostrata	Fungus (synonym for Exserohilum rostratum)		
Drechslera spicifera	Fungus (synonym for Curvularia spicifera)		
Dyella	Bacterium		
Dysgonomonas	Bacterium		
Dysgonomonas capnocytophagoides	Bacterium		
Dysgonomonas gadei	Bacterium		
Eastern equine encephalitis virus	Virus (synonym for Alphavirus eastern)		
Ebolavirus	Virus (synonym for Orthoebolavirus)		
Echovirus	Virus		
Ectobacillus funiculus	Bacterium	Yes	
Edwardsiella	Bacterium		3GCR, Carbapenems
Edwardsiella anguillimortifera	Bacterium (synonym for Edwardsiella tarda)		3GCR, Carbapenems
Edwardsiella hoshinae	Bacterium		3GCR, Carbapenems
Edwardsiella ictaluri	Bacterium		3GCR, Carbapenems
Edwardsiella tarda	Bacterium		3GCR, Carbapenems
Eggerthella	Bacterium		
Eggerthella lenta	Bacterium		
Eggerthella sinensis	Bacterium		
Eggerthia	Bacterium		
Eggerthia catenaformis	Bacterium		
Ehrlichia	Bacterium		
Ehrlichia sennetsu	Bacterium		
Eikenella	Bacterium		
Eikenella corrodens	Bacterium		
Elizabethkingia	Bacterium		
Elizabethkingia meningoseptica	Bacterium		

Name	Type	Common Commensal	Recorded Resistances
Emmonsia	Fungus		
Empedobacter	Bacterium		
Empedobacter brevis	Bacterium		
Enterobacter	Bacterium		3GCR, Carbapenems, Colistin
Enterobacter aerogenes	Bacterium (synonym for Klebsiella aerogenes)		3GCR, Carbapenems, Colistin
Enterobacter agglomerans	Bacterium (synonym for Pantoea agglomerans)		3GCR, Carbapenems, Colistin
Enterobacter amnigenus	Bacterium (synonym for Lelliottia amnigena)		3GCR
Enterobacter asburiae	Bacterium		3GCR, Carbapenems, Colistin
Enterobacter bugandensis	Bacterium		3GCR, Carbapenems, Colistin
Enterobacter cancerogenus	Bacterium		3GCR, Carbapenems, Colistin
Enterobacter cloacae	Bacterium		3GCR, Carbapenems, Colistin
Enterobacter cloacae complex	Bacterium		
Enterobacter cloacae subsp. dissolvens	Bacterium		3GCR, Carbapenems, Colistin
Enterobacter cowanii	Bacterium (synonym for Kosakonia cowanii)		3GCR
Enterobacter dissolvens	Bacterium (synonym for Enterobacter cloacae subsp. dissolvens)		3GCR, Carbapenems, Colistin
Enterobacter gergoviae	Bacterium (synonym for Pluralibacter gergoviae)		3GCR
Enterobacter hormaechei	Bacterium		3GCR, Carbapenems, Colistin
Enterobacter huaxiensis	Bacterium		3GCR, Carbapenems, Colistin
Enterobacter intermedius	Bacterium (synonym for Kluyvera intermedia)		3GCR, Carbapenems, Colistin
Enterobacter kobei	Bacterium		3GCR, Carbapenems, Colistin
Enterobacter ludwigii	Bacterium		3GCR, Carbapenems, Colistin
Enterobacter nimipressuralis	Bacterium (synonym for Lelliottia nimipressuralis)		3GCR
Enterobacter pyrinus	Bacterium (synonym for Pluralibacter pyrinus)		3GCR
Enterobacter quasihormaechei	Bacterium		3GCR, Carbapenems, Colistin
Enterobacter sakazakii	Bacterium (synonym for Cronobacter sakazakii)		3GCR, Carbapenems, Colistin
Enterobacter wuhouensis	Bacterium		3GCR, Carbapenems, Colistin
Enterocloster aldenensis	Bacterium		
Enterocloster bolteae	Bacterium		
Enterocloster citroniae	Bacterium		
Enterocloster clostridioformis	Bacterium		
Enterocloster lavalensis	Bacterium		
Enterococcus	Bacterium		VRE
Enterococcus asini	Bacterium		VRE
Enterococcus avium	Bacterium		VRE
Enterococcus canis	Bacterium		VRE
Enterococcus casseliflavus	Bacterium		
Enterococcus cecorum	Bacterium		VRE
Enterococcus dispar	Bacterium		VRE
Enterococcus durans	Bacterium		VRE
Enterococcus faecalis	Bacterium		VRE
Enterococcus faecium	Bacterium		VRE
Enterococcus flavescens	Bacterium (synonym for Enterococcus casseliflavus)		
Enterococcus gallinarum	Bacterium		
Enterococcus gilvus	Bacterium		VRE
Enterococcus haemoperoxidus	Bacterium		VRE
Enterococcus hermanniensis	Bacterium		VRE

Name	Type	Common Commensal	Recorded Resistances
Enterococcus hirae	Bacterium		VRE
Enterococcus italicus	Bacterium		VRE
Enterococcus malodoratus	Bacterium		VRE
Enterococcus moraviensis	Bacterium		VRE
Enterococcus mundtii	Bacterium		VRE
Enterococcus pallens	Bacterium		VRE
Enterococcus phoeniculicola	Bacterium		VRE
Enterococcus pseudoavium	Bacterium		VRE
Enterococcus raffinosus	Bacterium		VRE
Enterococcus ratti	Bacterium		VRE
Enterococcus saccharolyticus	Bacterium		VRE
Enterococcus saccharominimus	Bacterium (synonym for Enterococcus italicus)		VRE
Enterococcus seriolicida	Bacterium (synonym for Lactococcus garvieae)		
Enterococcus solitarius	Bacterium (synonym for Tetragenococcus solitarius)		
Enterococcus sulfureus	Bacterium		VRE
Enterococcus villorum	Bacterium		VRE
Enterocytozoon	Protozoon		
Enterovirus	Virus		
Enterovirus B	Virus (synonym for Enterovirus betacoxsackie)		
Enterovirus betacoxsackie	Virus		
Enterovirus C	Virus (synonym for Enterovirus coxsackiepol)		
Enterovirus coxsackiepol	Virus		
Entomophthora coronata	Fungus (synonym for Conidiobolus coronatus)		
Epicoccum	Fungus		
Epstein-Barr virus	Virus (synonym for Lymphocryptovirus humangamma4)		
Erwinia	Bacterium		3GCR, Carbapenems, Colistin
Erwinia ananas	Bacterium (synonym for Pantoea ananatis)		3GCR, Carbapenems, Colistin
Erwinia ananatis	Bacterium (synonym for Pantoea ananatis)		3GCR, Carbapenems, Colistin
Erwinia cancerogena	Bacterium (synonym for Enterobacter cancerogenus)		3GCR, Carbapenems, Colistin
Erwinia dissolvens	Bacterium (synonym for Enterobacter cloacae subsp. dissolvens)		3GCR, Carbapenems, Colistin
Erwinia nimipressuralis	Bacterium (synonym for Lelliottia nimipressuralis)		3GCR
Erwinia persicina	Bacterium		3GCR, Carbapenems, Colistin
Erwinia persicinus	Bacterium (synonym for Erwinia persicina)		3GCR, Carbapenems, Colistin
Erwinia uredovora	Bacterium (synonym for Pantoea ananatis)		3GCR, Carbapenems, Colistin
Erysipelothrix	Bacterium		
Erysipelothrix inopinata	Bacterium		
Erysipelothrix rhusiopathiae	Bacterium		
Erysipelothrix tonsillarum	Bacterium		
Erythroparvovirus primate1	Virus		
Erythrovirus B19	Virus (synonym for Erythroparvovirus primate1)		
Escherichia	Bacterium		3GCR, Carbapenems, Colistin
Escherichia adecarboxylata	Bacterium (synonym for Leclercia adecarboxylata)		3GCR, Carbapenems, Colistin
Escherichia albertii	Bacterium		3GCR, Carbapenems, Colistin
Escherichia coli	Bacterium		3GCR, Carbapenems, Colistin
Escherichia fergusonii	Bacterium		3GCR, Carbapenems, Colistin

Name	Type	Common Commensal	Recorded Resistances
Escherichia hermannii	Bacterium		3GCR, Carbapenems, Colistin
Escherichia vulneris	Bacterium (synonym for Pseudescherichia vulneris)		3GCR, Carbapenems, Colistin
Eubacterium	Bacterium		
Eubacterium aerofaciens	Bacterium (synonym for Collinsella aerofaciens)		
Eubacterium alactolyticum	Bacterium (synonym for Pseudoramibacter alactolyticus)		
Eubacterium barkeri	Bacterium		
Eubacterium biforme	Bacterium (synonym for Holdemanella biformis)		
Eubacterium brachy	Bacterium		
Eubacterium budayi	Bacterium (synonym for Clostridium budayi)		
Eubacterium callanderi	Bacterium		
Eubacterium combesii	Bacterium (synonym for Clostridium combesii)		
Eubacterium contortum	Bacterium (synonym for Faecalicatena contorta)		
Eubacterium cylindroides	Bacterium (synonym for Faecalitalea cylindroides)		
Eubacterium dolichum	Bacterium (synonym for Amedibacillus dolichus)		
Eubacterium eligens	Bacterium (synonym for Lachnospira eligens)		
Eubacterium exiguum	Bacterium (synonym for Slackia exigua)		
Eubacterium fossor	Bacterium (synonym for Atopobium fossor)		
Eubacterium hadrum	Bacterium (synonym for Anaerostipes hadrus)		
Eubacterium hallii	Bacterium (synonym for Anaerobutyricum hallii)		
Eubacterium infirmum	Bacterium		
Eubacterium lentum	Bacterium (synonym for Eggerthella lenta)		
Eubacterium limosum	Bacterium		
Eubacterium minutum	Bacterium		
Eubacterium moniliforme	Bacterium (synonym for Clostridium moniliforme)		
Eubacterium nodatum	Bacterium		
Eubacterium plautii	Bacterium (synonym for Flavonifractor plautii)		
Eubacterium ramulus	Bacterium		
Eubacterium rectale	Bacterium (synonym for Agathobacter rectalis)		
Eubacterium saburreum	Bacterium (synonym for Lachnoanaerobaculum saburreum)		
Eubacterium saphenum	Bacterium		
Eubacterium siraeum	Bacterium		
Eubacterium sulci	Bacterium		
Eubacterium tenue	Bacterium		
Eubacterium timidum	Bacterium (synonym for Mogibacterium timidum)		
Eubacterium ventriosum	Bacterium		
Eubacterium yurii	Bacterium		
Evansella clarkii	Bacterium	Yes	
Evansella vedderi	Bacterium	Yes	
Ewingella	Bacterium		3GCR, Carbapenems, Colistin
Ewingella americana	Bacterium		3GCR, Carbapenems, Colistin
Exiguobacterium	Bacterium	Yes	
Exiguobacterium acetylicum	Bacterium	Yes	
Exophiala	Fungus		
Exophiala bergeri	Fungus		
Exophiala dermatitidis	Fungus		

Name	Type	Common Commensal	Recorded Resistances
Exophiala exophialae	Fungus		
Exophiala jeanselmei	Fungus		
Exophiala spinifera	Fungus		
Exophiala werneckii	Fungus (synonym for Hortaea werneckii)		
Exserohilum	Fungus		
Exserohilum rostratum	Fungus		
Ezakiella coagulans	Bacterium		Carbapenems
Facklamia	Bacterium		
Facklamia hominis	Bacterium		
Facklamia ignava	Bacterium		
Facklamia languida	Bacterium		
Facklamia sourekii	Bacterium		
Facklamia tabacinasalis	Bacterium (synonym for Ruoffia tabacinasalis)		
Faecalibacterium	Bacterium		
Faecalibacterium prausnitzii	Bacterium		
Faecalicatena contorta	Bacterium		
Faecalicatena orotica	Bacterium		
Faecalispora sporosphaeroides	Bacterium		
Faecalitalea cylindroides	Bacterium		
Falciformispora senegalensis	Fungus		
Falciformispora tompkinsii	Fungus		
Falcivibrio grandis	Bacterium (synonym for Mobiluncus mulieris)		
Falcivibrio vaginalis	Bacterium (synonym for Mobiluncus holmesii)		
Falsarthrobacter nasiphocae	Bacterium	Yes	
Falsibacillus pallidus	Bacterium	Yes	
Fannyhessea vaginae	Bacterium		
Ferdinandcohnia humi	Bacterium	Yes	
Fictibacillus barbaricus	Bacterium	Yes	
Fictibacillus gelatini	Bacterium	Yes	
Filifactor	Bacterium		
Filifactor alocis	Bacterium		
Filifactor villosus	Bacterium		
Finegoldia	Bacterium		
Finegoldia magna	Bacterium		
Fissuricella filamenta	Fungus (synonym for Trichosporon asteroides)		
Flavimonas oryzihabitans	Bacterium (synonym for Pseudomonas oryzihabitans)		3GCR, Carbapenems, Colistin
Flavivirus	Virus (synonym for Orthoflavivirus)		
Flavobacterium	Bacterium		
Flavobacterium breve	Bacterium (synonym for Empedobacter brevis)		
Flavobacterium devorans	Bacterium		
Flavobacterium gleum	Bacterium (synonym for Chryseobacterium gleum)		
Flavobacterium heparinum	Bacterium (synonym for Pedobacter heparinus)		
Flavobacterium indologenes	Bacterium (synonym for Chryseobacterium indologenes)		
Flavobacterium marinotypicum	Bacterium (synonym for Microbacterium maritypicum)	Yes	
Flavobacterium meningosepticum	Bacterium (synonym for Elizabethkingia meningoseptica)		

Name	Type	Common Commensal	Recorded Resistances
Flavobacterium multivorum	Bacterium (synonym for Sphingobacterium multivorum)		
Flavobacterium odoratum	Bacterium (synonym for Myroides odoratus)		
Flavobacterium spiritivorum	Bacterium (synonym for Sphingobacterium spiritivorum)		
Flavobacterium thalpophilum	Bacterium (synonym for Sphingobacterium thalpophilum)		
Flavobacterium yabuuchiae	Bacterium (synonym for Sphingobacterium spiritivorum)		
Flavonifractor	Bacterium		
Flavonifractor plautii	Bacterium		
Fluoribacter	Bacterium (synonym for Legionella)		
Fluoribacter bozemanae	Bacterium (synonym for Legionella bozemanae)		
Fluoribacter dumoffii	Bacterium (synonym for Legionella dumoffii)		
Fluoribacter gormanii	Bacterium (synonym for Legionella gormanii)		
Fonsecaea	Fungus		
Fonsecaea compacta	Fungus (synonym for Fonsecaea pedrosoi)		
Fonsecaea compactum	Fungus (synonym for Fonsecaea pedrosoi)		
Fonsecaea dermatitidis	Fungus (synonym for Exophiala dermatitidis)		
Fonsecaea pedrosoi	Fungus		
Francisella	Bacterium		
Francisella tularensis	Bacterium		
Fusarium	Fungus		
Fusarium chlamydosporum	Fungus		
Fusarium culmorum	Fungus		
Fusarium dimerum	Fungus (synonym for Bisifusarium dimerum)		
Fusarium graminearum	Fungus		
Fusarium incarnatum	Fungus		
Fusarium javanicum	Fungus		
Fusarium oxysporum	Fungus		
Fusarium poae	Fungus		
Fusarium semitectum	Fungus (synonym for Fusarium incarnatum)		
Fusarium sporotrichioides	Fungus (synonym for Fusarium chlamydosporum)		
Fusarium tricinctum	Fungus		
Fusarium verticillioides	Fungus		
Fusicoccum dimidiatum	Fungus (synonym for Neoscytalidium dimidiatum)		
Fusobacterium	Bacterium		
Fusobacterium alocis	Bacterium (synonym for Filifactor alocis)		
Fusobacterium canifelinum	Bacterium		
Fusobacterium equinum	Bacterium (synonym for Fusobacterium gonidiaformans)		
Fusobacterium gonidiaformans	Bacterium		
Fusobacterium mortiferum	Bacterium		
Fusobacterium naviforme	Bacterium		
Fusobacterium necrogenes	Bacterium		
Fusobacterium necrophorum	Bacterium		
Fusobacterium necrophorum subsp. funduliforme	Bacterium (synonym for Fusobacterium necrophorum)		
Fusobacterium necrophorum subsp. necrophorum	Bacterium		

Name	Type	Common Commensal	Recorded Resistances
Fusobacterium nucleatum	Bacterium		
Fusobacterium nucleatum subsp. fusiforme	Bacterium (synonym for Fusobacterium nucleatum)		
Fusobacterium nucleatum subsp. nucleatum	Bacterium		
Fusobacterium nucleatum subsp. polymorphum	Bacterium		
Fusobacterium periodonticum	Bacterium		
Fusobacterium plauti	Bacterium (synonym for Flavonifractor plautii)		
Fusobacterium plautii	Bacterium (synonym for Flavonifractor plautii)		
Fusobacterium polymorphum	Bacterium (synonym for Fusobacterium nucleatum subsp. polymorphum)		
Fusobacterium prausnitzii	Bacterium (synonym for Faecalibacterium prausnitzii)		
Fusobacterium russii	Bacterium		
Fusobacterium sulci	Bacterium (synonym for Eubacterium sulci)		
Fusobacterium ulcerans	Bacterium		
Fusobacterium varium	Bacterium		
Fusobacterium vincentii	Bacterium (synonym for Fusobacterium nucleatum)		
Gallicola	Bacterium		
Gallicola barnesae	Bacterium		
Gamma-hemolytic streptococci	Bacterium		
Gammainfluenzavirus influenzae	Virus		
Gardnerella	Bacterium		
Gardnerella vaginalis	Bacterium		
Gemella	Bacterium		
Gemella bergeri	Bacterium		
Gemella bergeriae	Bacterium (synonym for Gemella bergeri)		
Gemella haemolysans	Bacterium		
Gemella morbillorum	Bacterium		
Gemella sanguinis	Bacterium		
Geobacillus	Bacterium		
Geobacillus kaustophilus	Bacterium	Yes	
Geobacillus stearothermophilus	Bacterium		
Geobacillus thermocatenulatus	Bacterium	Yes	
Geobacillus thermodenitrificans	Bacterium		
Geobacillus thermoglucosidasius	Bacterium (synonym for Parageobacillus thermoglucosidasius)		
Geotrichum	Fungus		
Geotrichum candidum	Fungus		
Geotrichum klebahnii	Fungus		
Geotrichum penicillatum	Fungus (synonym for Geotrichum klebahnii)		
Gleimia europaea	Bacterium	Yes	
Glenosporella	Fungus		
Gliocladium	Fungus (synonym for Sphaerostilbella)		
Globicatella	Bacterium		
Globicatella sanguinis	Bacterium		
Globicatella sulfidifaciens	Bacterium		
Glutamicibacter arilaitensis	Bacterium	Yes	
Glutamicibacter bergerei	Bacterium	Yes	
Glutamicibacter creatinolyticus	Bacterium	Yes	

Name	Type	Common Commensal	Recorded Resistances
Glutamicibacter nicotianae	Bacterium	Yes	
Glutamicibacter protophormiae	Bacterium	Yes	
Glutamicibacter uratoxydans	Bacterium	Yes	
Gordonia	Bacterium	Yes	
Gordonia bronchialis	Bacterium	Yes	
Gordonia otitidis	Bacterium	Yes	
Gordonia polyisoprenivorans	Bacterium	Yes	
Gordonia rubripertincta	Bacterium	Yes	
Gordonia rubropertinctus	Bacterium (synonym for Gordonia rubripertincta)	Yes	
Gordonia sputi	Bacterium	Yes	
Gordonia terrae	Bacterium	Yes	
Gottfriedia luciferensis	Bacterium	Yes	
Gracilibacillus dipsosauri	Bacterium	Yes	
Granulicatella	Bacterium		
Granulicatella adiacens	Bacterium		
Granulicatella elegans	Bacterium		
Graphium	Fungus		
Grimontia	Bacterium		
Grimontia hollisae	Bacterium		
Group A streptococcus	Bacterium (synonym for Streptococcus pyogenes)		
Group B streptococcus	Bacterium (synonym for Streptococcus agalactiae)		
Haemophilus	Bacterium		3GCR, Carbapenems, Colistin
Haemophilus actinomycetemcomitans	Bacterium (synonym for Aggregatibacter actinomycetemcomitans)		
Haemophilus aegyptius	Bacterium		3GCR, Carbapenems, Colistin
Haemophilus aphrophilus	Bacterium (synonym for Aggregatibacter aphrophilus)		
Haemophilus ducreyi	Bacterium		3GCR, Carbapenems, Colistin
Haemophilus haemoglobinophilus	Bacterium		3GCR, Carbapenems, Colistin
Haemophilus haemolyticus	Bacterium		3GCR, Carbapenems, Colistin
Haemophilus influenzae	Bacterium		3GCR, Carbapenems, Colistin
Haemophilus massiliensis	Bacterium		3GCR, Carbapenems, Colistin
Haemophilus parahaemolyticus	Bacterium		3GCR, Carbapenems, Colistin
Haemophilus parainfluenzae	Bacterium		3GCR, Carbapenems, Colistin
Haemophilus paraphrohaemolyticus	Bacterium		3GCR, Carbapenems, Colistin
Haemophilus pittmaniae	Bacterium		3GCR, Carbapenems, Colistin
Haemophilus segnis	Bacterium (synonym for Aggregatibacter segnis)		
Haemophilus sputorum	Bacterium		3GCR, Carbapenems, Colistin
Hafnia	Bacterium		3GCR, Carbapenems
Hafnia alvei	Bacterium		3GCR, Carbapenems
Hafnia paralvei	Bacterium		3GCR, Carbapenems
Halalkalibacter krulwichiae	Bacterium	Yes	
Halalkalibacterium halodurans	Bacterium	Yes	
Halomonas	Bacterium		
Halomonas venusta	Bacterium		
Halopseudomonas pertucinogena	Bacterium		3GCR, Carbapenems, Colistin
Hansenula polymorpha	Fungus (synonym for Ogataea polymorpha)		
Hathewaya histolytica	Bacterium		

Name	Type	Common Commensal	Recorded Resistances
Hathewaya limosa	Bacterium		
Helcococcus	Bacterium		
Helcococcus kunzii	Bacterium		
Helcococcus sueciensis	Bacterium		
Helicobacter	Bacterium		
Helicobacter acinonychis	Bacterium		
Helicobacter acinonyx	Bacterium (synonym for Helicobacter acinonychis)		
Helicobacter aurati	Bacterium		
Helicobacter bilis	Bacterium		
Helicobacter bizzozeronii	Bacterium		
Helicobacter canadensis	Bacterium		
Helicobacter canis	Bacterium		
Helicobacter cholecystus	Bacterium		
Helicobacter cinaedi	Bacterium		
Helicobacter felis	Bacterium		
Helicobacter fennelliae	Bacterium		
Helicobacter ganmani	Bacterium		
Helicobacter hepaticus	Bacterium		
Helicobacter mesocricetorum	Bacterium		
Helicobacter muridarum	Bacterium		
Helicobacter mustelae	Bacterium		
Helicobacter nemestrinae	Bacterium (synonym for Helicobacter pylori)		
Helicobacter pametensis	Bacterium		
Helicobacter pullorum	Bacterium		
Helicobacter pylori	Bacterium		
Helicobacter rappini	Bacterium		
Helicobacter rodentium	Bacterium		
Helicobacter salomonis	Bacterium		
Helicobacter trogontum	Bacterium		
Helicobacter typhlonius	Bacterium		
Helminthosporium	Fungus		
Helminthosporium halodes	Fungus (synonym for Exserohilum rostratum)		
Helminthosporium rostratum	Fungus (synonym for Exserohilum rostratum)		
Helminthosporium spiciferum	Fungus (synonym for Curvularia spicifera)		
Hepacivirus C	Virus (synonym for Hepacivirus hominis)		
Hepacivirus hominis	Virus		
Hepatitis A virus	Virus (synonym for Hepatovirus ahepa)		
Hepatitis B virus	Virus (synonym for Orthohepadnavirus hominoidei)		
Hepatitis C virus	Virus (synonym for Hepacivirus hominis)		
Hepatitis D virus	Virus (synonym for Deltavirus)		
Hepatitis E virus	Virus (synonym for Paslahepevirus balayani)		
Hepatitis G virus	Virus (synonym for Pegivirus hominis)		
Hepatovirus A	Virus (synonym for Hepatovirus ahepa)		
Hepatovirus ahepa	Virus		
Herbaspirillum	Bacterium		
Herpes simplex virus type 1	Virus (synonym for Simplexvirus humanalpha1)		
Herpes simplex virus type 2	Virus (synonym for Simplexvirus humanalpha2)		

Name	Type	Common Commensal	Recorded Resistances
Herpesviridae	Virus (synonym for Orthoherpesviridae)		
Heyndrickxia oleronia	Bacterium	Yes	
Heyndrickxia shackletonii	Bacterium		
Heyndrickxia sporothermodurans	Bacterium	Yes	
Holdemanella biformis	Bacterium		
Holdemania	Bacterium		
Holdemania filiformis	Bacterium		
Hormiscium dermatitidis	Fungus (synonym for Exophiala dermatitidis)		
Hormodendrum	Fungus		
Hormodendrum dermatitidis	Fungus (synonym for Exophiala dermatitidis)		
Hormonema	Fungus (synonym for Sydowia)		
Hortaea werneckii	Fungus		
Human adenovirus	Virus		
Human alphaherpesvirus 1	Virus (synonym for Simplexvirus humanalpha1)		
Human alphaherpesvirus 2	Virus (synonym for Simplexvirus humanalpha2)		
Human alphaherpesvirus 3	Virus (synonym for Varicellovirus humanalpha3)		
Human betaherpesvirus 5	Virus (synonym for Cytomegalovirus humanbeta5)		
Human betaherpesvirus 6A	Virus (synonym for Roseolovirus humanbeta6a)		
Human betaherpesvirus 6B	Virus (synonym for Roseolovirus humanbeta6b)		
Human bocavirus	Virus		
Human coronavirus	Virus		
Human coxsackievirus	Virus		
Human coxsackievirus A	Virus		
Human coxsackievirus B	Virus		
Human cytomegalovirus	Virus (synonym for Cytomegalovirus humanbeta5)		
Human gammaherpesvirus 4	Virus (synonym for Lymphocryptovirus humangamma4)		
Human gammaherpesvirus 8	Virus (synonym for Rhadinovirus humangamma8)		
Human herpes simplex virus	Virus		
Human herpesvirus 6	Virus		
Human herpesvirus 6A	Virus (synonym for Roseolovirus humanbeta6a)		
Human herpesvirus 6B	Virus (synonym for Roseolovirus humanbeta6b)		
Human herpesvirus 8	Virus (synonym for Rhadinovirus humangamma8)		
Human immunodeficiency virus	Virus		
Human immunodeficiency virus 1	Virus (synonym for Lentivirus humimdef1)		
Human immunodeficiency virus 2	Virus (synonym for Lentivirus humimdef2)		
Human metapneumovirus	Virus (synonym for Metapneumovirus hominis)		
Human orthopneumovirus	Virus (synonym for Orthopneumovirus hominis)		
Human orthorubulavirus 2	Virus (synonym for Orthorubulavirus laryngotracheitidis)		
Human orthorubulavirus 4	Virus (synonym for Orthorubulavirus hominis)		
Human papillomavirus	Virus		
Human parainfluenza virus	Virus		
Human parainfluenza virus 1	Virus (synonym for Respirovirus laryngotracheitidis)		
Human parainfluenza virus 2	Virus (synonym for Orthorubulavirus laryngotracheitidis)		
Human parainfluenza virus 3	Virus (synonym for Respirovirus pneumoniae)		
Human parainfluenza virus 4	Virus (synonym for Orthorubulavirus hominis)		

Name	Type	Common Commensal	Recorded Resistances
Human pegivirus	Virus		
Human polyomavirus 1	Virus (synonym for Betapolyomavirus hominis)		
Human polyomavirus 2	Virus (synonym for Betapolyomavirus secuhominis)		
Human respiratory syncytial virus	Virus (synonym for Orthopneumovirus hominis)		
Human respirovirus 1	Virus (synonym for Respirovirus laryngotracheitidis)		
Human respirovirus 3	Virus (synonym for Respirovirus pneumoniae)		
Human rhinovirus	Virus		
Human T-lymphotropic virus 1	Virus (synonym for Deltaretrovirus priTlym1)		
Human T-lymphotropic virus 2	Virus (synonym for Deltaretrovirus priTlym2)		
Hungatella hathewayi	Bacterium		
Hypomyces	Fungus		
Ignavigranum	Bacterium		
Ignavigranum ruoffiae	Bacterium		
Influenza A virus	Virus (synonym for Alphainfluenzavirus influenzae)		
Influenza B virus	Virus (synonym for Betainfluenzavirus influenzae)		
Influenza C virus	Virus (synonym for Gammainfluenzavirus influenzae)		
Influenza D virus	Virus (synonym for Deltainfluenzavirus influenzae)		
Influenza virus	Virus		
Inquilinus	Bacterium		
Inquilinus limosus	Bacterium		
Intestinibacter bartlettii	Bacterium		
Janibacter	Bacterium	Yes	
Janibacter hoylei	Bacterium	Yes	
JC polyomavirus	Virus (synonym for Betapolyomavirus secuhominis)		
JC virus	Virus (synonym for Betapolyomavirus secuhominis)		
Jeotgalibacillus marinus	Bacterium	Yes	
Jonesia	Bacterium		
Jonesia denitrificans	Bacterium		
Kandleria vitulina	Bacterium		
Kaposi's sarcoma-associated herpesvirus	Virus (synonym for Rhadinovirus humangamma8)		
Kerstersia	Bacterium		
Kerstersia gyiorum	Bacterium		
Kingella	Bacterium		
Kingella denitrificans	Bacterium		
Kingella indologenes	Bacterium (synonym for Suttonella indologenes)		
Kingella kingae	Bacterium		
Kingella oralis	Bacterium		
Kingella potus	Bacterium		
Klebsiella	Bacterium		3GCR, Carbapenems, Colistin
Klebsiella aerogenes	Bacterium		3GCR, Carbapenems, Colistin
Klebsiella granulomatis	Bacterium		3GCR, Carbapenems, Colistin
Klebsiella grimontii	Bacterium		3GCR, Carbapenems, Colistin
Klebsiella ornithinolytica	Bacterium		3GCR, Carbapenems
Klebsiella oxytoca	Bacterium		3GCR, Carbapenems, Colistin
Klebsiella ozaenae	Bacterium (synonym for Klebsiella pneumoniae subsp. ozaenae)		3GCR, Carbapenems, Colistin
Klebsiella pasteurii	Bacterium		3GCR, Carbapenems, Colistin

Name	Type	Common Commensal	Recorded Resistances
Klebsiella planticola	Bacterium		3GCR, Carbapenems
Klebsiella pneumoniae	Bacterium		3GCR, Carbapenems, Colistin
Klebsiella pneumoniae subsp. ozaenae	Bacterium		3GCR, Carbapenems, Colistin
Klebsiella pneumoniae subsp. pneumoniae	Bacterium		3GCR, Carbapenems, Colistin
Klebsiella pneumoniae subsp. rhinoscleromatis	Bacterium		3GCR, Carbapenems, Colistin
Klebsiella quasipneumoniae	Bacterium		3GCR, Carbapenems, Colistin
Klebsiella quasipneumoniae subsp. quasipneumoniae	Bacterium		3GCR, Carbapenems, Colistin
Klebsiella quasipneumoniae subsp. similipneumoniae	Bacterium		3GCR, Carbapenems, Colistin
Klebsiella rhinoscleromatis	Bacterium (synonym for Klebsiella pneumoniae subsp. rhinoscleromatis)		3GCR, Carbapenems, Colistin
Klebsiella singaporensis	Bacterium (synonym for Klebsiella variicola)		3GCR, Carbapenems, Colistin
Klebsiella terrigena	Bacterium		3GCR, Carbapenems
Klebsiella trevisanii	Bacterium (synonym for Klebsiella planticola)		3GCR, Carbapenems
Klebsiella variicola	Bacterium		3GCR, Carbapenems, Colistin
Kluyvera	Bacterium		3GCR, Carbapenems, Colistin
Kluyvera ascorbata	Bacterium		3GCR, Carbapenems, Colistin
Kluyvera cryocrescens	Bacterium		3GCR, Carbapenems, Colistin
Kluyvera georgiana	Bacterium		3GCR, Carbapenems, Colistin
Kluyvera intermedia	Bacterium		3GCR, Carbapenems, Colistin
Kocuria	Bacterium	Yes	
Kocuria erythromyxa	Bacterium (synonym for Kocuria rosea)	Yes	
Kocuria kristinae	Bacterium (synonym for Rothia kristinae)	Yes	
Kocuria rosea	Bacterium	Yes	
Kocuria varians	Bacterium	Yes	
Kodamaea	Fungus		
Kodamaea ohmeri	Fungus		
Kosakonia	Bacterium		3GCR
Kosakonia cowanii	Bacterium		3GCR
Koserella trabulsii	Bacterium (synonym for Yokenella regensburgei)		3GCR, Carbapenems, Colistin
Kroppenstedtia	Bacterium		
Kroppenstedtia eburnea	Bacterium		
Kurthia	Bacterium		
Kyrpidia tusciae	Bacterium	Yes	
Kytococcus	Bacterium	Yes	
Kytococcus sedentarius	Bacterium	Yes	
Lachnoanaerobaculum	Bacterium		
Lachnoanaerobaculum orale	Bacterium		
Lachnoanaerobaculum saburreum	Bacterium		
Lachnospira eligens	Bacterium		
Lacrimispora amygdalina	Bacterium		
Lacrimispora celerecrescens	Bacterium		
Lacrimispora indolis	Bacterium		
Lacrimispora sphenoides	Bacterium		
Lacticaseibacillus casei	Bacterium		
Lacticaseibacillus paracasei	Bacterium		
Lacticaseibacillus rhamnosus	Bacterium (synonym for Lactobacillus rhamnosus)		
Lactiplantibacillus plantarum	Bacterium		

Name	Type	Common Commensal	Recorded Resistances
Lactobacillus	Bacterium		
Lactobacillus acidophilus	Bacterium		
Lactobacillus amylovorus	Bacterium		
Lactobacillus antri	Bacterium (synonym for Limosilactobacillus antri)		
Lactobacillus arizonensis	Bacterium (synonym for Lactiplantibacillus plantarum)		
Lactobacillus brevis	Bacterium (synonym for Levilactobacillus brevis)		
Lactobacillus buchneri	Bacterium (synonym for Lentilactobacillus buchneri)		
Lactobacillus casei	Bacterium (synonym for Lacticaseibacillus casei)		
Lactobacillus catenaformis	Bacterium (synonym for Eggerthia catenaformis)		
Lactobacillus cellobiosus	Bacterium (synonym for Limosilactobacillus fermentum)		
Lactobacillus confusus	Bacterium (synonym for Weissella confusa)		
Lactobacillus crispatus	Bacterium		
Lactobacillus delbrueckii	Bacterium		
Lactobacillus fermentum	Bacterium (synonym for Limosilactobacillus fermentum)		
Lactobacillus gasseri	Bacterium		
Lactobacillus gastricus	Bacterium (synonym for Limosilactobacillus gastricus)		
Lactobacillus iners	Bacterium		
Lactobacillus jensenii	Bacterium		
Lactobacillus johnsonii	Bacterium		
Lactobacillus kalixensis	Bacterium		
Lactobacillus minutus	Bacterium (synonym for Atopobium minutum)		
Lactobacillus oris	Bacterium (synonym for Limosilactobacillus oris)		
Lactobacillus paracasei	Bacterium (synonym for Lacticaseibacillus paracasei)		
Lactobacillus plantarum	Bacterium (synonym for Lactiplantibacillus plantarum)		
Lactobacillus reuteri	Bacterium (synonym for Limosilactobacillus reuteri)		
Lactobacillus rhamnosus	Bacterium		
Lactobacillus rimae	Bacterium (synonym for Lancefieldella rimae)		Carbapenems
Lactobacillus sakei	Bacterium (synonym for Latilactobacillus sakei)		
Lactobacillus salivarius	Bacterium (synonym for Ligilactobacillus salivarius)		
Lactobacillus ultunensis	Bacterium		
Lactobacillus vaginalis	Bacterium (synonym for Limosilactobacillus vaginalis)		
Lactobacillus vitulinus	Bacterium (synonym for Kandleria vitulina)		
Lactococcus	Bacterium		
Lactococcus garvieae	Bacterium		
Lactococcus lactis	Bacterium		
Lancefieldella parvula	Bacterium		Carbapenems
Lancefieldella rimae	Bacterium		Carbapenems
Lasiodiplodia	Fungus		
Lasiodiplodia theobromae	Fungus		
Lassa mammarenavirus	Virus (synonym for Mammarenavirus lassaense)		
Lassa virus	Virus (synonym for Mammarenavirus lassaense)		
Latilactobacillus sakei	Bacterium		
Leclercia	Bacterium		3GCR, Carbapenems, Colistin
Leclercia adecarboxylata	Bacterium		3GCR, Carbapenems, Colistin
Lecythophora	Fungus (synonym for Coniochaeta)		

Name	Type	Common Commensal	Recorded Resistances
Lecythophora hoffmannii	Fungus (synonym for Coniochaeta hoffmannii)		
Lecythophora mutabilis	Fungus (synonym for Coniochaeta mutabilis)		
Lederbergia galactosidilytica	Bacterium	Yes	
Lederbergia lenta	Bacterium	Yes	
Legionella	Bacterium		
Legionella adelaidensis	Bacterium		
Legionella anisa	Bacterium		
Legionella beliardensis	Bacterium		
Legionella birminghamensis	Bacterium		
Legionella bozemanae	Bacterium		
Legionella bozemanii	Bacterium (synonym for Legionella bozemanae)		
Legionella brunensis	Bacterium		
Legionella busanensis	Bacterium		
Legionella cardiaca	Bacterium		
Legionella cherrii	Bacterium		
Legionella cincinnatiensis	Bacterium		
Legionella drancourtii	Bacterium		
Legionella drozanskii	Bacterium		
Legionella dumoffii	Bacterium		
Legionella erythra	Bacterium		
Legionella fairfieldensis	Bacterium		
Legionella fallonii	Bacterium		
Legionella feeleii	Bacterium		
Legionella geestiana	Bacterium		
Legionella gormanii	Bacterium		
Legionella hackeliae	Bacterium		
Legionella indianapolisensis	Bacterium		
Legionella israelensis	Bacterium		
Legionella jamestowniensis	Bacterium		
Legionella jordanis	Bacterium		
Legionella lansingensis	Bacterium		
Legionella londiniensis	Bacterium		
Legionella longbeachae	Bacterium		
Legionella lytica	Bacterium		
Legionella maceachernii	Bacterium		
Legionella micdadei	Bacterium		
Legionella moravica	Bacterium		
Legionella nagasakiensis	Bacterium		
Legionella nautarum	Bacterium		
Legionella oakridgensis	Bacterium		
Legionella parisiensis	Bacterium		
Legionella pittsburghensis	Bacterium (synonym for Legionella micdadei)		
Legionella pneumophila	Bacterium		
Legionella quateirensis	Bacterium		
Legionella quinlivanii	Bacterium		
Legionella rowbothamii	Bacterium		
Legionella rubrilucens	Bacterium		

Name	Type	Common Commensal	Recorded Resistances
Legionella sainthelensi	Bacterium		
Legionella santicrucis	Bacterium		
Legionella shakespearei	Bacterium		
Legionella spiritensis	Bacterium		
Legionella steelei	Bacterium		
Legionella steigerwaltii	Bacterium		
Legionella taurinensis	Bacterium		
Legionella tucsonensis	Bacterium		
Legionella wadsworthii	Bacterium		
Legionella waltersii	Bacterium		
Legionella worsleiensis	Bacterium		
Leifsonia	Bacterium	Yes	
Leifsonia aquatica	Bacterium	Yes	
Leifsonia xyli	Bacterium	Yes	
Lelliottia	Bacterium		3GCR
Lelliottia amnigena	Bacterium		3GCR
Lelliottia nimipressuralis	Bacterium		3GCR
Leminorella	Bacterium		3GCR, Carbapenems, Colistin
Leminorella grimontii	Bacterium		3GCR, Carbapenems, Colistin
Leminorella richardii	Bacterium		3GCR, Carbapenems, Colistin
Lentilactobacillus buchneri	Bacterium		
Lentivirus humimdef1	Virus		
Lentivirus humimdef2	Virus		
Leptosphaeria	Fungus		
Leptosphaeria senegalensis	Fungus (synonym for Falciformispora senegalensis)		
Leptosphaeria tompkinsii	Fungus (synonym for Falciformispora tompkinsii)		
Leptospira	Bacterium		
Leptospira interrogans	Bacterium		
Leptotrichia	Bacterium		
Leptotrichia buccalis	Bacterium		
Leptotrichia goodfellowii	Bacterium (synonym for Pseudoleptotrichia goodfellowii)		
Leptotrichia trevisanii	Bacterium		
Leptotrichia wadei	Bacterium		
Leuconostoc	Bacterium		
Leuconostoc amelibiosum	Bacterium (synonym for Leuconostoc citreum)		
Leuconostoc citreum	Bacterium		
Leuconostoc lactis	Bacterium		
Leuconostoc mesenteroides	Bacterium		
Leuconostoc pseudomesenteroides	Bacterium		
Levilactobacillus brevis	Bacterium		
Levinea amalonatica	Bacterium (synonym for Citrobacter amalonaticus)		3GCR, Carbapenems, Colistin
Ligilactobacillus salivarius	Bacterium		
Limosilactobacillus antri	Bacterium		
Limosilactobacillus fermentum	Bacterium		
Limosilactobacillus gastricus	Bacterium		
Limosilactobacillus oris	Bacterium		

Name	Type	Common Commensal	Recorded Resistances
Limosilactobacillus reuteri	Bacterium		
Limosilactobacillus vaginalis	Bacterium		
Listeria	Bacterium		
Listeria denitrificans	Bacterium (synonym for Jonesia denitrificans)		
Listeria grayi	Bacterium		
Listeria innocua	Bacterium		
Listeria ivanovii	Bacterium		
Listeria monocytogenes	Bacterium		
Listeria seeligeri	Bacterium		
Listeria welshimeri	Bacterium		
Listonella	Bacterium		
Loboa	Fungus		
Lodderomyces	Fungus		
Lodderomyces elongisporus	Fungus		
Lomentospora prolificans	Fungus		
Lucibacterium harveyi	Bacterium (synonym for Vibrio harveyi)		Colistin
Lymphocryptovirus humangamma4	Virus		
Lymphocytic choriomeningitis mammarenavirus	Virus (synonym for Mammarenavirus choriomeningitidis)		
Lymphocytic choriomeningitis virus	Virus (synonym for Mammarenavirus choriomeningitidis)		
Lysinibacillus odysseyi	Bacterium	Yes	
Lysinibacillus sphaericus	Bacterium	Yes	
Lyssavirus rabies	Virus		
Macrococcus	Bacterium		
Macrococcus bovicus	Bacterium		
Macrococcus carouselicus	Bacterium		
Macrococcus caseolyticus	Bacterium		
Macrococcus equipercicus	Bacterium		
Macrosporium	Fungus (synonym for Alternaria)		
Madariaga virus	Virus (synonym for Alphavirus madariaga)		
Madurella	Fungus		
Madurella grisea	Fungus (synonym for Trematosphaeria grisea)		
Madurella mycetomatis	Fungus		
Madurella mycetomi	Fungus (synonym for Madurella mycetomatis)		
Magnusiomyces	Fungus		
Malacoplasma penetrans	Bacterium		
Malassezia	Fungus		
Malassezia furfur	Fungus		
Malassezia globosa	Fungus		
Malassezia pachydermatis	Fungus		
Malassezia restricta	Fungus		
Malassezia sympodialis	Fungus		
Malbranchea	Fungus		
Mammaliicoccus fleurettii	Bacterium (synonym for Staphylococcus fleurettii)	Yes	
Mammaliicoccus lentus	Bacterium (synonym for Staphylococcus lentus)	Yes	
Mammaliicoccus sciuri	Bacterium (synonym for Staphylococcus sciuri)	Yes	

Name	Type	Common Commensal	Recorded Resistances
Mammaliicoccus vitulinus	Bacterium (synonym for Staphylococcus vitulinus)	Yes	
Mammarenavirus choriomeningitidis	Virus		
Mammarenavirus lassaense	Virus		
Mannheimia	Bacterium		
Mannheimia haemolytica	Bacterium		
Marburgvirus	Virus (synonym for Orthomarburgvirus)		
Margalitia shackletonii	Bacterium (synonym for Heyndrickxia shackletonii)		
Marquandomyces marquandii	Fungus		
Massilia	Bacterium		
Massilia timonae	Bacterium		
Mastadenovirus	Virus		
Measles morbillivirus	Virus (synonym for Morbillivirus hominis)		
Measles virus	Virus (synonym for Morbillivirus hominis)		
Medicopsis romeroi	Fungus		
Mediterraneibacter gnavus	Bacterium		
Megamonas	Bacterium		
Megamonas hypermegale	Bacterium		
Megamonas hypermegas	Bacterium (synonym for Megamonas hypermegale)		
Megasphaera	Bacterium		
Megasphaera elsdenii	Bacterium		
Mesobacillus boroniphilus	Bacterium	Yes	
Mesobacillus jeotgali	Bacterium	Yes	
Mesobacillus subterraneus	Bacterium	Yes	
Metabacillus fastidiosus	Bacterium	Yes	
Metabacillus idriensis	Bacterium	Yes	
Metabacillus indicus	Bacterium	Yes	
Metamycoplasma buccale	Bacterium		
Metamycoplasma faucium	Bacterium		
Metamycoplasma hominis	Bacterium		
Metamycoplasma orale	Bacterium		
Metamycoplasma salivarium	Bacterium		
Metapneumovirus hominis	Virus		
Metarhizium	Fungus		
Metarhizium anisopliae	Fungus		
Methylobacterium	Bacterium		
Methylobacterium mesophilicum	Bacterium		
Methylobacterium zatmanii	Bacterium (synonym for Methylorubrum zatmanii)		
Methylorubrum zatmanii	Bacterium		
Metschnikowiaceae	Fungus		
Meyerozyma	Fungus		
Meyerozyma guilliermondii	Fungus		
Microascus paisii	Fungus		
Microbacterium	Bacterium	Yes	
Microbacterium arborescens	Bacterium	Yes	
Microbacterium hydrocarbonoxydans	Bacterium	Yes	
Microbacterium imperiale	Bacterium	Yes	
Microbacterium lacticum	Bacterium	Yes	

Name	Type	Common Commensal	Recorded Resistances
Microbacterium liquefaciens	Bacterium	Yes	
Microbacterium maritypicum	Bacterium	Yes	
Microbacterium oxydans	Bacterium	Yes	
Microbacterium paraoxydans	Bacterium	Yes	
Microbacterium resistens	Bacterium	Yes	
Micrococcus	Bacterium	Yes	
Micrococcus antarcticus	Bacterium	Yes	
Micrococcus flavus	Bacterium	Yes	
Micrococcus halobius	Bacterium (synonym for Nesterenkonia halobia)		
Micrococcus kristinae	Bacterium (synonym for Rothia kristinae)	Yes	
Micrococcus luteus	Bacterium	Yes	
Micrococcus lylae	Bacterium	Yes	
Micrococcus nishinomiyaensis	Bacterium (synonym for Dermacoccus nishinomiyaensis)	Yes	
Micrococcus roseus	Bacterium (synonym for Kocuria rosea)	Yes	
Micrococcus sedentarius	Bacterium (synonym for Kytococcus sedentarius)	Yes	
Micromonospora	Bacterium		
Micromonospora chalcea	Bacterium		
Micropolyspora brevicatena	Bacterium (synonym for Nocardia brevicatena)		
Microsporidium	Protozoon		
Microsporon brachytomum	Fungus		
Microsporum	Fungus		
Microsporum brachytomum	Fungus (synonym for Microsporon brachytomum)		
Microsporum canis	Fungus		
Millerozyma farinosa	Fungus		
Mitsuokella	Bacterium		
Mitsuokella dentalis	Bacterium (synonym for Prevotella dentalis)		
Mitsuokella multacida	Bacterium		
Mixta calida	Bacterium		3GCR, Carbapenems, Colistin
Mobiluncus	Bacterium		
Mobiluncus curtisii	Bacterium		
Mobiluncus holmesii	Bacterium		
Mobiluncus mulieris	Bacterium		
Moellerella	Bacterium		3GCR, Carbapenems, Colistin
Moellerella wisconsensis	Bacterium		3GCR, Carbapenems, Colistin
Moesziomyces bullatus	Fungus		
Mogibacterium	Bacterium		
Mogibacterium diversum	Bacterium		
Mogibacterium neglectum	Bacterium		
Mogibacterium timidum	Bacterium		
Mogibacterium vescum	Bacterium		
Molluscipoxvirus molluscum	Virus		
Molluscum contagiosum virus	Virus (synonym for Molluscipoxvirus molluscum)		
Monkeypox virus	Virus (synonym for Orthopoxvirus monkeypox)		
Monosporium apiospermum	Fungus (synonym for Scedosporium apiospermum)		
Moraxella	Bacterium		
Moraxella anatipestifer	Bacterium (synonym for Riemerella anatipestifera)		

Name	Type	Common Commensal	Recorded Resistances
Moraxella anatipestifera	Bacterium (synonym for Riemerella anatipestifera)		
Moraxella atlantae	Bacterium		
Moraxella boevrei	Bacterium		
Moraxella bovis	Bacterium		
Moraxella canis	Bacterium		
Moraxella caprae	Bacterium		
Moraxella catarrhalis	Bacterium		
Moraxella cuniculi	Bacterium		
Moraxella lacunata	Bacterium		
Moraxella lincolnii	Bacterium		
Moraxella nonliquefaciens	Bacterium		
Moraxella oblonga	Bacterium		
Moraxella osloensis	Bacterium		
Moraxella ovis	Bacterium		
Moraxella phenylpyruvica	Bacterium (synonym for Psychrobacter phenylpyruvicus)		
Moraxella urethralis	Bacterium (synonym for Oligella urethralis)		
Morbillivirus	Virus		
Morbillivirus hominis	Virus		
Morganella	Bacterium		3GCR, Carbapenems
Morganella morganii	Bacterium		3GCR, Carbapenems
Morganella morganii subsp. sibonii	Bacterium		3GCR, Carbapenems
Mortierella	Fungus		
Mortierella wolfii	Fungus (synonym for Actinomortierella wolfii)		
Mucor	Fungus		
Mucor circinelloides	Fungus		
Mucor hiemalis	Fungus		
Mucor janssenii	Fungus		
Mucor javanicus	Fungus (synonym for Mucor circinelloides)		
Mucor pusillus	Fungus (synonym for Rhizomucor pusillus)		
Mucor ramosissimus	Fungus		
Mumps orthorubulavirus	Virus (synonym for Orthorubulavirus parotitidis)		
Mumps virus	Virus (synonym for Orthorubulavirus parotitidis)		
Mycobacterium	Bacterium		
Mycobacterium abscessus	Bacterium		
Mycobacterium abscessus subsp. massiliense	Bacterium		
Mycobacterium africanum	Bacterium (synonym for Mycobacterium tuberculosis)		
Mycobacterium agri	Bacterium		
Mycobacterium aichiense	Bacterium		
Mycobacterium alsense	Bacterium		
Mycobacterium alvei	Bacterium		
Mycobacterium arosiense	Bacterium		
Mycobacterium arupense	Bacterium		
Mycobacterium asiaticum	Bacterium		
Mycobacterium aubagnense	Bacterium		
Mycobacterium aurum	Bacterium		
Mycobacterium austroafricanum	Bacterium		

Name	Type	Common Commensal	Recorded Resistances
Mycobacterium avium	Bacterium		
Mycobacterium avium complex	Bacterium		
Mycobacterium avium subsp. paratuberculosis	Bacterium		
Mycobacterium bacteremicum	Bacterium		
Mycobacterium basiliense	Bacterium		
Mycobacterium boenickei	Bacterium		
Mycobacterium bohemicum	Bacterium		
Mycobacterium bouchedurhonense	Bacterium (synonym for Mycobacterium avium)		
Mycobacterium bovis	Bacterium (synonym for Mycobacterium tuberculosis)		
Mycobacterium branderi	Bacterium		
Mycobacterium brisbanense	Bacterium		
Mycobacterium brumae	Bacterium		
Mycobacterium canariasense	Bacterium		
Mycobacterium canetti	Bacterium (synonym for Mycobacterium tuberculosis subsp. canetti)		
Mycobacterium caprae	Bacterium (synonym for Mycobacterium tuberculosis)		
Mycobacterium celatum	Bacterium		
Mycobacterium chelonae	Bacterium		
Mycobacterium chesapeaki	Bacterium		
Mycobacterium chimaera	Bacterium (synonym for Mycobacterium intracellulare subsp. chimaera)		
Mycobacterium chitae	Bacterium		
Mycobacterium chlorophenolicum	Bacterium		
Mycobacterium chubuense	Bacterium		
Mycobacterium colombiense	Bacterium		
Mycobacterium conceptionense	Bacterium		
Mycobacterium confluentis	Bacterium		
Mycobacterium conspicuum	Bacterium		
Mycobacterium cookii	Bacterium		
Mycobacterium cosmeticum	Bacterium		
Mycobacterium diernhoferi	Bacterium		
Mycobacterium doricum	Bacterium		
Mycobacterium duvalii	Bacterium		
Mycobacterium elephantis	Bacterium		
Mycobacterium europaeum	Bacterium		
Mycobacterium fallax	Bacterium		
Mycobacterium farcinogenes	Bacterium		
Mycobacterium flavescens	Bacterium		
Mycobacterium florentinum	Bacterium		
Mycobacterium fortuitum	Bacterium		
Mycobacterium fortuitum complex	Bacterium		
Mycobacterium fragae	Bacterium		
Mycobacterium frederiksbergense	Bacterium		
Mycobacterium gadium	Bacterium		
Mycobacterium gastri	Bacterium		
Mycobacterium genavense	Bacterium		
Mycobacterium gilvum	Bacterium		

Name	Type	Common Commensal	Recorded Resistances
Mycobacterium goodii	Bacterium		
Mycobacterium gordonae	Bacterium		
Mycobacterium grossiae	Bacterium		
Mycobacterium haemophilum	Bacterium		
Mycobacterium hassiacum	Bacterium		
Mycobacterium heckeshornense	Bacterium		
Mycobacterium heidelbergense	Bacterium		
Mycobacterium heraklionense	Bacterium		
Mycobacterium hiberniae	Bacterium		
Mycobacterium hodleri	Bacterium		
Mycobacterium holsaticum	Bacterium		
Mycobacterium houstonense	Bacterium		
Mycobacterium immunogenum	Bacterium		
Mycobacterium interjectum	Bacterium		
Mycobacterium intermedium	Bacterium		
Mycobacterium intracellulare	Bacterium		
Mycobacterium intracellulare subsp. chimaera	Bacterium		
Mycobacterium iranicum	Bacterium		
Mycobacterium kansasii	Bacterium		
Mycobacterium komossense	Bacterium		
Mycobacterium koreense	Bacterium		
Mycobacterium kubicae	Bacterium		
Mycobacterium kumamotonense	Bacterium		
Mycobacterium kyorinense	Bacterium		
Mycobacterium lacus	Bacterium		
Mycobacterium lentiflavum	Bacterium		
Mycobacterium leprae	Bacterium		
Mycobacterium lepromatosis	Bacterium		
Mycobacterium llatzerense	Bacterium		
Mycobacterium longobardum	Bacterium		
Mycobacterium madagascariense	Bacterium		
Mycobacterium mageritense	Bacterium		
Mycobacterium malmoense	Bacterium		
Mycobacterium mantenii	Bacterium		
Mycobacterium marinum	Bacterium		
Mycobacterium marseillense	Bacterium		
Mycobacterium massiliense	Bacterium (synonym for Mycobacterium abscessus subsp. massiliense)		
Mycobacterium microti	Bacterium (synonym for Mycobacterium tuberculosis)		
Mycobacterium monacense	Bacterium		
Mycobacterium montefiorense	Bacterium		
Mycobacterium moriokaense	Bacterium		
Mycobacterium mucogenicum	Bacterium		
Mycobacterium mungi	Bacterium		
Mycobacterium murale	Bacterium		
Mycobacterium nebraskense	Bacterium		
Mycobacterium neoaurum	Bacterium		

Name	Type	Common Commensal	Recorded Resistances
Mycobacterium neworleansense	Bacterium		
Mycobacterium nonchromogenicum	Bacterium		
Mycobacterium noviomagense	Bacterium		
Mycobacterium novocastrense	Bacterium		
Mycobacterium obuense	Bacterium		
Mycobacterium palustre	Bacterium		
Mycobacterium paraffinicum	Bacterium		
Mycobacterium parafortuitum	Bacterium		
Mycobacterium paragordonae	Bacterium		
Mycobacterium parakoreense	Bacterium		
Mycobacterium parascrofulaceum	Bacterium		
Mycobacterium paraseoulense	Bacterium		
Mycobacterium paratuberculosis	Bacterium (synonym for Mycobacterium avium subsp. paratuberculosis)		
Mycobacterium parmense	Bacterium		
Mycobacterium peregrinum	Bacterium		
Mycobacterium phlei	Bacterium		
Mycobacterium phocaicum	Bacterium		
Mycobacterium pinnipedii	Bacterium (synonym for Mycobacterium tuberculosis)		
Mycobacterium porcinum	Bacterium		
Mycobacterium poriferae	Bacterium		
Mycobacterium psychrotolerans	Bacterium		
Mycobacterium rhodesiae	Bacterium		
Mycobacterium riyadhense	Bacterium		
Mycobacterium saskatchewanense	Bacterium		
Mycobacterium scrofulaceum	Bacterium		
Mycobacterium senegalense	Bacterium		
Mycobacterium senuense	Bacterium		
Mycobacterium seoulense	Bacterium		
Mycobacterium septicum	Bacterium		
Mycobacterium setense	Bacterium		
Mycobacterium sherrisii	Bacterium		
Mycobacterium shimoidei	Bacterium		
Mycobacterium shinjukuense	Bacterium		
Mycobacterium shottsii	Bacterium		
Mycobacterium simiae	Bacterium		
Mycobacterium smegmatis	Bacterium		
Mycobacterium sphagni	Bacterium		
Mycobacterium szulgai	Bacterium		
Mycobacterium terrae	Bacterium		
Mycobacterium terrae complex	Bacterium		
Mycobacterium thermoresistibile	Bacterium		
Mycobacterium timonense	Bacterium		
Mycobacterium tokaiense	Bacterium		
Mycobacterium triplex	Bacterium		
Mycobacterium triviale	Bacterium		
Mycobacterium tuberculosis	Bacterium		

Name	Type	Common Commensal	Recorded Resistances
Mycobacterium tuberculosis complex	Bacterium		
Mycobacterium tuberculosis subsp. canetti	Bacterium		
Mycobacterium tusciae	Bacterium		
Mycobacterium ulcerans	Bacterium		
Mycobacterium vaccae	Bacterium		
Mycobacterium vanbaalenii	Bacterium (synonym for Mycobacterium austroafricanum)		
Mycobacterium wolinskyi	Bacterium		
Mycobacterium xenopi	Bacterium		
Mycoplasma	Bacterium		
Mycoplasma buccale	Bacterium (synonym for Metamycoplasma buccale)		
Mycoplasma faucium	Bacterium (synonym for Metamycoplasma faucium)		
Mycoplasma fermentans	Bacterium (synonym for Mycoplasmopsis fermentans)		
Mycoplasma genitalium	Bacterium (synonym for Mycoplasmoides genitalium)		
Mycoplasma hominis	Bacterium (synonym for Metamycoplasma hominis)		
Mycoplasma lipophilum	Bacterium (synonym for Mycoplasmopsis lipophila)		
Mycoplasma orale	Bacterium (synonym for Metamycoplasma orale)		
Mycoplasma penetrans	Bacterium (synonym for Malacoplasma penetrans)		
Mycoplasma pirum	Bacterium (synonym for Mycoplasmoides pirum)		
Mycoplasma pneumoniae	Bacterium (synonym for Mycoplasmoides pneumoniae)		
Mycoplasma primatum	Bacterium (synonym for Mycoplasmopsis primatum)		
Mycoplasma salivarium	Bacterium (synonym for Metamycoplasma salivarium)		
Mycoplasma spermatophilum	Bacterium		
Mycoplasmoides genitalium	Bacterium		
Mycoplasmoides pirum	Bacterium		
Mycoplasmoides pneumoniae	Bacterium		
Mycoplasmopsis fermentans	Bacterium		
Mycoplasmopsis lipophila	Bacterium		
Mycoplasmopsis primatum	Bacterium		
Mycotorula lipolytica	Fungus (synonym for Yarrowia lipolytica)		
Myroides	Bacterium		
Myroides odoratus	Bacterium		
Nakazawaea	Fungus (synonym for Pichia)		
Nannizzia	Fungus		
Necator	Fungus		
Neisseria	Bacterium		3GCR, Carbapenems
Neisseria animaloris	Bacterium		3GCR, Carbapenems
Neisseria bacilliformis	Bacterium		3GCR, Carbapenems
Neisseria canis	Bacterium		3GCR, Carbapenems
Neisseria cinerea	Bacterium		3GCR, Carbapenems
Neisseria cuniculi	Bacterium (synonym for Moraxella cuniculi)		
Neisseria dentiae	Bacterium		3GCR, Carbapenems
Neisseria dumasiana	Bacterium		3GCR, Carbapenems
Neisseria elongata	Bacterium		3GCR, Carbapenems
Neisseria elongata subsp. elongata	Bacterium		3GCR, Carbapenems
Neisseria elongata subsp. glycolytica	Bacterium		3GCR, Carbapenems
Neisseria elongata subsp. nitroreducens	Bacterium		3GCR, Carbapenems

Name	Type	Common Commensal	Recorded Resistances
Neisseria flava	Bacterium		3GCR, Carbapenems
Neisseria flavescens	Bacterium		3GCR, Carbapenems
Neisseria gonorrhoeae	Bacterium		3GCR, Carbapenems
Neisseria iguanae	Bacterium		3GCR, Carbapenems
Neisseria lactamica	Bacterium		3GCR, Carbapenems
Neisseria macacae	Bacterium		3GCR, Carbapenems
Neisseria meningitidis	Bacterium		3GCR, Carbapenems
Neisseria mucosa	Bacterium		3GCR, Carbapenems
Neisseria oralis	Bacterium		3GCR, Carbapenems
Neisseria ovis	Bacterium (synonym for Moraxella ovis)		
Neisseria perflava	Bacterium		3GCR, Carbapenems
Neisseria polysaccharea	Bacterium		3GCR, Carbapenems
Neisseria shayeganii	Bacterium		3GCR, Carbapenems
Neisseria sicca	Bacterium		3GCR, Carbapenems
Neisseria subflava	Bacterium		3GCR, Carbapenems
Neisseria wadsworthii	Bacterium		3GCR, Carbapenems
Neisseria weaveri	Bacterium		3GCR, Carbapenems
Neisseria zoodegmatis	Bacterium		3GCR, Carbapenems
Neobacillus bataviensis	Bacterium	Yes	
Neobacillus drentensis	Bacterium	Yes	
Neobacillus fumarioli	Bacterium	Yes	
Neobacillus niacini	Bacterium	Yes	
Neobacillus novalis	Bacterium	Yes	
Neobacillus pocheonensis	Bacterium	Yes	
Neobacillus soli	Bacterium	Yes	
Neobacillus vireti	Bacterium	Yes	
Neorickettsia	Bacterium		
Neorickettsia sennetsu	Bacterium (synonym for Ehrlichia sennetsu)		
Neosartorya	Fungus		
Neosartorya fischeri	Fungus (synonym for Aspergillus fischeri)		
Neoscytalidium dimidiatum	Fungus		
Nesterenkonia	Bacterium		
Nesterenkonia halobia	Bacterium		
Niallia circulans	Bacterium (synonym for Bacillus circulans)	Yes	
Niallia nealsonii	Bacterium (synonym for Bacillus nealsonii)	Yes	
Nigrospora	Fungus		
Nitratidesulfovibrio vulgaris	Bacterium		
Nocardia	Bacterium		
Nocardia abscessus	Bacterium		
Nocardia africana	Bacterium		
Nocardia anaemiae	Bacterium		
Nocardia aobensis	Bacterium		
Nocardia araoensis	Bacterium		
Nocardia arthritidis	Bacterium		
Nocardia asiatica	Bacterium		
Nocardia asteroides	Bacterium		
Nocardia asteroides complex	Bacterium		

Name	Type	Common Commensal	Recorded Resistances
Nocardia beijingensis	Bacterium		
Nocardia brasiliensis	Bacterium		
Nocardia brevicatena	Bacterium		
Nocardia carnea	Bacterium		
Nocardia cyriacigeorgica	Bacterium		
Nocardia elegans	Bacterium		
Nocardia farcinica	Bacterium		
Nocardia higoensis	Bacterium		
Nocardia inohanensis	Bacterium		
Nocardia kruczakiae	Bacterium		
Nocardia mexicana	Bacterium		
Nocardia niigatensis	Bacterium		
Nocardia nova	Bacterium		
Nocardia orientalis	Bacterium (synonym for Amycolatopsis orientalis)		
Nocardia otitidiscaviarum	Bacterium		
Nocardia paucivorans	Bacterium		
Nocardia pneumoniae	Bacterium		
Nocardia pseudobrasiliensis	Bacterium		
Nocardia sienata	Bacterium		
Nocardia testacea	Bacterium		
Nocardia transvalensis	Bacterium		
Nocardia veterana	Bacterium		
Nocardia yamanashiensis	Bacterium		
Nocardiopsis	Bacterium		
Nocardiopsis dassonvillei	Bacterium		
Nocardiopsis synnemataformans	Bacterium		
Norovirus	Virus		
Norovirus norwalkense	Virus		
North American eastern equine encephalitis virus	Virus (synonym for Alphavirus eastern)		
Norwalk virus	Virus (synonym for Norovirus norwalkense)		
Obesumbacterium	Bacterium		3GCR, Carbapenems, Colistin
Obesumbacterium proteus	Bacterium		3GCR, Carbapenems, Colistin
Ochrobactrum	Bacterium (synonym for Brucella)		
Ochrobactrum anthropi	Bacterium (synonym for Brucella anthropi)		
Ochrobactrum intermedium	Bacterium (synonym for Brucella intermedia)		
Ochroconis	Fungus (synonym for Scolecobasidium)		
Ochroconis constricta	Fungus (synonym for Scolecobasidium constrictum)		
Ochroconis gallopava	Fungus (synonym for Verruconis gallopava)		
Ochroconis humicola	Fungus		
Odoribacter	Bacterium		
Odoribacter splanchnicus	Bacterium		
Oerskovia	Bacterium	Yes	
Ogataea	Fungus		
Ogataea angusta	Fungus		
Ogataea polymorpha	Fungus		
Oidiodendron	Fungus		
Oligella	Bacterium		

Name	Type	Common Commensal	Recorded Resistances
Oligella ureolytica	Bacterium		
Oligella urethralis	Bacterium		
Orbivirus	Virus		
Orf virus	Virus (synonym for Parapoxvirus orf)		
Orientia	Bacterium		
Orientia tsutsugamushi	Bacterium		
Orthobunyavirus encephalitidis	Virus		
Orthoebolavirus	Virus		
Orthoflavivirus	Virus		
Orthoflavivirus denguei	Virus		
Orthoflavivirus encephalitidis	Virus		
Orthoflavivirus flavi	Virus		
Orthoflavivirus louisense	Virus		
Orthoflavivirus nilense	Virus		
Orthoflavivirus zikaense	Virus		
Orthohepadnavirus hominoidei	Virus		
Orthoherpesviridae	Virus		
Orthomarburgvirus	Virus		
Orthonairovirus haemorrhagiae	Virus		
Orthopneumovirus hominis	Virus		
Orthopoxvirus monkeypox	Virus		
Orthopoxvirus vaccinia	Virus		
Orthopoxvirus variola	Virus		
Orthoreovirus	Virus		
Orthorubulavirus hominis	Virus		
Orthorubulavirus laryngotracheitidis	Virus		
Orthorubulavirus parotitidis	Virus		
Paecilomyces	Fungus		
Paecilomyces javanicus	Fungus (synonym for Cordyceps javanica)		
Paecilomyces lilacinus	Fungus (synonym for Purpureocillium lilacinum)		
Paecilomyces marquandii	Fungus (synonym for Marquandomyces marquandii)		
Paecilomyces variotii	Fungus		
Paenarthrobacter aurescens	Bacterium	Yes	
Paenarthrobacter histidinolovorans	Bacterium	Yes	
Paenarthrobacter ilicis	Bacterium	Yes	
Paenarthrobacter nicotinovorans	Bacterium	Yes	
Paenarthrobacter nitroguajacolicus	Bacterium	Yes	
Paenarthrobacter ureafaciens	Bacterium	Yes	
Paenibacillus	Bacterium	Yes	
Paenibacillus agaridevorans	Bacterium	Yes	
Paenibacillus alvei	Bacterium	Yes	
Paenibacillus edaphicus	Bacterium	Yes	
Paenibacillus ehimensis	Bacterium	Yes	
Paenibacillus gordonae	Bacterium (synonym for Paenibacillus validus)	Yes	
Paenibacillus larvae	Bacterium	Yes	
Paenibacillus lentimorbus	Bacterium	Yes	
Paenibacillus macerans	Bacterium	Yes	

Name	Type	Common Commensal	Recorded Resistances
Paenibacillus mucilaginosus	Bacterium	Yes	
Paenibacillus pabuli	Bacterium	Yes	
Paenibacillus polymyxa	Bacterium	Yes	
Paenibacillus popilliae	Bacterium	Yes	
Paenibacillus provencensis	Bacterium	Yes	
Paenibacillus thiaminolyticus	Bacterium	Yes	
Paenibacillus urinalis	Bacterium	Yes	
Paenibacillus validus	Bacterium	Yes	
Paeniclostridium ghonii	Bacterium		
Paeniclostridium sordellii	Bacterium		
Paeniglutamicibacter gangotriensis	Bacterium	Yes	
Paeniglutamicibacter kerguelensis	Bacterium	Yes	
Paeniglutamicibacter psychrophenolicus	Bacterium	Yes	
Paeniglutamicibacter sulfureus	Bacterium	Yes	
Pandoraea	Bacterium		
Pandoraea apista	Bacterium		
Pandoraea norimbergensis	Bacterium		
Pandoraea pnomenusa	Bacterium		
Pandoraea pulmonicola	Bacterium		
Pandoraea sputorum	Bacterium		
Pantoea	Bacterium		3GCR, Carbapenems, Colistin
Pantoea agglomerans	Bacterium		3GCR, Carbapenems, Colistin
Pantoea ananas	Bacterium (synonym for Pantoea ananatis)		3GCR, Carbapenems, Colistin
Pantoea ananatis	Bacterium		3GCR, Carbapenems, Colistin
Pantoea brenneri	Bacterium		3GCR, Carbapenems, Colistin
Pantoea calida	Bacterium (synonym for Mixta calida)		3GCR, Carbapenems, Colistin
Pantoea conspicua	Bacterium		3GCR, Carbapenems, Colistin
Pantoea dispersa	Bacterium		3GCR, Carbapenems, Colistin
Pantoea eucrina	Bacterium		3GCR, Carbapenems, Colistin
Pantoea septica	Bacterium		3GCR, Carbapenems, Colistin
Parabacteroides	Bacterium		
Parabacteroides chongii	Bacterium		
Parabacteroides distasonis	Bacterium		
Parabacteroides goldsteinii	Bacterium		
Parabacteroides gordonii	Bacterium		
Parabacteroides merdae	Bacterium		
Paraburkholderia	Bacterium		
Paraburkholderia fungorum	Bacterium		
Paraclostridium bifermentans	Bacterium		
Paracoccus	Bacterium		
Paracoccus yeei	Bacterium		
Parageobacillus thermantarcticus	Bacterium	Yes	
Parageobacillus thermoglucosidasius	Bacterium		
Parapoxvirus orf	Virus		
Parascardovia	Bacterium		
Parascardovia denticolens	Bacterium		
Parvimonas	Bacterium		

Name	Type	Common Commensal	Recorded Resistances
Parvimonas micra	Bacterium		
Paslahepevirus balayani	Virus		
Pasteurella	Bacterium		Colistin
Pasteurella aerogenes	Bacterium		Colistin
Pasteurella bettyae	Bacterium		Colistin
Pasteurella caballi	Bacterium		Colistin
Pasteurella canis	Bacterium		Colistin
Pasteurella dagmatis	Bacterium		Colistin
Pasteurella haemolytica	Bacterium (synonym for Mannheimia haemolytica)		
Pasteurella multocida	Bacterium		Colistin
Pasteurella multocida subsp. gallicida	Bacterium		Colistin
Pasteurella multocida subsp. multocida	Bacterium		Colistin
Pasteurella multocida subsp. septica	Bacterium		Colistin
Pasteurella pneumotropica	Bacterium (synonym for Rodentibacter pneumotropicus)		Colistin
Pasteurella stomatis	Bacterium		Colistin
Pasteurella ureae	Bacterium (synonym for Actinobacillus ureae)		
Patulibacter	Bacterium		
Pauljensenia hongkongensis	Bacterium	Yes	
Pectobacterium	Bacterium		3GCR, Carbapenems, Colistin
Pediococcus	Bacterium		
Pediococcus acidilactici	Bacterium		
Pediococcus pentosaceus	Bacterium		
Pediococcus urinaeequi	Bacterium (synonym for Aerococcus urinaeequi)	Yes	
Pedobacter	Bacterium		
Pedobacter antarcticus	Bacterium		
Pedobacter heparinus	Bacterium		
Pedobacter piscium	Bacterium (synonym for Pedobacter antarcticus)		
Pegivirus C	Virus (synonym for Pegivirus hominis)		
Pegivirus hominis	Virus		
Penicillium	Fungus		
Penicillium aurantiogriseum var. aurantiogriseum	Fungus		
Penicillium commune	Fungus		
Penicillium decumbens	Fungus		
Penicillium expansum	Fungus		
Penicillium griseofulvum	Fungus		
Penicillium lilacinum	Fungus (synonym for Purpureocillium lilacinum)		
Penicillium marneffei	Fungus (synonym for Talaromyces marneffei)		
Penicillium patulum	Fungus (synonym for Penicillium griseofulvum)		
Penicillium puberulum	Fungus (synonym for Penicillium aurantiogriseum var. aurantiogriseum)		
Penicillium rubrum	Fungus (synonym for Talaromyces ruber)		
Penicillium spinulosum	Fungus		
Penicillium verruculosum	Fungus (synonym for Talaromyces verruculosus)		
Penicillium viridicatum	Fungus		
Peptococcus	Bacterium		
Peptococcus asaccharolyticus	Bacterium (synonym for Peptoniphilus asaccharolyticus)		

Name	Type	Common Commensal	Recorded Resistances
Peptococcus niger	Bacterium		
Peptococcus saccharolyticus	Bacterium (synonym for Staphylococcus saccharolyticus)	Yes	
Peptoniphilus	Bacterium		
Peptoniphilus asaccharolyticus	Bacterium		
Peptoniphilus harei	Bacterium		
Peptoniphilus ivorii	Bacterium		
Peptoniphilus lacrimalis	Bacterium		
Peptostreptococcus	Bacterium		
Peptostreptococcus anaerobius	Bacterium		
Peptostreptococcus asaccharolyticus	Bacterium (synonym for Peptoniphilus asaccharolyticus)		
Peptostreptococcus hydrogenalis	Bacterium (synonym for Anaerococcus hydrogenalis)		
Peptostreptococcus lactolyticus	Bacterium (synonym for Anaerococcus lactolyticus)		
Peptostreptococcus magnus	Bacterium (synonym for Finegoldia magna)		
Peptostreptococcus micros	Bacterium (synonym for Parvimonas micra)		
Peptostreptococcus octavius	Bacterium (synonym for Anaerococcus octavius)		
Peptostreptococcus parvulus	Bacterium (synonym for Lancefieldella parvula)		Carbapenems
Peptostreptococcus prevotii	Bacterium (synonym for Anaerococcus prevotii)		
Peptostreptococcus productus	Bacterium (synonym for Blautia producta)		
Peptostreptococcus tetradius	Bacterium (synonym for Anaerococcus tetradius)		
Peptostreptococcus vaginalis	Bacterium (synonym for Anaerococcus vaginalis)		
Peribacillus asahii	Bacterium	Yes	
Peribacillus butanolivorans	Bacterium	Yes	
Peribacillus muralis	Bacterium	Yes	
Peribacillus psychrosaccharolyticus	Bacterium	Yes	
Peribacillus simplex	Bacterium	Yes	
Petriellidium boydii	Fungus (synonym for Scedosporium boydii)		
Phaeoacremonium	Fungus		
Phaeoacremonium parasiticum	Fungus		
Phaeoannellomyces	Fungus		
Phaeoannellomyces werneckii	Fungus (synonym for Hortaea werneckii)		
Phaeococcomyces	Fungus		
Phaeococcomyces exophialae	Fungus (synonym for Exophiala exophialae)		
Phialemonium	Fungus		
Phialemonium curvatum	Fungus (synonym for Thyridium curvatum)		
Phialemonium dimorphosporum	Fungus		
Phialemonium obovatum	Fungus		
Phialophora	Fungus		
Phialophora americana	Fungus		
Phialophora compacta	Fungus (synonym for Fonsecaea pedrosoi)		
Phialophora dermatitidis	Fungus (synonym for Exophiala dermatitidis)		
Phialophora europaea	Fungus (synonym for Cyphellophora europaea)		
Phialophora gougerotii	Fungus (synonym for Sporotrichum gougerotii)		
Phialophora hoffmannii	Fungus (synonym for Coniochaeta hoffmannii)		
Phialophora jeanselmei	Fungus (synonym for Exophiala jeanselmei)		
Phialophora pedrosoi	Fungus (synonym for Fonsecaea pedrosoi)		

Name	Type	Common Commensal	Recorded Resistances
Phialophora richardsiae	Fungus (synonym for Pleurostoma richardsiae)		
Phialophora spinifera	Fungus (synonym for Exophiala spinifera)		
Phialophora verrucosa	Fungus		
Phlebovirus riftense	Virus		
Phocaeicola dorei	Bacterium		Carbapenems
Phocaeicola massiliensis	Bacterium		Carbapenems
Phocaeicola vulgatus	Bacterium		Carbapenems
Photobacterium	Bacterium		
Photobacterium damselae	Bacterium		
Photobacterium damselae subsp. damselae	Bacterium		
Photorhabdus	Bacterium		3GCR, Carbapenems, Colistin
Photorhabdus asymbiotica	Bacterium		3GCR, Carbapenems, Colistin
Photorhabdus luminescens	Bacterium		3GCR, Carbapenems, Colistin
Photorhabdus temperata	Bacterium		3GCR, Carbapenems, Colistin
Pichia	Fungus		
Pichia angusta	Fungus (synonym for Ogataea angusta)		
Pichia farinosa	Fungus (synonym for Millerozyma farinosa)		
Pichia fermentans	Fungus		
Pichia guilliermondii	Fungus (synonym for Meyerozyma guilliermondii)		
Pichia inconspicua	Fungus		
Pichia kudriavzevii	Fungus		
Pichia ohmeri	Fungus (synonym for Kodamaea ohmeri)		
Pichia polymorpha	Fungus (synonym for Schwanniomyces polymorphus)		
Pichiaceae	Fungus		
Pichiales	Fungus		
Pichiomycetes	Fungus		
Picornaviridae	Virus		
Piedraia	Fungus		
Piedraia hortae	Fungus		
Pithomyces	Fungus		
Pityrosporum	Fungus		
Pityrosporum canis	Fungus (synonym for Malassezia pachydermatis)		
Pityrosporum furfur	Fungus (synonym for Malassezia furfur)		
Pityrosporum orbiculare	Fungus		
Pityrosporum ovale	Fungus (synonym for Malassezia furfur)		
Pityrosporum pachydermatis	Fungus (synonym for Malassezia pachydermatis)		
Plasmodium	Fungus		
Plesiomonas	Bacterium		3GCR, Carbapenems, Colistin
Plesiomonas shigelloides	Bacterium		3GCR, Carbapenems, Colistin
Pleurostoma	Fungus		
Pleurostoma richardsiae	Fungus		
Pleurostomophora	Fungus (synonym for Pleurostoma)		
Pleurostomophora richardsiae	Fungus (synonym for Pleurostoma richardsiae)		
Pluralibacter	Bacterium		3GCR
Pluralibacter gergoviae	Bacterium		3GCR
Pluralibacter pyrinus	Bacterium		3GCR
Poliovirus	Virus		

Name	Type	Common Commensal	Recorded Resistances
Poliovirus 1	Virus		
Poliovirus 2	Virus		
Poliovirus 3	Virus		
Polyomaviridae	Virus		
Porphyromonas	Bacterium		
Porphyromonas asaccharolytica	Bacterium		
Porphyromonas cangingivalis	Bacterium		
Porphyromonas canoris	Bacterium		
Porphyromonas cansulci	Bacterium (synonym for Porphyromonas crevioricanis)		
Porphyromonas catoniae	Bacterium		
Porphyromonas circumdentaria	Bacterium		
Porphyromonas crevioricanis	Bacterium		
Porphyromonas endodontalis	Bacterium		
Porphyromonas gingivalis	Bacterium		
Porphyromonas gingivicanis	Bacterium		
Porphyromonas gulae	Bacterium		
Porphyromonas levii	Bacterium		
Porphyromonas macacae	Bacterium		
Porphyromonas salivosa	Bacterium (synonym for Porphyromonas macacae)		
Porphyromonas somerae	Bacterium		
Poxviridae	Virus		
Pragia	Bacterium		3GCR, Carbapenems, Colistin
Pragia fontium	Bacterium		3GCR, Carbapenems, Colistin
Prevotella	Bacterium		
Prevotella bivia	Bacterium		
Prevotella buccae	Bacterium		
Prevotella buccalis	Bacterium		
Prevotella corporis	Bacterium		
Prevotella dentalis	Bacterium		
Prevotella denticola	Bacterium		
Prevotella disiens	Bacterium		
Prevotella enoeca	Bacterium		
Prevotella heparinolytica	Bacterium		
Prevotella intermedia	Bacterium		
Prevotella loescheii	Bacterium		
Prevotella marshii	Bacterium		
Prevotella melaninogenica	Bacterium		
Prevotella multiformis	Bacterium		
Prevotella multisaccharivorax	Bacterium		
Prevotella nigrescens	Bacterium		
Prevotella oralis	Bacterium		
Prevotella oralis group	Bacterium		
Prevotella oris	Bacterium		
Prevotella oulora	Bacterium (synonym for Prevotella oulorum)		
Prevotella oulorum	Bacterium		
Prevotella pallens	Bacterium		
Prevotella ruminicola	Bacterium		

Name	Type	Common Commensal	Recorded Resistances
Prevotella salivae	Bacterium		
Prevotella shahii	Bacterium		
Prevotella tannerae	Bacterium (synonym for Alloprevotella tannerae)		Colistin
Prevotella veroralis	Bacterium		
Prevotella zoogleoformans	Bacterium (synonym for Bacteroides zoogleoformans)		
Priestia endophytica	Bacterium	Yes	
Priestia flexa	Bacterium	Yes	
Priestia megaterium	Bacterium	Yes	
Primate erythroparvovirus 1	Virus (synonym for Erythroparvovirus primate1)		
Primate T-lymphotropic virus 1	Virus (synonym for Deltaretrovirus priTlym1)		
Primate T-lymphotropic virus 2	Virus (synonym for Deltaretrovirus priTlym2)		
Primate T-lymphotropic virus 3	Virus (synonym for Deltaretrovirus priTlym3)		
Propionibacterium	Bacterium	Yes	
Propionibacterium acidipropionici	Bacterium (synonym for Acidipropionibacterium acidipropionici)	Yes	
Propionibacterium acnes	Bacterium (synonym for Cutibacterium acnes)	Yes	
Propionibacterium australiense	Bacterium	Yes	
Propionibacterium avidum	Bacterium (synonym for Cutibacterium avidum)	Yes	
Propionibacterium cyclohexanicum	Bacterium	Yes	
Propionibacterium freudenreichii	Bacterium	Yes	
Propionibacterium granulosum	Bacterium (synonym for Cutibacterium granulosum)	Yes	
Propionibacterium innocuum	Bacterium (synonym for Propioniferax innocua)	Yes	
Propionibacterium jensenii	Bacterium (synonym for Acidipropionibacterium jensenii)	Yes	
Propionibacterium lymphophilum	Bacterium (synonym for Propionimicrobium lymphophilum)		
Propionibacterium microaerophilum	Bacterium (synonym for Acidipropionibacterium microaerophilum)	Yes	
Propionibacterium propionicum	Bacterium (synonym for Arachnia propionica)	Yes	
Propionibacterium thoenii	Bacterium (synonym for Acidipropionibacterium thoenii)	Yes	
Propioniferax	Bacterium	Yes	
Propioniferax innocua	Bacterium	Yes	
Propionimicrobium	Bacterium		
Propionimicrobium lymphophilum	Bacterium		
Proteus	Bacterium		3GCR, Carbapenems
Proteus faecis	Bacterium		3GCR, Carbapenems
Proteus hauseri	Bacterium		3GCR, Carbapenems
Proteus mirabilis	Bacterium		3GCR, Carbapenems
Proteus myxofaciens	Bacterium		3GCR, Carbapenems
Proteus penneri	Bacterium		3GCR, Carbapenems
Proteus rettgeri	Bacterium (synonym for Providencia rettgeri)		3GCR, Carbapenems
Proteus vulgaris	Bacterium		3GCR, Carbapenems
Prototheca	Protozoon		
Prototheca filamenta	Fungus (synonym for Trichosporon asteroides)		
Prototheca wickerhamii	Protozoon		
Prototheca zopfii	Protozoon		
Providencia	Bacterium		3GCR, Carbapenems

Name	Type	Common Commensal	Recorded Resistances
Providencia alcalifaciens	Bacterium		3GCR, Carbapenems
Providencia friedericiana	Bacterium (synonym for Providencia rustigianii)		3GCR, Carbapenems
Providencia heimbachae	Bacterium		3GCR, Carbapenems
Providencia rettgeri	Bacterium		3GCR, Carbapenems
Providencia rustigianii	Bacterium		3GCR, Carbapenems
Providencia stuartii	Bacterium		3GCR, Carbapenems
Pseudalkalibacillus decolorationis	Bacterium	Yes	
Pseudalkalibacillus hwajinpoensis	Bacterium	Yes	
Pseudallescheria	Fungus (synonym for Scedosporium)		
Pseudallescheria boydii	Fungus (synonym for Scedosporium boydii)		
Pseudarthrobacter chlorophenolicus	Bacterium	Yes	
Pseudarthrobacter oxydans	Bacterium	Yes	
Pseudarthrobacter polychromogenes	Bacterium	Yes	
Pseudarthrobacter scleromae	Bacterium	Yes	
Pseudarthrobacter sulfonivorans	Bacterium	Yes	
Pseudescherichia vulneris	Bacterium		3GCR, Carbapenems, Colistin
Pseudobacillus badius	Bacterium		
Pseudoclavibacter	Bacterium		
Pseudoflavonifractor	Bacterium		
Pseudoflavonifractor capillosus	Bacterium		
Pseudoglutamicibacter albus	Bacterium	Yes	
Pseudoglutamicibacter cumminsii	Bacterium	Yes	
Pseudoleptotrichia goodfellowii	Bacterium		
Pseudomonas	Bacterium		3GCR, Carbapenems, Colistin
Pseudomonas acidovorans	Bacterium (synonym for Delftia acidovorans)		
Pseudomonas aeruginosa	Bacterium		3GCR, Carbapenems, Colistin
Pseudomonas alcaligenes	Bacterium		3GCR, Carbapenems, Colistin
Pseudomonas andersonii	Bacterium		3GCR, Carbapenems, Colistin
Pseudomonas antimicrobica	Bacterium (synonym for Burkholderia gladioli)		
Pseudomonas asiatica	Bacterium		3GCR, Carbapenems, Colistin
Pseudomonas beteli	Bacterium (synonym for Stenotrophomonas beteli)		
Pseudomonas betle	Bacterium (synonym for Stenotrophomonas beteli)		
Pseudomonas cepacia	Bacterium (synonym for Burkholderia cepacia)		
Pseudomonas chloritidismutans	Bacterium (synonym for Stutzerimonas stutzeri)		
Pseudomonas chlororaphis	Bacterium		3GCR, Carbapenems, Colistin
Pseudomonas cocovenenans	Bacterium (synonym for Burkholderia gladioli)		
Pseudomonas delafieldii	Bacterium (synonym for Acidovorax delafieldii)		
Pseudomonas diminuta	Bacterium (synonym for Brevundimonas diminuta)		
Pseudomonas facilis	Bacterium (synonym for Acidovorax facilis)		
Pseudomonas fluorescens	Bacterium		3GCR, Carbapenems, Colistin
Pseudomonas fulva	Bacterium		3GCR, Carbapenems, Colistin
Pseudomonas gladioli	Bacterium (synonym for Burkholderia gladioli)		
Pseudomonas japonica	Bacterium		3GCR, Carbapenems, Colistin
Pseudomonas juntendi	Bacterium		3GCR, Carbapenems, Colistin
Pseudomonas luteola	Bacterium		
Pseudomonas mallei	Bacterium (synonym for Burkholderia mallei)		

Name	Type	Common Commensal	Recorded Resistances
Pseudomonas maltophilia	Bacterium (synonym for Stenotrophomonas maltophilia)		
Pseudomonas marginalis	Bacterium		3GCR, Carbapenems, Colistin
Pseudomonas mendocina	Bacterium		3GCR, Carbapenems, Colistin
Pseudomonas monteilii	Bacterium		3GCR, Carbapenems, Colistin
Pseudomonas mosselii	Bacterium		3GCR, Carbapenems, Colistin
Pseudomonas nosocomialis	Bacterium (synonym for Stutzerimonas nosocomialis)		3GCR, Carbapenems, Colistin
Pseudomonas oleovorans	Bacterium		3GCR, Carbapenems, Colistin
Pseudomonas oryzihabitans	Bacterium		3GCR, Carbapenems, Colistin
Pseudomonas otitidis	Bacterium		3GCR, Carbapenems, Colistin
Pseudomonas paucimobilis	Bacterium (synonym for Sphingomonas paucimobilis)		
Pseudomonas perfectomarina	Bacterium (synonym for Stutzerimonas stutzeri)		
Pseudomonas pertucinogena	Bacterium (synonym for Halopseudomonas pertucinogena)		3GCR, Carbapenems, Colistin
Pseudomonas pickettii	Bacterium (synonym for Ralstonia pickettii)		
Pseudomonas poae	Bacterium		3GCR, Carbapenems, Colistin
Pseudomonas pseudoalcaligenes	Bacterium (synonym for Pseudomonas oleovorans)		3GCR, Carbapenems, Colistin
Pseudomonas pseudomallei	Bacterium (synonym for Burkholderia pseudomallei)		
Pseudomonas putida	Bacterium		3GCR, Carbapenems, Colistin
Pseudomonas pyrrocinia	Bacterium (synonym for Burkholderia pyrrocinia)		
Pseudomonas stutzeri	Bacterium (synonym for Stutzerimonas stutzeri)		
Pseudomonas testosteroni	Bacterium (synonym for Comamonas testosteroni)		
Pseudomonas veronii	Bacterium		3GCR, Carbapenems, Colistin
Pseudomonas vesicularis	Bacterium (synonym for Brevundimonas vesicularis)		
Pseudomonas yangonensis	Bacterium		3GCR, Carbapenems, Colistin
Pseudoramibacter	Bacterium		
Pseudoramibacter alactolyticus	Bacterium		
Pseudoxanthomonas	Bacterium		
Pseudoxanthomonas koreensis	Bacterium		
Pseudozyma	Fungus		
Pseudozyma aphidis	Fungus (synonym for Moesziomyces bullatus)		
Psychrobacillus	Bacterium		
Psychrobacter	Bacterium		
Psychrobacter immobilis	Bacterium		
Psychrobacter phenylpyruvicus	Bacterium		
Pullulanibacillus naganoensis	Bacterium	Yes	
Pullularia	Fungus (synonym for Aureobasidium)		
Pullularia pullulans	Fungus (synonym for Aureobasidium pullulans)		
Purpureocillium lilacinum	Fungus		
Pyrenochaeta	Fungus		
Pyrenochaeta romeroi	Fungus (synonym for Medicopsis romeroi)		
Pyrenophora	Fungus		
Quambalaria cyanescens	Fungus		
Rabies virus	Virus (synonym for Lyssavirus rabies)		
Rahnella	Bacterium		3GCR, Carbapenems, Colistin
Rahnella aquatilis	Bacterium		3GCR, Carbapenems, Colistin
Ralstonia	Bacterium		

Name	Type	Common Commensal	Recorded Resistances
Ralstonia gilardii	Bacterium (synonym for Cupriavidus gilardii)		
Ralstonia insidiosa	Bacterium		
Ralstonia mannitolilytica	Bacterium		
Ralstonia pickettii	Bacterium		
Ralstonia respiraculi	Bacterium (synonym for Cupriavidus respiraculi)		
Ramichloridium cerophilum	Fungus (synonym for Zasmidium cerophilum)		
Raoultella	Bacterium (synonym for Klebsiella)		3GCR, Carbapenems, Colistin
Raoultella ornithinolytica	Bacterium (synonym for Klebsiella ornithinolytica)		3GCR, Carbapenems
Raoultella planticola	Bacterium (synonym for Klebsiella planticola)		3GCR, Carbapenems
Raoultella terrigena	Bacterium (synonym for Klebsiella terrigena)		3GCR, Carbapenems
Respirovirus	Virus		
Respirovirus laryngotracheitidis	Virus		
Respirovirus pneumoniae	Virus		
Rhabdoviridae	Virus		
Rhadinovirus humangamma8	Virus		
Rhinocladiella aquaspersa	Fungus		
Rhinocladiella atrovirens	Fungus		
Rhinocladiella pedrosoi	Fungus (synonym for Fonsecaea pedrosoi)		
Rhinocladiella spinifera	Fungus (synonym for Exophiala spinifera)		
Rhinosporidium	Protozoon		
Rhizobium	Bacterium		
Rhizobium radiobacter	Bacterium (synonym for Agrobacterium radiobacter)		
Rhizobium rubi	Bacterium (synonym for Agrobacterium rubi)		
Rhizobium viscosum	Bacterium	Yes	
Rhizobium vitis	Bacterium (synonym for Allorhizobium vitis)		
Rhizomucor	Fungus		
Rhizomucor miehei	Fungus		
Rhizomucor pusillus	Fungus		
Rhodococcus	Bacterium	Yes	
Rhodococcus aurantiacus	Bacterium (synonym for Tsukamurella paurometabola)	Yes	
Rhodococcus bronchialis	Bacterium (synonym for Gordonia bronchialis)	Yes	
Rhodococcus chlorophenolicus	Bacterium (synonym for Mycobacterium chlorophenolicum)		
Rhodococcus chubuensis	Bacterium	Yes	
Rhodococcus equi	Bacterium	Yes	
Rhodococcus erythropolis	Bacterium	Yes	
Rhodococcus fascians	Bacterium	Yes	
Rhodococcus globerulus	Bacterium	Yes	
Rhodococcus gordoniae	Bacterium	Yes	
Rhodococcus hoagii	Bacterium	Yes	
Rhodococcus luteus	Bacterium (synonym for Rhodococcus fascians)	Yes	
Rhodococcus obuensis	Bacterium	Yes	
Rhodococcus rhodochrous	Bacterium	Yes	
Rhodococcus roseus	Bacterium (synonym for Rhodococcus rhodochrous)	Yes	
Rhodococcus rubropertinctus	Bacterium (synonym for Gordonia rubripertincta)	Yes	
Rhodococcus terrae	Bacterium (synonym for Gordonia terrae)	Yes	
Rhodotorula	Fungus		

Name	Type	Common Commensal	Recorded Resistances
Rhodotorula glutinis	Fungus		
Rhodotorula minuta	Fungus (synonym for Cystobasidium minutum)		
Rhodotorula mucilaginosa	Fungus		
Rhodotorula mucilaginosa var. mucilaginosa	Fungus (synonym for Rhodotorula mucilaginosa)		
Rhodotorula pilimanae	Fungus (synonym for Rhodotorula mucilaginosa)		
Rickettsia	Bacterium		
Rickettsia akari	Bacterium		
Rickettsia conorii	Bacterium		
Rickettsia parkeri	Bacterium		
Rickettsia prowazekii	Bacterium		
Rickettsia rickettsii	Bacterium		
Rickettsia sibirica	Bacterium		
Rickettsia tsutsugamushi	Bacterium (synonym for Orientia tsutsugamushi)		
Rickettsia typhi	Bacterium		
Riemerella	Bacterium		
Riemerella anatipestifer	Bacterium (synonym for Riemerella anatipestifera)		
Riemerella anatipestifera	Bacterium		
Rift Valley fever phlebovirus	Virus (synonym for Phlebovirus riftense)		
Rift Valley fever virus	Virus (synonym for Phlebovirus riftense)		
Robertmurraya korlensis	Bacterium	Yes	
Robertmurraya siralis	Bacterium	Yes	
Robinsoniella	Bacterium		
Robinsoniella peoriensis	Bacterium		
Rochalimaea elizabethae	Bacterium (synonym for Bartonella elizabethae)		
Rochalimaea henselae	Bacterium (synonym for Bartonella henselae)		
Rochalimaea quintana	Bacterium (synonym for Bartonella quintana)		
Rochalimaea vinsonii	Bacterium (synonym for Bartonella vinsonii)		
Rodentibacter pneumotropicus	Bacterium		Colistin
Roseolovirus humanbeta6a	Virus		
Roseolovirus humanbeta6b	Virus		
Roseomonas	Bacterium	Yes	
Roseomonas cervicalis	Bacterium	Yes	
Roseomonas gilardii	Bacterium	Yes	
Roseomonas mucosa	Bacterium	Yes	
Rossellomorea aquimaris	Bacterium	Yes	
Rossellomorea marisflavi	Bacterium	Yes	
Rossellomorea vietnamensis	Bacterium	Yes	
Rotavirus	Virus		
Rothia	Bacterium	Yes	
Rothia aeria	Bacterium	Yes	
Rothia dentocariosa	Bacterium	Yes	
Rothia kristinae	Bacterium	Yes	
Rothia mucilaginosa	Bacterium	Yes	
Rubella virus	Virus (synonym for Rubivirus rubellae)		
Rubivirus	Virus		
Rubivirus rubellae	Virus		
Ruminococcus	Bacterium		

Name	Type	Common Commensal	Recorded Resistances
Ruminococcus gnavus	Bacterium (synonym for Mediterraneibacter gnavus)		
Ruminococcus hansenii	Bacterium (synonym for Blautia hansenii)		
Ruminococcus productus	Bacterium (synonym for Blautia producta)		
Rummeliibacillus	Bacterium	Yes	
Rummeliibacillus pycnus	Bacterium	Yes	
Ruoffia tabacinasalis	Bacterium		
Saccharomonospora	Bacterium		
Saccharomonospora glauca	Bacterium		
Saccharomonospora viridis	Bacterium		
Saccharomycotina	Fungus		
Saccharopolyspora	Bacterium		
Saccharopolyspora rectivirgula	Bacterium		
Saint Louis encephalitis virus	Virus (synonym for Orthoflavivirus louisense)		
Saksenaea	Fungus		
Saksenaea vasiformis	Fungus		
Salipaludibacillus agaradhaerens	Bacterium	Yes	
Salisediminibacterium selenitireducens	Bacterium	Yes	
Salmonella	Bacterium		3GCR, Carbapenems, Colistin
Salmonella Bareilly	Bacterium (synonym for Salmonella enterica subsp. enterica Bareilly)		
Salmonella bongori	Bacterium		3GCR, Carbapenems, Colistin
Salmonella choleraesuis	Bacterium (synonym for Salmonella enterica subsp. enterica Choleraesuis)		
Salmonella enterica	Bacterium		3GCR, Carbapenems, Colistin
Salmonella enterica subsp. arizonae	Bacterium		3GCR, Carbapenems, Colistin
Salmonella enterica subsp. diarizonae	Bacterium		3GCR, Carbapenems, Colistin
Salmonella enterica subsp. enterica	Bacterium		3GCR, Carbapenems, Colistin
Salmonella enterica subsp. enterica Bareilly	Bacterium		
Salmonella enterica subsp. enterica Choleraesuis	Bacterium		
Salmonella enterica subsp. enterica Enteritidis	Bacterium		
Salmonella enterica subsp. enterica Infantis	Bacterium		
Salmonella enterica subsp. enterica Isangi	Bacterium		
Salmonella enterica subsp. enterica Kottbus	Bacterium		
Salmonella enterica subsp. enterica Livingstone	Bacterium		
Salmonella enterica subsp. enterica Montevideo	Bacterium		
Salmonella enterica subsp. enterica Newport	Bacterium		
Salmonella enterica subsp. enterica Ohio	Bacterium		
Salmonella enterica subsp. enterica Senftenberg	Bacterium		
Salmonella enterica subsp. enterica Tennessee	Bacterium		
Salmonella enterica subsp. enterica Typhi	Bacterium		
Salmonella enterica subsp. enterica Typhimurium	Bacterium		
Salmonella enterica subsp. enterica Urbana	Bacterium		
Salmonella enterica subsp. enterica Virchow	Bacterium		
Salmonella enterica subsp. enterica Worthington	Bacterium		
Salmonella enterica subsp. houtenae	Bacterium		3GCR, Carbapenems, Colistin
Salmonella enterica subsp. indica	Bacterium		3GCR, Carbapenems, Colistin
Salmonella enterica subsp. salamae	Bacterium		3GCR, Carbapenems, Colistin

Name	Type	Common Commensal	Recorded Resistances
Salmonella enteritidis	Bacterium (synonym for Salmonella enterica subsp. enterica Enteritidis)		
Salmonella Infantis	Bacterium (synonym for Salmonella enterica subsp. enterica Infantis)		
Salmonella Isangi	Bacterium (synonym for Salmonella enterica subsp. enterica Isangi)		
Salmonella Kottbus	Bacterium (synonym for Salmonella enterica subsp. enterica Kottbus)		
Salmonella Livingstone	Bacterium (synonym for Salmonella enterica subsp. enterica Livingstone)		
Salmonella Montevideo	Bacterium (synonym for Salmonella enterica subsp. enterica Montevideo)		
Salmonella Newport	Bacterium (synonym for Salmonella enterica subsp. enterica Newport)		
Salmonella Ohio	Bacterium (synonym for Salmonella enterica subsp. enterica Ohio)		
Salmonella Senftenberg	Bacterium (synonym for Salmonella enterica subsp. enterica Senftenberg)		
Salmonella Tennessee	Bacterium (synonym for Salmonella enterica subsp. enterica Tennessee)		
Salmonella typhi	Bacterium (synonym for Salmonella enterica subsp. enterica Typhi)		
Salmonella typhimurium	Bacterium (synonym for Salmonella enterica subsp. enterica Typhimurium)		
Salmonella Urbana	Bacterium (synonym for Salmonella enterica subsp. enterica Urbana)		
Salmonella Virchow	Bacterium (synonym for Salmonella enterica subsp. enterica Virchow)		
Salmonella Worthington	Bacterium (synonym for Salmonella enterica subsp. enterica Worthington)		
Sarcina	Bacterium		
Sarcina ventriculi	Bacterium		
Sarcinosporon	Fungus (synonym for Trichosporon)		
Sarcinosporon inkin	Fungus (synonym for Trichosporon inkin)		
Sarcocystis	Fungus		
Sarocladium kiliense	Fungus		
Sarocladium strictum	Fungus		
SARS-CoV-2	Virus (synonym for Severe acute respiratory syndrome coronavirus 2)		
Scardovia	Bacterium		
Scardovia inopinata	Bacterium		
Scedosporium	Fungus		
Scedosporium apiospermum	Fungus		
Scedosporium boydii	Fungus		
Scedosporium inflatum	Fungus (synonym for Lomentospora prolificans)		
Scedosporium prolificans	Fungus (synonym for Lomentospora prolificans)		
Schaalia cardiffensis	Bacterium	Yes	
Schaalia funkei	Bacterium	Yes	
Schaalia georgiae	Bacterium	Yes	
Schaalia meyeri	Bacterium	Yes	
Schaalia odontolytica	Bacterium	Yes	

Name	Type	Common Commensal	Recorded Resistances
Schaalia radingae	Bacterium	Yes	
Schaalia turicensis	Bacterium	Yes	
Schinkia azotoformans	Bacterium	Yes	
Schizophyllum	Fungus		
Schizophyllum commune	Fungus		
Schwanniomyces	Fungus (synonym for Debaryomyces)		
Schwanniomyces polymorphus	Fungus		
Scolecobasidium	Fungus		
Scolecobasidium constrictum	Fungus		
Scolecobasidium humicola	Fungus (synonym for Ochroconis humicola)		
Scopulariopsis	Fungus		
Scopulariopsis acremonium	Fungus (synonym for Acaulium acremonium)		
Scopulariopsis brevicaulis	Fungus		
Scopulariopsis brumptii	Fungus (synonym for Microascus paisii)		
Scopulariopsis candida	Fungus		
Scytalidium	Fungus		
Scytalidium dimidiatum	Fungus (synonym for Neoscytalidium dimidiatum)		
Scytalidium hyalinum	Fungus (synonym for Neoscytalidium dimidiatum)		
Selenomonas	Bacterium		
Selenomonas artemidis	Bacterium		
Selenomonas dianae	Bacterium		
Selenomonas flueggei	Bacterium		
Selenomonas infelix	Bacterium		
Selenomonas noxia	Bacterium		
Selenomonas sputigena	Bacterium		
Sepedonium	Fungus (synonym for Hypomyces)		
Serinales	Fungus		
Serpulina	Bacterium (synonym for Brachyspira)		
Serpulina pilosicoli	Bacterium (synonym for Brachyspira pilosicoli)		
Serratia	Bacterium		3GCR, Carbapenems
Serratia entomophila	Bacterium		3GCR, Carbapenems
Serratia ficaria	Bacterium		3GCR, Carbapenems
Serratia fonticola	Bacterium		3GCR, Carbapenems
Serratia grimesii	Bacterium		3GCR, Carbapenems
Serratia liquefaciens	Bacterium		3GCR, Carbapenems
Serratia marcescens	Bacterium		3GCR, Carbapenems
Serratia marinorubra	Bacterium (synonym for Serratia rubidaea)		3GCR, Carbapenems
Serratia odorifera	Bacterium		3GCR, Carbapenems
Serratia plymuthica	Bacterium		3GCR, Carbapenems
Serratia proteamaculans	Bacterium		3GCR, Carbapenems
Serratia quinivorans	Bacterium		3GCR, Carbapenems
Serratia rubidaea	Bacterium		3GCR, Carbapenems
Serratia ureilytica	Bacterium		3GCR, Carbapenems
Severe acute respiratory syndrome coronavirus 2	Virus		
Severe acute respiratory syndrome-related coronavirus	Virus (synonym for Betacoronavirus pandemicum)		
Shewanella	Bacterium		
Shewanella algae	Bacterium		

Name	Type	Common Commensal	Recorded Resistances
Shewanella putrefaciens	Bacterium		
Shigella	Bacterium		3GCR, Carbapenems, Colistin
Shigella boydii	Bacterium		3GCR, Carbapenems, Colistin
Shigella dysenteriae	Bacterium		3GCR, Carbapenems, Colistin
Shigella flexneri	Bacterium		3GCR, Carbapenems, Colistin
Shigella sonnei	Bacterium		3GCR, Carbapenems, Colistin
Shouchella clausii	Bacterium	Yes	
Shouchella gibsonii	Bacterium (synonym for Alkalicoccobacillus gibsonii)		
Shouchella patagoniensis	Bacterium	Yes	
Simian virus 40	Virus (synonym for Betapolyomavirus macacae)		
Siminovitchia farraginis	Bacterium	Yes	
Siminovitchia fordii	Bacterium	Yes	
Siminovitchia fortis	Bacterium	Yes	
Simonsiella	Bacterium		
Simonsiella crassa	Bacterium (synonym for Alysiella crassa)		
Simonsiella muelleri	Bacterium		
Simplexvirus humanalpha1	Virus		
Simplexvirus humanalpha2	Virus		
Slackia	Bacterium		
Slackia exigua	Bacterium		
Slackia heliotrinireducens	Bacterium		
Sneathia	Bacterium		
Sneathia sanguinegens	Bacterium		
Sodalis praecaptivus	Bacterium		3GCR, Carbapenems, Colistin
Solibacillus	Bacterium	Yes	
Solibacillus silvestris	Bacterium	Yes	
Solobacterium	Bacterium		
Solobacterium moorei	Bacterium		
South American eastern equine encephalitis virus	Virus (synonym for Alphavirus madariaga)		
Sphaerostilbella	Fungus		
Sphingobacterium	Bacterium		
Sphingobacterium faecium	Bacterium		
Sphingobacterium heparinum	Bacterium (synonym for Pedobacter heparinus)		
Sphingobacterium mizutae	Bacterium (synonym for Sphingobacterium mizutaii)		
Sphingobacterium mizutaii	Bacterium		
Sphingobacterium multivorum	Bacterium		
Sphingobacterium piscium	Bacterium (synonym for Pedobacter antarcticus)		
Sphingobacterium spiritivorum	Bacterium		
Sphingobacterium thalpophilum	Bacterium		
Sphingomonas	Bacterium		
Sphingomonas parapaucimobilis	Bacterium		
Sphingomonas paucimobilis	Bacterium		
Spirillum	Bacterium		
Sporobolomyces	Fungus		
Sporobolomyces roseus	Fungus		
Sporobolomyces salmonicolor	Fungus		
Sporolactobacillus	Bacterium		

Name	Type	Common Commensal	Recorded Resistances
Sporolactobacillus laevolacticus	Bacterium		
Sporopachydermia	Fungus		
Sporosarcina	Bacterium		
Sporosarcina pasteurii	Bacterium	Yes	
Sporosarcina psychrophila	Bacterium	Yes	
Sporothrix	Fungus		
Sporothrix cyanescens	Fungus (synonym for Quambalaria cyanescens)		
Sporothrix schenckii	Fungus		
Sporotrichum beurmannii	Fungus (synonym for Sporothrix schenckii)		
Sporotrichum gougerotii	Fungus		
Sporotrichum pruinosum	Fungus		
Sporotrichum schenckii	Fungus (synonym for Sporothrix schenckii)		
St. Louis encephalitis virus	Virus (synonym for Orthoflavivirus louisense)		
Stachybotrys	Fungus		
Stachybotrys chartarum	Fungus		
Staphylococcus	Bacterium		
Staphylococcus argenteus	Bacterium		
Staphylococcus arlettae	Bacterium	Yes	
Staphylococcus aureus	Bacterium		MRSA
Staphylococcus auricularis	Bacterium	Yes	
Staphylococcus borealis	Bacterium	Yes	
Staphylococcus capitis	Bacterium	Yes	
Staphylococcus capitis subsp. capitis	Bacterium	Yes	
Staphylococcus capitis subsp. urealyticus	Bacterium	Yes	
Staphylococcus caprae	Bacterium	Yes	
Staphylococcus carnosus	Bacterium	Yes	
Staphylococcus carnosus subsp. carnosus	Bacterium	Yes	
Staphylococcus carnosus subsp. utilis	Bacterium (synonym for Staphylococcus carnosus)	Yes	
Staphylococcus casei	Bacterium	Yes	
Staphylococcus caseolyticus	Bacterium (synonym for Macrococcus caseolyticus)		
Staphylococcus chromogenes	Bacterium	Yes	
Staphylococcus coagulans	Bacterium		
Staphylococcus cohnii	Bacterium	Yes	
Staphylococcus cohnii subsp. cohnii	Bacterium (synonym for Staphylococcus cohnii)	Yes	
Staphylococcus cohnii subsp. urealyticus	Bacterium (synonym for Staphylococcus ureilyticus)	Yes	
Staphylococcus condimenti	Bacterium	Yes	
Staphylococcus cornubiensis	Bacterium		
Staphylococcus delphini	Bacterium		
Staphylococcus epidermidis	Bacterium	Yes	
Staphylococcus equorum	Bacterium	Yes	
Staphylococcus equorum subsp. equorum	Bacterium	Yes	
Staphylococcus equorum subsp. linens	Bacterium	Yes	
Staphylococcus felis	Bacterium	Yes	
Staphylococcus fleurettii	Bacterium	Yes	
Staphylococcus gallinarum	Bacterium	Yes	
Staphylococcus haemolyticus	Bacterium	Yes	
Staphylococcus hominis	Bacterium	Yes	

Name	Type	Common Commensal	Recorded Resistances
Staphylococcus hominis subsp. hominis	Bacterium	Yes	
Staphylococcus hominis subsp. novobiosepticus	Bacterium	Yes	
Staphylococcus hyicus	Bacterium		
Staphylococcus hyicus subsp. chromogenes	Bacterium (synonym for Staphylococcus chromogenes)	Yes	
Staphylococcus hyicus subsp. hyicus	Bacterium (synonym for Staphylococcus hyicus)		
Staphylococcus intermedius	Bacterium		
Staphylococcus kloosii	Bacterium	Yes	
Staphylococcus lentus	Bacterium	Yes	
Staphylococcus lugdunensis	Bacterium	Yes	
Staphylococcus lutrae	Bacterium		
Staphylococcus massiliensis	Bacterium	Yes	
Staphylococcus muscae	Bacterium	Yes	
Staphylococcus nepalensis	Bacterium	Yes	
Staphylococcus pasteuri	Bacterium	Yes	
Staphylococcus pettenkoferi	Bacterium	Yes	
Staphylococcus piscifermentans	Bacterium	Yes	
Staphylococcus pseudintermedius	Bacterium		
Staphylococcus pseudolugdunensis	Bacterium	Yes	
Staphylococcus pulvereri	Bacterium (synonym for Staphylococcus vitulinus)	Yes	
Staphylococcus saccharolyticus	Bacterium	Yes	
Staphylococcus saprophyticus	Bacterium	Yes	
Staphylococcus saprophyticus subsp. bovis	Bacterium (synonym for Staphylococcus saprophyticus)	Yes	
Staphylococcus saprophyticus subsp. saprophyticus	Bacterium	Yes	
Staphylococcus schleiferi	Bacterium		
Staphylococcus schleiferi subsp. coagulans	Bacterium (synonym for Staphylococcus coagulans)		
Staphylococcus schleiferi subsp. schleiferi	Bacterium (synonym for Staphylococcus schleiferi)		
Staphylococcus sciuri	Bacterium	Yes	
Staphylococcus sciuri subsp. carnaticus	Bacterium (synonym for Staphylococcus sciuri)	Yes	
Staphylococcus sciuri subsp. lentus	Bacterium (synonym for Staphylococcus lentus)	Yes	
Staphylococcus sciuri subsp. rodentium	Bacterium (synonym for Staphylococcus sciuri)	Yes	
Staphylococcus sciuri subsp. sciuri	Bacterium	Yes	
Staphylococcus simulans	Bacterium	Yes	
Staphylococcus succinus	Bacterium	Yes	
Staphylococcus succinus subsp. casei	Bacterium (synonym for Staphylococcus casei)	Yes	
Staphylococcus succinus subsp. succinus	Bacterium (synonym for Staphylococcus succinus)	Yes	
Staphylococcus ureilyticus	Bacterium	Yes	
Staphylococcus vitulinus	Bacterium	Yes	
Staphylococcus warneri	Bacterium	Yes	
Staphylococcus xylosus	Bacterium	Yes	
Stemphylium	Fungus		
Stenotrophomonas	Bacterium		
Stenotrophomonas beteli	Bacterium		
Stenotrophomonas maltophilia	Bacterium		
Stephanoascus	Fungus (synonym for Blastobotrys)		
Stephanoascus ciferrii	Fungus (synonym for Trichomonascus ciferrii)		
Stomatococcus mucilaginosus	Bacterium (synonym for Rothia mucilaginosa)	Yes	
Streptobacillus	Bacterium		

Name	Type	Common Commensal	Recorded Resistances
Streptobacillus moniliformis	Bacterium		
Streptococcus	Bacterium		
Streptococcus acidominimus	Bacterium		
Streptococcus adjacens	Bacterium (synonym for Granulicatella adiacens)		
Streptococcus agalactiae	Bacterium		
Streptococcus alactolyticus	Bacterium	Yes	
Streptococcus anginosus	Bacterium	Yes	
Streptococcus anginosus group	Bacterium		
Streptococcus australis	Bacterium	Yes	
Streptococcus bovis	Bacterium (synonym for Streptococcus equinus)	Yes	
Streptococcus bovis group	Bacterium		
Streptococcus canis	Bacterium		
Streptococcus caprinus	Bacterium (synonym for Streptococcus gallolyticus)	Yes	
Streptococcus cecorum	Bacterium (synonym for Enterococcus cecorum)		VRE
Streptococcus constellatus	Bacterium	Yes	
Streptococcus constellatus subsp. constellatus	Bacterium	Yes	
Streptococcus constellatus subsp. pharyngis	Bacterium	Yes	
Streptococcus criceti	Bacterium	Yes	
Streptococcus cristatus	Bacterium	Yes	
Streptococcus defectivus	Bacterium (synonym for Abiotrophia defectiva)		
Streptococcus dentisani	Bacterium (synonym for Streptococcus oralis subsp. dentisani)	Yes	
Streptococcus devriesei	Bacterium	Yes	
Streptococcus downei	Bacterium	Yes	
Streptococcus durans	Bacterium (synonym for Enterococcus durans)		VRE
Streptococcus dysgalactiae	Bacterium		
Streptococcus dysgalactiae subsp. dysgalactiae	Bacterium		
Streptococcus dysgalactiae subsp. equisimilis	Bacterium		
Streptococcus entericus	Bacterium		
Streptococcus equi	Bacterium		
Streptococcus equi subsp. equi	Bacterium		
Streptococcus equi subsp. zooepidemicus	Bacterium		
Streptococcus equinus	Bacterium	Yes	
Streptococcus faecalis	Bacterium (synonym for Enterococcus faecalis)		VRE
Streptococcus faecium	Bacterium (synonym for Enterococcus faecium)		VRE
Streptococcus ferus	Bacterium	Yes	
Streptococcus gallinaceus	Bacterium	Yes	
Streptococcus gallinarum	Bacterium (synonym for Enterococcus gallinarum)		
Streptococcus gallolyticus	Bacterium	Yes	
Streptococcus gallolyticus subsp. gallolyticus	Bacterium	Yes	
Streptococcus gallolyticus subsp. macedonicus	Bacterium	Yes	
Streptococcus gallolyticus subsp. pasteurianus	Bacterium	Yes	
Streptococcus gordonii	Bacterium	Yes	
Streptococcus halichoeri	Bacterium		
Streptococcus hansenii	Bacterium (synonym for Blautia hansenii)		
Streptococcus hongkongensis	Bacterium		
Streptococcus hyovaginalis	Bacterium	Yes	

Name	Type	Common Commensal	Recorded Resistances
Streptococcus infantarius	Bacterium	Yes	
Streptococcus infantarius subsp. coli	Bacterium	Yes	
Streptococcus infantarius subsp. infantarius	Bacterium	Yes	
Streptococcus infantis	Bacterium	Yes	
Streptococcus iniae	Bacterium		
Streptococcus intermedius	Bacterium	Yes	
Streptococcus intestinalis	Bacterium (synonym for Streptococcus alactolyticus)	Yes	
Streptococcus lactarius	Bacterium	Yes	
Streptococcus lactis	Bacterium (synonym for Lactococcus lactis)		
Streptococcus lutetiensis	Bacterium	Yes	
Streptococcus macacae	Bacterium	Yes	
Streptococcus massiliensis	Bacterium		
Streptococcus merionis	Bacterium		
Streptococcus milleri group	Bacterium (synonym for Streptococcus anginosus group)		
Streptococcus minor	Bacterium	Yes	
Streptococcus mitis	Bacterium	Yes	
Streptococcus mitis group	Bacterium		
Streptococcus morbillorum	Bacterium (synonym for Gemella morbillorum)		
Streptococcus mutans	Bacterium	Yes	
Streptococcus mutans group	Bacterium		
Streptococcus oligofermentans	Bacterium (synonym for Streptococcus cristatus)	Yes	
Streptococcus oralis	Bacterium	Yes	
Streptococcus oralis subsp. dentisani	Bacterium	Yes	
Streptococcus oralis subsp. tigurinus	Bacterium	Yes	
Streptococcus ovis	Bacterium		
Streptococcus parasanguinis	Bacterium	Yes	
Streptococcus parauberis	Bacterium		
Streptococcus parvulus	Bacterium (synonym for Lancefieldella parvula)		Carbapenems
Streptococcus pasteurianus	Bacterium (synonym for Streptococcus gallolyticus subsp. pasteurianus)	Yes	
Streptococcus peroris	Bacterium	Yes	
Streptococcus pluranimalium	Bacterium		
Streptococcus pneumoniae	Bacterium		
Streptococcus porcinus	Bacterium		
Streptococcus pseudopneumoniae	Bacterium	Yes	
Streptococcus pseudoporcinus	Bacterium		
Streptococcus pyogenes	Bacterium		
Streptococcus ratti	Bacterium	Yes	
Streptococcus saccharolyticus	Bacterium (synonym for Enterococcus saccharolyticus)		VRE
Streptococcus salivarius	Bacterium	Yes	
Streptococcus salivarius group	Bacterium		
Streptococcus salivarius subsp. salivarius	Bacterium	Yes	
Streptococcus salivarius subsp. thermophilus	Bacterium	Yes	
Streptococcus sanguinis	Bacterium	Yes	
Streptococcus sanguinis group	Bacterium		
Streptococcus sanguis	Bacterium (synonym for Streptococcus sanguinis)	Yes	

Name	Type	Common Commensal	Recorded Resistances
Streptococcus shiloi	Bacterium (synonym for Streptococcus iniae)		
Streptococcus sinensis	Bacterium	Yes	
Streptococcus sobrinus	Bacterium	Yes	
Streptococcus suis	Bacterium		
Streptococcus thermophilus	Bacterium (synonym for Streptococcus salivarius subsp. thermophilus)	Yes	
Streptococcus thoraltensis	Bacterium	Yes	
Streptococcus tigurinus	Bacterium (synonym for Streptococcus oralis subsp. tigurinus)	Yes	
Streptococcus uberis	Bacterium		
Streptococcus urinalis	Bacterium		
Streptococcus vestibularis	Bacterium	Yes	
Streptomyces	Bacterium		
Streptomyces albus	Bacterium		
Streptomyces bikiniensis	Bacterium		
Streptomyces candidus	Bacterium		
Streptomyces caviscabies	Bacterium (synonym for Streptomyces griseus)		
Streptomyces clavuligerus	Bacterium		
Streptomyces erumpens	Bacterium (synonym for Streptomyces griseus)		
Streptomyces filamentosus	Bacterium		
Streptomyces griseus	Bacterium		
Streptomyces lincolnensis	Bacterium		
Streptomyces roseosporus	Bacterium (synonym for Streptomyces filamentosus)		
Streptomyces setonii	Bacterium (synonym for Streptomyces griseus)		
Streptomyces somaliensis	Bacterium		
Streptomyces spectabilis	Bacterium		
Streptomyces thermovulgaris	Bacterium		
Streptomyces venezuelae	Bacterium		
Stutzerimonas nosocomialis	Bacterium		3GCR, Carbapenems, Colistin
Stutzerimonas stutzeri	Bacterium		
Succinivibrio	Bacterium		
Succinivibrio dextrinosolvens	Bacterium		
Sungouiella	Fungus		
Sungouiella intermedia	Fungus		
Sutcliffiella cohnii	Bacterium	Yes	
Sutcliffiella halmapala	Bacterium	Yes	
Sutcliffiella horikoshii	Bacterium	Yes	
Sutterella	Bacterium		
Sutterella wadsworthensis	Bacterium		
Suttonella	Bacterium		
Suttonella indologenes	Bacterium		
Sydowia	Fungus		
Syncephalastrum	Fungus		
Syncephalastrum racemosum	Fungus		
Talaromyces	Fungus		
Talaromyces marneffei	Fungus		
Talaromyces ruber	Fungus		

Name	Type	Common Commensal	Recorded Resistances
Talaromyces verruculosus	Fungus		
Tanapox virus	Virus (synonym for Yatapoxvirus tanapox)		
Tannerella	Bacterium		
Tannerella forsythia	Bacterium		
Tatlockia micdadei	Bacterium (synonym for Legionella micdadei)		
Tatumella	Bacterium		3GCR, Carbapenems, Colistin
Tatumella ptyseos	Bacterium		3GCR, Carbapenems, Colistin
Tatumella saanichensis	Bacterium		3GCR, Carbapenems, Colistin
Tausonia pullulans	Fungus		
Terrisporobacter glycolicus	Bacterium		
Tetragenococcus	Bacterium		
Tetragenococcus halophilus	Bacterium		
Tetragenococcus solitarius	Bacterium		
Thermoactinomyces	Bacterium		
Thyridium curvatum	Fungus		
Tick-borne encephalitis virus	Virus (synonym for Orthoflavivirus encephalitidis)		
Tissierella	Bacterium		
Tissierella praeacuta	Bacterium		
Togaviridae	Virus		
Torula dimidiata	Fungus (synonym for Neoscytalidium dimidiatum)		
Torula lipolytica	Fungus (synonym for Yarrowia lipolytica)		
Torulopsis aggregata	Fungus (synonym for Wickerhamiella pararugosa)		
Torulopsis haemulonii	Fungus (synonym for Candidozyma haemuli)		
Torulopsis inconspicua	Fungus (synonym for Pichia inconspicua)		
Torulopsis pintolopesii	Fungus (synonym for Candida pintolopesii)		
Trabulsiella	Bacterium		3GCR, Carbapenems, Colistin
Trabulsiella guamensis	Bacterium		3GCR, Carbapenems, Colistin
Trematosphaeria grisea	Fungus		
Treponema	Bacterium		
Treponema carateum	Bacterium		
Treponema pallidum	Bacterium		
Trichomonascaceae	Fungus		
Trichomonascus ciferrii	Fungus		
Trichophyton	Fungus		
Trichosporon	Fungus		
Trichosporon asteroides	Fungus		
Trichosporon beigelii	Fungus		
Trichosporon cutaneum	Fungus (synonym for Cutaneotrichosporon cutaneum)		
Trichosporon inkin	Fungus		
Trichosporon loubieri	Fungus (synonym for Apiotrichum loubieri)		
Trichosporon mucoides	Fungus (synonym for Cutaneotrichosporon mucoides)		
Trichosporon mycotoxinivorans	Fungus (synonym for Apiotrichum mycotoxinovorans)		
Trichosporon ovoides	Fungus		
Trichosporon penicillatum	Fungus (synonym for Geotrichum klebahnii)		
Trichosporon pullulans	Fungus (synonym for Tausonia pullulans)		
Trichothecium	Fungus		
Tropheryma	Bacterium		

Name	Type	Common Commensal	Recorded Resistances
Tropheryma whipplei	Bacterium		
Trueperella	Bacterium	Yes	
Trueperella bernardiae	Bacterium	Yes	
Trueperella pyogenes	Bacterium	Yes	
Tsukamurella	Bacterium	Yes	
Tsukamurella inchonensis	Bacterium	Yes	
Tsukamurella paurometabola	Bacterium	Yes	
Tsukamurella pulmonis	Bacterium	Yes	
Tsukamurella strandjordii	Bacterium	Yes	
Tsukamurella tyrosinosolvens	Bacterium	Yes	
Turicella	Bacterium (synonym for Corynebacterium)	Yes	
Turicella otitidis	Bacterium (synonym for Corynebacterium otitidis)	Yes	
Turicibacter	Bacterium		
Turicibacter sanguinis	Bacterium		
Ulocladium	Fungus (synonym for Alternaria)		
Ureaplasma	Bacterium		
Ureaplasma parvum	Bacterium		
Ureaplasma urealyticum	Bacterium		
Ureibacillus massiliensis	Bacterium	Yes	
Ustilago	Fungus		
Vaccinia virus	Virus (synonym for Orthopoxvirus vaccinia)		
Vagococcus	Bacterium		
Vagococcus fluvialis	Bacterium		
Varicella zoster virus	Virus (synonym for Varicellovirus humanalpha3)		
Varicellovirus	Virus		
Varicellovirus humanalpha3	Virus		
Variola virus	Virus (synonym for Orthopoxvirus variola)		
Veillonella	Bacterium		
Veillonella alcalescens	Bacterium (synonym for Veillonella parvula)		
Veillonella atypica	Bacterium		
Veillonella caviae	Bacterium		
Veillonella criceti	Bacterium		
Veillonella dispar	Bacterium		
Veillonella montpellierensis	Bacterium		
Veillonella parvula	Bacterium		
Veillonella ratti	Bacterium		
Veillonella rodentium	Bacterium		
Venezuelan equine encephalitis virus	Virus (synonym for Alphavirus venezuelan)		
Verruconis gallopava	Fungus		
Verticillium	Fungus		
Vibrio	Bacterium		Colistin
Vibrio alginolyticus	Bacterium		Colistin
Vibrio carchariae	Bacterium (synonym for Vibrio harveyi)		Colistin
Vibrio cholerae	Bacterium		Colistin
Vibrio cincinnatiensis	Bacterium		Colistin
Vibrio damsela	Bacterium (synonym for Photobacterium damselae)		
Vibrio damselae	Bacterium (synonym for Photobacterium damselae)		

Name	Type	Common Commensal	Recorded Resistances
Vibrio fluvialis	Bacterium		Colistin
Vibrio furnissii	Bacterium		Colistin
Vibrio harveyi	Bacterium		Colistin
Vibrio metschnikovii	Bacterium		Colistin
Vibrio mimicus	Bacterium		Colistin
Vibrio parahaemolyticus	Bacterium		Colistin
Vibrio succinogenes	Bacterium (synonym for Wolinella succinogenes)		
Vibrio trachuri	Bacterium (synonym for Vibrio harveyi)		Colistin
Vibrio vulnificus	Bacterium		Colistin
Virgibacillus	Bacterium	Yes	
Virgibacillus halodenitrificans	Bacterium	Yes	
Virgibacillus pantothenticus	Bacterium	Yes	
Virgibacillus salexigens	Bacterium	Yes	
Viridans streptococci	Bacterium		
Wangiella	Fungus (synonym for Exophiala)		
Wangiella dermatitidis	Fungus (synonym for Exophiala dermatitidis)		
Wautersia	Bacterium		
Wautersia gilardii	Bacterium (synonym for Cupriavidus gilardii)		
Wautersia paucula	Bacterium (synonym for Cupriavidus pauculus)		
Weeksella	Bacterium		
Weeksella virosa	Bacterium		
Weeksella zoohelcum	Bacterium (synonym for Bergeyella zoohelcum)		
Weissella	Bacterium		
Weissella confusa	Bacterium		
Weizmannia coagulans	Bacterium (synonym for Bacillus coagulans)	Yes	
West Nile virus	Virus (synonym for Orthoflavivirus nilense)		
Western equine encephalitis virus	Virus (synonym for Alphavirus western)		
Wickerhamiella	Fungus		
Wickerhamiella pararugosa	Fungus		
Winkia neuii	Bacterium	Yes	
Wolinella	Bacterium		
Wolinella curva	Bacterium (synonym for Campylobacter curvus)		
Wolinella recta	Bacterium (synonym for Campylobacter rectus)		
Wolinella succinogenes	Bacterium		
Xanthomonas	Bacterium		
Xanthomonas maltophilia	Bacterium (synonym for Stenotrophomonas maltophilia)		
Xenoacremonium recifei	Fungus		
Xenorhabdus	Bacterium		3GCR, Carbapenems, Colistin
Xenorhabdus luminescens	Bacterium (synonym for Photorhabdus luminescens)		3GCR, Carbapenems, Colistin
Xenorhabdus nematophila	Bacterium		3GCR, Carbapenems, Colistin
Yarrowia lipolytica	Fungus		
Yatapoxvirus tanapox	Virus		
Yellow fever virus	Virus (synonym for Orthoflavivirus flavi)		
Yersinia	Bacterium		3GCR, Carbapenems, Colistin
Yersinia aldovae	Bacterium		3GCR, Carbapenems, Colistin
Yersinia aleksiciae	Bacterium		3GCR, Carbapenems, Colistin

Name	Type	Common Commensal	Recorded Resistances
Yersinia bercovieri	Bacterium		3GCR, Carbapenems, Colistin
Yersinia canariae	Bacterium		3GCR, Carbapenems, Colistin
Yersinia enterocolitica	Bacterium		3GCR, Carbapenems, Colistin
Yersinia frederiksenii	Bacterium		3GCR, Carbapenems, Colistin
Yersinia intermedia	Bacterium		3GCR, Carbapenems, Colistin
Yersinia kristensenii	Bacterium		3GCR, Carbapenems, Colistin
Yersinia mollaretii	Bacterium		3GCR, Carbapenems, Colistin
Yersinia pestis	Bacterium		3GCR, Carbapenems, Colistin
Yersinia pseudotuberculosis	Bacterium		3GCR, Carbapenems, Colistin
Yersinia rohdei	Bacterium		3GCR, Carbapenems, Colistin
Yersinia ruckeri	Bacterium		3GCR, Carbapenems, Colistin
Yokenella	Bacterium		3GCR, Carbapenems, Colistin
Yokenella regensburgei	Bacterium		3GCR, Carbapenems, Colistin
Zasmidium cerophilum	Fungus		
Zika virus	Virus (synonym for Orthoflavivirus zikaense)		

References

- [NHSN]

  NHSN Organism List: https://www.cdc.gov/nhsn/xls/master-organism-com-commensals-lists.xlsx

  Source: Centers for Disease Control and Prevention National Healthcare Safety Network (NHSN)

  Available on the NHSN website for no charge.

  Reference to specific commercial products, manufacturers, companies, or trademarks does not constitute its endorsement or recommendation by the U.S. Government, Department of Health and Human Services, or Centers for Disease Control and Prevention.

  Not subject to copyright but some requirements must be followed.

- [LPSN]

  List of Prokaryotic names with Standing in Nomenclature: https://lpsn.dsmz.de/

  Parte, A.C., Sardà Carbasse, J., Meier-Kolthoff, J.P., Reimer, L.C. and Göker, M. List of Prokaryotic names with Standing in Nomenclature (LPSN) moves to the DSMZ. *International Journal of Systematic and Evolutionary Microbiology*, **Volume 70, Issue 11**, 23 July 2020, Pages 5607-5612; DOI: 10.1099/ijsem.0.004332

  Licensed under the Creative Commons Attribution-NonCommercial 4.0 International (CC BY-NC 4.0) license.

- [MycoBank]

  MycoBank database: https://www.mycobank.org/

  Vincent Robert, Duong Vu, Ammar Ben Hadj Amor, Nathalie van de Wiele, Carlo Brouwer, Bernard Jabas, Szaniszlo Szoke, Ahmed Dridi, Maher Triki, Samy ben Daoud, Oussema Chouchen, Lea Vaas, Arthur de Cock, Joost A. Stalpers, Dora Stalpers, Gerard J.M. Verkley, Marizeth Groenewald, Felipe Borges dos Santos, Gerrit Stegehuis, Wei Li, Linhuan Wu, Run Zhang, Juncai Ma, Miaomiao Zhou, Sergio Pérez Gorjón, Lily Eurwilaichitr, Supawadee Ingsriswang, Karen Hansen, Conrad Schoch, Barbara Robbertse, Laszlo Irinyi, Wieland Meyer, Gianluigi Cardinali, David L. Hawksworth, John W. Taylor, and Pedro W. Crous. MycoBank gearing up for new horizons. *IMA Fungus*, **Volume 4, No 2**, 17 December 2013, Pages 371–379; DOI: 10.5598/imafungus.2013.04.02.16

  Licensed under the Creative Commons Attribution-NonCommercial-NoDerivatives 4.0 International (CC BY-NC-ND 4.0) license.

- [ICTV]

  International Committee on Taxonomy of Viruses (ICTV) database: https://ictv.global/taxonomy/

Lefkowitz EJ, Dempsey DM, Hendrickson RC, Orton RJ, Siddell SG, Smith DB. Virus taxonomy: the database of the International Committee on Taxonomy of Viruses (ICTV) *Nucleic Acids Research*, **Volume 46, Issue D1**, 4 January 2018, Pages D708–D717, DOI: 10.1093/nar/gkx932

Licensed under the Creative Commons Attribution-ShareAlike 4.0 International (CC BY-SA 4.0) license.
